# An intramolecularly self-templated synthesis of macrocycles: self-filling effects on the formation of prismarenes†

**DOI:** 10.1039/d1sc02199k

**Published:** 2021-06-03

**Authors:** Paolo Della Sala, Rocco Del Regno, Luca Di Marino, Carmela Calabrese, Carmine Palo, Carmen Talotta, Silvano Geremia, Neal Hickey, Amedeo Capobianco, Placido Neri, Carmine Gaeta

**Affiliations:** Dipartimento di Chimica e Biologia, “A. Zambelli” Università di Salerno Via Giovanni Paolo II I-84084 Fisciano Italy cgaeta@unisa.it; Centro di Eccellenza in Biocristallografia, Dipartimento di Scienze Chimiche e Farmaceutiche Università di Trieste Via L. Giorgieri 1 I-34127 Trieste Italy sgeremia@units.it

## Abstract

Ethyl- and propyl-prism[6]arenes are obtained in high yields and in short reaction times, independent of the nature and size of the solvent, in the cyclization of 2,6-dialkoxynaphthalene with paraformaldehyde. **PrS[6]Et** or **PrS[6]nPr** adopt, both in solution and in the solid state, a folded cuboid-shaped conformation, in which four inward oriented alkyl chains fill the cavity of the macrocycle. On these bases, we proposed that the cyclization of **PrS[6]Et** or **PrS[6]nPr** occurs through an intramolecular thermodynamic self-templating effect. In other words, the self-filling of the internal cavity of **PrS[6]Et** or **PrS[6]nPr** stabilizes their cuboid structure, driving the equilibrium toward their formation. Molecular recognition studies, both in solution and in the solid state, show that the introduction of guests into the macrocycle cavity forces the cuboid scaffold to open, through an induced-fit mechanism. An analogous conformational change from a closed to an open state occurs during the *endo*-cavity complexation process of the pentamer, **PrS[5]**. These results represent a rare example of a thermodynamically controlled cyclization process driven through an intramolecular self-template effect, which could be exploited in the synthesis of novel macrocycles.

## Abstract 2

Ethyl- and propyl-prism[6]arenes are obtained by an intramolecular thermodynamic self-template effect: the self-filling of the internal cavity stabilizes their cuboid structure, driving the equilibrium toward their formation.

## Graphical abstract

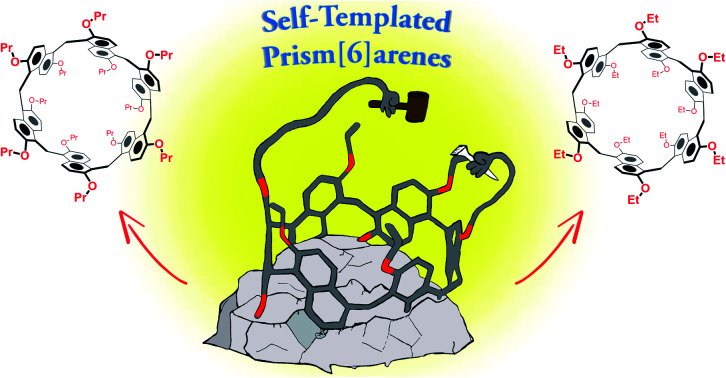

## Introduction

Since the birth of supramolecular chemistry, the field of macrocyclic hosts and related host–guest complexes has grown considerably.^1,2^ The internal cavity of macrocyclic hosts is reminiscent of the binding sites of natural bioreceptors and for this reason macrocycles have been the focus of biomimetic chemistry research.^3^ The equilibrium of inclusion of a guest inside the host cavity is thermodynamically influenced by many factors, such as host–guest desolvation processes, ion pair rupture, conformational control of the host and guest, and host/guest weak interactions.^4,5^ The strength of such non-covalent interactions in guest@host complexes plays a crucial role in overcoming the other unfavorable thermodynamic factors.^4–6^ In general, an extended contact area^4,5^ between the host and guest and a high level of preorganization of the host can lead to stronger complexation*.* Aromatic cyclophane^7^ macrocycles, such as calixarenes,^8^ resorcinarenes,^7^ and pillararenes,^9^ show a variety of deep cavities that can wrap the included guest. Recently, naphthol-based macrocycles with a deeper π-cavity have generated a great deal of interest. Thus, oxatubarenes, naphthotubes, and naphthocages were reported by Jiang and coworkers, which show interesting conformational properties and recognition abilities.^10–16^

**Fig. 1:**
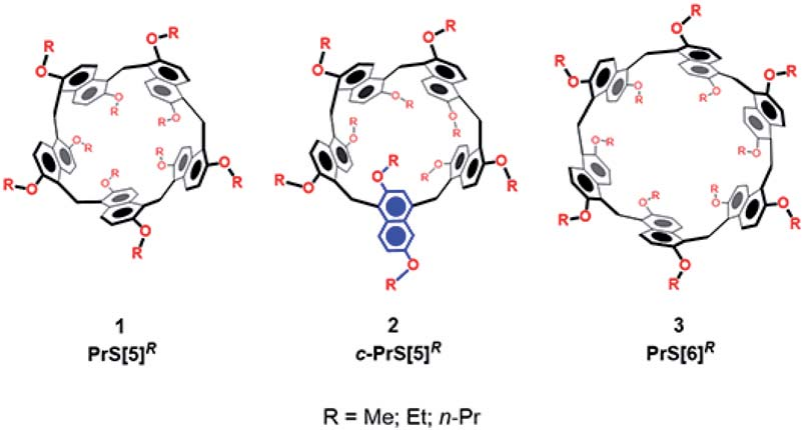
The prism[*n*]arene family.

**Scheme 1:**
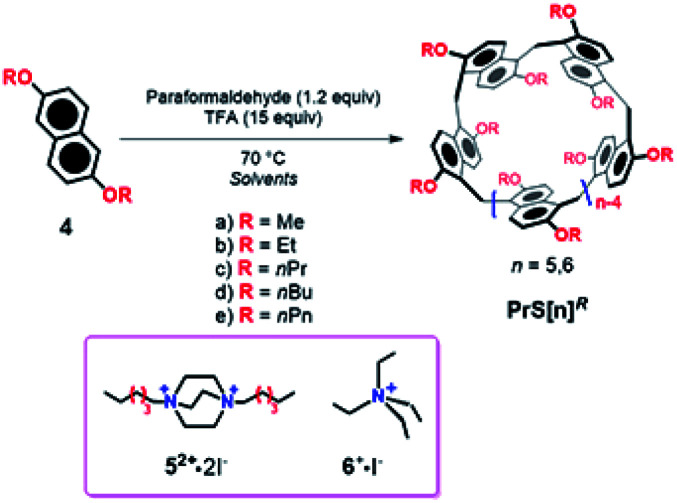
Synthesis of **PrS[n]R** prismarenes.

The scientific interest toward the design of new macrocyclic hosts^17^ with deep cavities and conformational adaptive behavior^16^ has recently prompted us to synthesize new examples of naphthol-based macrocycles named calix[2]naphth[2]arene^18^ and prismarenes (Fig. 1).^19,20^ The first report on prismarenes was mainly focused on the synthesis and recognition properties of the pentameric **PrS[5]Me** macrocycle (Fig. 1) constituted by 1,5-methylene bridged 2,6-dimethoxynaphthalene units.^19^ With the aim to explore the supramolecular properties of the larger hexameric **PrS[6]** macrocycle, we decided to investigate its efficient synthesis by changing the solvent, templating agents, and alkoxy-chains. In this way, a rare and peculiar intramolecularly self-templated synthesis was discovered. 2,6-Dimethoxynaphthalene-based prism[*n*]arenes **PrS[n]Me** (Fig. 1) were obtained by a thermodynamically controlled cyclization process (Scheme 1).^21*a*,22^

Starting from **4a**, under the conditions reported in Scheme 1, the isomeric prismarenes **PrS[5]Me** and **c-PrS[5]Me** (the isomer with one 1,4 methylene bridge unit) were, respectively, the kinetic and the thermodynamic adduct.

Specifically, the pentamer **PrS[5]Me** was formed faster than its confused isomer **c-PrS[5]Me**, and DFT calculations confirmed that the transition state for the formation of the confused isomer was higher in energy than that for **PrS[5]Me**.^19^ In addition, DFT calculations confirmed the crucial role played by the solvent 1,2-DCE in the macrocyclization steps for the formation of the two isomeric **PrS[5]Me** and **c-PrS[5]Me** pentamers.^19^ Under the conditions reported in Scheme 1, and in the presence of the solvent 1,2-DCE, the most stable product **c-PrS[5]Me** prevailed over time (Table 1, entry 1). When the template **52+** or **6+** (Scheme 1 and Table 1, entries 2 and 3) was added to the reaction mixture in 1,2-DCE, the formation of *endo*-cavity inclusion complexes, **52+@PrS[5]Me** or **6+@PrS[6]Me**, respectively, stabilized the desired product *via* supramolecular interactions,^19^ and shifted the equilibrium toward **PrS[5]Me** (Table 1, entry 2) or **PrS[6]Me** (Table 1, entry 3), respectively (thermodynamic templating^21*b*^).^19^ The crucial factor which determines the greater thermodynamic stability of **c-PrS[5]Me** in the equilibrium mixture (Scheme 1), in the absence of guest **52+** or **6+**, is its ability to self-fill the internal cavity.^19^ In fact, the 1,4-naphthalene ring assumes an orientation in which the 2-OMe group is inward oriented with respect to the cavity and completely fills it.^19^

**Table 1:** Solvent and alkoxy-chain effects in the synthesis of **PrS[n]R**

Entry^a^	Solvent	Monomer	Time	**c-PrS[5]R**	**PrS[5]R**	**PrS[6]R**
1	1,2-DCE	**4a** (ref. 19)	22 h	40	0.3	—
2	1,2-DCE	**4a** (ref. 19)^b^	22 h	16	47	—
3	1,2-DCE	**4a** (ref. 19)^c^	72 h	6.0	0.3	20
4	1,2-DCE	**4b**	45 min	—	—	75
5	1,2-DCE	**4c**	90 min	—	—	65
6	1,2-DCE	**4b** b	22 h	—	10	35
7	1,2-DCE	**4b** c	22 h	—	—	60
8	1,2-DCE	**4c** b	22 h	—	25	25
9	1,2-DCE	**4c** c ^,^ d	22 h	—	—	55
10	1,2-DCE	**4d**	40 min	—	—	30
11	1,2-DCE	**4e**	12 h	—	—	8
12	Cl-CyHex	**4b**	40 min	—	—	71
13	Cl-CyHex	**4c**	2 h	—	—	64
14	Toluene	**4a**	24 h	11	—	—
15	Toluene	**4b**	3 h	—	—	74
16	Toluene	**4c**	8 h	—	—	57
17	CyHex	**4a**	24 h	7	—	—
18	CyHex	**4b**	30 min	—	—	76
19	CyHex	**4c**	40 min	—	—	65
20	Decaline	**4b**	30 min	—	—	75
21	Decaline	**4c**	45 min	—	—	70

a [Monomer] = 5 mM; TFA and paraformaldehyde; 70 °C.

b In the presence of **52+**.

c In the presence of **6+**.

d In the presence of **72+** (see Scheme 2) as an iodide salt; derivatives **PrS[5]nPr** and **PrS[6]nPr** were obtained in 10% and 47% yield, respectively.

Considering these results, the question arises as to whether the pentamers and their confused isomers are still the kinetic and thermodynamic products, respectively, when starting from **4b–e** bearing longer alkoxy groups. In other words, can the length of alkoxy substituents influence the synthesis, the conformational, and supramolecular properties of prismarenes?

## Results and discussion

### Synthesis of ethoxy- and propoxy-prismarenes

As previously reported, when methoxy-derivative **4a** (ref. 19) was reacted in the presence of paraformaldehyde and TFA in 1,2-DCE as the solvent, **c-PrS[5]Me** and its **PrS[5]Me** isomer were formed in 40% and 0.3% yield (Table 1, entry 1), respectively, while no trace of **PrS[6]Me** hexamer was detected in the reaction mixture.^19^ We showed that under these conditions, **c-PrS[5]Me** was the thermodynamic product, while **PrS[5]Me** was the kinetic one and its formation was templated by the 1,2-DCE solvent. Thus, under these conditions, 1,2-DCE didn't act as a template for the prism[6]arene methoxy derivative.^19^ Differently, when 2,6-diethoxynaphthalene **4b** was reacted in 1,2-DCE (Table 1, entry 4), unexpectedly, the **PrS[6]Et** hexamer was formed in a very high yield (75%) after 45 min. Analogously, when 2,6-dipropoxynaphthalene **4c** was used as the precursor, the **PrS[6]nPr** hexamer was again the favored product (65% yield) after 90 min (Table 1, entry 5). These results were unexpected because, according to the data previously reported by Ogoshi^22^ and by us,^19^ the 1,2-DCE solvent does not play any role as a template for the larger pillar[6]arene and prism[6]arene methoxy-derivatives. The formation of ethoxy- and propoxy-prismarenes **PrS[n]R** (*n* = 5 and 6, R = Et and *n*Pr) as described in Scheme 1 and Table 1 was monitored as a function of time by HPLC (ESI†). The formation of the cyclic oligomers proceeded through many linear intermediate compounds that, after being initially formed, were then consumed as the cyclization reaction progressed.

In the case of **4b** in 1,2-DCE (Scheme 1 and Table 1, entry 4), **PrS[6]Et** was formed faster than the pentamer **PrS[5]Et** (ESI†). In fact, the hexamer started to appear after 10 min and remained the favored product over time, whereas an equilibrium value of 75% was reached after 45 min. HPLC analysis (ESI†) revealed that the pentamer appeared after 15 min but vanished after 30 min. Analogous results were obtained by monitoring the formation of **PrS[6]Et** and **PrS[5]Et** over time using different solvents (ESI†), such as chloro-cyclohexane (Cl-CyHex, Table 1, entry 12), cyclohexane (CyHex, Table 1, entry 18), toluene (Table 1, entry 15), and decaline (Table 1, entry 20). Independent of the nature and size of the solvent, the hexamer **PrS[6]Et** is always kinetically and thermodynamically favored with respect to **PrS[5]Et**, and its formation occurs in very high yields (71–76%) in short reaction times (30–45 min). Similar results were observed for the reaction in Scheme 1 starting from propoxy precursor **4c**. In this case, the hexamer **PrS[6]nPr** is the kinetically and thermodynamically favored product independent of the nature of the solvent (Table 1, entries 5, 13, 16, 19, and 21). These results are surprising; in fact, as reported in the literature, only halogenated solvents play a fundamental role as templates in the formation of π-electron rich macrocycles as in the synthesis of pillararenes.^22,23^ To confirm the thermodynamic nature of the hexamers, **PrS[5]Et** or **PrS[5]nPr** (*vide infra*) was heated to 70 °C in 1,2-DCE, in the presence of TFA. Under these conditions, a conversion to **PrS[6]Et** and **PrS[6]nPr** in 83% and 85% yield, respectively, was observed after 22 h, confirming their higher thermodynamic stability.

As previously reported,^19^ starting from the methoxy-derivative **4a** in 1,2-DCE **c-PrS[5]Me** is preferentially formed, while no evidence of the hexamer was detected in the reaction mixture (Table 1, entry 1). Therefore, we further investigated whether **PrS[6]Me** would be formed using the more bulky cyclohexane or toluene as the solvent. Starting from methoxy-derivative **4a** in toluene, the pentamer **c-PrS[5]Me** was formed in a very low yield (11%, Table 1, entry 14) in addition to a copious quantity of polymeric material. Analogous results were obtained in cyclohexane (7% of **c-PrS[5]Me**, Table 1, entry 17).

Under these conditions, no hexamer **PrS[6]Me** was detected. When the reaction in Scheme 1 was performed using a monomer with even longer alkyl chains than propyl, namely 2,6-dibutoxy- and 2,6-dipentoxynaphthalene **4d** and **4e** in 1,2-DCE, hexamers **PrS[6]nBu** and **PrS[6]nPent** were formed in lower yields of 30% and 8%, respectively (Table 1, entries 10 and 11). Thus, in summary, in the presence of small methoxy groups, the hexamer **PrS[6]Me** is not formed in any solvent (CyHex, toluene, or 1,2-DCE), whereas in the presence of ethoxy and propoxy groups the hexamer **PrS[6]R** is always formed in high yields (75 and 65%) independent of the nature and size of the solvent. Interestingly, the yield of prism[6]arene decreases (from 75% to 8%) as the chain length increases (entries 4, 5, 10, and 11) from ethoxy (**4b**, entry 4, Table 1) to pentoxy (**4e**, entry 11, Table 1). In addition, the pentamer **PrS[5]Me** is the kinetically favored product while its confused isomer prevails under thermodynamic conditions. Differently, in the presence of ethoxy and propoxy functions the hexamers **PrS[6]R** are both kinetically and thermodynamically favored products. These results led us to conclude that the length of the alkyl chains on the rims of prismarenes plays a “special role”, driving their cyclization process. In detail, we can invoke a kinetically and thermodynamically self-templating effect by the Et and *n*Pr alkyl chains in the synthesis of prismarene hexamers. At this point, we studied the role of the ammonium templates **52+** and **6+** in the formation of the ethoxy and propoxy prismarenes. In fact, we previously showed that the cation **52+** acted as a template for the synthesis of the methylated pentamer **PrS[5]Me** (47%, Table 1, entry 2).^19^ Differently, starting from **4b**, under the conditions in Scheme 1 and in the presence of **52+**·**2I−**, the hexamer **PrS[6]Et** was preferentially formed in 35% yield after 22 h (thermodynamic conditions), while the pentamer **PrS[5]Et** was obtained in 10% yield (Table 1, entry 6). HPLC analysis of the reaction mixture (Scheme 1) (ESI†) showed that after 60 min the hexamer was formed faster than **PrS[5]Et**, which was not detected in the reaction mixture. After 70 min, **PrS[6]Et** started to decrease slightly, while **PrS[5]Et** increased.

Starting from propoxy derivative **4c**, the hexamer was still the kinetically favored product, while after 3 h the concentration of the pentamer **PrS[5]nPr** started to increase, and after 22 h an equilibrium value of 1/1 was reached (Table 1, entry 8). By comparison between the data in Table 1 (entry 2)^19^ and the new results (entries 6 and 8), we can conclude that the cation **52+** is surely less effective as a thermodynamic template in the synthesis of ethoxy and propoxy pentamers. In detail, the thermodynamic effect of the host–guest complexation invoked for the synthesis of **PrS[5]Me**,^19^ is overcome by the intramolecular “self-templating” effect of the ethyl and propyl chains. At this point, we investigated the role of **6+** in the synthesis of **PrS[6]Et** starting from **4b** in 1,2-DCE (Scheme 1).

As previously reported,^19^**6+** is an effective template for the synthesis of **PrS[6]Me** (Table 1, entry 3).^19^ We monitored the progress of the reaction, and in the presence of **6+**·**I−** after 45 min the yield of **PrS[6]Et** was <10%, significantly lower than that obtained after the same time (75%, Table 1, entry 4) in the absence of **6+**. After 22 h (Table 1, entry 7), the yield of **PrS[6]Et** reached a value of 60%. Analogous results were observed starting from **4c** in 1,2-DCE in the presence of **6+**·**I−** that gave **PrS[6]nPr** in 55% yield after 22 h (Table 1, entry 9), while after 90 min the yield of **PrS[6]nPr** was <10%, once again significantly lower than that obtained after the same time in the absence of **6+** (65%, Table 1, entry 5). From the analysis of these data, it emerges that, in contrast to the clear cation templating effect on the formation of the methylated-analogue **PrS[6]Me** (Table 1, entry 3),^19^ the ammonium cation **6+** has a negative influence on the kinetics of the formation of prismarene hexamers **PrS[6]Et** and **PrS[6]nPr**. This is demonstrated by lower yields over longer reaction times. In other words, kinetically and thermodynamically self-templating by the Et and *n*Pr alkyl chains in the synthesis of prismarene hexamers is verified by the higher yield (65–75%) of the product and a shorter reaction time, in the absence of cations. With these results in hand, we investigated the nature of the self-templating effect from the alkyl chains by detailed X-ray, DFT, and 2D NMR studies.

### Structural properties of ethoxy- and propoxy-prismarenes in the solid state: the role of the alkyl chains

**Fig. 2:**
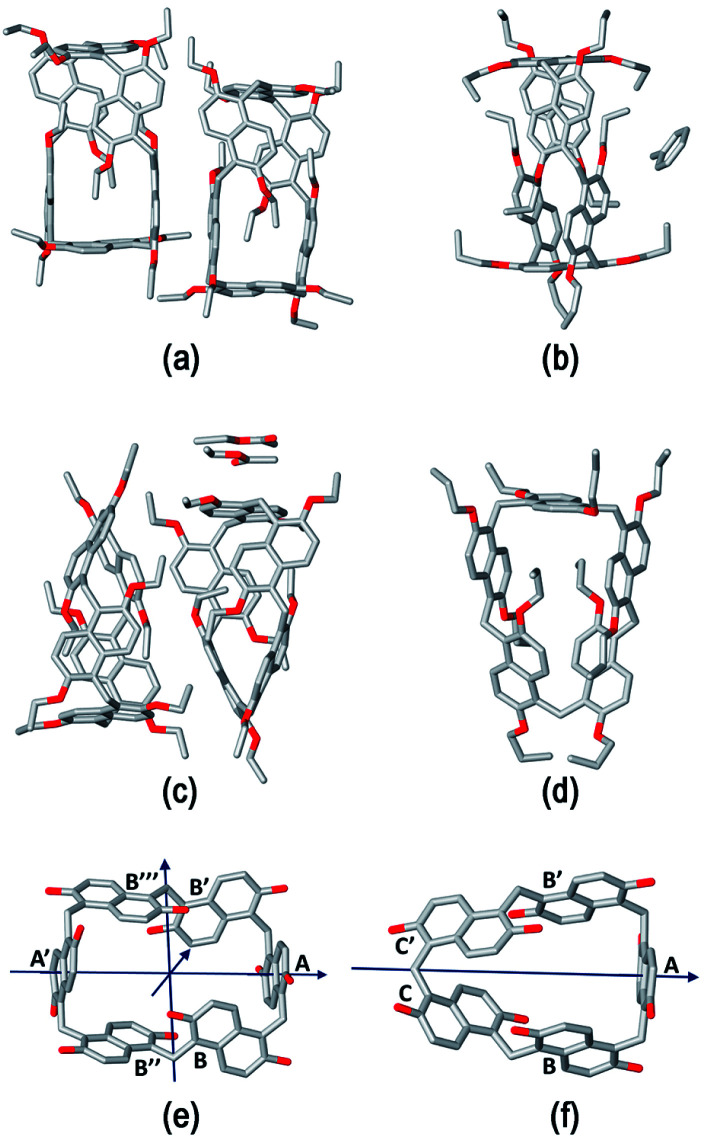
Stick representation of the asymmetric units of (a) **PrS[6]Et**, (b) **PrS[6]nPr**, (c) **PrS[5]Et**, and (d) **PrS[5]nPr**. Hydrogen atoms and one orientation of the disordered alkyl groups and a toluene molecule are omitted for clarity. For **PrS[6]nPr**, the entire molecule generated by a crystallographic two-fold axis is shown. (e) and (f) Stick representation of (e) the *D*_2_ symmetric **PrS[6]R** and (f) the *C*_2_ symmetric **PrS[5]R** scaffolds. The pseudo-two-fold symmetry axes are represented with arrows. For each molecule, the same letter indicates symmetry equivalent naphthalene rings. The alkyl groups are omitted for clarity.

**Fig. 3:**
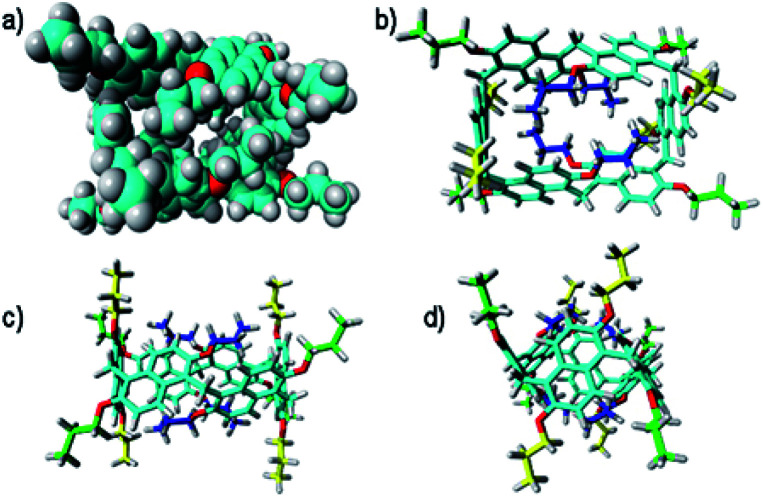
Different views of the DFT optimized structure of **Pr[6]nPr**. Top views of the CPK (a) and tube (b) structure. Side views (c) and (d). In blue are the propoxy chains inward oriented and filling the cavity, in green are the propoxy chains outside oriented, and in yellow are the propoxy chains on the parallel naphthalene rings.

The structure assumed by prismarenes were studied experimentally, both in solution and in the solid state, by single crystal X-ray diffraction (Fig. 2) and NMR techniques (ESI†), respectively, and theoretically by DFT calculations (Fig. 3 and ESI†).

Detailed structural models of the series **PrS[6]R** and **PrS[5]R** (R = Et and *n-*Pr) were obtained by single crystal X-ray diffraction experiments (Fig. 2 and ESI†) using synchrotron radiation and cryo-cooling techniques (see the ESI†). All four centrosymmetric crystal structures are composed of a racemic mixture of inherently^24–27^ chiral macrocyclic molecules, in which the naphthalene moieties are 1,5 connected, with the alkoxy groups at the 2,6-positions (Fig. 2). The ethyl derivatives, **PrS[6]Et** (Fig. 2a) and **PrS[5]Et** (Fig. 2c), show two crystallographic independent molecules in the asymmetric units, while the propyl derivatives, **PrS[6]nPr** (Fig. 2b) and **PrS[5]nPr** (Fig. 2d), contain a half molecule which lies on a two-fold crystallographic axis and one entire macrocyclic molecule, respectively. The prism[6]arene derivatives show a common dissymmetric conformation for the independent molecules, with a pseudo-*D*_2_ point symmetry of the macro-ring (Fig. 2e).

The DFT-optimized **PrS[6]nPr** molecule (Fig. 3) is very similar to the corresponding experimental solid-state structure as demonstrated by a rmsd value of 0.34 Å (ESI†) obtained by superimposing the two macrocyclic models (excluding the mobile alkyl chains).

The macro-ring is folded into a nearly square cuboid-shape (Fig. 2a, e and 3a) with the side-faces defined by the six naphthalene planar groups. Each aromatic planar group makes an angle of about 90° with respect to the contiguous naphthalene moieties (see ESI, Table S6†). Two opposite naphthalene rings (A and A′ of Fig. 2e at a 12.0 Å distance) define the square faces of the prism with about 6.5 Å side lengths. A twofold symmetry axis passes through the centers of these naphthalene rings. Thus, these two naphthalene units show a canting angle *θ* value of about 90° (see ESI, Table S5†).^28^ A second orthogonal twofold axis passes through two opposite methylene bridges of naphthalene moieties located on two edges of elongated faces of the parallelepiped. These naphthalene moieties show supplementary canting angles of *ca.* 40°/140° for the B/B′′ and B′′′/B′ couples (Fig. 2e and Table S5†). The third orthogonal twofold axis passes through the two open edges of the prism (Fig. 2e). The four inward oriented alkyl chains of the naphthalene moieties located on the elongated faces of the prism fill the cavity of the macrocycle (Fig. 3). These chains, organized in couples oriented in an antiparallel fashion, establish C–H⋯π interactions with the aromatic walls and various inter-chain van der Waals interactions (Fig. 3).

In detail, four strong C–H⋯π interactions are present with a C–H⋯π^centroid^ distance of 2.9 Å and a C–H⋯π^centroid^ angle of 148°. Clearly, this self-filling of the central cavity of the hexamer (Fig. 3) plays a crucial role in the thermodynamic stabilization of the hexameric skeleton, and it is relevant with respect to the conformational properties in solution and recognition abilities within the family **PrS[6]nPr**, **PrS[6]Et**, and **PrS[6]Me**. In addition, the inward oriented alkyl chains enhance the rigidity of the cuboid scaffold, as evidenced by NMR studies (*vide infra*). All three independent molecules of the prism[5]arene derivatives show a pseudo-*C*_2_ point symmetry of the macro-ring and therefore a similar dissymmetric conformation (Fig. 2c, d and f). This pseudo-symmetry was also observed in the three pseudo-polymorphic crystal forms of **PrS[5]Me**.^19^

The superimposition of the DFT-optimized **PrS[5]nPr** molecule with the solid-state structure shows a rmsd value of 0.07 Å. The pseudo-twofold symmetry axis passes through the methylene bridge connecting the C and C′ rings and the barycenter of the opposite naphthalene moiety (A in Fig. 2f). The canting angles reported in Table S5† reflect this situation. The dihedral angles of the B/B′ and C′/C couples (see Fig. 2f) are supplementary angles (*ca.* 70°/110° for B/B′ and *ca.* 40°/140° for C′/C), while the A angle is *ca.* 90°. In this way, two adjacent naphthalene rings (ESI†) are tilted in a way to give a self-filling of the cavity with their chains (ESI†). To gain insight into the structural features of the potential cavities of the prism[*n*]arenes (*n* = 5 and 6), the surfaces and volumes of the ideal prismatic solids enclosed by the aromatic walls were evaluated (ESI† shows the comparison with the analogous pillararene and pagodarene^17^). The geometrical parameters used for these calculations were obtained from the CCDC database as mean values of distances and angles observed in the X-ray structures containing the relevant monomeric unit (see Fig. S107† for details).

The prism[6]arene shows a cavity opening of 52.4 Å^2^ almost twice that (27.3 Å^2^) of the prism[5]arene, and, analogously, a 2 : 1 volume cavity ratio was calculated for the two prisms (490 Å^3^/255 Å^3^). Another important geometric feature is the internal potential contact surface areas (*A*) derived from the total area of the rectangular prism faces. The ratio between the contact surface areas of prism[6]arene and prism[5]arene is 252 Å^2^/186 Å^2^ = 1.35.

### Conformational properties of ethoxy- and propoxy-prismarenes in solution: the role of the alkyl chains

The 1D and 2D NMR spectra (243 K, 600 MHz) of **PrS[6]nPr** (Fig. S20† and 4a) in CD_2_Cl_2_ are consistent with the *D*_2_ symmetry of the cuboid structure found in the solid state.

**Fig. 4:**
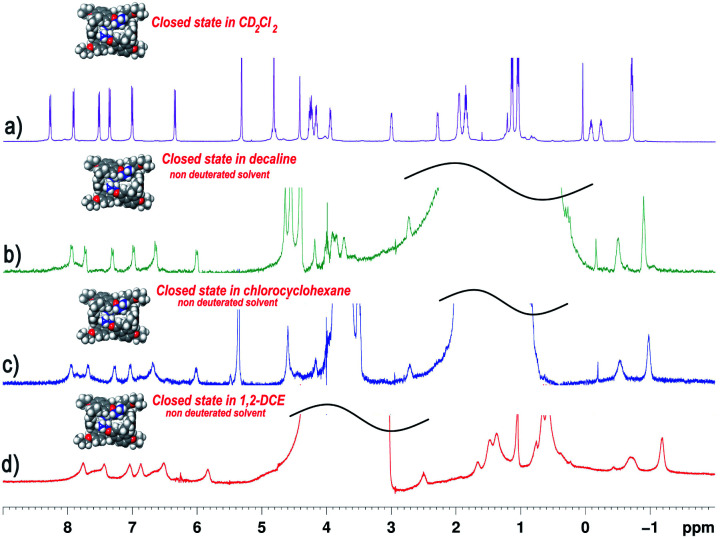
^1^H NMR spectra of **Pr[6]nPr** in: (a) CD_2_Cl_2_ at 243 K (600 MHz); (b) non-deuterated decaline (298 K); (c) non-deuterated chlorocyclohexane (298 K); (d) non-deuterated 1,2-dichloroethane (298 K). The spectra are consistent with the *D*_2_ symmetry of the cuboid structure of the prism[6]arene.

**Fig. 5:**
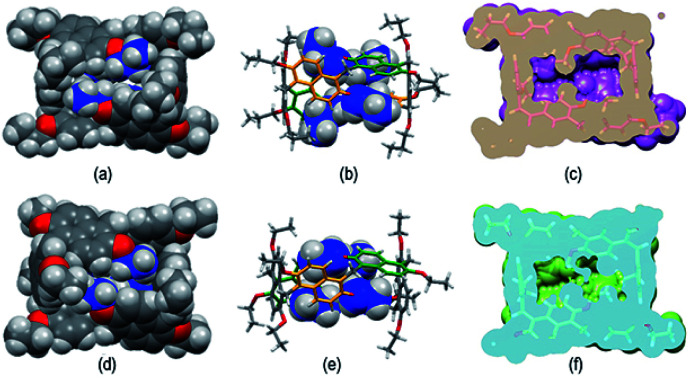
Self-filling of the cavities of prism[6]arenes as obtained by X-ray structures. (a) and (b) Different views of **PrS[6]nPr**. (d) and (e) Different views of **PrS[6]Et**. (c) and (f) Self-filling of the cuboid cavity. Cross sections of the **PrS[6]Et** (f) and **PrS[6]nPr** (c) molecules. An enclosed internal cavity was obtained from accessible surface area calculations using a 0.4 Å probe. The free cavity volume is 49 Å^3^ and 77 Å^3^ for **PrS[6]Et** and **PrS[6]nPr**, respectively.

In fact, a close inspection of the ^1^H NMR spectrum of **PrS[6]nPr** revealed the presence of 3 aromatic AX/AB systems (COSY) at 8.28/7.35 (9.6 Hz), 7.91/7.01 (9.6 Hz), and 7.52/6.35 (9.6 Hz) ppm (Fig. S29c and e†), and two singlets at 4.82 and 4.42 ppm in a 2 : 1 ratio, attributable to the methylene-bridged groups. In addition, the OCH_2_ groups showed three diastereotopic AB systems at 4.25/4.23, 4.17/3.95, and 3.00/2.29 ppm (Fig. S29c, d and f†). The presence of diastereotopic resonances for the OCH_2_ groups of **PrS[6]nPr** (Fig. 4a and S29c, d, f†) is due to the planar chirality of the macrocycle, in agreement with the considerations previously reported by Ogoshi and coworkers^24^ for pillar[5]arenes. Thus, this 1D and 2D NMR analysis indicates that in solution **PrS[6]nPr** also adopts a conformation with a pseudo-*D*_2_ point symmetry. The presence of shielded ^1^H NMR signals at negative chemical shift values (Fig. 4a), attributable to the terminal methylene and methyl groups of the propyl-chains (CH_3_: −0.6 ppm, 12H, ESI†), clearly corroborates the finding that **PrS[6]nPr** also adopts in solution the cuboid structure depicted in Fig. 3a and 5a, in which there is a folding of the propyl chains inside the cavity. The 1D and 2D NMR spectra of **PrS[6]Et** show analogous features that confirm its cuboid structure in solution. A broad singlet is present in the ^1^H NMR spectrum of **PrS[6]Et** in CD_2_Cl_2_ at 298 K at −1.12 ppm (12H), attributable to the methyl groups belonging to the four ethyl groups folded inside the cavity. Impressively, these OCH_2_CH_3_ methyl groups experienced an up-field shift of △*δ* = *δ*_macrocycle_ − *δ*_monomer-_**4b** = −2.74 ppm, significantly larger than that shown by the methyl groups of **PrS[6]nPr** of −1.67 ppm. This is evident from the close inspection of the X-ray structures in Fig. 5a and d. In Fig. 5b, the terminal methyl group of the chains of **PrS[6]nPr** are outward oriented, while the four ethyl groups of **PrS[6]Et** are entirely included inside the cavity of the macrocycle. The CPK visualization of the ethyl chains in Fig. 5d clearly reveals that they are close-packed inside the cavity, and fill the inner space more efficiently than the propyl chains of **PrS[6]nPr** in Fig. 5b. To quantify the differences in the self-filling of **PrS[6]Et** and **PrS[6]nPr**, the free volume of each enclosed internal cavity was evaluated from accessible surface area calculations (see the ESI† and Fig. 5). The smaller cavity volume of 49 Å^3^ obtained for **PrS[6]Et** with respect to 77 Å^3^ for **PrS[6]nPr** indicates the more efficient packing of this ethyl derivative (Fig. 5c and f).

These results support the hypothesis that the nature of the alkyl chains plays a crucial role in the thermodynamically controlled cyclization of the hexamer. The cyclization process of the prism[6]arene hexamer is thermodynamically controlled by the alkyl chains on the rims and by their ability to fill the internal cavity of the macrocycle. In particular, the ethyl chains show a better steric complementarity with the internal cavity of the prism[6]arene and fill it in a more effective way (90%) than the propyl chains (84%). Consequently, **PrS[6]Et** is obtained in higher yields (about 75%) than **PrS[6]nPr** independent of the used solvent. Longer butyl and pentyl chains are less effective at self-filling the internal cavity and consequently the yield of the hexamer is lower.

Analogously, no evidence of the hexamer was detected in the reaction mixture starting from 2,6-dimethoxynaphthalene **4a**; in this case, the methyl groups is too small to produce an effective filling of the internal cavity of the hexamer. Consequently, the equilibrium of formation of the methoxy-prismarenes is shifted toward the confused prism[5]arene **c-PrS[5]Me**, which shows a more effective self-filling of the internal cavity. When the **52+** or **6+** cation is present, the filling of the **PrS[5]Me** or **PrS[6]Me** cavity with the formation of the host–guest complex stabilizes the pentamer or hexamer respectively, which remains the preferential product over time.

On these bases, we can invoke an intramolecular thermodynamic self-templating effect in which the formation of the prism[6]arene is not driven by an external templating agent (*e.g.*: solvent) but by “intramolecular self-templating”^29^ of the alkyl chains. In other words, the self-filling of the internal cavity of **PrS[6]Et** or **PrS[6]nPr** stabilizes their cuboid structure driving the equilibrium toward their formation. This represents a rare example of a documented thermodynamically controlled cyclization process driven through an intramolecular self-template effect.

Ogoshi reported that 1,2-DCE and chlorocyclohexane are thermodynamic template solvents in the synthesis of pillar[5]arenes.^22^ In detail, DCE fills the pillar[5]arene cavity and its equilibrium of formation is driven by the thermodynamic effect of the complexation, solvent@host.^22^ The same authors clearly showed that 1,2-DCE didn't play a template role in the synthesis of the pillar[6]arene.^22^ In an attempt to examine if the solvents in Table 1 play a thermodynamic templating role for the selective formation of prism[6]arenes, by an *endo*-cavity inclusion process (solvent@**PrS[6]R** complexation), we performed ^1^H NMR experiments following a standard procedure already reported for pillararenes.^22^ The cuboid structure of the prism[6]arene shows an inaccessible closed-cavity (closed state, Fig. 5a, b and d, e) and consequently a conformational change of the prism[6]arene should be expected during an eventual *endo*-cavity inclusion of the solvent (*vide infra*).

The ^1^H NMR spectra of **PrS[6]nPr** in Fig. 4b–d were acquired in non-deuterated solvents such as chloro-cyclohexane, decaline, and 1,2-DCE. As is evident, the ^1^H NMR spectra in these solvents agree with the *D*_2_-symmetry of the cuboid structure. This result clearly indicated that in the presence of these solvents, the prism[6]arene remains in a closed state (Fig. 4) and consequently the inclusion of solvent molecules inside the cavity doesn't occur. To confirm this assumption, ^1^H NMR experiments were performed in which **PrS[6]nPr** and **PrS[6]Et** were titrated with 1 equiv. of solvent such as cyclohexane, chloro-cyclohexane, toluene, decaline, and 1,2-DCE and in no case was the shift of the solvent signals observed (ESI†), confirming that the host cuboid structure was retained. From these results, it can be inferred that these solvents don't act as a thermodynamic template for prism[6]arene formation. These results are consistent with the finding that, independent of the nature and size of the solvent, the ethoxy- and propoxy-hexamers are always kinetically and thermodynamically favored with respect to the pentamer.

In order to investigate whether the solvent 1,2-DCE plays a role in the stabilization of the transition states (TSs) for the macrocyclization steps of the intermediate carbocation of **PrS[6]Et**, a preliminary DFT study (ESI†) was performed. The transition state holding the solvent molecule inside the cavity (ESI†) is predicted to lie above the one holding the ethyl group inside the cavity by 3.4 kcal mol^−1^ in terms of internal energy and 2.3 kcal mol^−1^ in terms of Gibbs free energy. Based on the reasonable hypothesis that a late transition state is involved in the rate-determining step, DFT results suggest that the ethyl group could indeed fill the cavity, thus acting as an internal kinetic templating agent.

### Molecular recognition properties of ethoxy- and propoxy-prism[*n*]arenes: the role of the alkyl chains

**Fig. 6:**
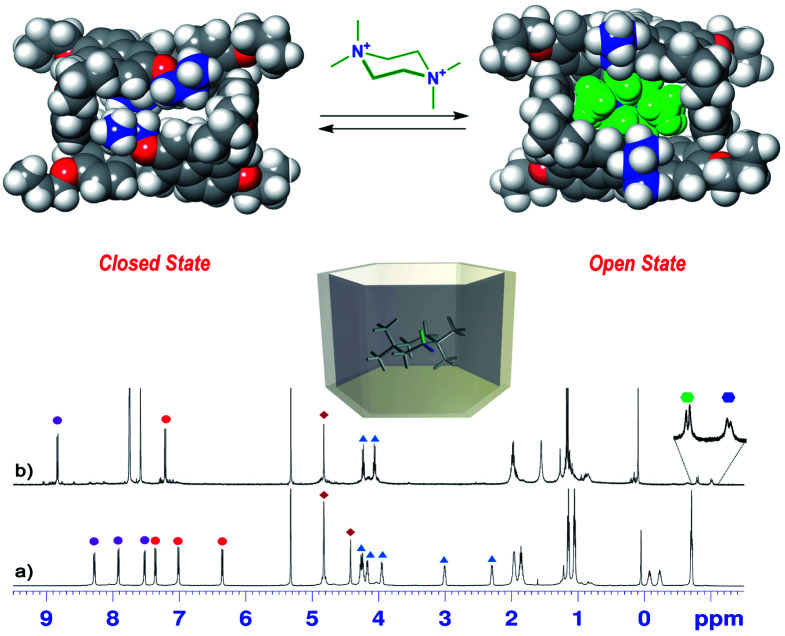
^1^H NMR spectra of (a) **PrS[6]nPr** in CD_2_Cl_2_ at 243 K (600 MHz) and (b) a 1 : 1 mixture of **PrS[6]nPr** and **72+**·(BArF)_2_ (3 mM). The correspondence between the marked signals (red and purple circles for aromatic H-atoms and blue triangles for OCH_2_ groups) indicates the conformational change from a closed state to an open state upon complexation with **72+**. The blue and green hexagons indicate the diastereotopic methylene H atoms of **72+** shielded inside the macrocycle. (Top) DFT optimized structure of **PrS[6]nPr** in the closed state and of its complex with **72+** (**72+@PrS[6]nPr**).

On the basis of these conformational aspects, a question arises as to whether **PrS[6]nPr** and **PrS[6]Et** are also capable of adopting a conformation with an open-cavity (open state) in the presence of appropriate guests. Due to the presence of π-electron rich cavities,^19^ it is worth investigating the recognition abilities of **PrS[6]nPr** and **PrS[6]Et** hosts toward cationic ammonium guests (Scheme 2). The complexation of the *N*,*N*,*N′*,*N′*-tetramethylpiperazonium **72+** cation (Scheme 2) showed very interesting features. The addition of 1 equiv. of **72+** as a barfate salt (BArF^−^)^30,31^ to a solution of **PrS[6]nPr** in CD_2_Cl_2_ caused significant changes in its ^1^H NMR spectrum (Fig. 6), indicative of the formation of a **72+@PrS[6]nPr** complex with the cation hosted inside the cavity of **PrS[6]nPr**.

**Scheme 2:**
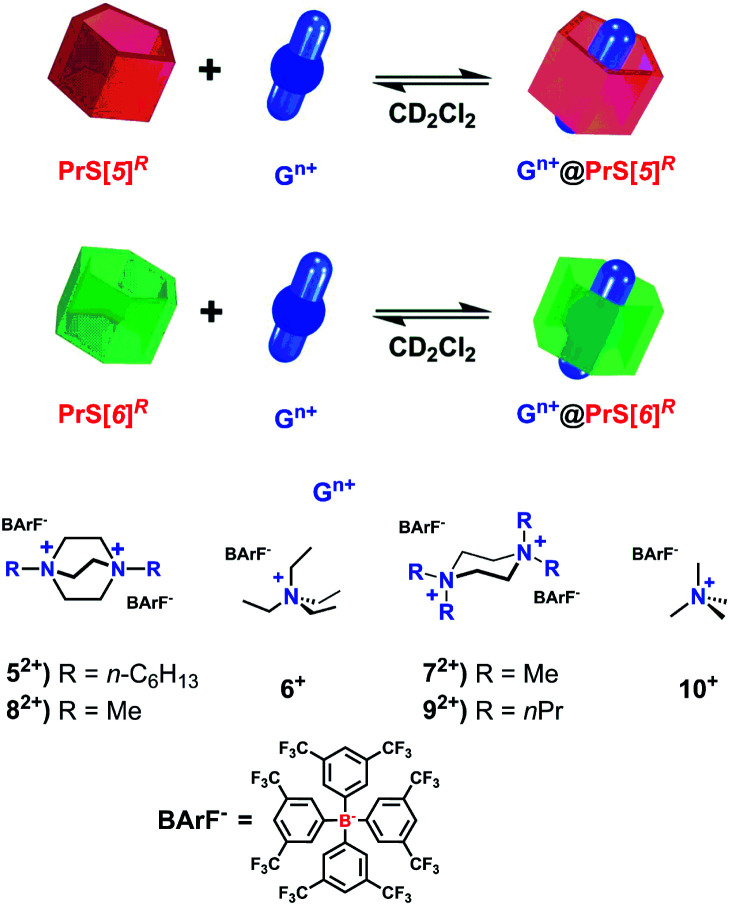
Schematic complexation equilibrium of **PrS[n]R** with ammonium guests **52+–10+** as barfate salts.

Furthermore, in the presence of **72+**, a more symmetric conformation of **PrS[6]nPr** emerged in the 1D and 2D NMR spectra (ESI†). In fact, the ^1^H NMR signals of the macrocycle in the **72+@PrS[6]nPr** complex (Fig. 6b) are in agreement with an average *D*_6_ symmetry.^32^ The most plausible rationalization of this behavior is that the introduction of the guest into the macrocycle cavity forces the cuboid scaffold to open. This assumption was corroborated by DFT calculations (Fig. 6 top). The optimized structure (ESI†) of the complex **72+@PrS[6]nPr** shows an opening of the cuboid scaffold (open state in Fig. 6), with the guest **72+** that occupies the central cavity in which the average plane of the cyclohexane ring is tilted by 20° with respect to the mean plane of the host methylene bridges. An association constant value of 1.2 × 10^8^ M^−1^ (Table 2, 298 K, CD_2_Cl_2_, see the ESI†) was determined for the **72+@PrS[6]nPr** complex by a series of competition experiments (ESI†).^33,34^ The host–guest complex is stabilized by ion-dipole (N^+^⋯OR), cation⋯π, C–H⋯π, and van der Waals interactions.

**Table 2:** Binding constant values of prism[*n*]arene host–guest complexes determined by ^1^H NMR experiments in CD_2_Cl_2_ (600 MHz) (see the ESI for details). Errors <15% calculated as mean values of three measurements

	**52+**	**6+**	**72+**	**82+**	**92+**
**PrS[5]Me**	3.9 × 10^7^	90	1.8 × 10^7^	6.6 × 10^6^	5.8 × 10^3^
**PrS[5]Et**	1.4 × 10^8^	50	2.8 × 10^8^	2.9 × 10^6^	1.0 × 10^5^
**PrS[5]nPr**	1.7 × 10^8^	100	1.4 × 10^9^	6.3 × 10^7^	4.8 × 10^5^
**PrS[6]Me**	50	2700	—a	4200	3700
**PrS[6]Et**	790	2745	1.0 × 10^8^	420	—a
**PrS[6]nPr**	400	340	1.2 × 10^8^	370	—a

a An intricate mixture of complexes was present at the equilibrium due to the presence of macrocyclic hosts in different conformations.

Regarding the C–H⋯π interactions, Natural Bond Orbital (NBO)^35,36^ (ESI†) and Non-Covalent Interactions (NCI) calculations (ESI†) revealed that a significant contribution to the stabilization energy of the **72+@PrS[6]nPr** complex was provided by the four equatorial C–H groups of the guest and the aromatic naphthalene walls (26%, see RDG based NCI analysis in the ESI†), while the axial C–H groups give a negligible contribution (3.0%). Finally, the ^+^NCH_3_ group also establishes cationic C–H⋯π interactions with the aromatic walls of the host, as confirmed by RDG-based NCI analysis (ESI†).

**Fig. 7:**
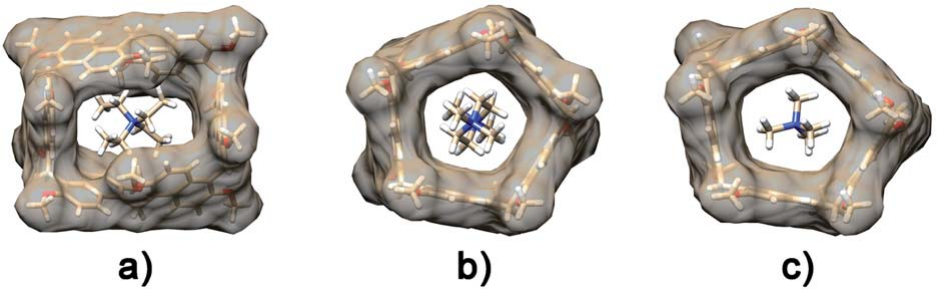
X-ray structural models of the host–guest complexes: (a) **6+@PrS[6]Me**, (b) **72+@PrS[5]Me**, and (c) **10+@PrS[5]Me**. The transparent van der Waals surface of the host is shown to illustrate the effective prismarene cavities. Counter ions, solvents, and disordered guest atoms are omitted for clarity.

An analogous conformational change from a closed to an open conformation was also revealed for the **PrS[6]Et** host after *endo*-cavity complexation with **72+**. Attempts to obtain crystals of **72+@PrS[6]nPr** or **72+@PrS[6]Et** complexes were unsuccessful. However diffracting crystals of **6+@PrS[6]Me**·BArF^19^ were obtained and the X-ray structure of this complex (Fig. 7a) provides interesting structural details regarding the open state of the prism[6]arene skeleton. The symmetric **PrS[6]Me** host lies on the crystallographic two-fold axis passing through the center of two opposite naphthalene rings of the host. The internal guest results in an opening of the cuboid arrangement towards the formation of a hexagonal prism. This is reflected by the supplementary canting angles of the B/B′′ and B′′′/B′ couples, which change from *ca.* 40°/140° of free **PrS[6]R** to *ca.* 60°/120° in the complex (Table S7†), closer to the ideal angle of 90° of a regular hexagonal prism.

**Fig. 8:**
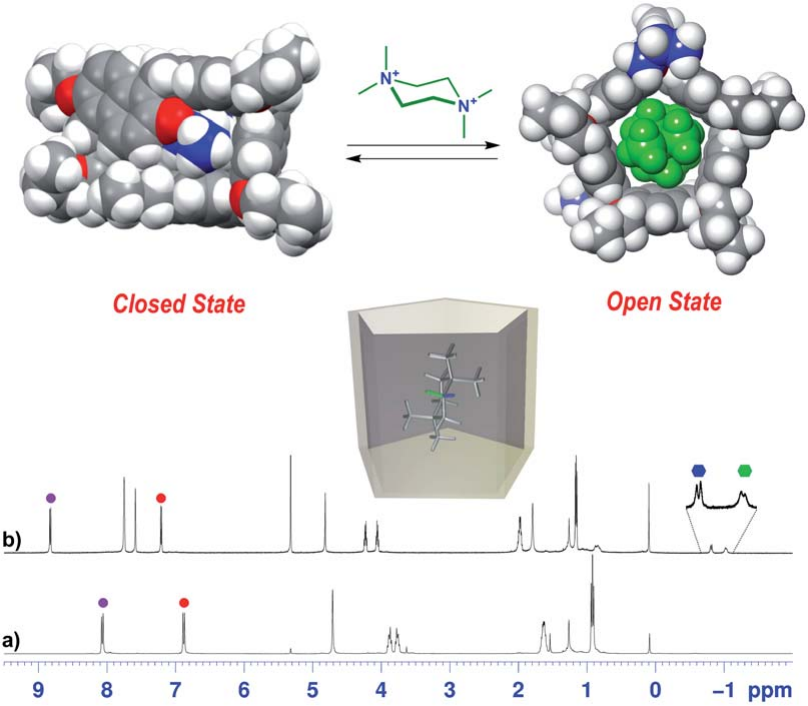
^1^H NMR spectra of (a) **PrS[5]nPr** in CD_2_Cl_2_ at 298 K (600 MHz) and (b) a 1 : 1 mixture of **PrS[5]nPr** and **72+**·(BArF)_2_ (3 mM). The circles indicate the signals of aromatic H atoms, while the hexagons indicate the diastereotopic methylene H atoms of **72+** shielded inside the macrocycle. The correspondence between the marked signals (red and purple circles for aromatic H atoms) indicates the conformational change from a closed state to an open state upon complexation with **72+**. The blue and green hexagons indicate the diastereotopic methylene H atoms of **72+** shielded inside the macrocycle. (Top) DFT optimized structure of **PrS[5]nPr** in the closed state and of its complex with **72+** (**72+@PrS[5]nPr**).

Furthermore, the interior dihedral angles between the naphthalene planes increase from an averaged value of 90° observed in free **PrS[6]R** molecules to an averaged value of 105°, closer to the ideal angle of 120° of a regular hexagonal prism. A close inspection of the solid state structure of the **6+@PrS[6]Me**·BArF complex reveals the important role of cationic C–H⋯π interactions in the host–guest complexation. Analogously, **PrS[5]nPr** undergoes a conformational change from a closed to an open state upon *endo*-cavity complexation with the cationic guest **72+**. When **PrS[5]nPr** was mixed with *N*,*N*,*N′*,*N′*-tetramethylpiperazonium salt **72+**·(BArF)_2_, in CD_2_Cl_2_ at room temperature, drastic changes were observed in the ^1^H NMR spectrum (Fig. 8). The presence of upfield shifted signals of **72+** methylene H atoms to negative values (Fig. 8b) is indicative of the inclusion of **72+** inside the cavity, shielded by the **PrS[5]nPr** walls. An AB system is observed at −0.82/−1.04 ppm (*J* = 11.5 Hz). Furthermore, the Δ*δ* value between the aromatic doublets experiences a variation from 1.18 (Δ*δ* = 8.07 − 6.89 = 1.18) for the free host (Fig. 8a) to 1.62 ppm (Δ*δ* = 8.82 − 7.20 = 1.62) (Fig. 8b) upon complexation. This result clearly indicates an opening of the folded structure of **PrS[5]nPr** (closed state in Fig. 8) that upon complexation with **72+** adopts the open state in Fig. 8 in which the **PrS[5]nPr** host shows a *D*_5_ symmetry.

In this conformation, the aromatic walls adopt canting angle values of about 90° and therefore define a regular pentagonal prism (Fig. 8).^37^ Thus, in the open state the aromatic H atoms move away from the cavity and experience a down-field shift (Fig. 8b) with respect to the closed state (Fig. 8a). The attempts to obtain crystals of **72+@PrS[5]nPr** or **72+@PrS[5]Et** complexes were also unsuccessful in this case. However diffracting crystals of **72+@PrS[5]Me**·BArF and **10+@PrS[5]Me**·BArF were obtained and the X-ray structures of these complexes (Fig. 7b and c) provide structural details regarding the open state of the prism[5]arene skeleton (see also the DFT optimized structure of the **72+@PrS[5]nPr** complex in Fig. 8 and the ESI†). The crystal structure of **72+@PrS[5]Me**·(BArF)_2_ (Fig. 7b) shows the guest located at the center of the prismatic host with the chair's plane perpendicular to the mean plane of methylene bridges of the macrocycle (85.8°). The pseudo-two-fold symmetry axis of **72+**, orthogonal to the chair's plane, forms an angle of about 50° with a crystallographic two-fold axis, thereby resulting in a two-position statistical disorder of the **72+** ion.

The X-ray structure of the **72+@PrS[5]Me** complex reveals a high steric complementarity between the **PrS[5]Me** cavity and the cationic **72+** guest that gives rise to relevant C–H⋯π interactions. The host resembles an almost regular pentagon prism with canting angles of 90.0° ± 3.2° and interior dihedral angles ranging from 104.6° to 110.5°, with an average angle of 107.9°, very close to the ideal 108° angle (Fig. 7b). Similarly, the crystal structure of **10+@PrS[5]Me**·BArF also evidences an opening of the prismatic host in both crystallographic independent host–guest complexes (see Tables S7 and S8†) (Fig. 7c). The macrocycle adopts an almost regular pentagon prism with canting angles of 90.0° ± 3.3° and interior dihedral angles ranging from 102.1° to 113.7°, with an average angle of 108°, equal to the ideal angle (Fig. 7c).

The tetramethylammonium guests are well-centered in the **PrS[5]Me** cavity to give the important cationic C–H⋯π interactions (Fig. 7c). By a series of NMR competition experiments, an association constant value of 1.4 × 10^9^ M^−1^ (298 K, CD_2_Cl_2_) was determined for the **72+@PrS[5]nPr** complex (Table 2). This was confirmed by fluorescence titration (*K*_ass_ = 1.7 × 10^9^ M^−1^, see the ESI†). Interestingly, NBO analysis (ESI†) conducted on **72+@PrS[5]nPr** indicates that both axial and equatorial C–H⋯π interactions play a significant role in the stabilization of the complex. In a similar way, **PrS[5]nPr** shows high affinity for dication *N*,*N′*-dihexyl-DABCO **52+** (ESI†). In fact, the ^1^H NMR spectrum of **PrS[5]nPr** shows significant changes upon addition of **52+** as a barfate salt (ESI†) and is indicative of the formation of pseudorotaxane **52+@PrS[5]nPr**. The association constant values for the formation of **52+@PrS[5]R** complexes (Table 2) were determined by competition experiments (ESI†). Values of 1.7 × 10^8^ M^−1^ and 1.4 × 10^8^ M^−1^ were obtained for **PrS[5]nPr** and **PrS[5]Et**, respectively.

Comparison among the affinities of the **PrS[5]R** hosts toward the *N*,*N′*-dialkyl-DABCO **52+** and **82+** cations in Table 2 indicates that the longer alkyl chains of axle **52+** play a crucial role in the stabilization of pseudorotaxanes **52+@PrS[5]R**.

## Conclusions

Prism[6]arenes bearing ethyl or propyl chains on their rims are obtained in high yields and in short reaction times, independent of the nature and size of the solvent. Differently, in the presence of small methoxy groups, the hexamer **PrS[6]Me** is not formed in any solvent (CyHex, toluene, or 1,2-DCE), and analogously the **PrS[6]Pentyl** derivative is formed in a very low yield. The reaction progress, monitored by HPLC, revealed that **PrS[6]R** (R = *n*Pr and Et) are the favored products, both kinetically and thermodynamically. Differently, as previously reported for methylated prismarenes,^19^ the pentamer **PrS[5]Me** is the kinetically favored product, while its confused isomer **c-PrS[5]Me** prevails under thermodynamic conditions. Based on these results, we have invoked a kinetically and thermodynamically self-templating effect by the Et and *n*Pr alkyl chains in the synthesis of prismarene hexamers. Insights into this effect were obtained by detailed X-ray and 1D and 2D NMR investigations supported by DFT calculations. These studies clearly showed that **PrS[6]Et** and **PrS[6]nPr** are folded into a square cuboid-shape with *D*_2_ symmetry. In this conformation, four inward oriented alkyl chains of the naphthalene moieties, located on the elongated faces of the prism, fill the cavity of the macrocycle, and establish C–H⋯π interactions with the aromatic walls and various inter-chain van der Waals interactions. 1D and 2D NMR studies show that the cuboid structure of the hexamers is also maintained in solutions of different solvents, such as 1,2-DCE, CH_2_Cl_2_, decaline, and chloro-cyclohexane. On these bases, we propose that the synthesis of the ethyl and propyl prism[6]arenes occurs under an intramolecular thermodynamic self-templating effect in which the formation of the prism[6]arene is not driven by an external templating agent (*e.g.*, solvent) but by intramolecular self-templating of the alkyl chains. In other words, the self-filling of the internal cavity of **PrS[6]Et** or **PrS[6]nPr** stabilizes their cuboid structure driving the equilibrium toward their formation.

The cuboid structure of the prism[6]arene shows an inaccessible closed cavity, and molecular recognition studies, both in solution and in the solid state, show that the introduction of guests into the macrocycle cavity forces the cuboid scaffold to open, through an induced-fit mechanism, which originates from the conformational flexibility of the prismarene macrocycle. An analogous conformational change from a closed to an open state occurs during the *endo*-cavity complexation process of the pentamer.

Thus, we here have described a rare example of a documented cyclization process kinetically and thermodynamically templated by intramolecular interactions. The in-depth investigation of the self-filling effects of the alkyl chains on the synthesis of prism[6]arenes, and on their conformational properties, could pave the way to the synthesis of macrocycles exploiting this novel thermodynamically controlled intramolecularly self-templated cyclization process, different from those currently known, based on the external effect of solvents or guests.

## Data availability

CCDC 2025594 (for **PrS[5]Et**); 2025589 (for **PrS[5]nPr**); 2025583 (for **PrS[6]Et**); 2025585 (for **PrS[6]nPr**); 2050614 (for **7·PrS[5]Me·(BArF)2**); 2050617 (for **3·PrS[5]Me·BArF**); 2050618 (for **6·PrS[6]Me·BArF**) contain the supplementary crystallographic data for this paper.

## Author contributions

P. D. S.: DFT calculations; R. D. R., L. D. M., C. C. and C. P.: synthesis; S. G. and N. H.: X-ray studies; C. T., N. C. I. and N. B. O. calculations; A. C.: TS DFT calculations; C. G. and S. G.: conceptualization, data analysis and manuscript writing; P. N.: supervision.

## Conflicts of interest

There are no conflicts to declare.

## Supplementary Material

SC-012-D1SC02199K-s001

SC-012-D1SC02199K-s002
