# Supplementary material for: An intramolecularly self-templated synthesis of macrocycles: self-filling effects on the formation of prismarenes
Source: Chem Sci. 2021 Jun 3;12(29):9952–61. doi: 10.1039/d1sc02199k (PMC8317625; doi:10.1039/d1sc02199k)
Supplement: SC-012-D1SC02199K-s001 [file SC-012-D1SC02199K-s001.pdf]

Electronic Supplementary Material (ESI) for Chemical Science.
This journal is © The Royal Society of Chemistry 2021

Supplementary Information

An Intramolecularly Self-Templated Synthesis of Macrocycles: The Self-Filling Effects on the Formation of Prismarenes

Paolo Della Sala,[a],‡ Rocco Del Regno,[a],‡ Luca Di Marino,[a] Carmela Calabrese,[a] Carmine Palo,[a] Carmen Talotta,[a] Silvano Geremia,*,[b] Neal Hickey,[b] Amedeo Capobianco,[a] Placido Neri[a] and Carmine Gaeta*,[a]

Table of Contents

	**Pages**
General Experimental Details	**S2**
General procedure for the synthesis of Prism[n]arenes PrS[n]^{R}	**S2-S5**
Copies of 1D and 2D NMR and HR Mass Spectra of PrS[n]^{R}	**S6-S34**
Copies of NMR titration experiments with reaction solvents	**S35-S41**
Copies of 1D and 2D of PrS[n]^{R} Complexes	**S42-S51**
^{1}H NMR determination of K_{ass} values.	**S52-S75**
Fluorescence Titration	**S76**
DFT Calculations	**S77-S94**
DFT - optimized structures of Complexes	**S95-S138**
NBO and NCI analysis	**S139-S142**
Determination of crystallographic structures of prismarenes: **PrS[5]^{Et}**, **PrS[5]^{nPr}**, **PrS[6]^{Et}** and **PrS[6]^{nPr}** and prismarenes host-guest complexes: **7@PrS[5]^{Me}**·(BArF)$_2$, **10@PrS[5]^{Me}**·BArF and **6@PrS[6]^{Me}**·BArF	**S143-156**
Potential void volumes of prismatic macrocycles.	**S157**
Comparison between DFT and Crystallographic Structures of **PrS[5]^{nPr}** and **PrS[6]^{nPr}**	**S158**
HPLC analysis of the cyclocondensation kinetics	**S159**

General Experimental Details

HR MALDI mass spectra were recorded on a Bruker Solarix FT-ICR mass spectrometer equipped with a 7T magnet. All samples were recorded in MALDI (4 laser shots were used for each scan) and they were prepared by mixing 10 µL of analyte in dichloromethane (1 mg/mL) with 10 µL of solution of 2,5-dihydroxybenzoic acid (10 mg/mL in Metanol). The mass spectra were calibrated externally, and a linear calibration was applied. 1,2-dichloroethane was dried by heating under reflux over calcium hydride. All chemicals reagents grade was used without further purification and were used as purchased by Merck, TCI and Fluorochem. Reaction temperatures were measured externally. Reactions were monitored by Merck TLC silica gel plates (0.25 mm) and visualized by UV light 254 nm, or by spraying with H_2SO_4-$Ce(SO_4)_2$. NMR spectra were recorded on a Bruker Avance-600 [600 (^1H) and 150 MHz (^{13}C)], Avance-400 [400 (^1H) and 100 MHz (^{13}C)], Avance-300 MHz [300 (^1H) and 75 MHz (^{13}C)] or Avance-250 MHz [250 (^1H) and 62.5 MHz (^{13}C)] spectrometers. Chemical shifts are reported relative to the residual solvent peak[1]. Standard pulse programs, provided by the manufacturer, were used for 2D COSY-45, 2D HSQC and 2D NOESY experiments.

General procedure for the synthesis of Prism[n]arenes PrS[n]^R.

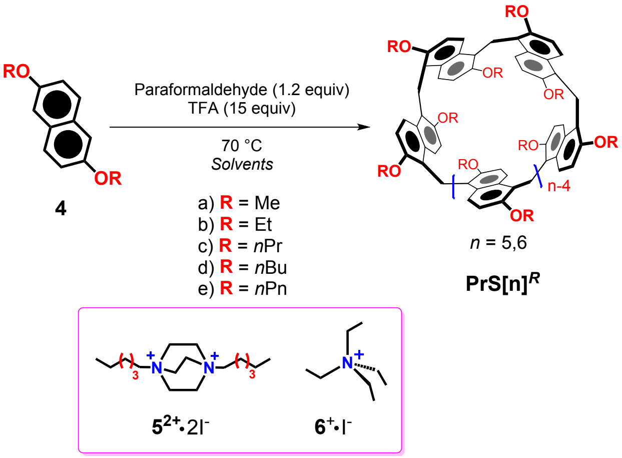

A solution of 2,6-dialkoxynaphthalene **4b-e**, paraformaldehyde (1.2 equiv) in appropriate solvent (Table S1, 5.0 mM) was heated at 70 °C, then trifluoroacetic acid (15 equiv) was added and the solution was stirred at 70 °C for the time indicated in Table S1. Then, an aqueous saturated solution of $NaHCO_3$ (100 mL) was added in the reaction mixture and the organic layer was extracted. Finally, the organic phase was dried on Na_2SO_4 and concentrated to give a light brown solid.

- Starting by 2,6-diethoxynaphthalene **4b** (250 mg, 1.2 mmol): The crude was purified through chromatographic column on silica gel (only dichloromethane) to give the macrocycle **PrS[6]**^{Et} as white solid (see Table S1).

[1] G. R. Fulmer, A. J. M. Miller, N. H. Sherden, H. E. Gottlieb, A. Nudelman, B.M. Stoltz, J.E. Bercaw, K.I. Goldberg, *Organometallics* 2010, **29**, 2176–2179.

- Starting by 2,6-dipropoxynaphthalene **4c** (250 mg, 1.0 mmol): The crude was purified through chromatographic column on silica gel (*n*-hexane/dichloromethane = 1/1) to give the macrocycle **PrS[6]**^{nPr} as white solid (see Table S1).
- Starting by 2,6-dibutoxynaphthalene **4d** (300 mg, 1.1 mmol): The crude was purified through chromatographic column on silica gel (*n*-hexane/dichloromethane = 4/6) to give the macrocycle **PrS[6]**^{nBu} as white solid (see Table S1).
- Starting by 2,6-dipentoxynaphthalene **4e** (300 mg, 1.0 mmol): The crude was purified through chromatographic column on silica gel (*n*-hexane/dichloromethane = 6/4) to give the macrocycle **PrS[6]**^{nPn} as white solid (see Table S1).

Table S1

Solvent	Monomer	Time	PrS[6]^{R} (%)
Cl-CyHex	**4b**	40 min	71
Cl-CyHex	**4c**	2 h	64
Toluene	**4b**	3 h	74
Toluene	**4c**	8 h	57
Cyclohexane	**4b**	30 min	76
Cyclohexane	**4c**	40 min	65
Decaline	**4b**	30 min	75
Decaline	**4c**	45 min	70
1,2-DCE	**4b**	45 min	75
1,2-DCE	**4c**	90 min	65
1,2-DCE	**4d**	40 min	30
1,2-DCE	**4e**	45 min	8

Quaternary ammonium-templated synthesis.

Templated synthesis in presence of $6^{+}\cdot I^{-}$ salt:

In a solution of 2,6-dialkoxynaphthalene **4b-c** in 1,2-dichloroethane (5 mM) was added paraformaldehyde (1.2 equiv) and the templating agent **$6^{+}\cdot I^{-}$** (1.0 equiv). The mixture was heated at 70° C and then trifluoroacetic acid (15 equiv) was added. The solution was stirred for 22 h at 70 °C and subsequently the solvent was evaporated under reduced pressure. The residue was dissolved in CH_2Cl_2 (30 mL) and the mixture was washed with an aqueous saturated solution of $NaHCO_3$ (30 mL). Finally, the organic layer was washed with a 10 % aqueous solution of sodium thiosulfate (100 mL), and the organic phase was dried on Na_2SO_4 and concentrated to give a light brown solid.

- Starting by 2,6-diethoxynaphthalene **4b** (250 mg, 1.2 mmol): The crude was purified through chromatographic column on silica gel (only dichloromethane) to give the macrocycle **PrS[6]**^{Et} as white solid (162 mg, 60 %).

- Starting by 2,6-dipropoxynaphthalene **4c** (250 mg, 1.0 mmol): The crude was purified through chromatographic column on silica gel (*n*-hexane/dichloromethane = 1/1) to give the macrocycle **PrS[6]**^{nPr} as white solid (150 mg, 55 %).

Templated synthesis in presence of $5^{2+}\cdot 2I^{-}$ salt:

In a solution of 2,6-dialkoxynaphthalene **4b-c** in 1,2-dichloroethane (5 mM) was added paraformaldehyde (1.2 equiv) and templating agent $\mathbf{5^{2+}\cdot I^{-}}$ (1.0 equiv). The mixture was heated at 70 °C and then trifluoroacetic acid (15 equiv) was added. The solution was stirred at 70 °C for 22 h. After, 250 mL of an aqueous saturated solution of $NaHCO_3$ was added and the organic layer was washed with a 10 % aqueous solution of sodium thiosulfate (200 mL). Finally, the organic layer was dried on Na_2SO_4 and evaporated in vacuum to give a light brown solid.

- Starting by 2,6-diethoxynaphthalene **4b** (1.00 g, 4.6 mmol): The crude product was purified through chromatographic column on silica gel (*n*-hexane/toluene/dichloromethane = 1/2/7) to give the macrocycles **PrS[5]**^{Et} (110 mg, 10 %) and **PrS[6]**^{Et} (370 mg, 35 %) as white solids.
- Starting by 2,6-dipropoxynaphthalene **4c** (1.00 g, 4.1 mmol): The crude product was purified through chromatographic column on silica gel (*n*-hexane/toluene/dichloromethane = 2.5/2.5/5) to give the macrocycles **PrS[5]**^{nPr} (270 mg, 25 %) and **PrS[6]**^{nPr} (260 mg, 25 %) as white solids.

Derivative PrS[5]^{Et}:

M.p.:.> 372 °C dec. ^{1}H NMR (CD_2Cl_2, 400 MHz, 298 K): δ 7.85 (*d*, 10H, Ar-*H*, *J* = 9.2 Hz), 6.81 (*d*, 10H, Ar-*H*, *J* = 9.2 Hz), 4.70 (*s*, 10H, ArC*H*$_2$Ar), 3.86 (*m*, 10H, OC*H*$_2$), 3.66 (*m*, 10H, OC*H*$_2$), 0.94 (*m*, 30H, C*H*$_3$). ^{13}C NMR (CD_2Cl_2, 100 MHz, 298 K): δ 152.0, 129.8, 125.1, 124.1, 114.5, 65.5, 22.2, 15.2. HR MS (MALDI) m/z [M]$^+$ calcd for $C_{75}H_{80}O_{10}$: 1140.5751; found: 1140.5796.

Derivative PrS[5]^{nPr}:

M. p. : > 333 °C dec. ^{1}H NMR (CD_2Cl_2, 400 MHz, 298 K): δ 8.06 (*d*, 10H, Ar-*H*, *J* = 9.6 Hz), 6.88 (*d*, 10H, Ar-*H*, *J* = 9.6 Hz), 4.71 (*s*, 10H, ArC*H*$_2$Ar), 3.88 (*m*, 10H, OC*H*$_2$), 3.76 (*m*, 10H, OC*H*$_2$), 1.63 (*m*, 20H, OCH$_2$C*H*$_2$), 0.92 (*t*, 30H, C*H*$_3$, *J* = 8.0 Hz).^{13}C NMR (CD_2Cl_2, 100 MHz, 298 K): δ 152.0, 129.9, 125.2, 123.7, 114.4, 71.5, 23.4, 21.7, 11.0. HR MS (MALDI) *m*/*z* [M]$^+$ calcd for $C_{85}H_{100}O_{10}$: 1280.7316; found: 1280.7388.

Derivative PrS[6]^{Et}:

M.p.:. > 340 °C dec. ^{1}H NMR (CD_2Cl_2, 600 MHz, 243 K, Figure S23): δ 8.19 (*d*, 4H, Ar-*H*, *J* = 9.6 Hz), 7.73 (*d*, 4H, Ar-*H*, *J* = 9.6 Hz), 7.40 (*d*, 4H, Ar-*H*, *J* = 9.6 Hz), 7.37 (*d*, 4H, Ar-*H*, *J* = 9.6 Hz), 6.91 (*d*, 4H, Ar-*H*, *J* = 9.6 Hz), 6.27 (*d*, 4H, Ar-*H*, *J* = 9.6 Hz), 4.82 (*s*, 8H,

ArCH_2Ar), 4.47 (*s*, 4H, ArCH_2Ar), 4.39 (*m*, 4H, OCH_2), 4.33 (*m*, 4H, OCH_2), 4.20 (*m*, 4H, OCH_2), 4.06 (*m*, 4H, OCH_2), 3.04 (*m*, 4H, OCH_2), 1.54 (*overlapped*, 16H, OCH_2 and CH_3), 1.42 (*t*, 12H, CH_3, *J* = 6.6 Hz), -0.94 (*t*, 12H, CH_3, *J* = 6.6 Hz). ^{13}C NMR (TCDE, 150 MHz, 298 K): δ 152.0, 151.6, 150.9, 129.6, 126.4, 124.9, 123.9, 123.5, 116.2, 114.8, 114.0, 66.0, 65.4, 65.1, 23.5, 21.2, 15.5, 13.5. HR MS (MALDI) m/z [M]$^+$ calcd for $C_{90}H_{96}O_{12}$: 1368.6902; found: 1368.6941.

Derivative PrS[6]^{nPr}:

M.p.:. > 306°C dec. ^{1}H NMR (CD_2Cl_2, 600 MHz, 243 K, Figure S26): δ 8.28 (*d*, 4H, Ar-*H, J* = 9.6 Hz), 7.91 (*d*, 4H, Ar-*H, J* = 9.6 Hz), 7.52 (*d*, 4H, Ar-*H, J* = 9.6 Hz), 7.35 (*d*, 4H, Ar-*H, J* = 9.6 Hz), 7.01 (*d*, 4H, Ar-*H, J* = 9.6 Hz), 6.35 (*d*, 4H, Ar-*H, J* = 9.6 Hz), 4.82 (*s*, 8H, ArCH_2Ar), 4.42 (*s*, 4H, ArCH_2Ar), 4.25 (*overlapped*, 8H, OCH_2), 4.17 (*m*, 4H, OCH_2), 3.95 (*m*, 4H, OCH_2), 3.00 (*m*, 4H, OCH_2), 2.29 (*m*, 4H, OCH_2), 1.95 (*m*, 8H, OCH$_2$CH_2), 1.85 (*m*, 8H, OCH$_2$CH_2), 1.14 (*t*, 12H, CH_3, *J* = 7.2 Hz), 1.05 (*m*, 12H, CH_3, *J* = 7.2 Hz), -0.08 (*m*, 4H, OCH$_2$CH_2), -0.24 (*m*, 4H, OCH$_2$CH_2), -0.71 (*m*, 12H, CH_3, *J* = 7.2 Hz). ^{13}C NMR (CD_2Cl_2, 150 MHz, 298 K): δ 153.3, 151.7, 130.2, 125.1, 124.9, 124.3, 115.8, 114.8, 114.3, 71.9, 71.7, 71.4, 23.7, 22.1, 21.6, 11.2, 10.4. HR MS (MALDI) m/z [M]$^+$ calcd for $C_{102}H_{120}O_{12}$: 1536.8780; found: 1536.8790.

Derivative PrS[6]^{nBu}:

M.p.:. > 310°C dec. ^{1}H NMR (TCDE, 400 MHz, 298 K): δ 8.13-7.81 (*overlapped*, 8H, Ar-*H*), 7.51 (*br*, 4H, Ar-*H*), 7.18 (*br*, 4H, Ar-*H*), 6.84 (*br*, 4H, Ar-*H*), 6.21 (*br*, 4H, Ar-*H*), 4.67 (*overlapped*, 8H, ArCH_2Ar), 4.27-3.83 (*overlapped*, 20H, ArCH_2Ar and OCH_2), 2.77 (*br*, 4H, OCH_2), 2.39 (*br*, 4H, OCH_2),1.70-1.30 (*overlapped*, 40H, OCH$_2$CH_2CH$_2$ and OCH$_2$CH$_2$CH_2), 0.80-0.71 (*overlapped*, 24H, CH_3), -0.26 (*br*, 8H, OCH$_2$CH$_2$CH_2), -0.73 (*br*, 12H, CH_3). ^{1}H NMR (TCDE, 400 MHz, 393 K): δ 7.83 (d, 12H, Ar-H, *J* = 12.0 Hz), 6.75 (d, 12H, Ar-H, *J* = 12.0 Hz), 4.63 (*s*, 12H, ArCH_2Ar), 3.58 (*br*, 24H, OCH_2), 1.10-1.20 (*overlapped*, 48H, OCH$_2$CH_2CH_2CH$_3$), 0.60 (br *t*, 36H, CH_3,). ^{13}C NMR (TCDE, 100 MHz, 298 K): δ 152.1, 151.6, 151.2, 129.6, 124.7, 124.4, 124.1, 115.0, 114.5, 114.0, 69.0, 31.9, 19.4, 18.4, 18.0, 13.9, 12.7. HR MS (MALDI) m/z [M]$^+$ calcd for $C_{114}H_{144}O_{12}$: 1705.0658; found: 1705.0698.

Derivative PrS[6]^{nPent}:

M.p.:. > 300°C dec. ^{1}H NMR (TCDE, 300 MHz, 393 K, Figure S17): δ 7.79 (*d*, 12H, Ar-*H, J* = 9.0 Hz), 6.72 (*d*, 12H, Ar-*H, J* = 9.0 Hz), 4.62 (*s*, 12H, ArCH_2Ar), 3.50 (*m*, 24H, OCH_2), 1.20 (*overlapped*, 72H, OCH$_2$CH_2CH_2CH_2), 0.72 (*m*, 36H, CH_3, *J* = 6.0 Hz). ^{13}C NMR (TCDE, 62.5 MHz, 298 K): δ 151.6, 151.0, 129.6, 124.5, 124.2, 114.3, 69.2, 29.6, 29.1, 28.2, 22.3, 13.9. HR MS (MALDI) m/z [M]$^+$ calcd for $C_{126}H_{168}O_{12}$: 1873.2536; found: 1873.2572.

Copies of 1D and 2D NMR and HR Mass Spectra of PrS[n]^{R}

Copies of 1D and 2D NMR and HR mass spectrum of PrS[5]^{Et}

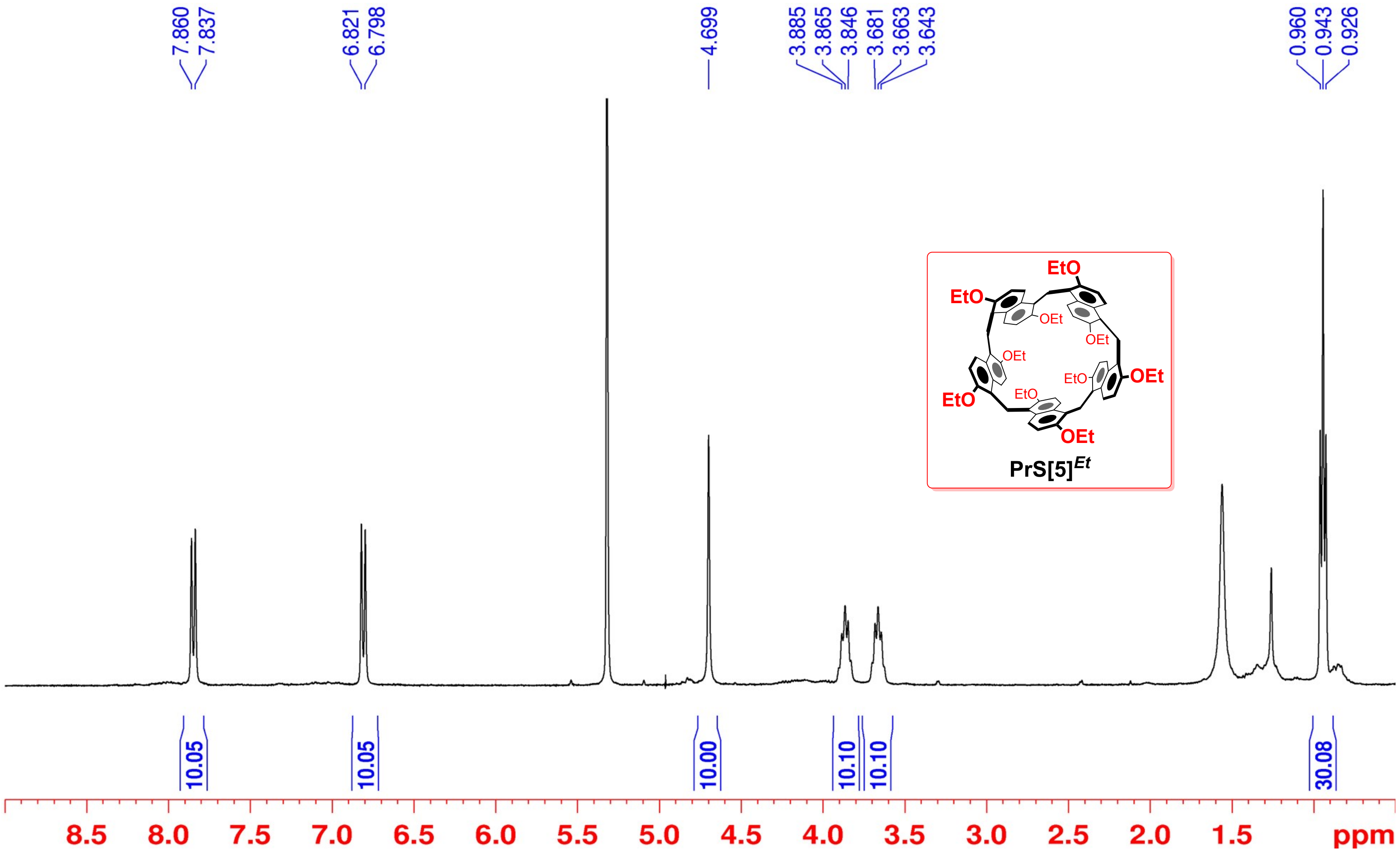

Figure S1: ^{1}H NMR spectrum of **PrS[5]^{Et}** (CD_2Cl_2, 400 MHz, 298 K).

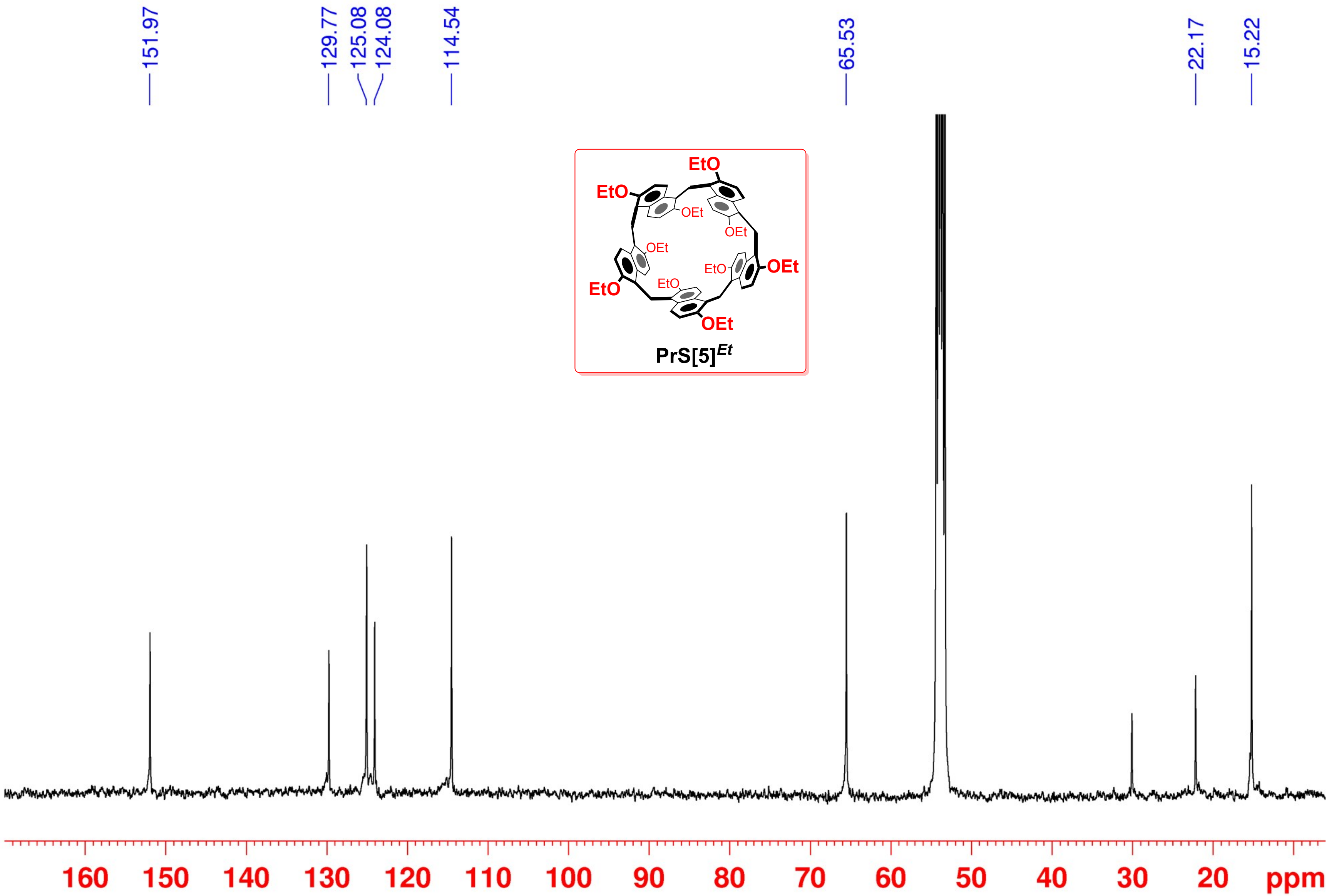

Figure S2: ^{13}C NMR spectrum of **PrS[5]*Et*** (CD_2Cl_2, 100 MHz, 298 K).

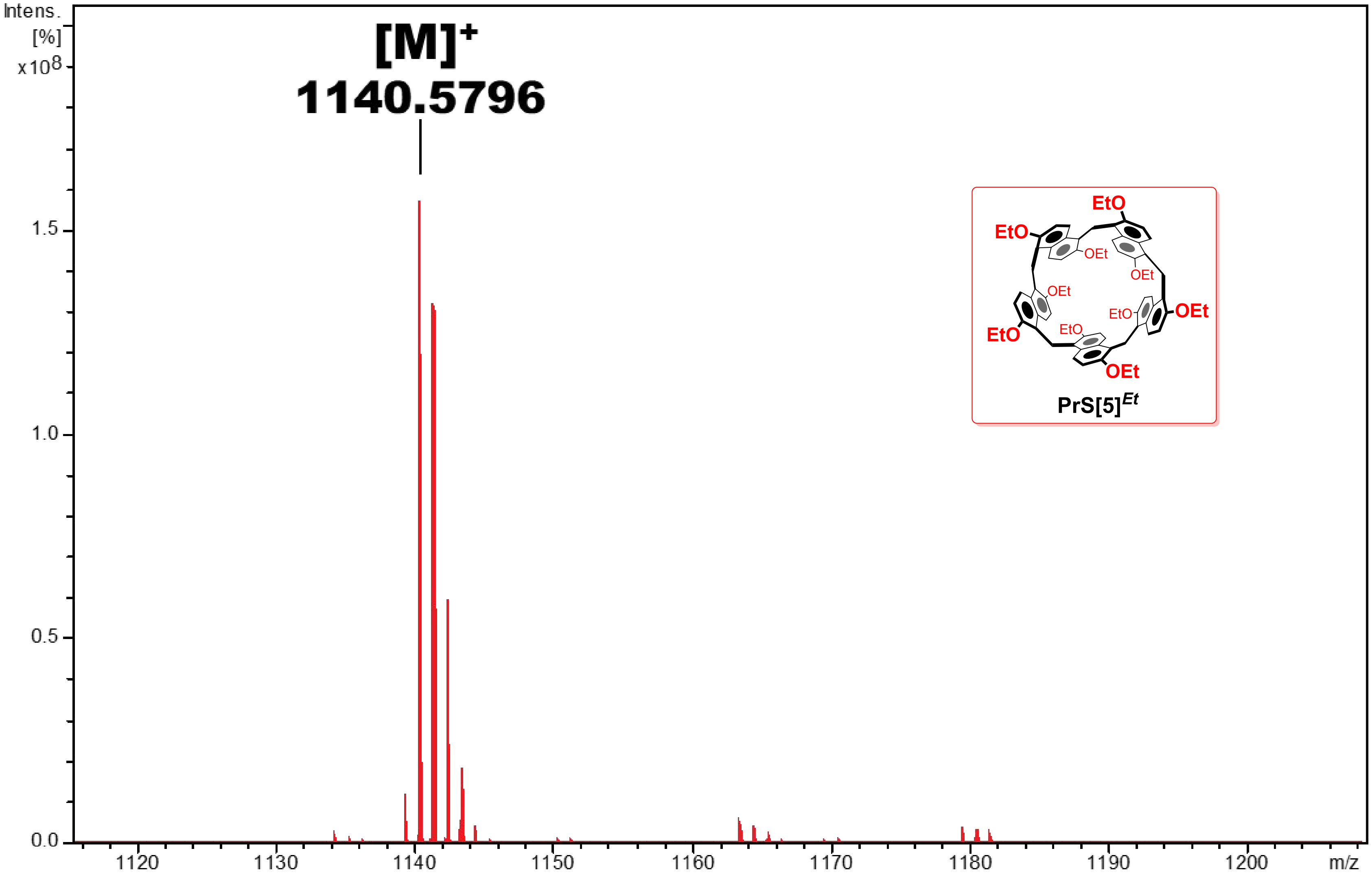

Figure S3: Significant portion of the HR MALDI FT-ICR mass spectrum of **PrS[5]**^{Et} $[M]^+$.

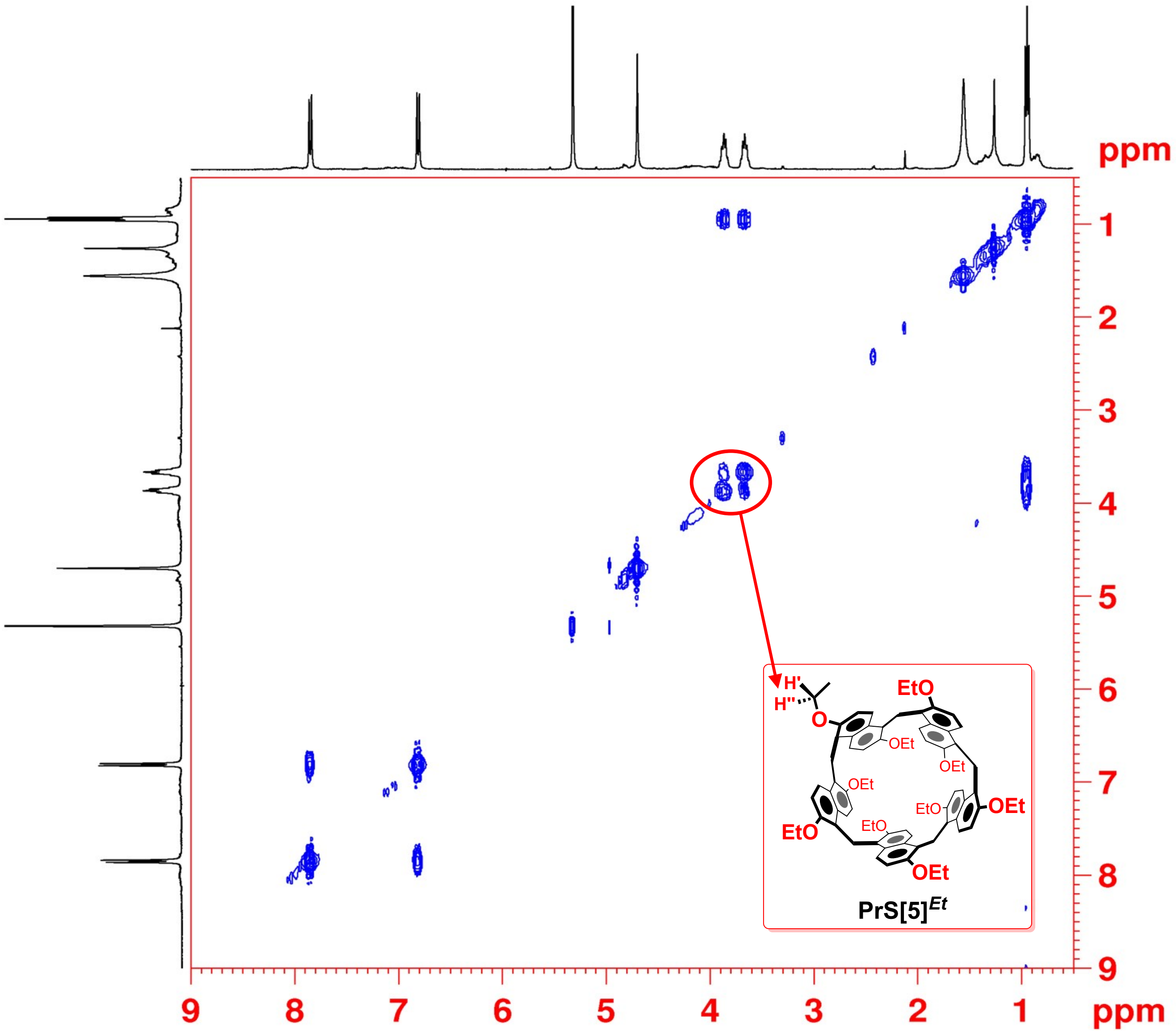

Figure S4: 2D-DQF COSY spectrum of **PrS[5]**^{Et} (CD_2Cl_2, 400 MHz, 298 K).

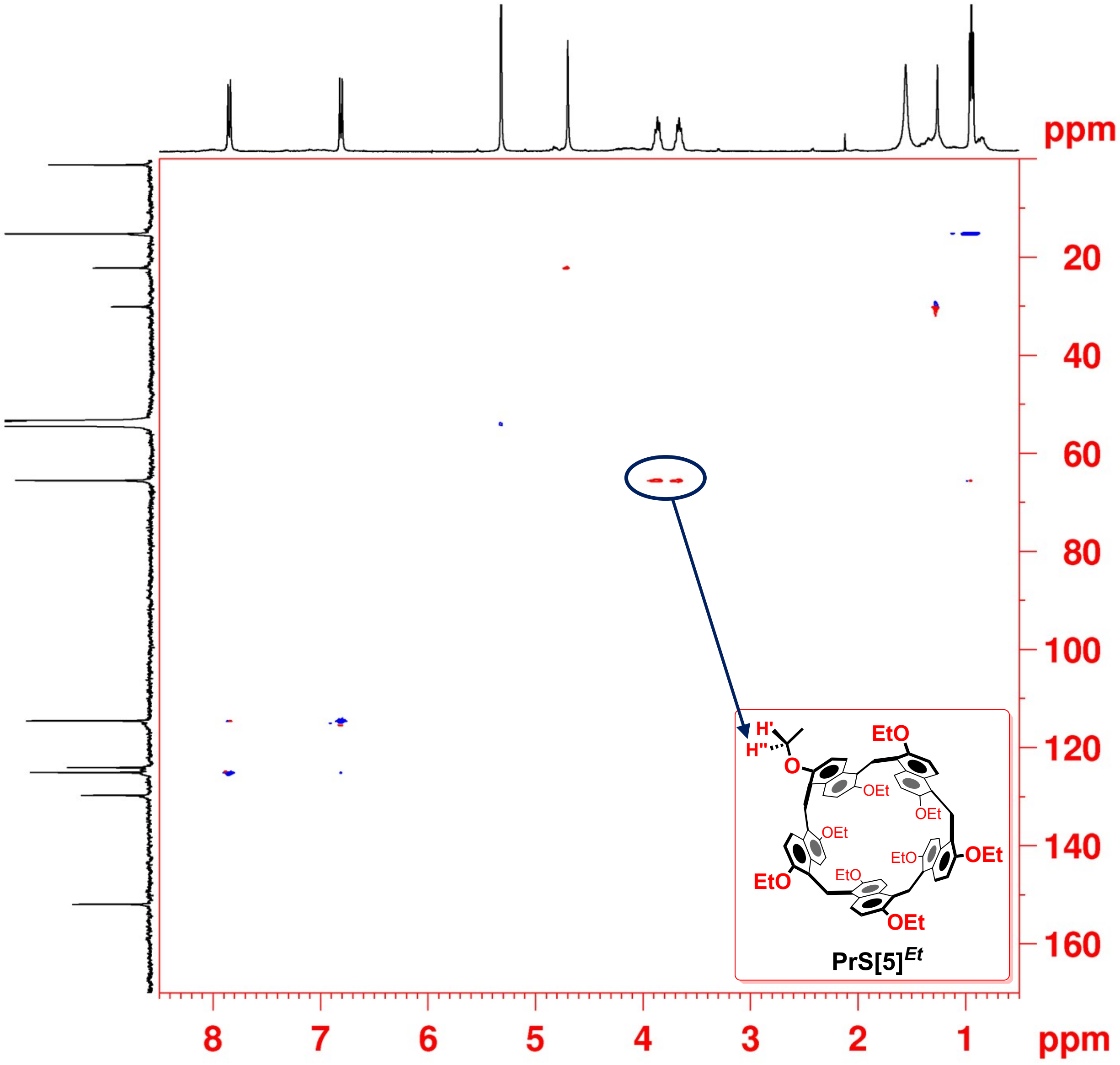

Figure S5: 2D-HSQC spectrum of **PrS[5]**^{Et} (CD_2Cl_2, 400 MHz, 298 K).

Copies of 1D and 2D NMR and HR mass spectrum of PrS[5]^{nPr}

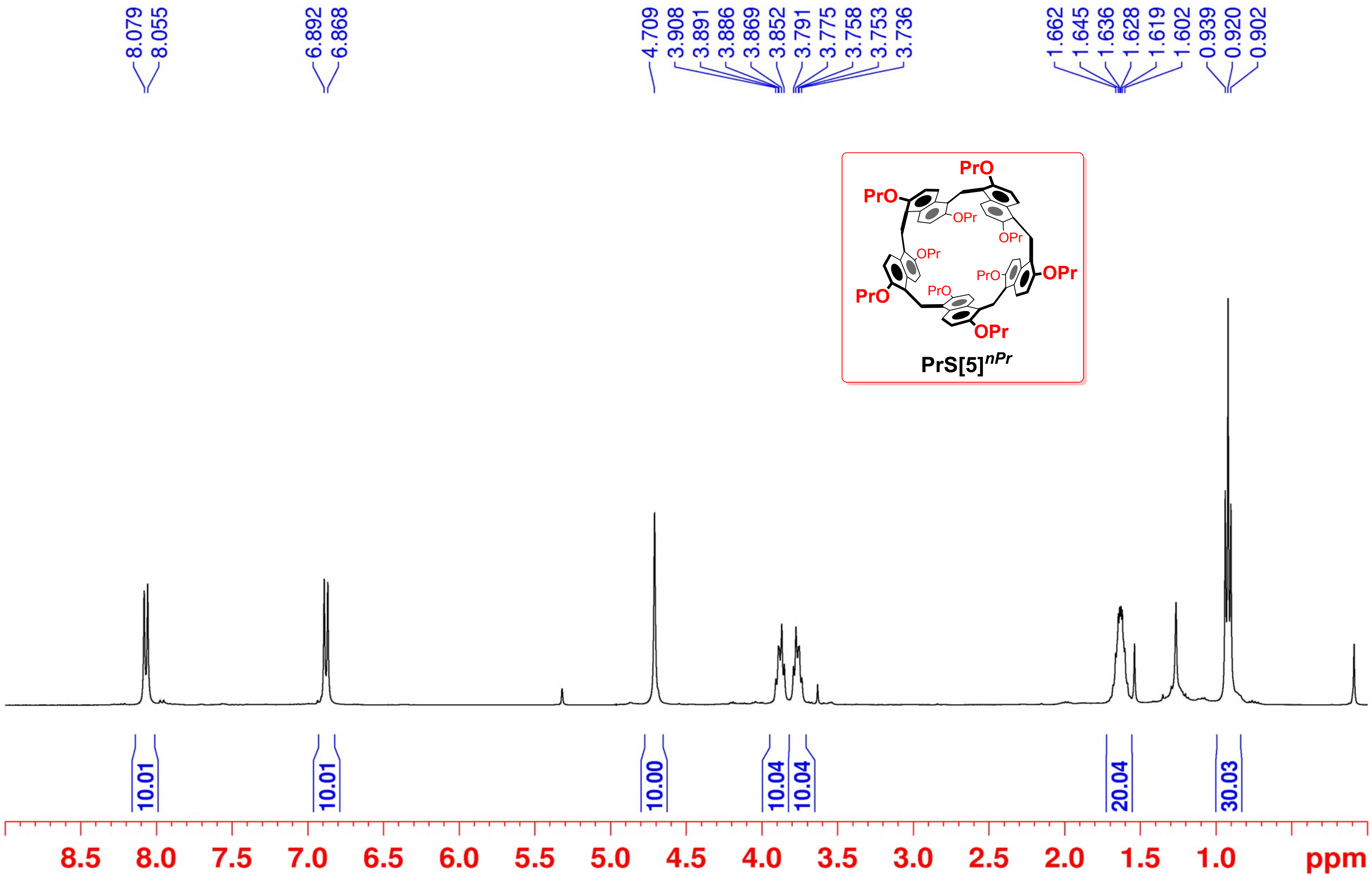

Figure S6: ^{1}H NMR spectrum of **PrS[5]^{nPr}** (CD_2Cl_2, 400 MHz, 298 K).

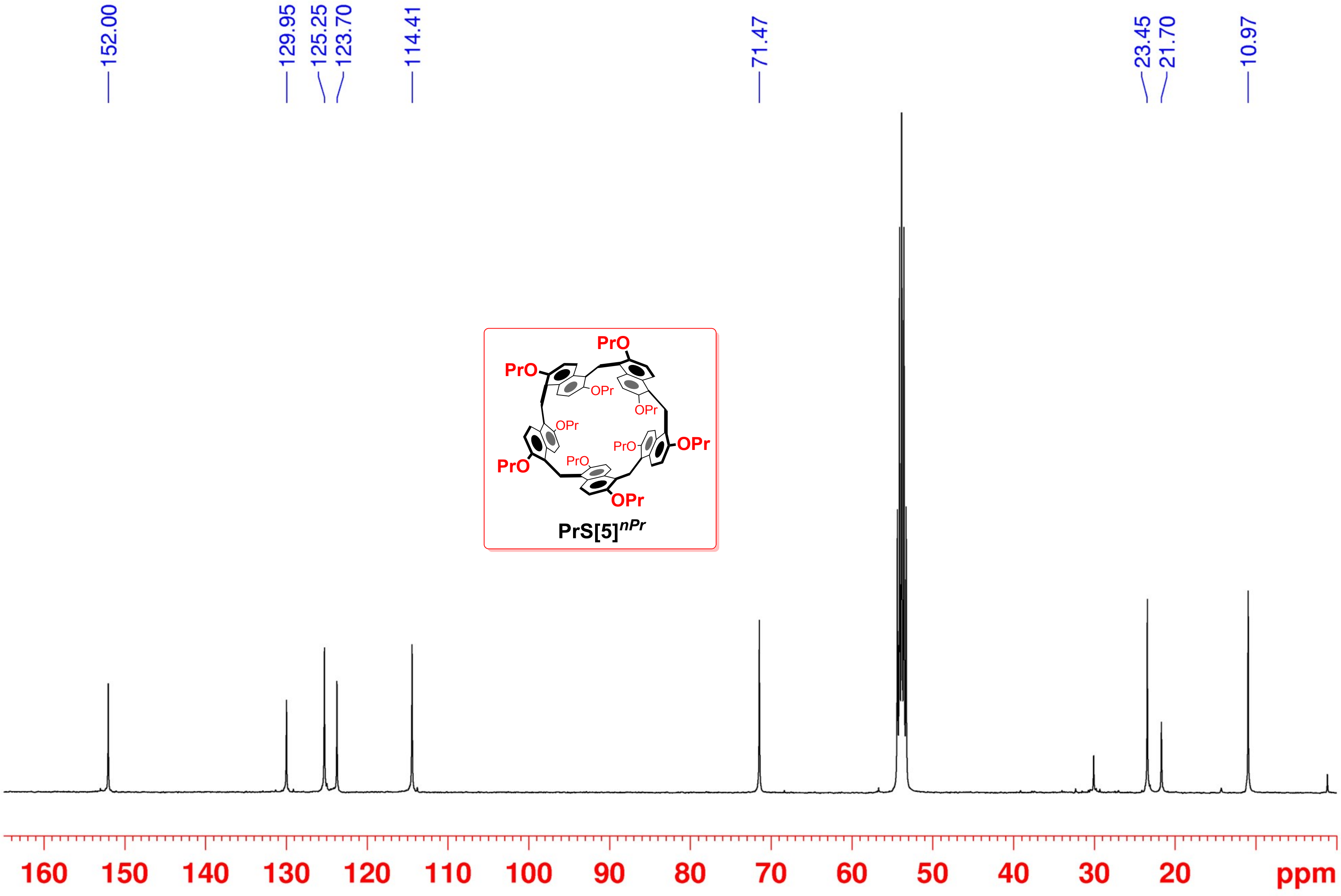

Figure S7: ^{13}C NMR spectra of **PrS[5]^{nPr}** (CD_2Cl_2, 100 MHz, 298 K).

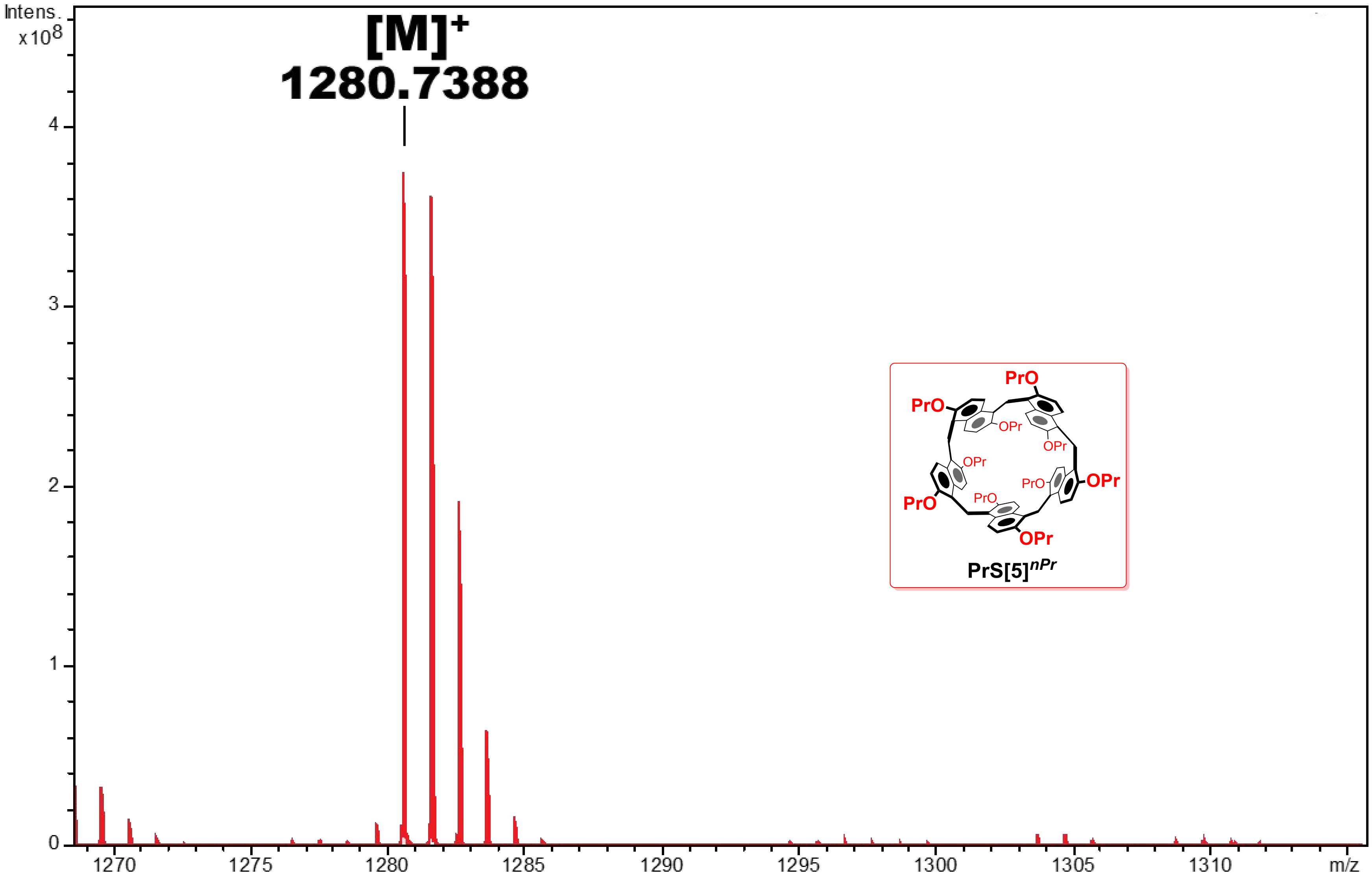

Figure S8: Significant portion of the HR MALDI FT-ICR mass spectrum of **PrS[5]**^{nPr} [M]$^+$.

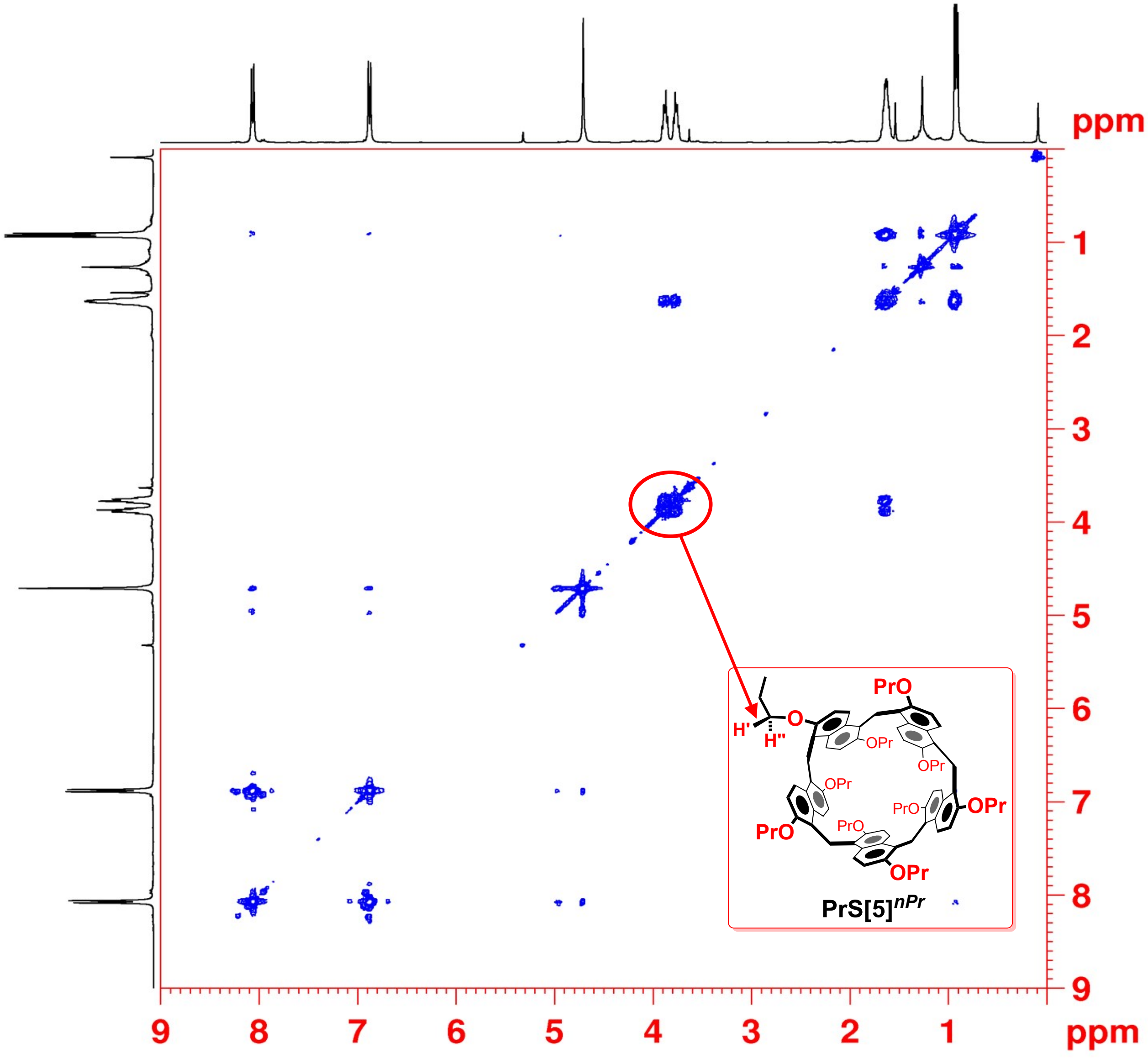

Figure S9: 2D-DQF COSY spectrum of **PrS[5]^{nPr}** (CD_2Cl_2, 400 MHz, 298 K).

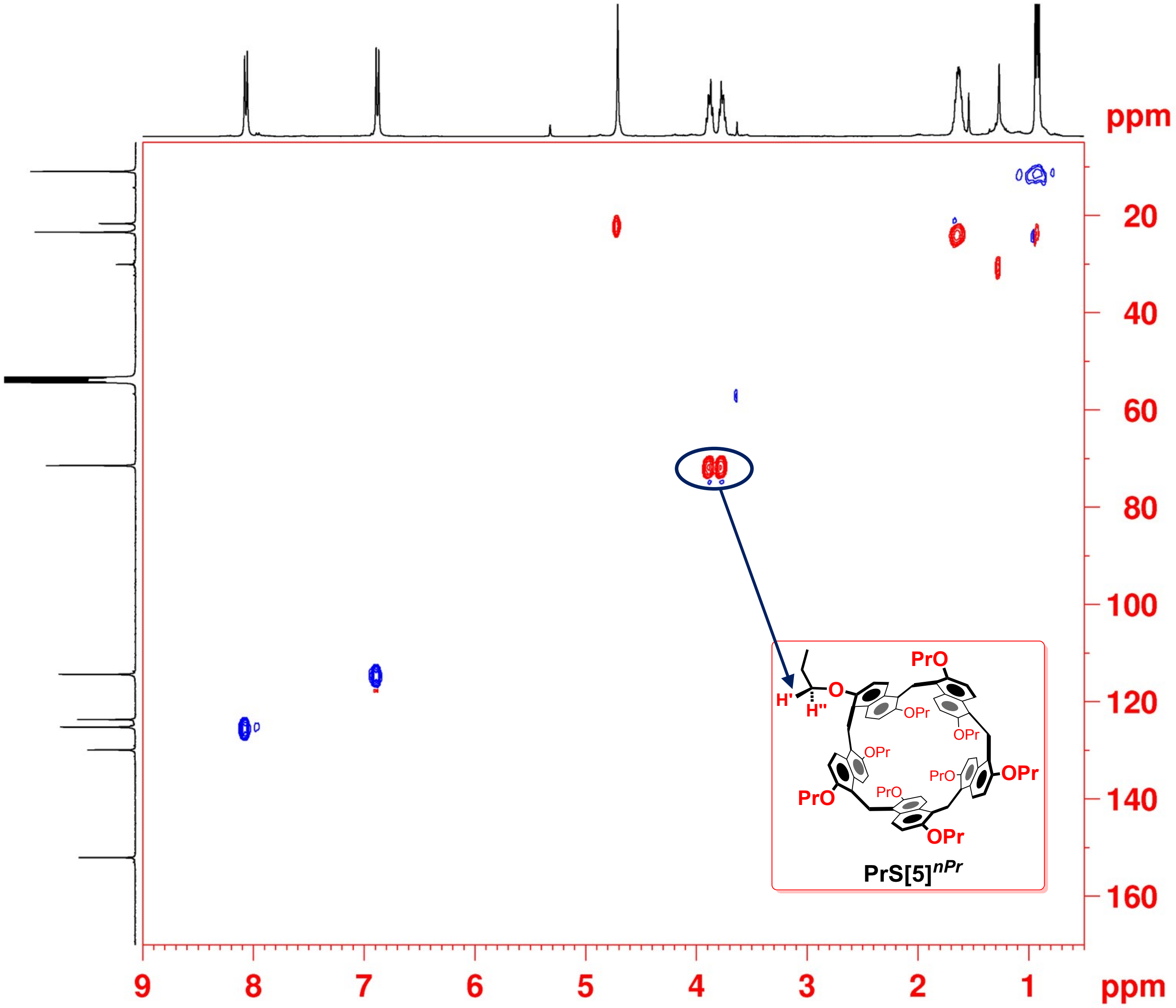

Figure S10: 2D-HSQC spectrum of **PrS[5]**^{nPr} (CD_2Cl_2, 400 MHz, 298 K).

Copies of NMR and HR mass spectrum of PrS[6]^{Et}

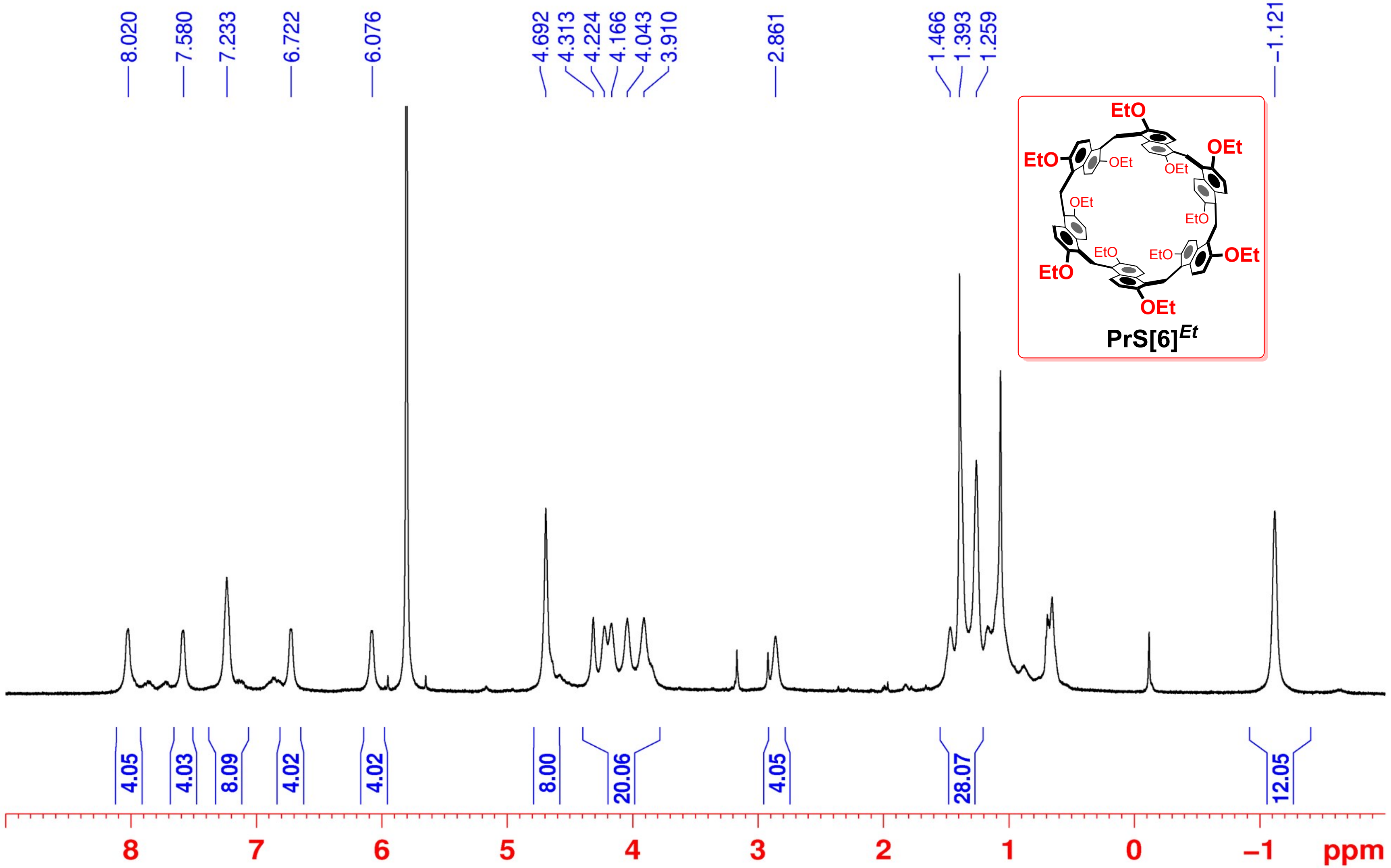

Figure S11: ^{1}H NMR spectrum of **PrS[6]^{Et}** (TCDE, 600 MHz, 298 K).

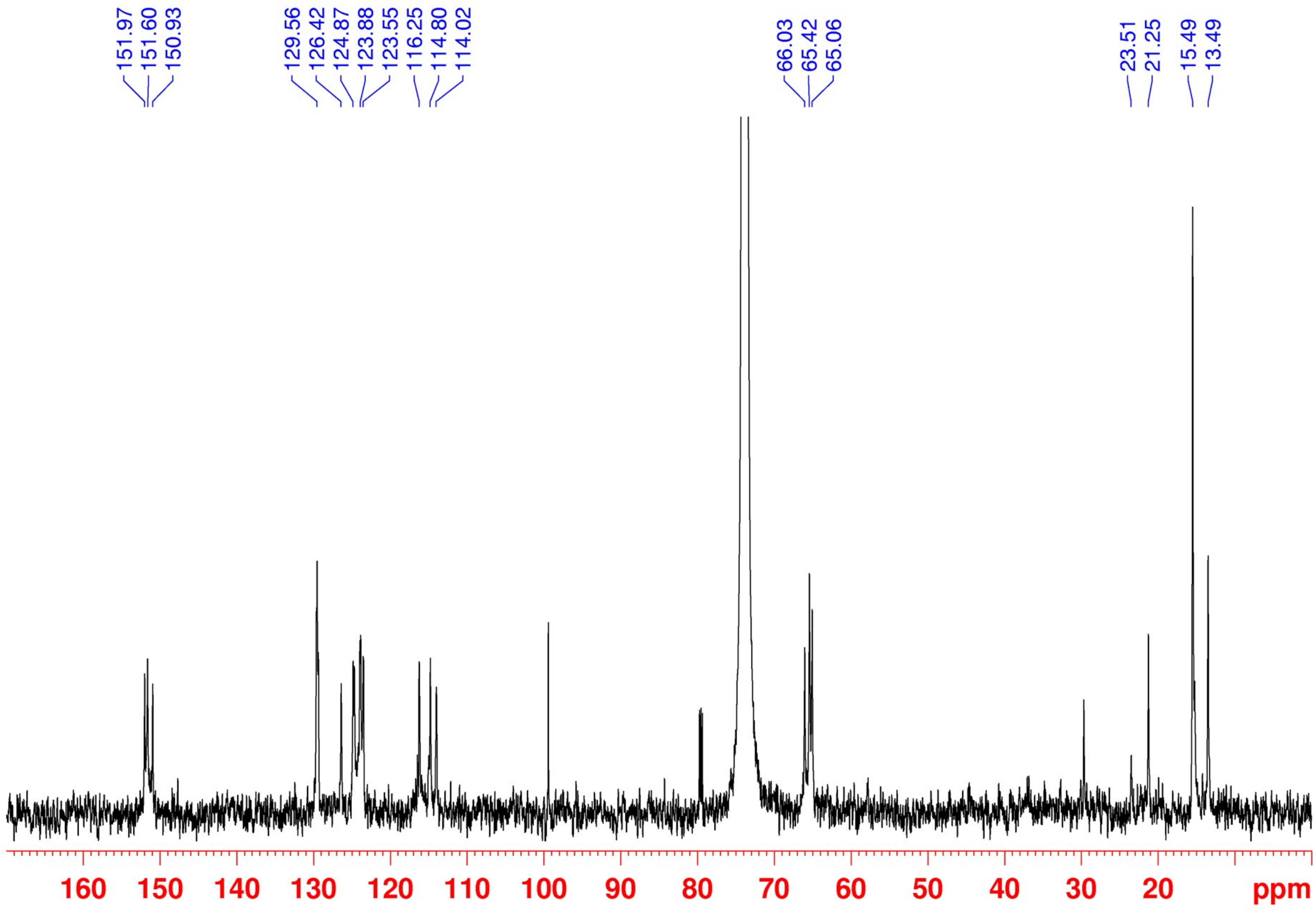

Figure S12: ^{13}C NMR spectra of **PrS[6]*Et*** (TCDE, 150 MHz, 298 K).

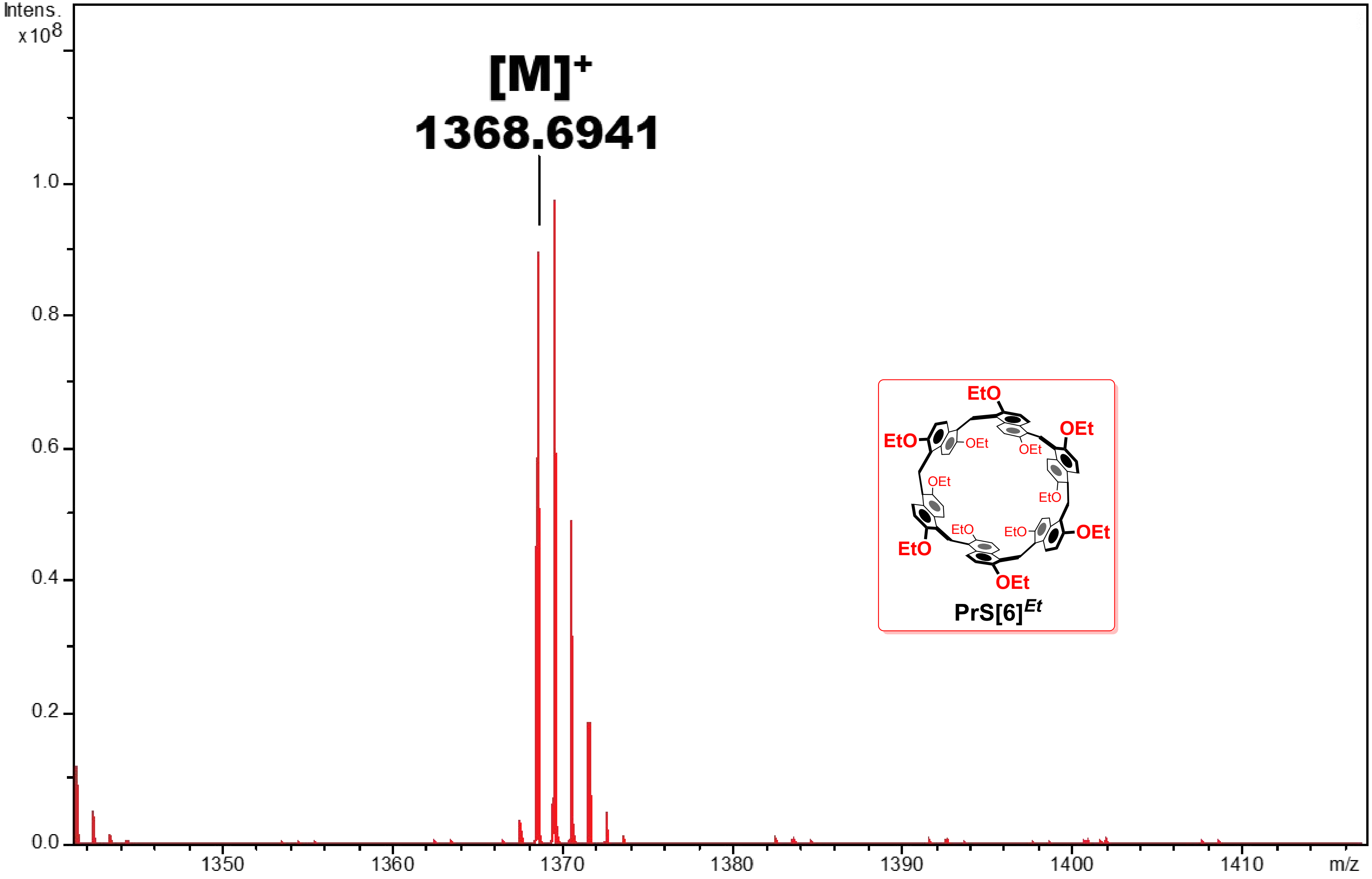

Figure S13: Significant portion of the HR MALDI FT-ICR mass spectrum of **PrS[6]*Et*** $[M]^+$.

Copies of NMR and HR mass spectrum of PrS[6]^{nPr}

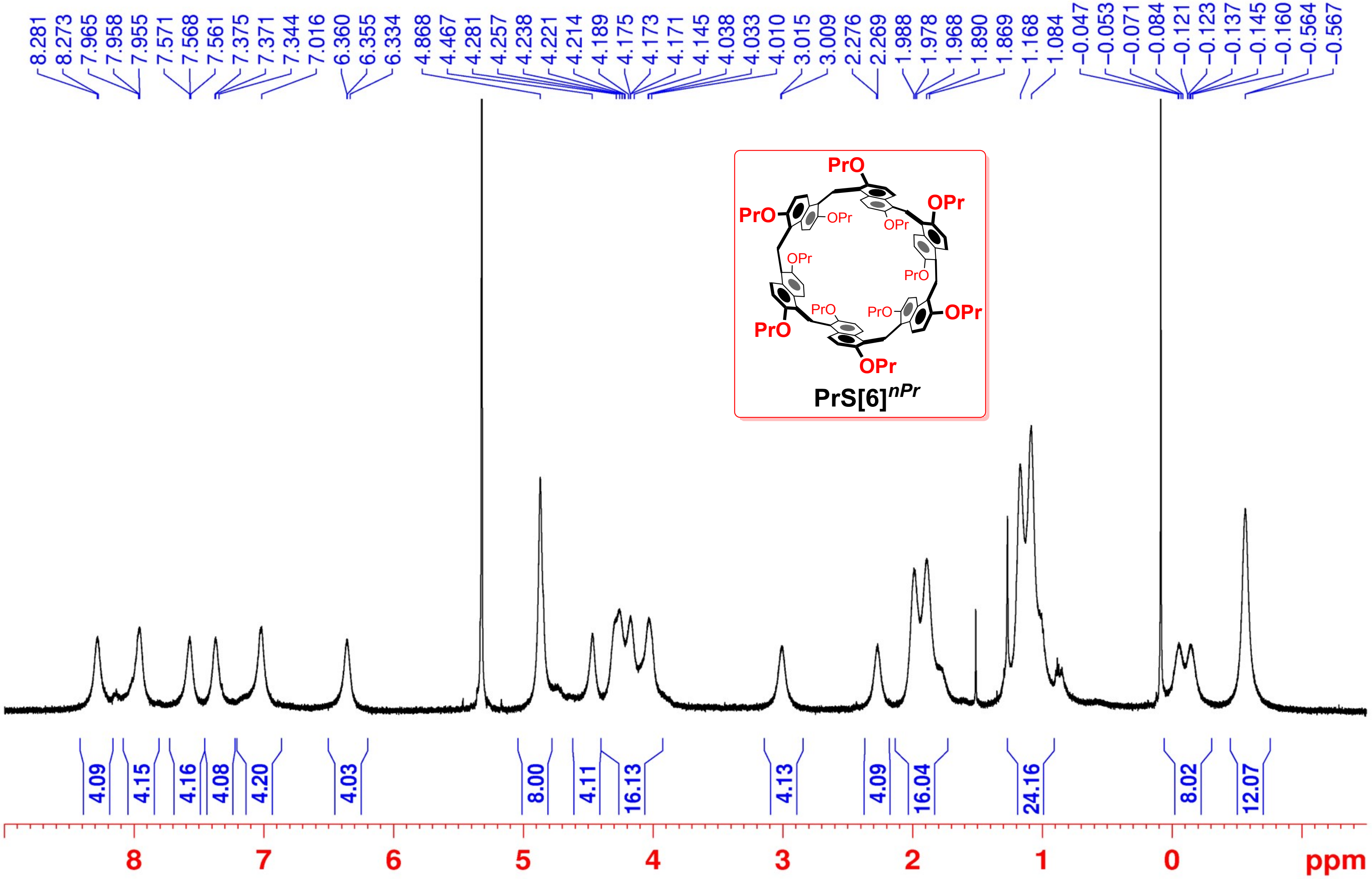

Figure S14: ^{1}H NMR spectrum of **PrS[6]^{nPr}** (CD_2Cl_2, 600 MHz, 298 K).

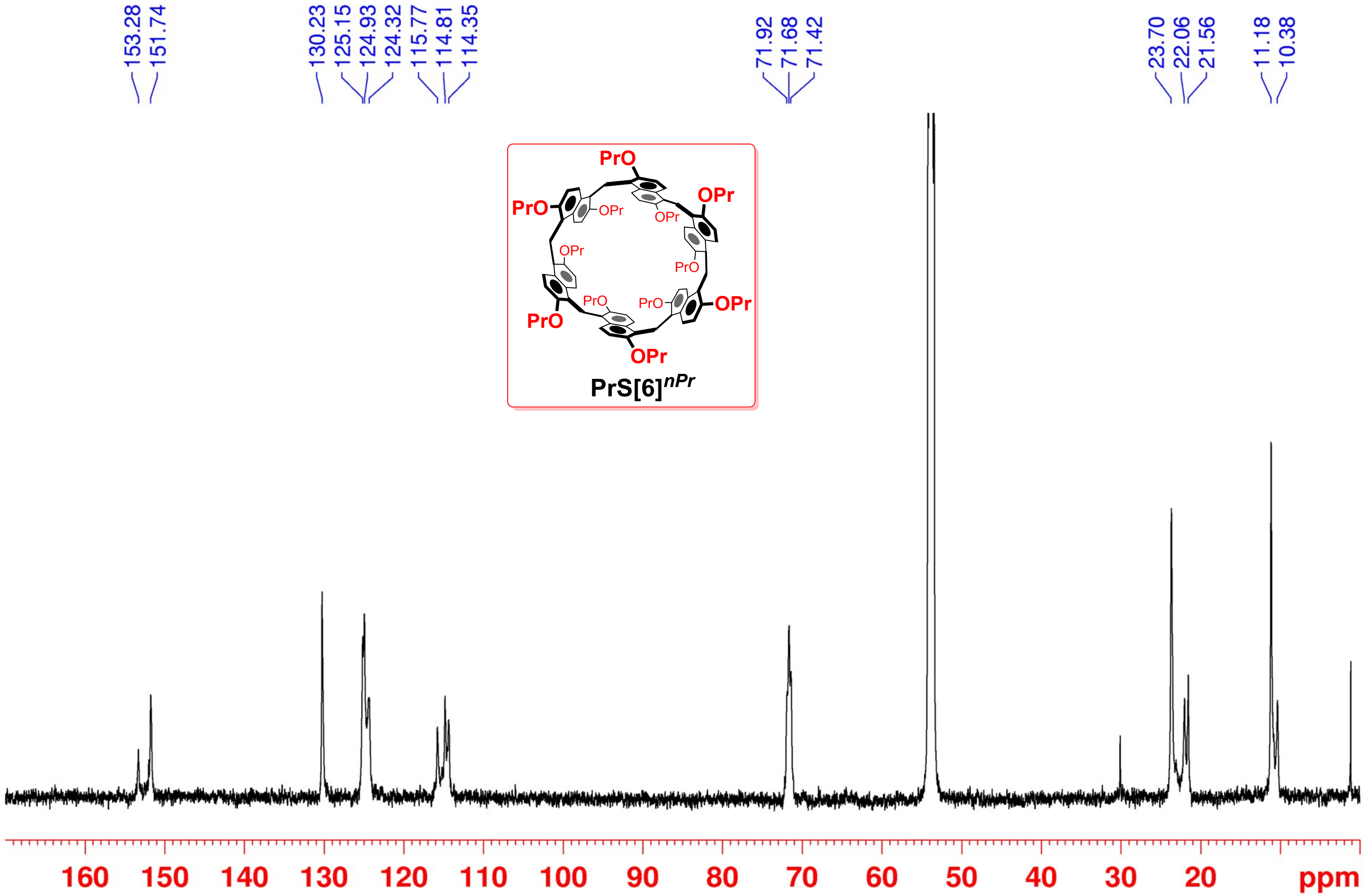

Figure S15: ^{13}C NMR spectra of **PrS[6]**^{nPr} (CD_2Cl_2, 150 MHz, 298 K).

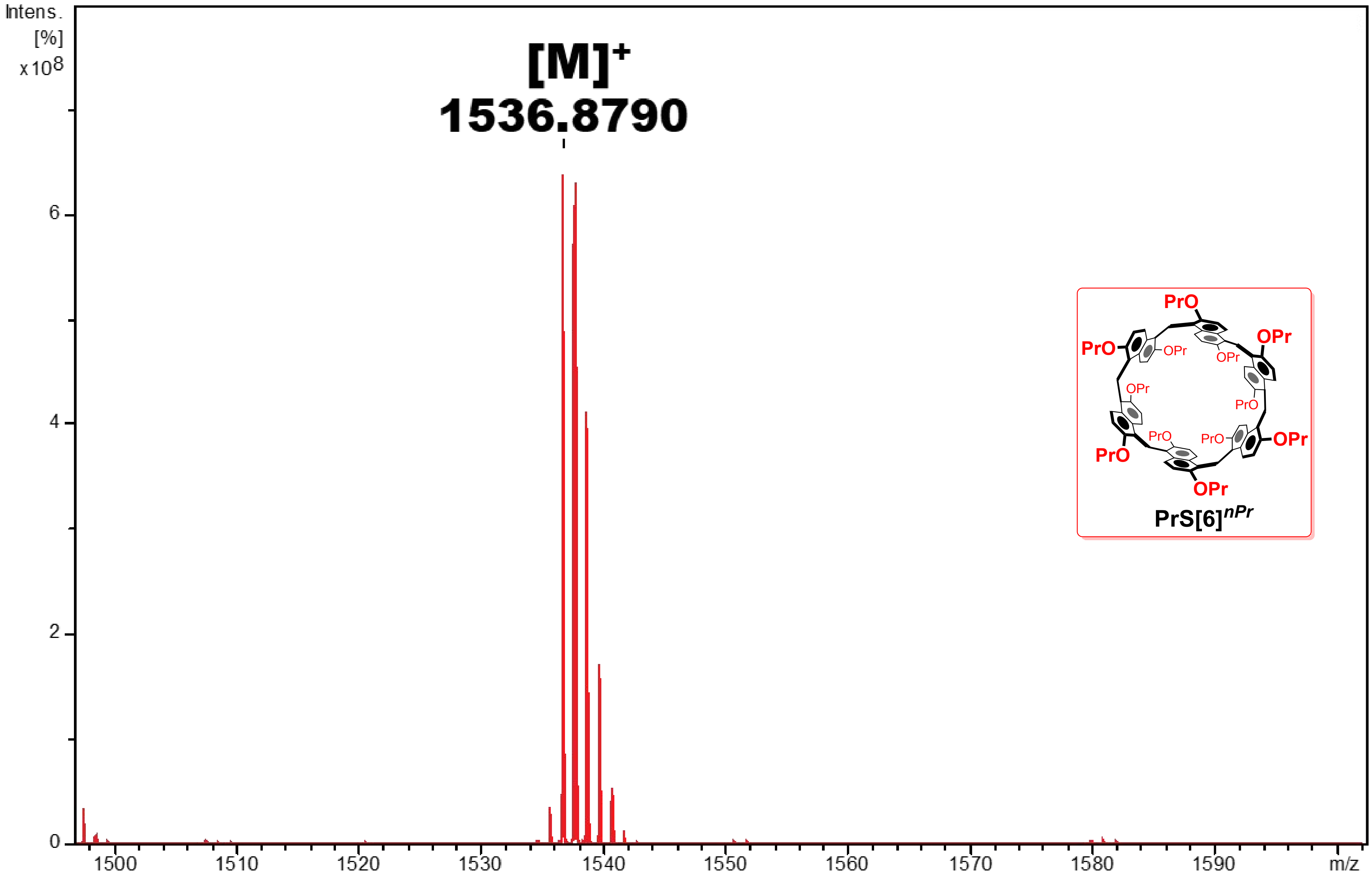

Figure S16: Significant portion of the HR MALDI FT-ICR mass spectrum of **PrS[6]*nPr*** $[M]^+$.

Copies of NMR and HR mass spectrum of PrS[6]^{nBu}

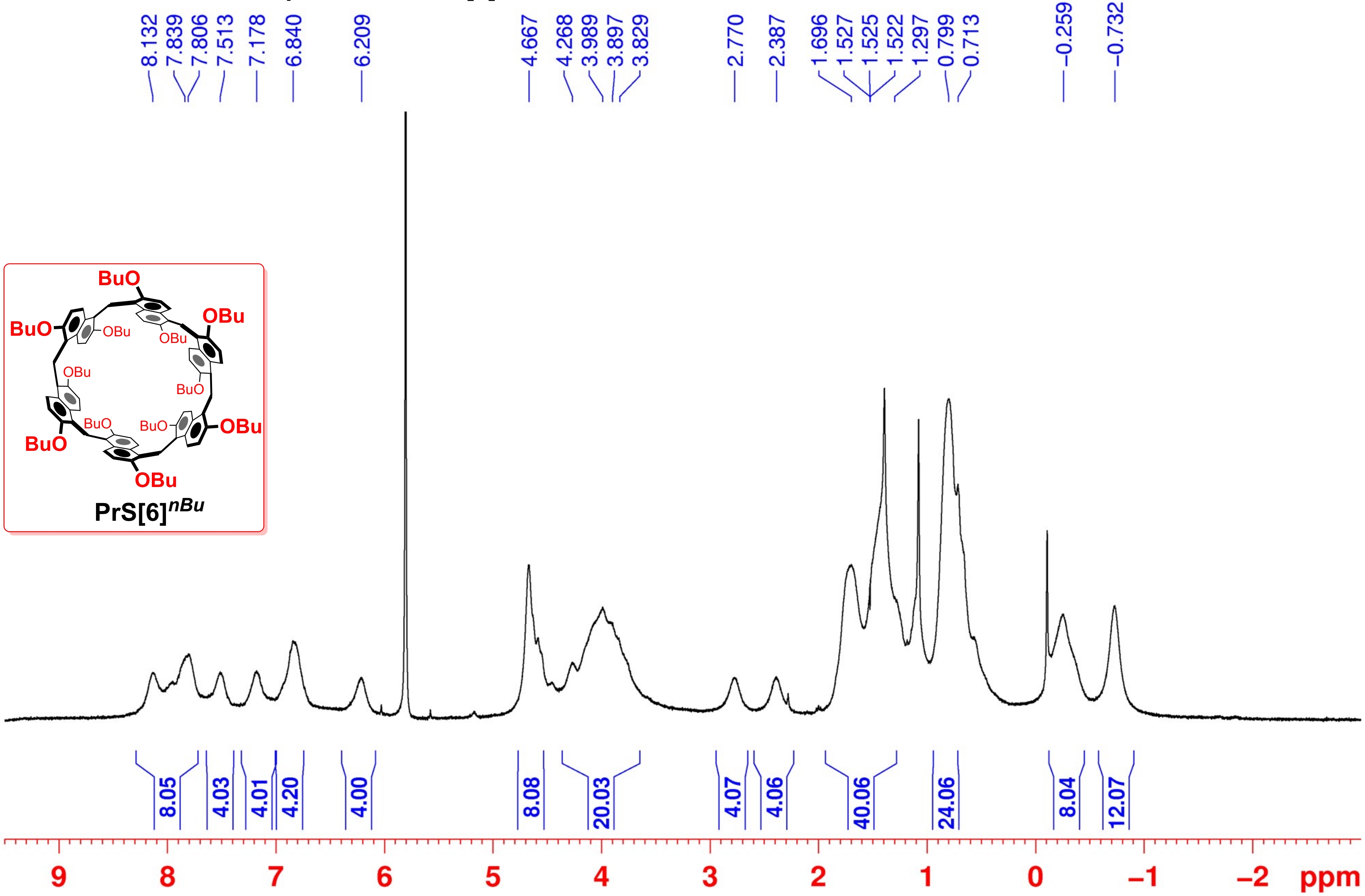

Figure S17: ^{1}H NMR spectrum of **PrS[6]^{nBu}** (TCDE, 400 MHz, 298 K).

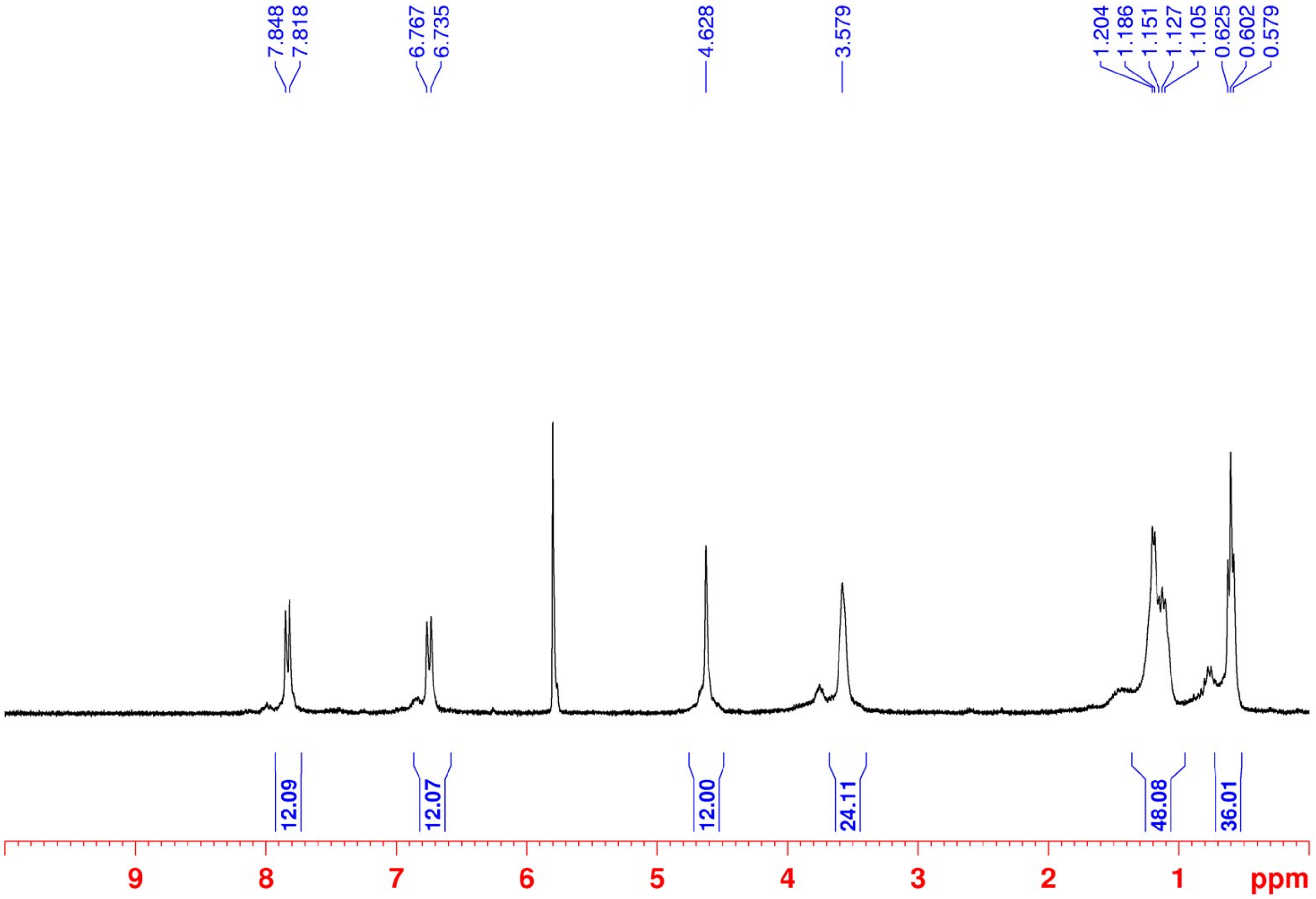

Figure S18: ^{1}H NMR spectrum of **PrS[6]^{nBu}** (TCDE, 400 MHz, 393 K).

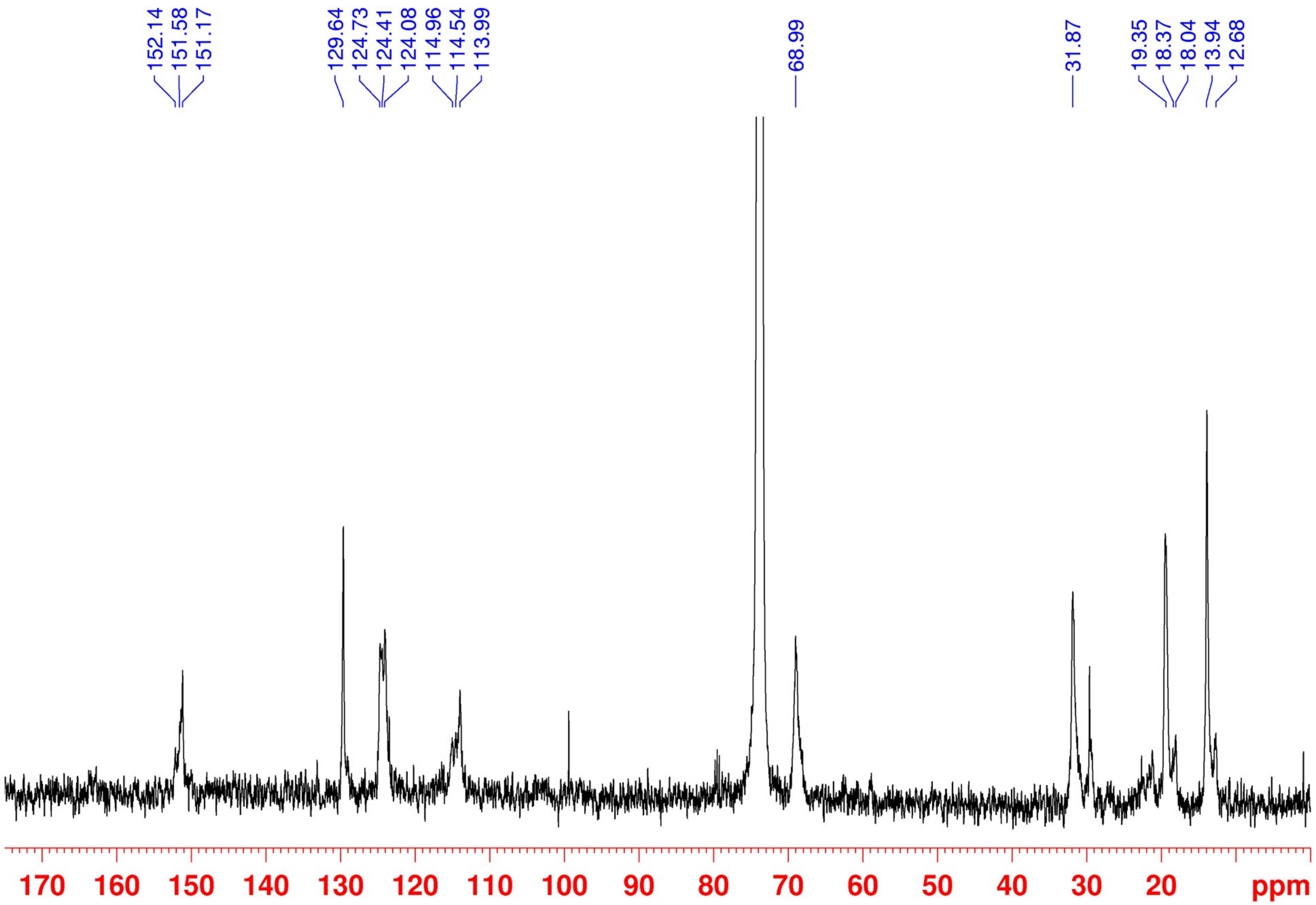

Figure S19: ^{13}C NMR spectra of **PrS[6]**^{nBu} (TCDE,100 MHz, 298 K).

Copies of NMR and HR mass spectrum of PrS[6]^{nPn}

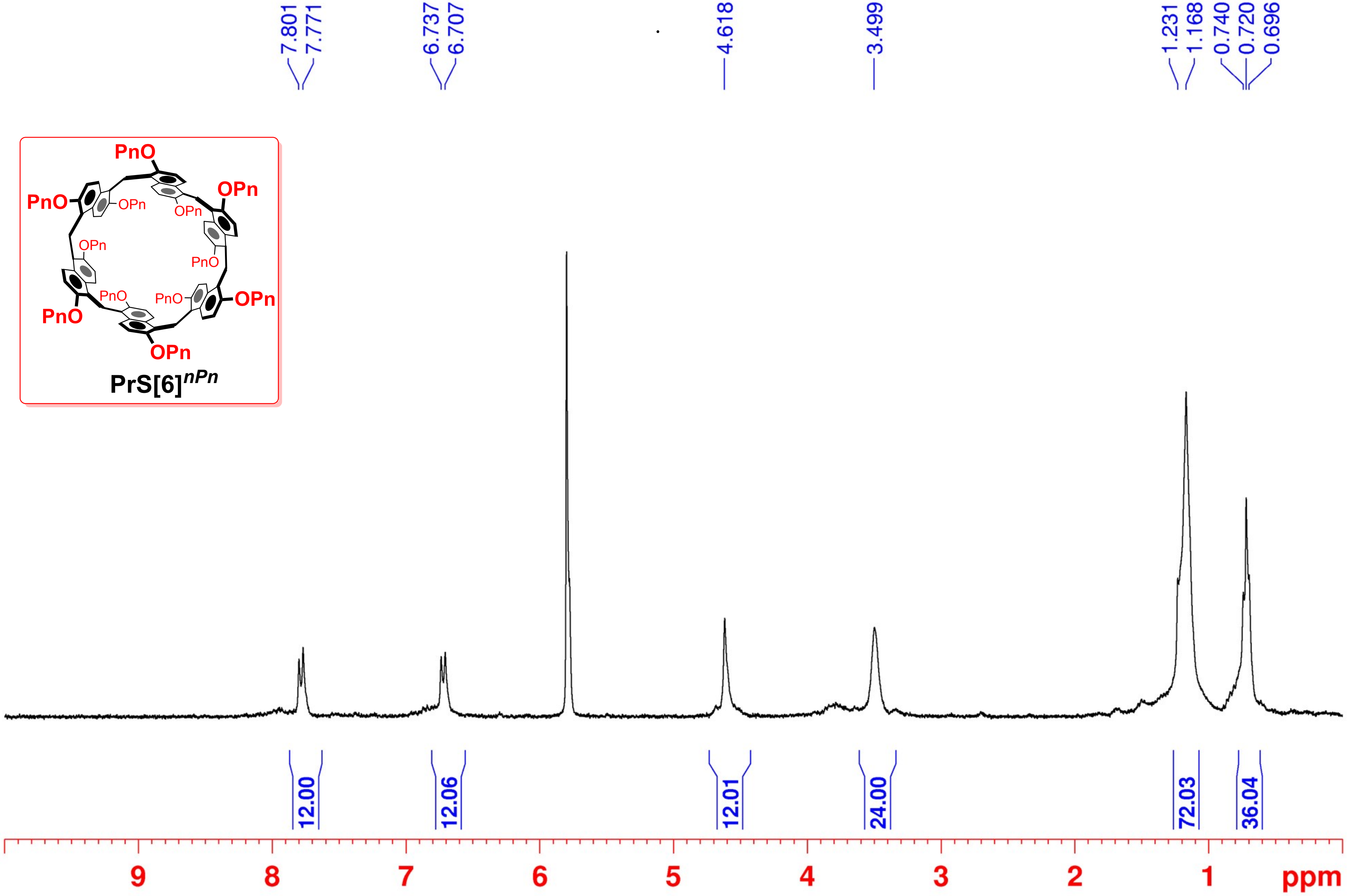

Figure S20: ^{1}H NMR spectrum of **PrS[6]^{nPn}** (TCDE, 300 MHz, 393 K).

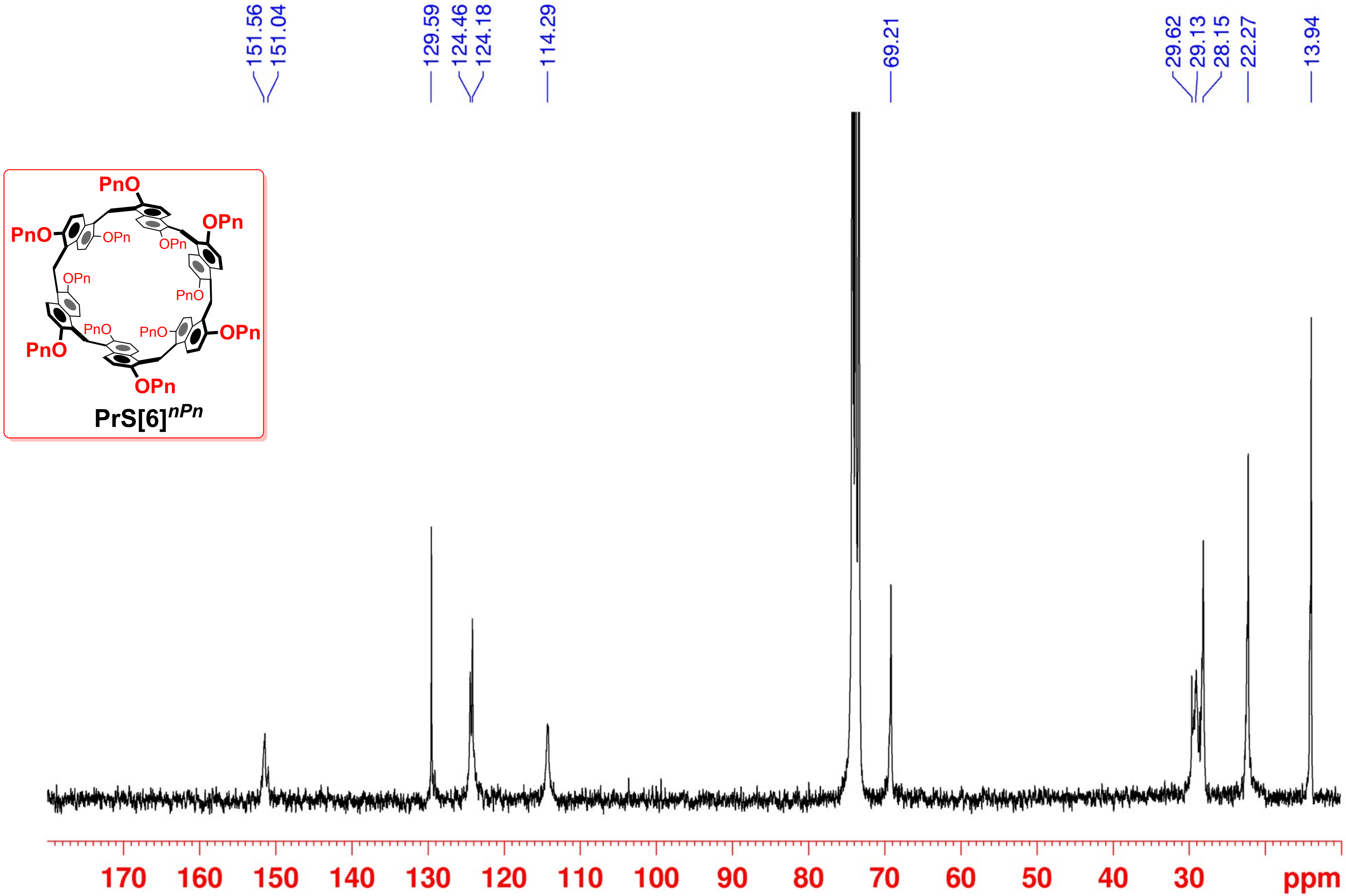

Figure S21: ^{13}C NMR spectra of **PrS[6]**^{nPn} (TCDE, 62.5 MHz, 298 K).

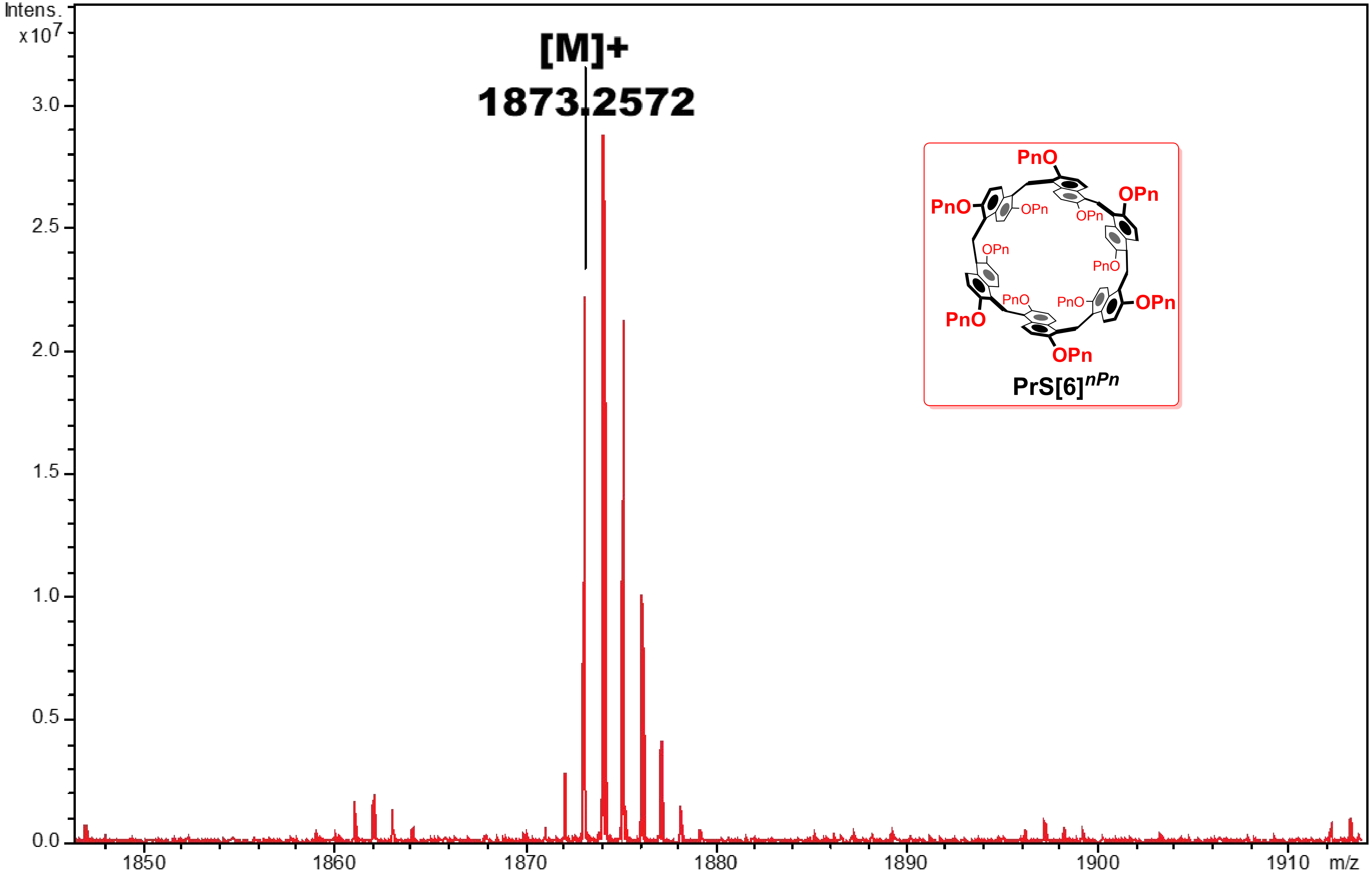

Figure S22: Significant portion of the HR MALDI FT-ICR mass spectrum of **PrS[6]*nPn*** $[M]^{+}$.

Low temperature 1D and 2D NMR spectra of PrS[6]^{Et}

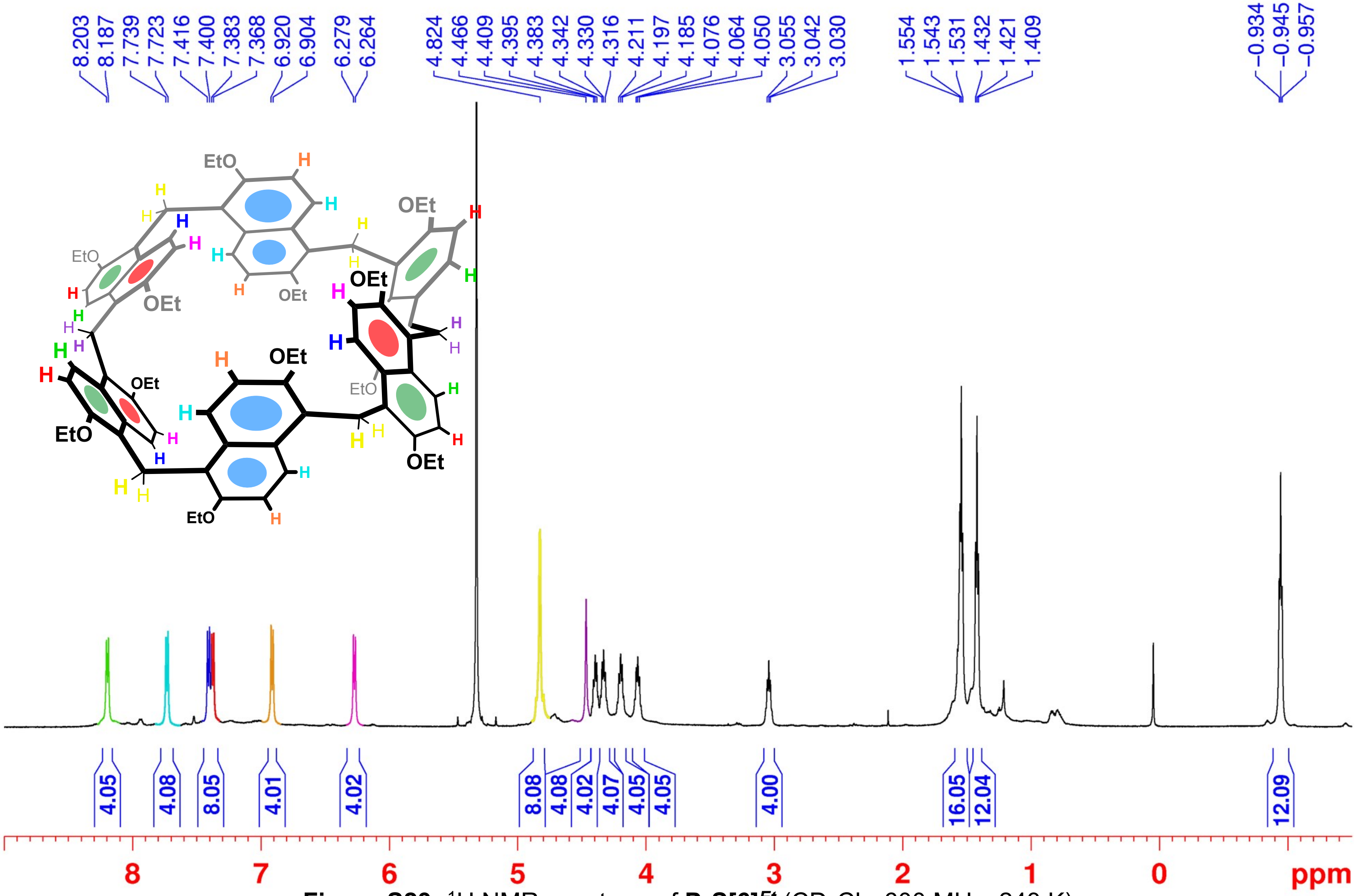

Figure S23: ^{1}H NMR spectrum of **PrS[6]^{Et}** (CD_2Cl_2, 600 MHz, 243 K).

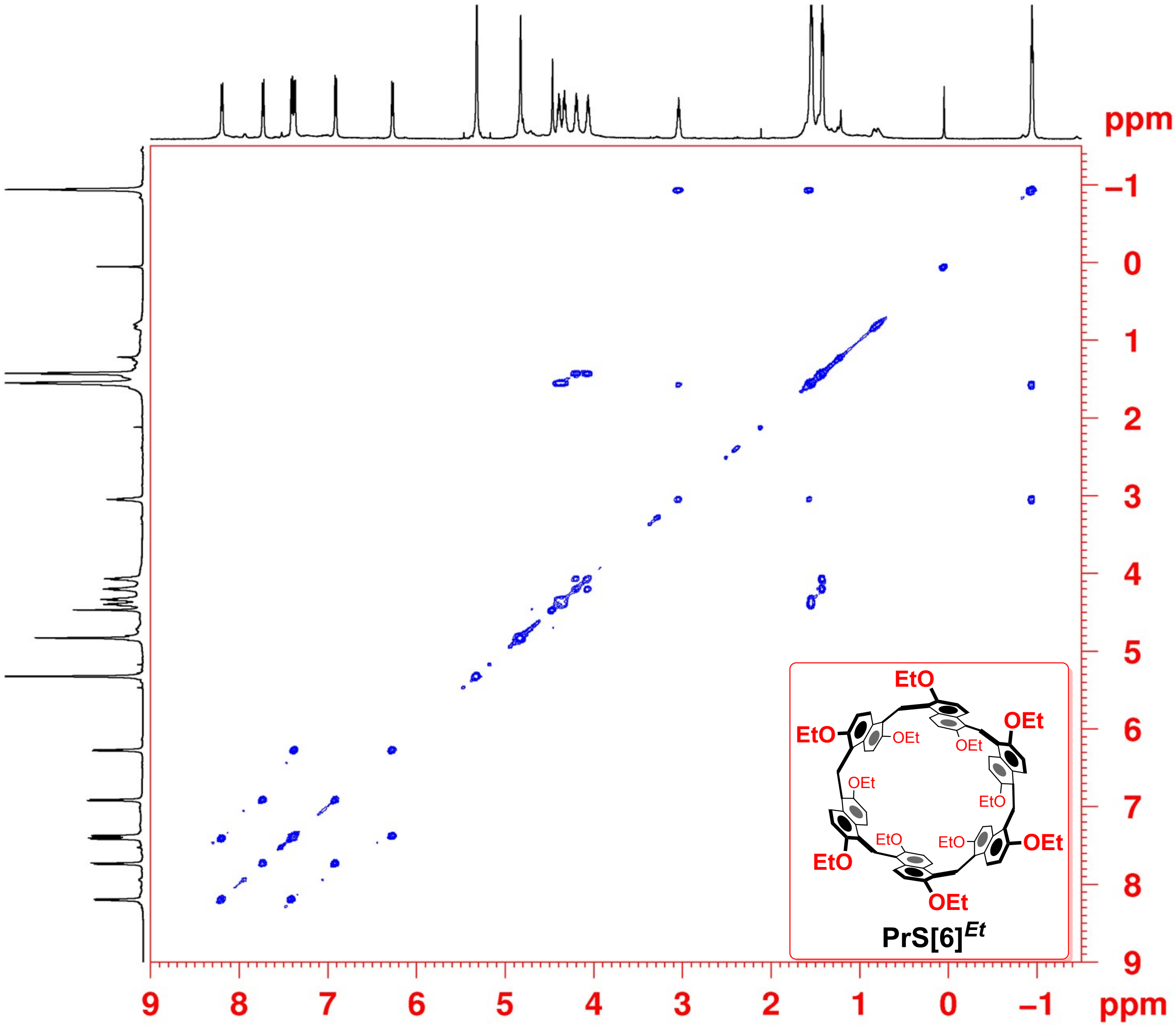

Figure S24: 2D-DQF COSY spectrum of **PrS[6]*Et*** (CD_2Cl_2, 600 MHz, 243 K).

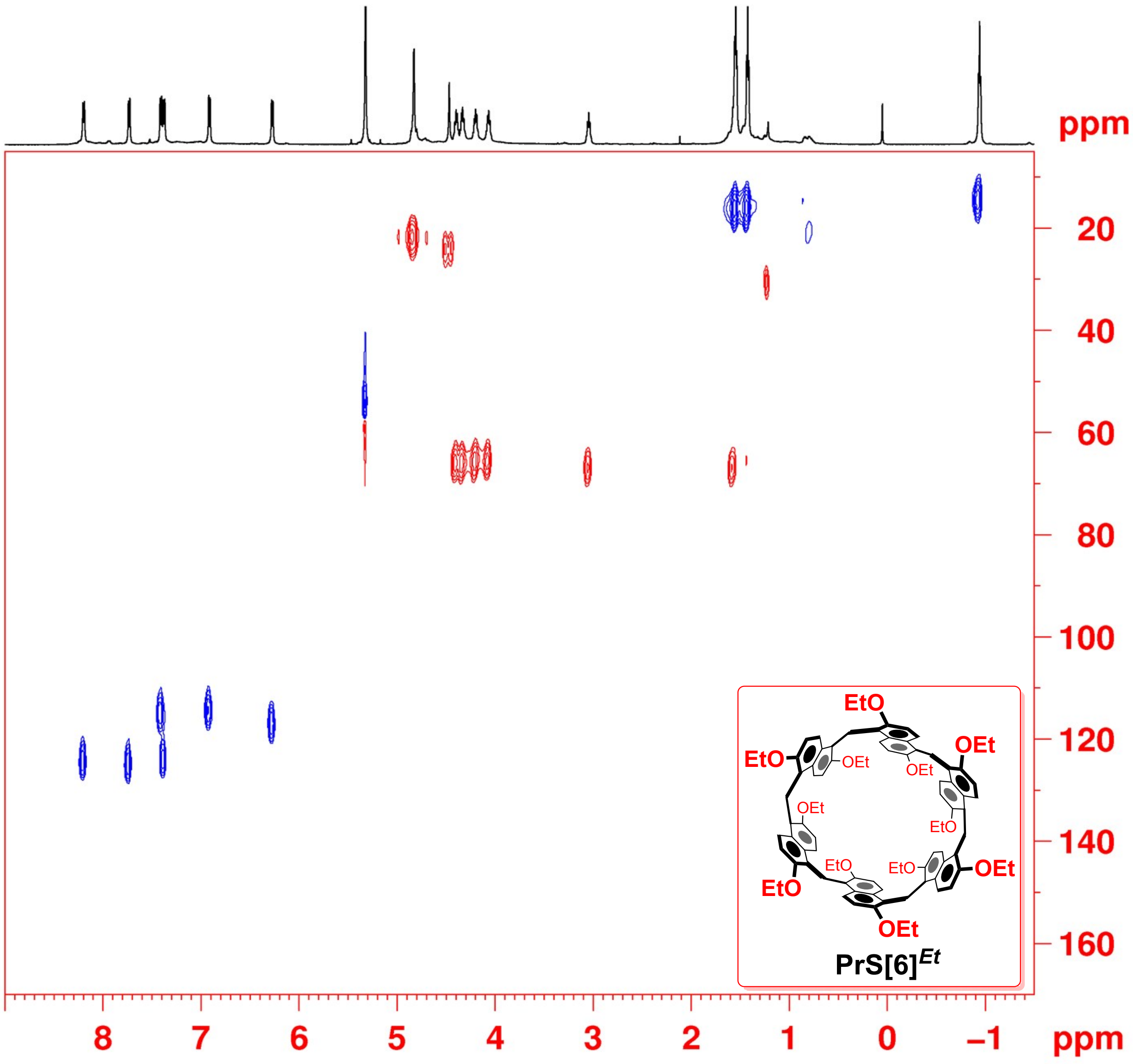

Figure S25: 2D-HSQC spectrum of **PrS[6]^{Et}** (CD_2Cl_2, 600 MHz, 243 K).

Low temperature 1D and 2D NMR spectra of PrS[6]^{nPr}

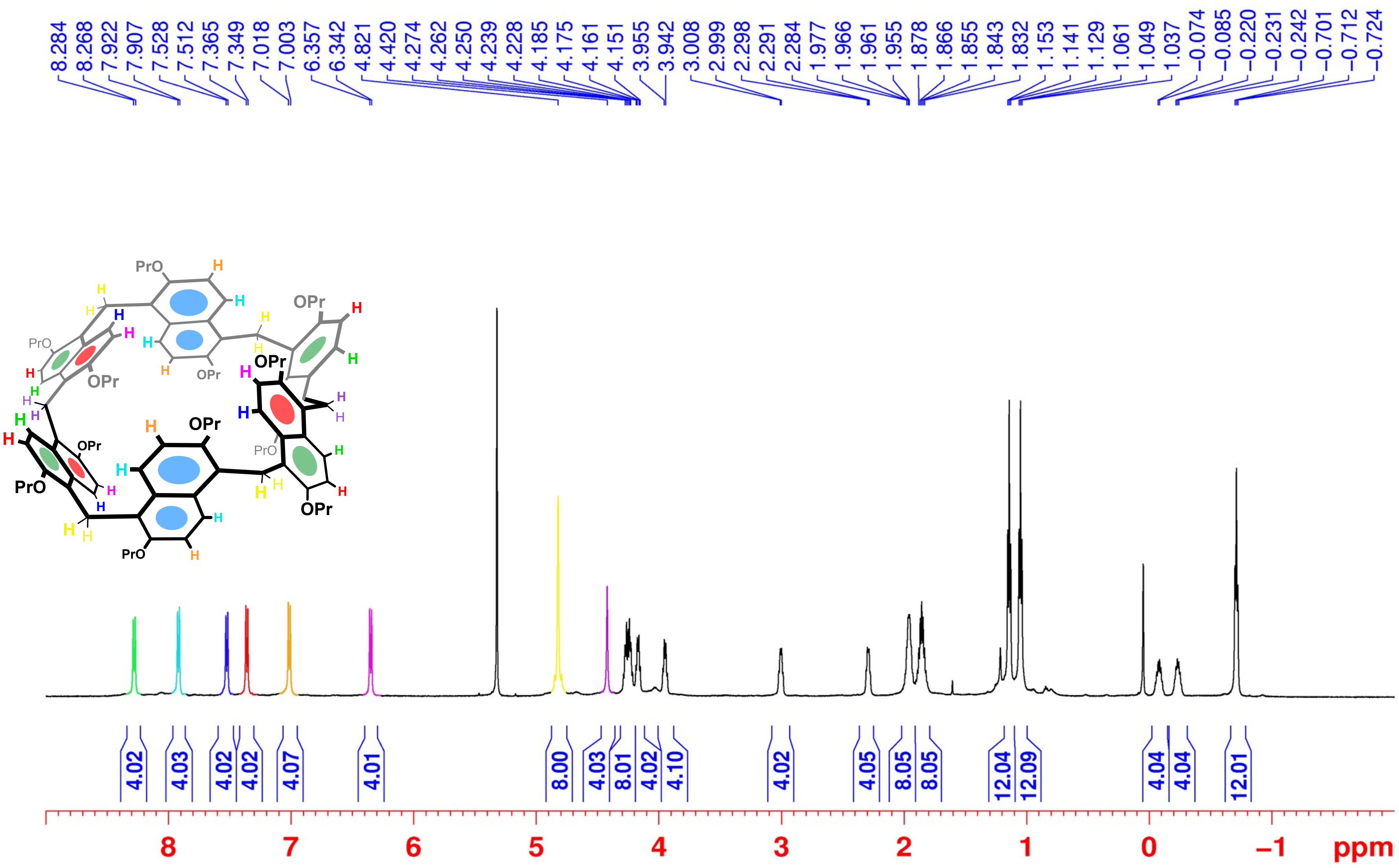

Figure S26: ^{1}H NMR spectrum of **PrS[6]^{nPr}** (CD_2Cl_2, 600 MHz, 243 K).

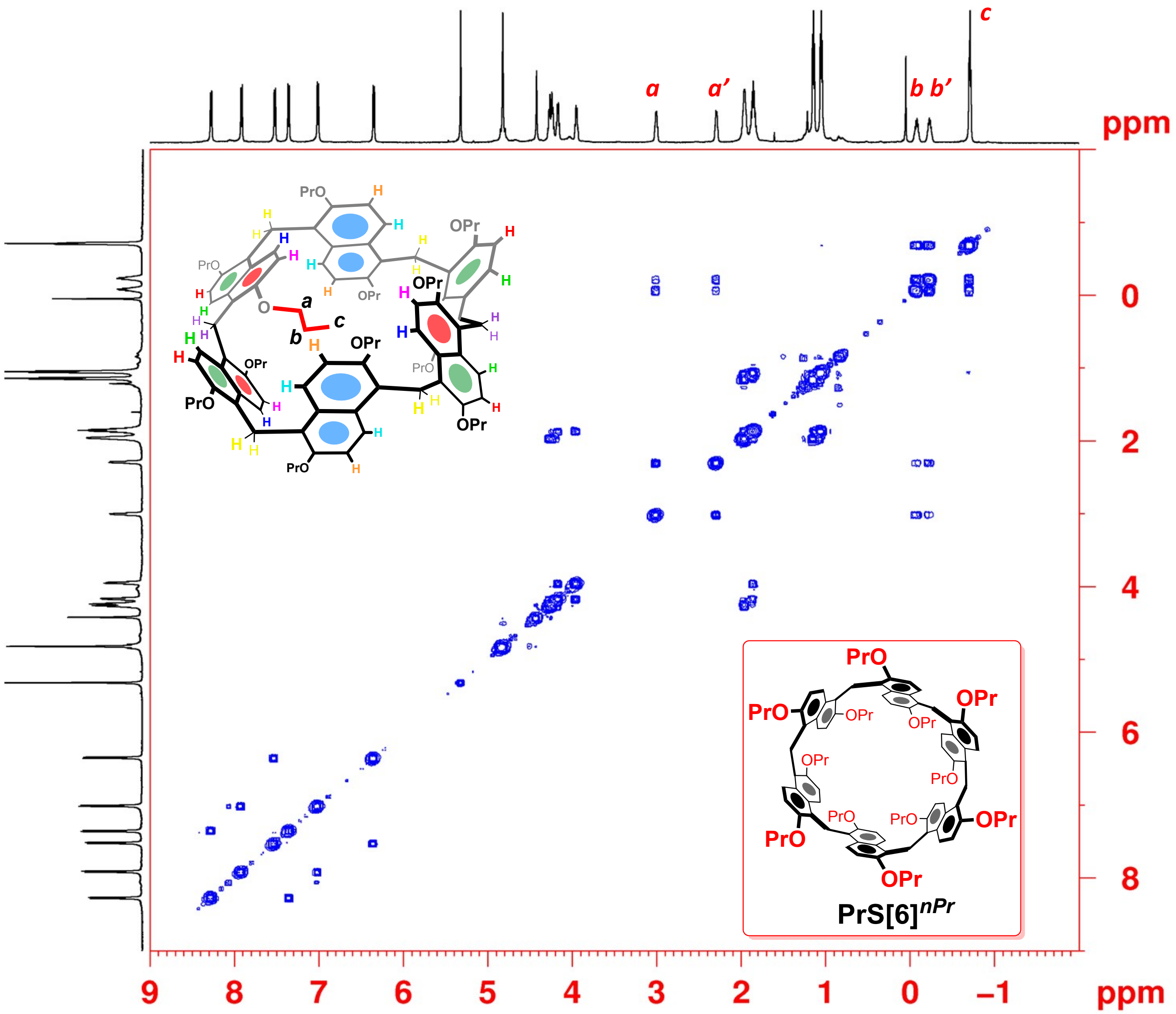

Figure S27: 2D-DQF COSY spectrum of **PrS[6]^{nPr}** (CD_2Cl_2, 600 MHz, 243 K).

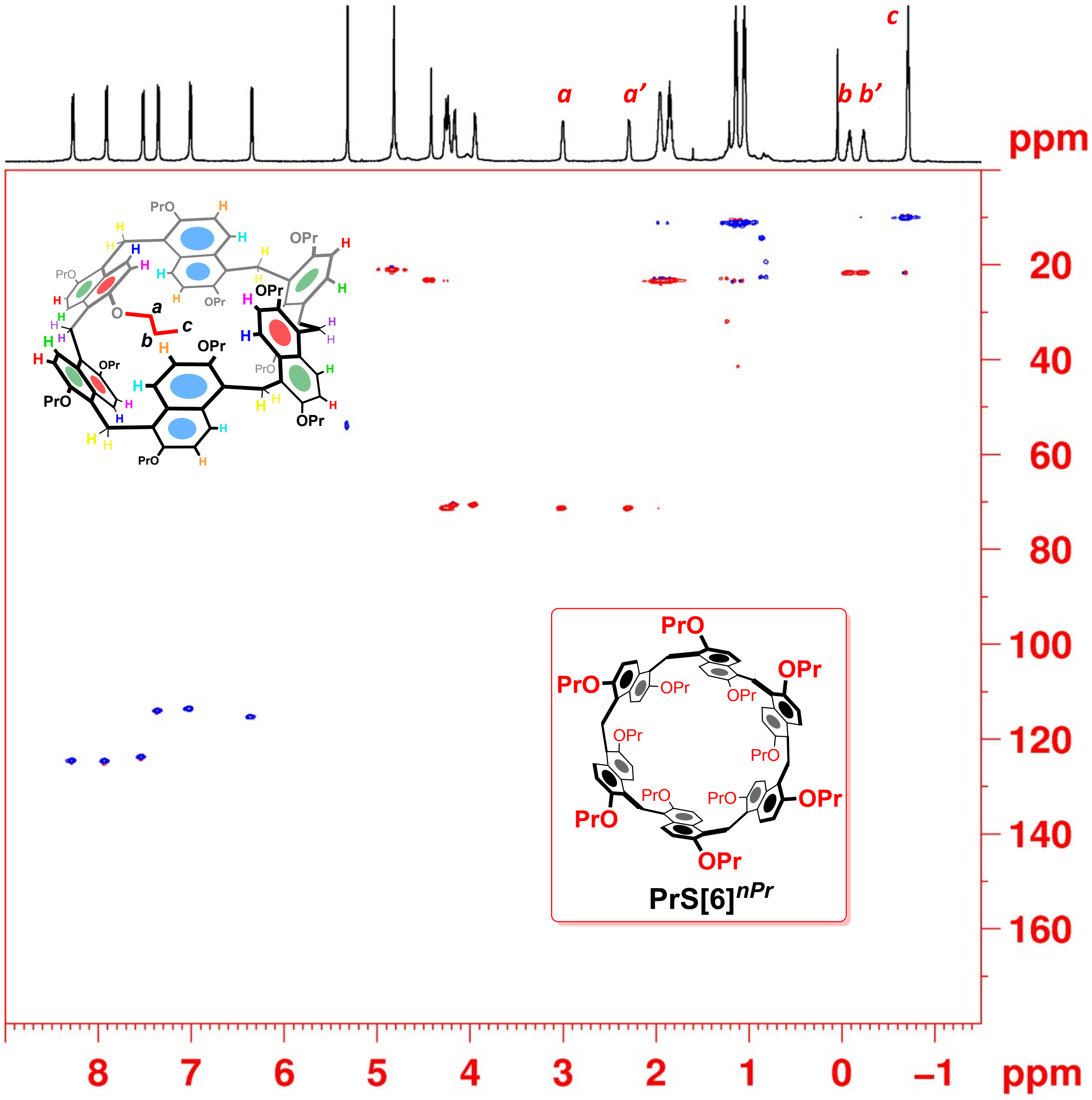

Figure S28: 2D-HSQC spectrum of **PrS[6]*^{nPr}*** (CD_2Cl_2, 600 MHz, 243 K).

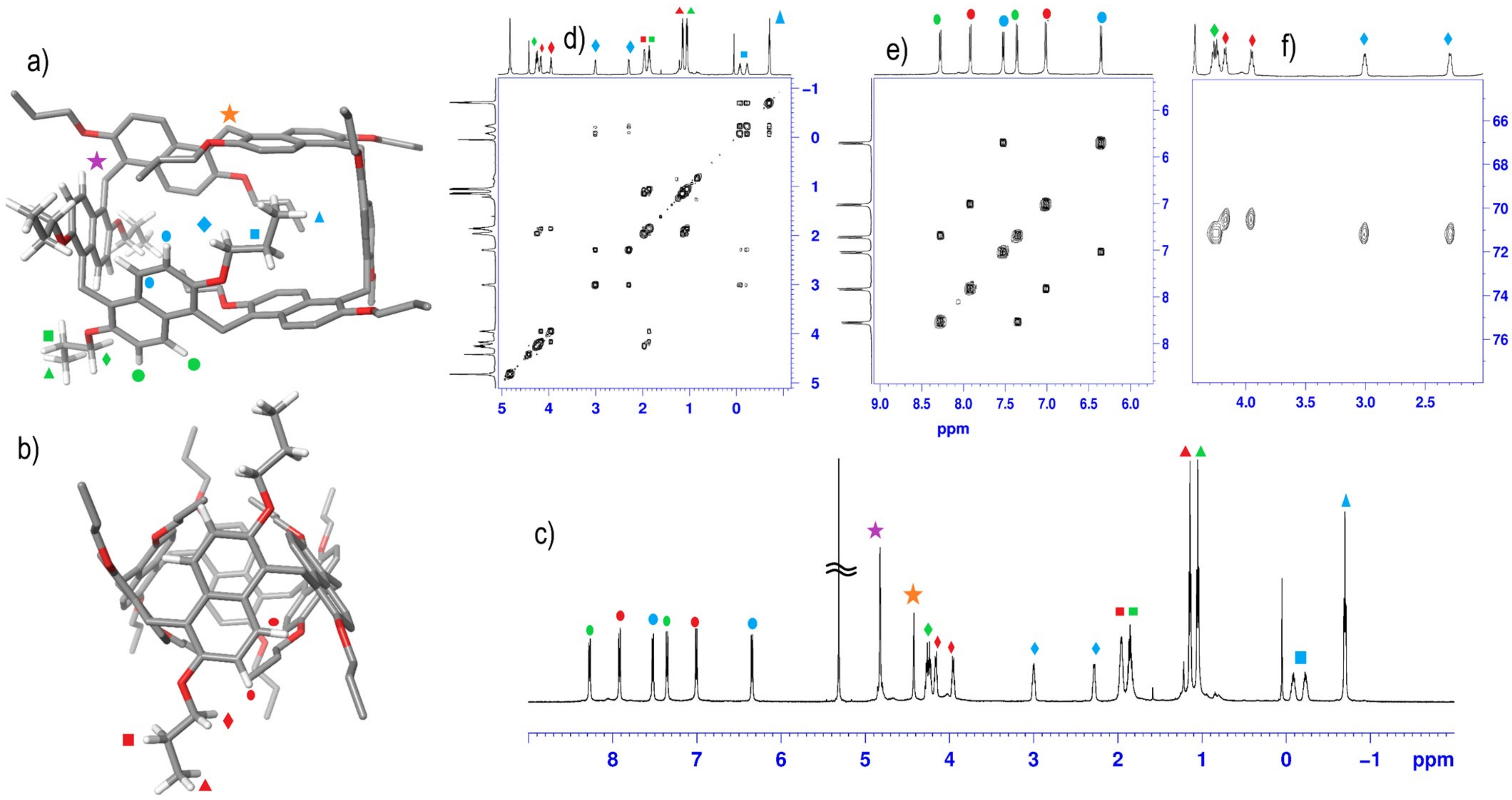

Figure S29. (a and b) Different views of the DFT-optimized structure of **PrS[6]*nPr*** at the B97D3/SVP/SVPFIT level of theory. (c) ^{1}H NMR spectrum of **PrS[6]*nPr*** in CD_2Cl_2 at 243 K (600 MHz). (d,e) Significant portions of the 2D COSY spectrum of **PrS[6]*nPr*** (600 MHz, CD_2Cl_2, 243 K). (f) Significant portions of the 2D HSQC spectrum of **PrS[6]*nPr*** in which the resonances of distereotopic OCH_2 groups are reported. The assignment of ^{1}H NMR signals is indicated by the colored symbols of (a and b).

Copies of NMR titration experiments with reaction solvents

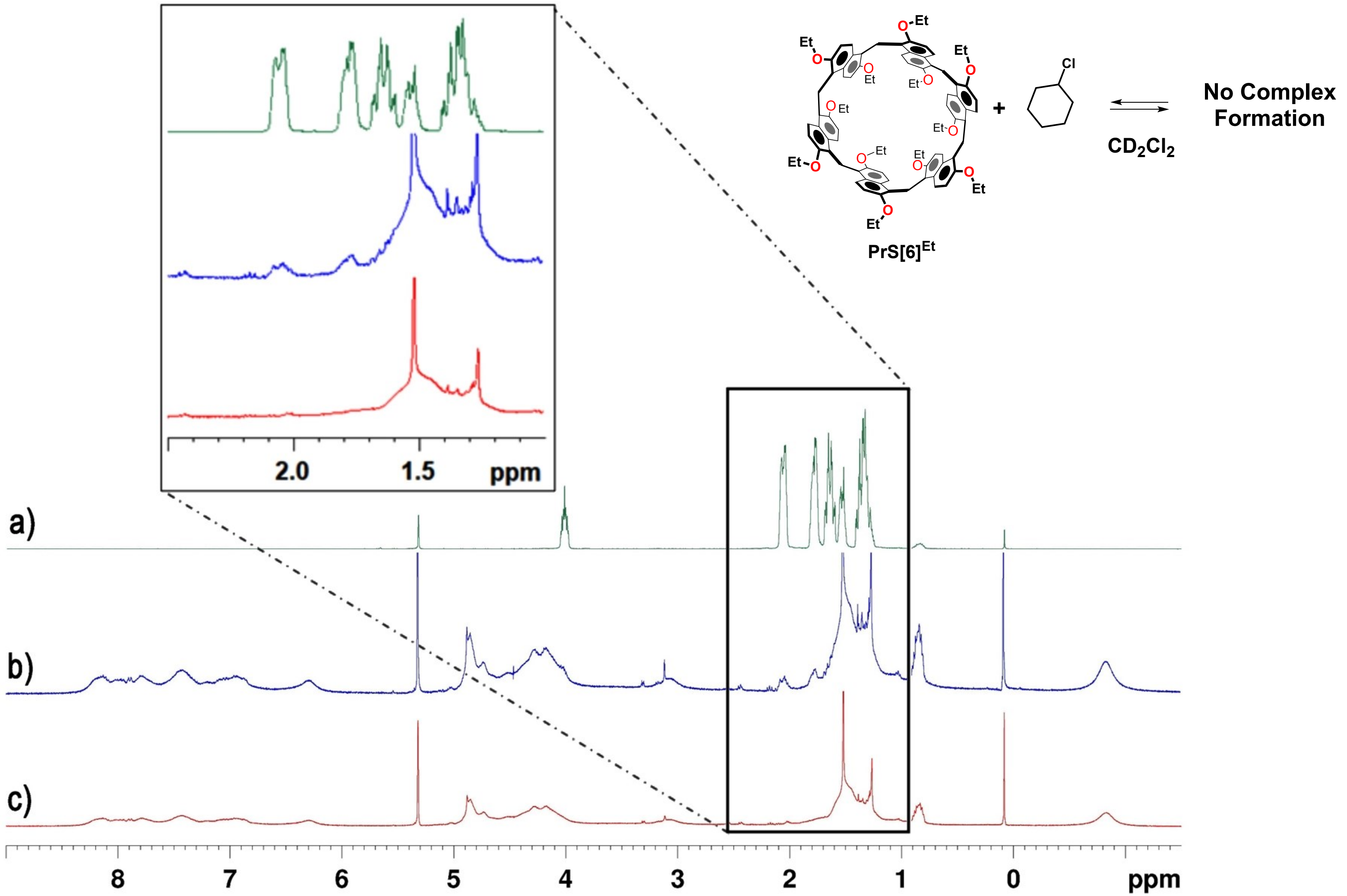

Figure S30: ^{1}H NMR spectra (400 MHz, CD_2Cl_2, 298 K) of: (a) chlorocyclohexane, (b) an equimolar solution of **PrS[6]*^Et*** and chlorocyclohexane and (c) **PrS[6]*^Et***.

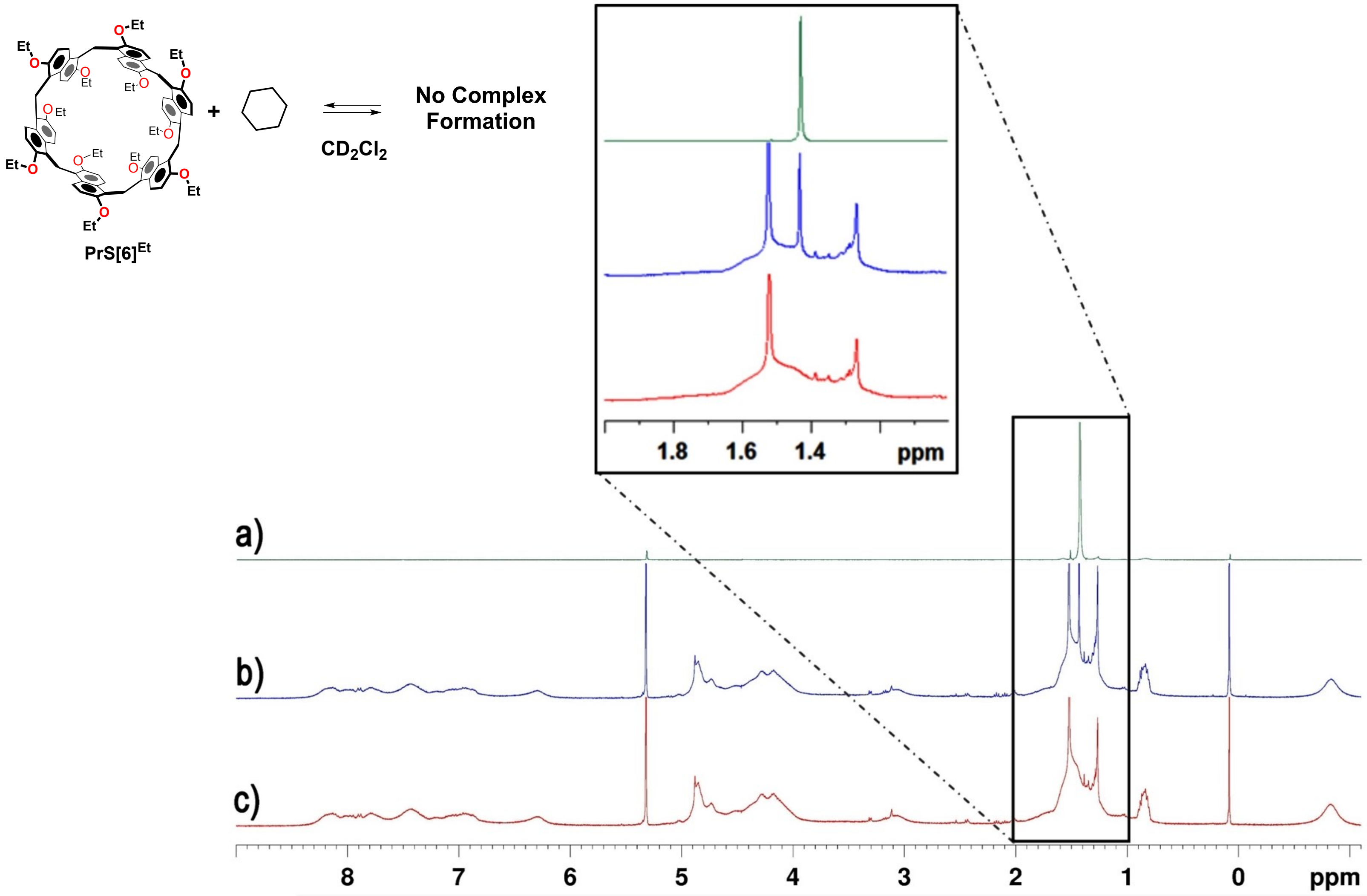

Figure S31: ^{1}H NMR spectra (400 MHz, CD_2Cl_2, 298 K) of: (a) cyclohexane, (b) an equimolar solution of **PrS[6]^{Et}** and cyclohexane and (c) **PrS[6]^{Et}**.

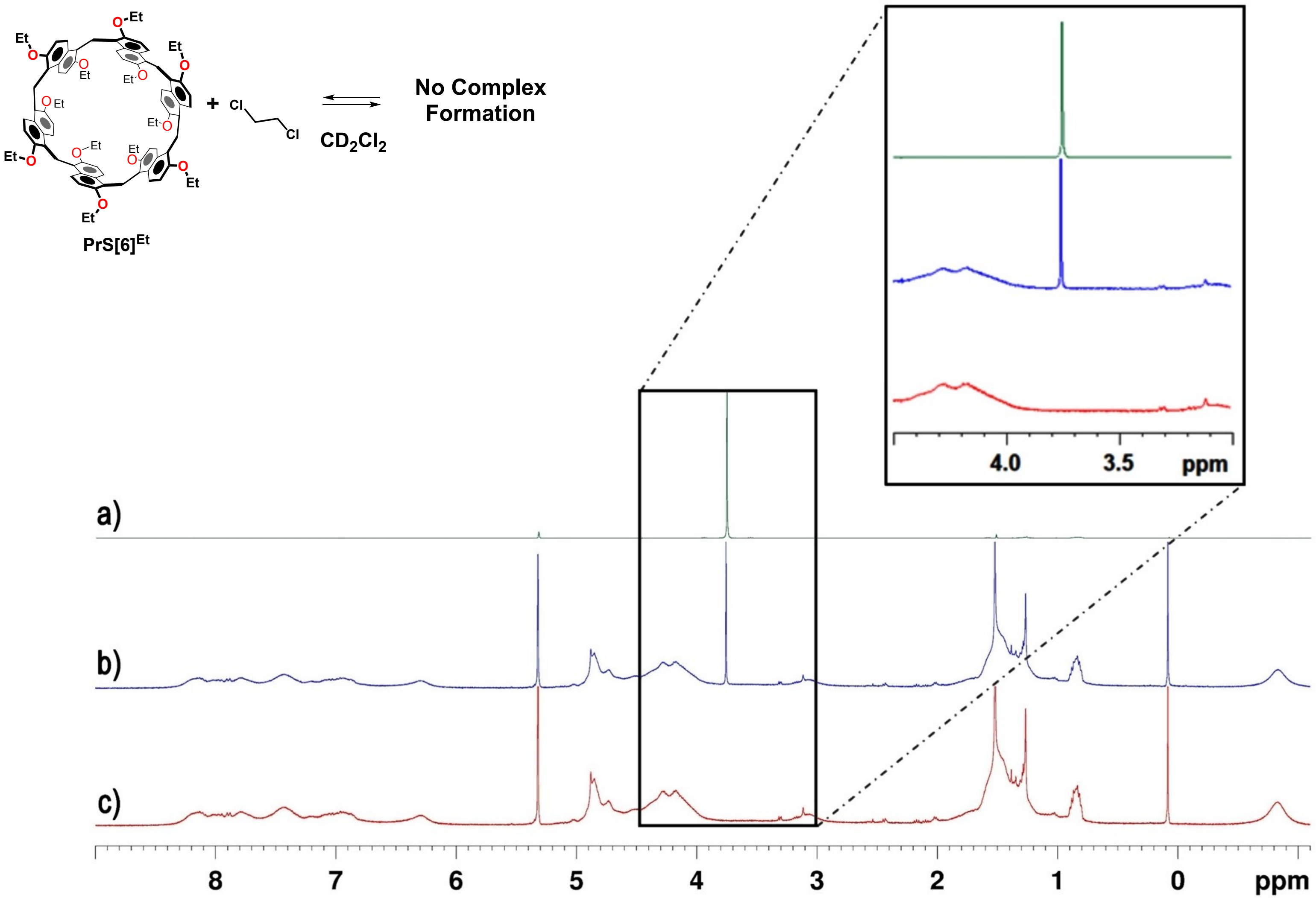

Figure S32: ^{1}H NMR spectra (400 MHz, CD_2Cl_2, 298 K) of: (a) 1,2-dichloroethane, (b) an equimolar solution of **PrS[6]*^{Et}*** and 1,2-dichloroethane and (c) **PrS[6]*^{Et}***.

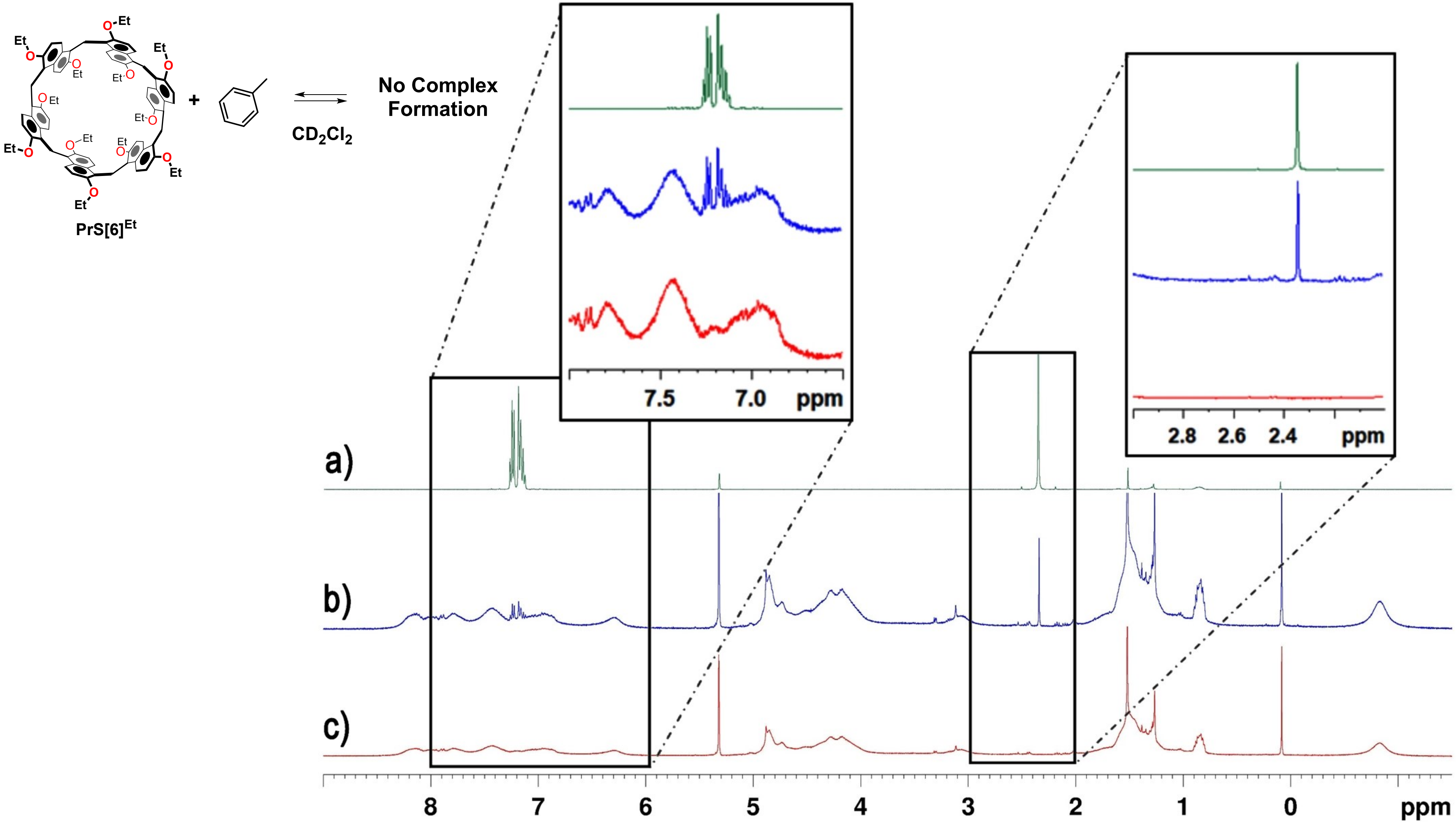

Figure S33: ^{1}H NMR spectra (400 MHz, CD_2Cl_2, 298 K) of: (a) toluene, (b) an equimolar solution of **PrS[6]**^{Et} and Toluene and (c) **PrS[6]**^{Et}.

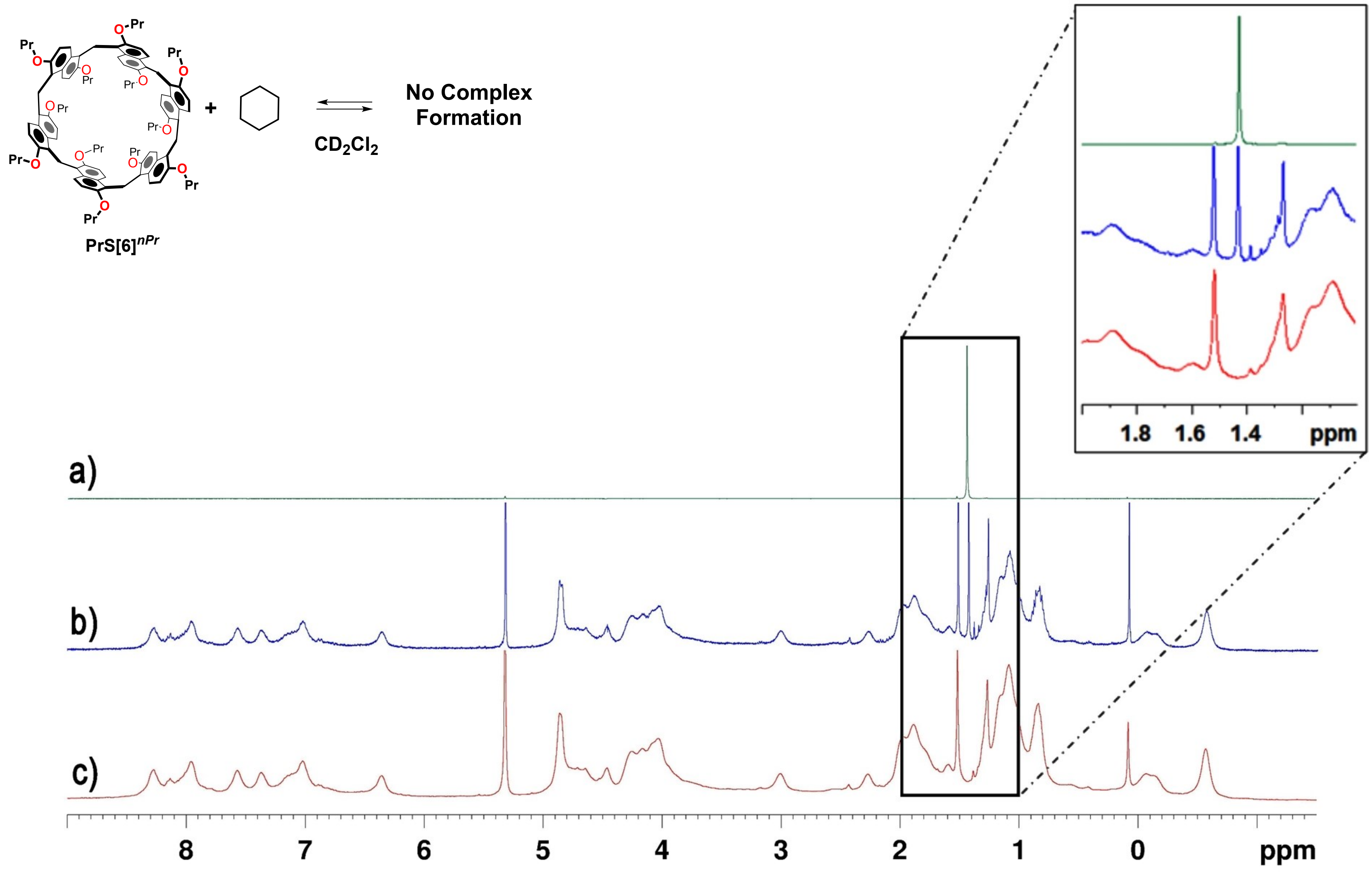

Figure S34: ^{1}H NMR spectra (400 MHz, CD_2Cl_2, 298 K) of: (a) cyclohexane, (b) an equimolar solution of **PrS[6]**^{nPr} and cyclohexane and (c) **PrS[6]**^{nPr}.

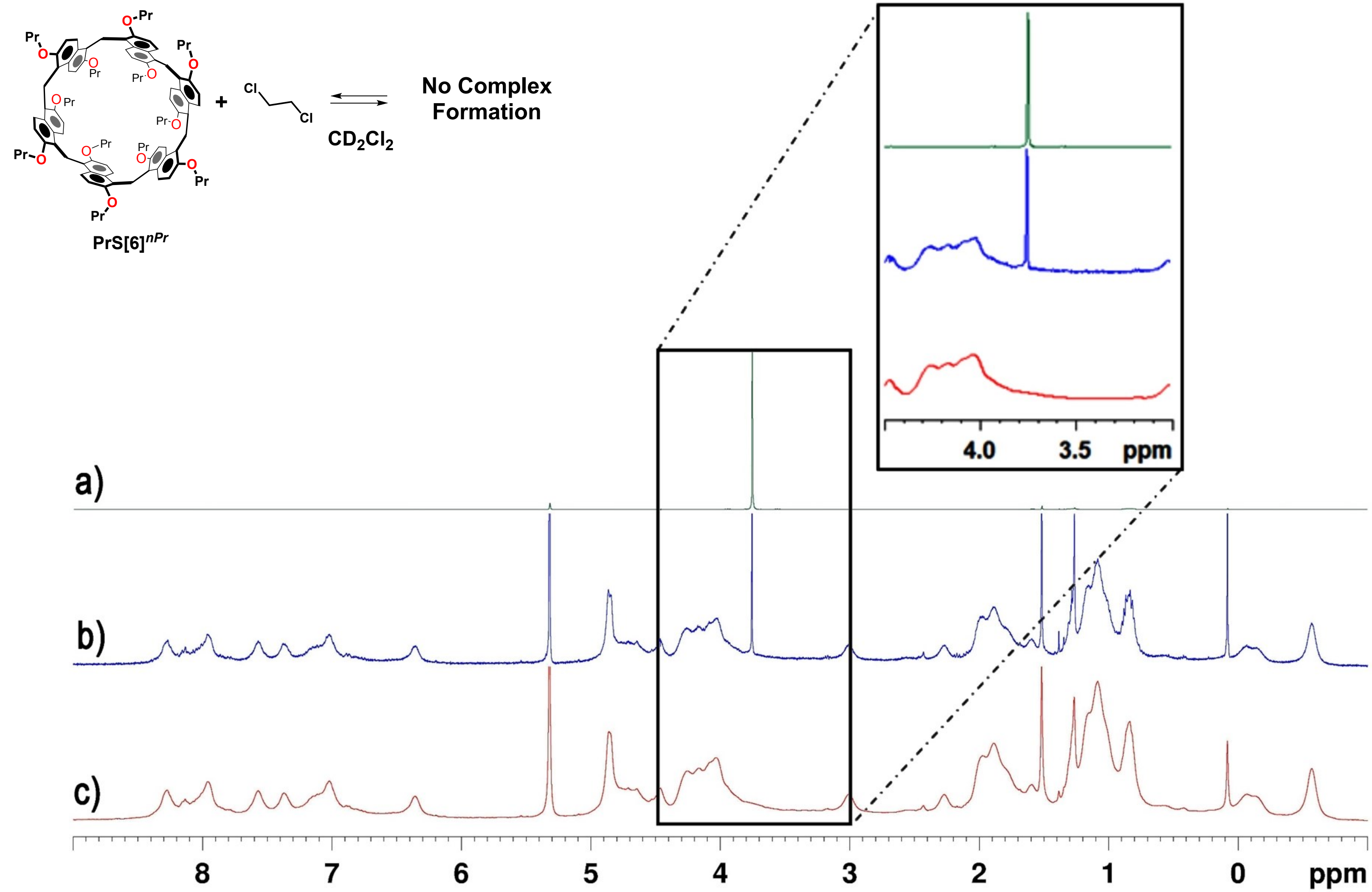

Figure S35: ^{1}H NMR spectra (400 MHz, CD_2Cl_2, 298 K) of: (a) 1,2-dichloroethane, (b) an equimolar solution of PrS[6]^{nPr} and 1,2-dichloroethane and (c) PrS[6]^{nPr}.

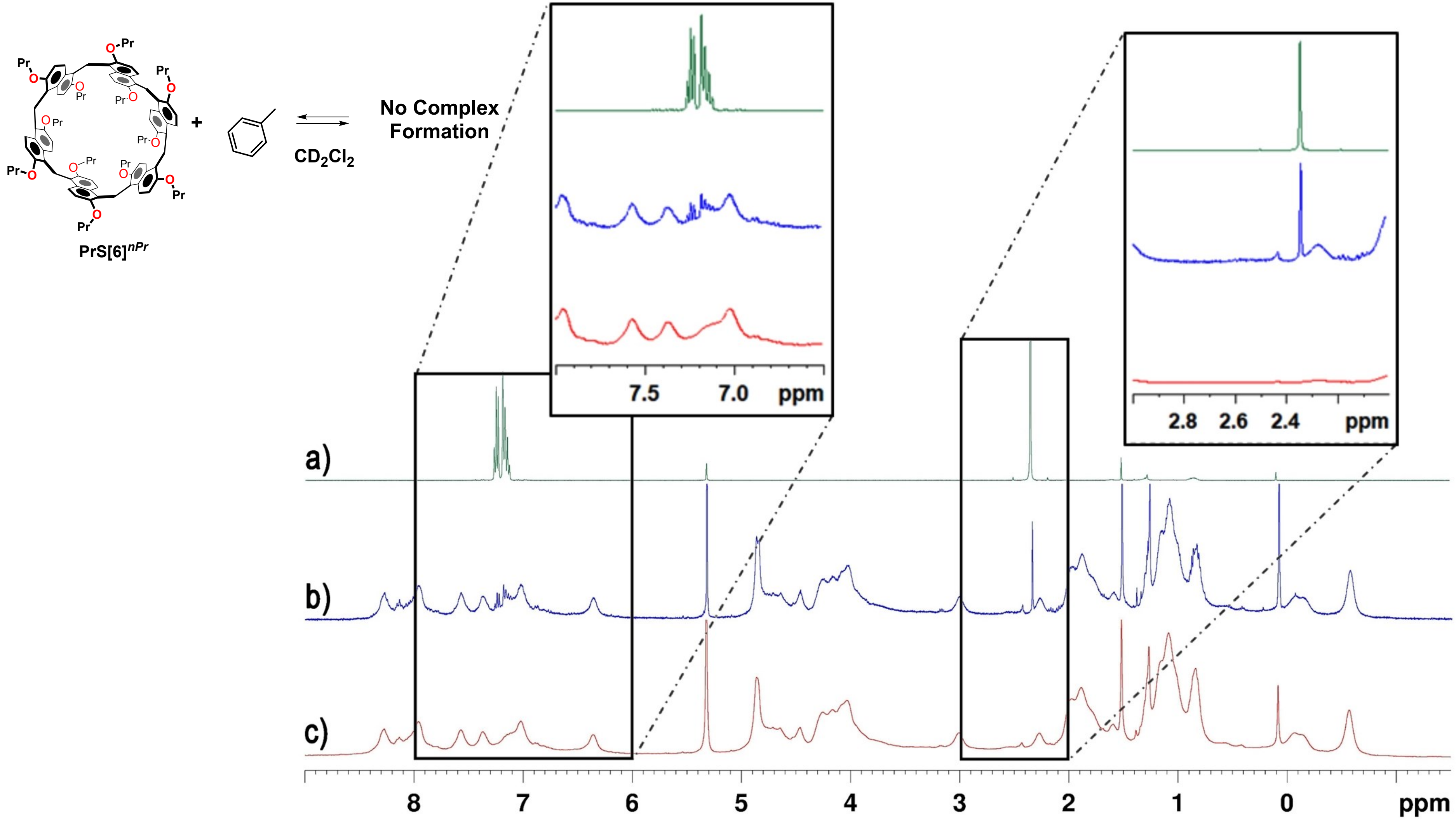

Figure S36: ^{1}H NMR spectra (400 MHz, CD_2Cl_2, 298 K) of: (a) toluene, (b) an equimolar solution of **PrS[6]*nPr*** and Toluene and (c) **PrS[6]*nPr***

Copies of 1D and 2D NMR spectra of prismarene complexes

5^{2+}@ PrS[5]*Et*

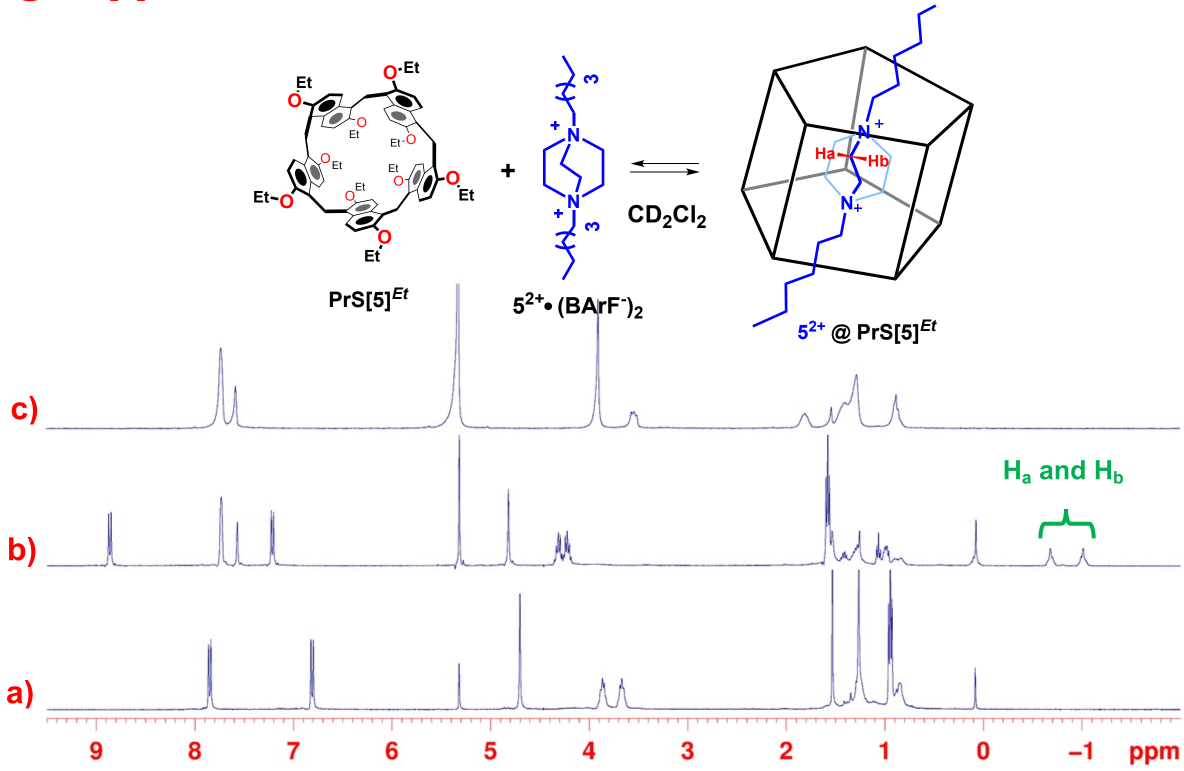

Figure S37: ^{1}H NMR spectra (600 MHz, CD$_2$Cl$_2$, 298 K) of: (a) **PrS[5]*Et***, (b) an equimolar solution of **PrS[5]*Et*** and **5^{2+}**·(BArF$^-$)$_2$ and (c) **5^{2+}**·(BArF$^-$)$_2$.

6^+@ PrS[5]*Et*

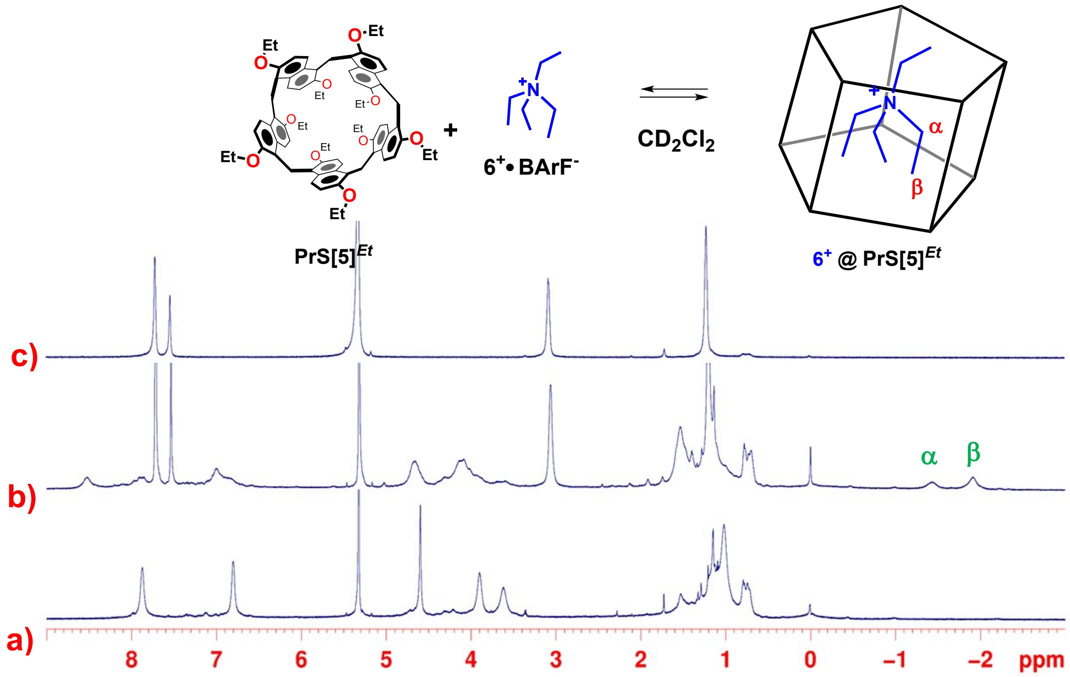

Figure S38: ^{1}H NMR spectra (600 MHz, CD$_2$Cl$_2$, 183 K) of: (a) **PrS[5]*Et***, (b) an equimolar solution of **PrS[5]*Et*** and **6^+**·(BArF$^-$) and (c) **6^+**·(BArF$^-$).

7^{2+}@ PrS[5]^{Et}

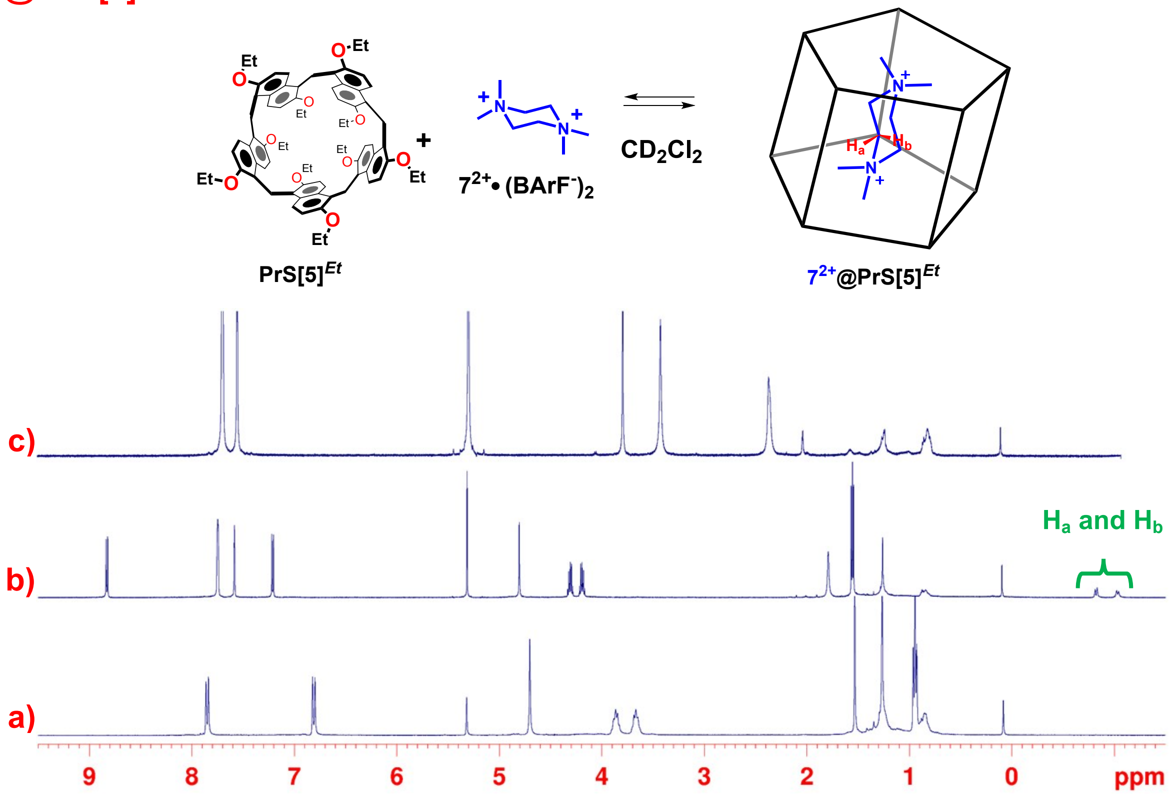

Figure S39: ^{1}H NMR spectra (600 MHz, CD_2Cl_2, 298 K) of: (a) **PrS[5]^{Et}**, (b) an equimolar solution of **PrS[5]^{Et}** and **7^{2+}**·$(BArF^{-})_2$ and (c) **7^{2+}**·$(BArF^{-})_2$.

8^{2+}@ PrS[5]^{Et}

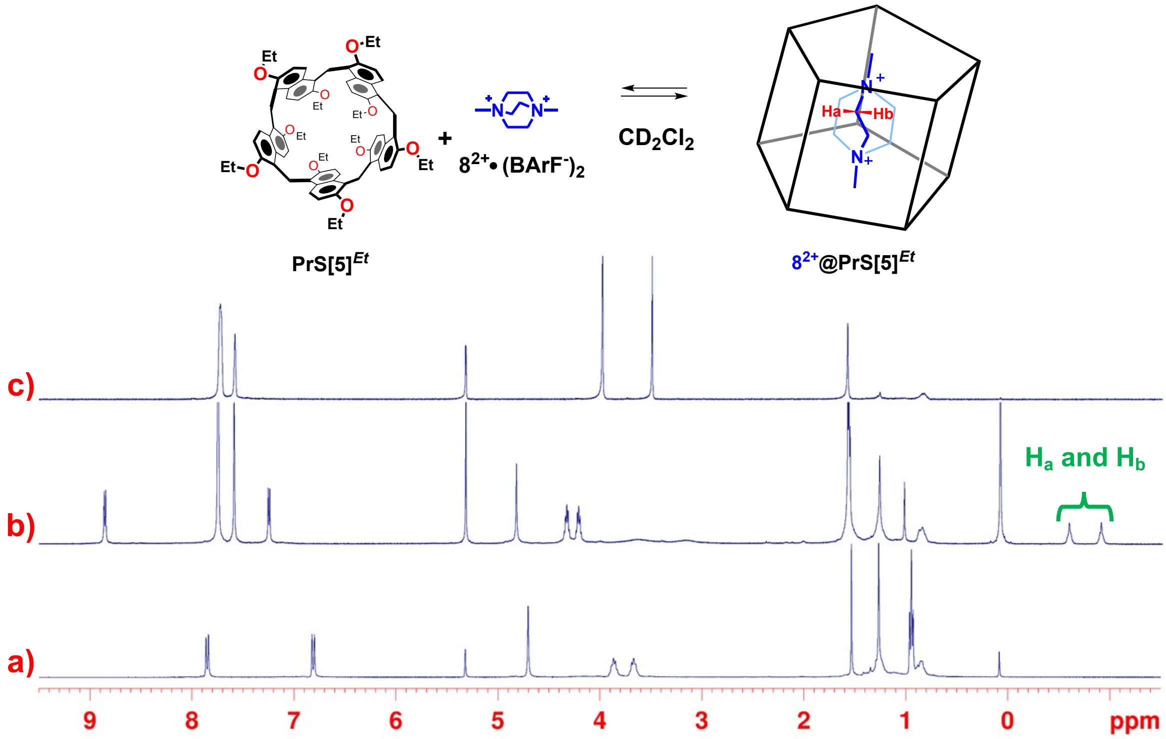

Figure S40: ^{1}H NMR spectra (600 MHz, CD_2Cl_2, 298 K) of: (a) **PrS[5]^{Et}**, (b) an equimolar solution of **PrS[5]^{Et}** and **8^{2+}**·$(BArF^{-})_2$ and (c) **8^{2+}**·$(BArF^{-})_2$.

9^{2+}@ PrS[5]^{Et}

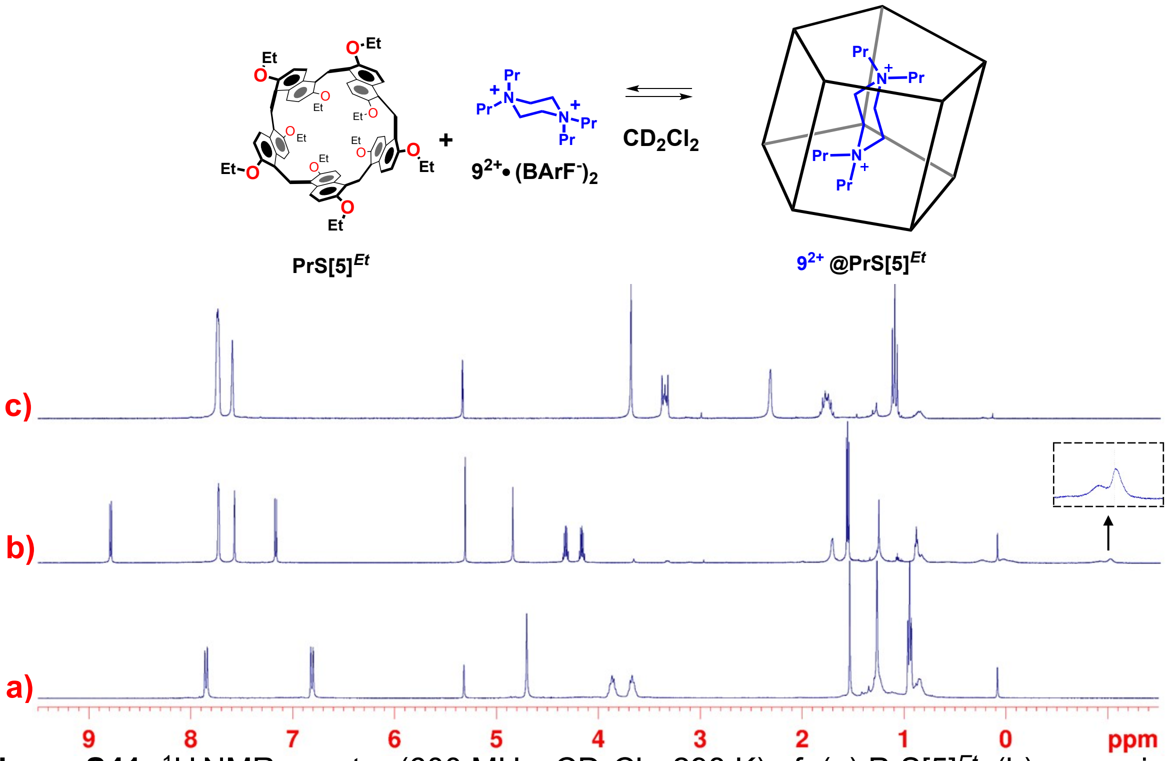

Figure S41: ^{1}H NMR spectra (600 MHz, CD_2Cl_2, 298 K) of: (a) PrS[5]^{Et}, (b) an equimolar solution of PrS[5]^{Et} and $\mathbf{9}^{2+}\cdot(BArF^-)_2$ and (c) $\mathbf{9}^{2+}\cdot(BArF^-)_2$.

5^{2+}@ PrS[6]^{Et}

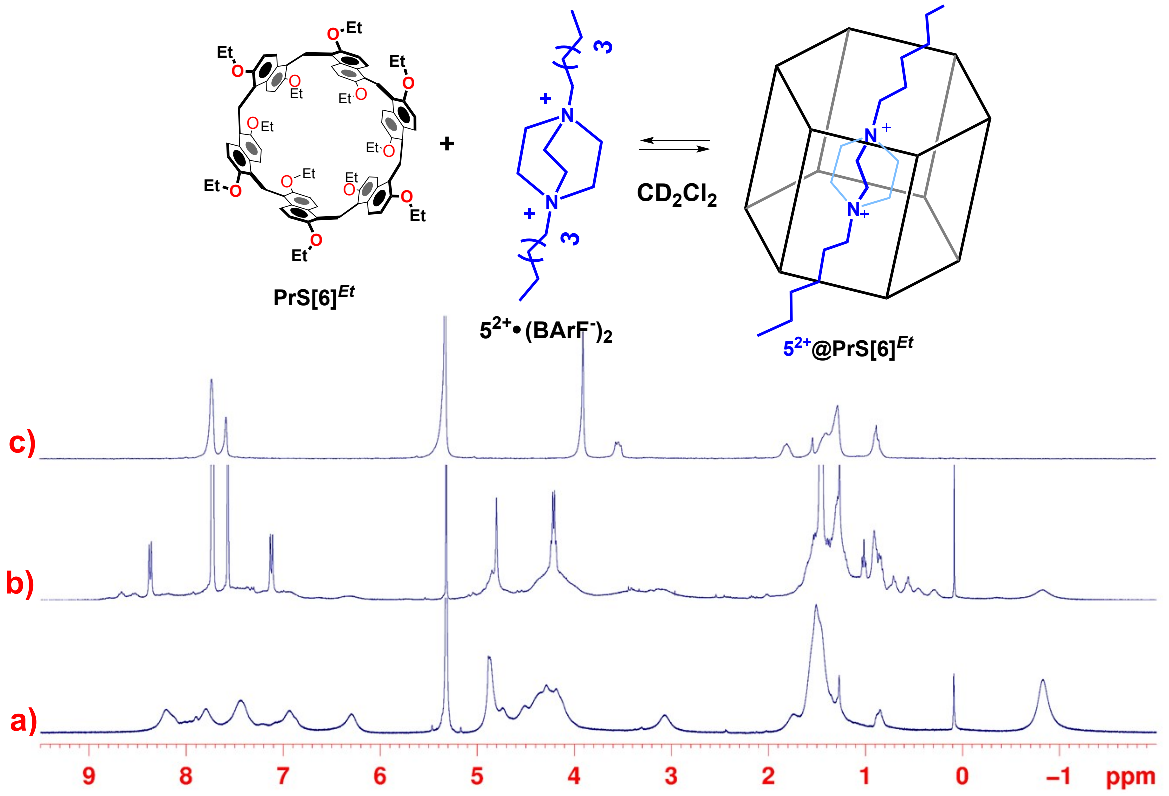

Figure S42: ^{1}H NMR spectra (600 MHz, CD_2Cl_2, 298 K) of: (a) PrS[6]^{Et}, (b) an equimolar solution of PrS[6]^{Et} and $\mathbf{5}^{2+}\cdot(BArF^-)_2$ and (c) $\mathbf{5}^{2+}\cdot(BArF^-)_2$.

6^+@ PrS[6]^{Et}

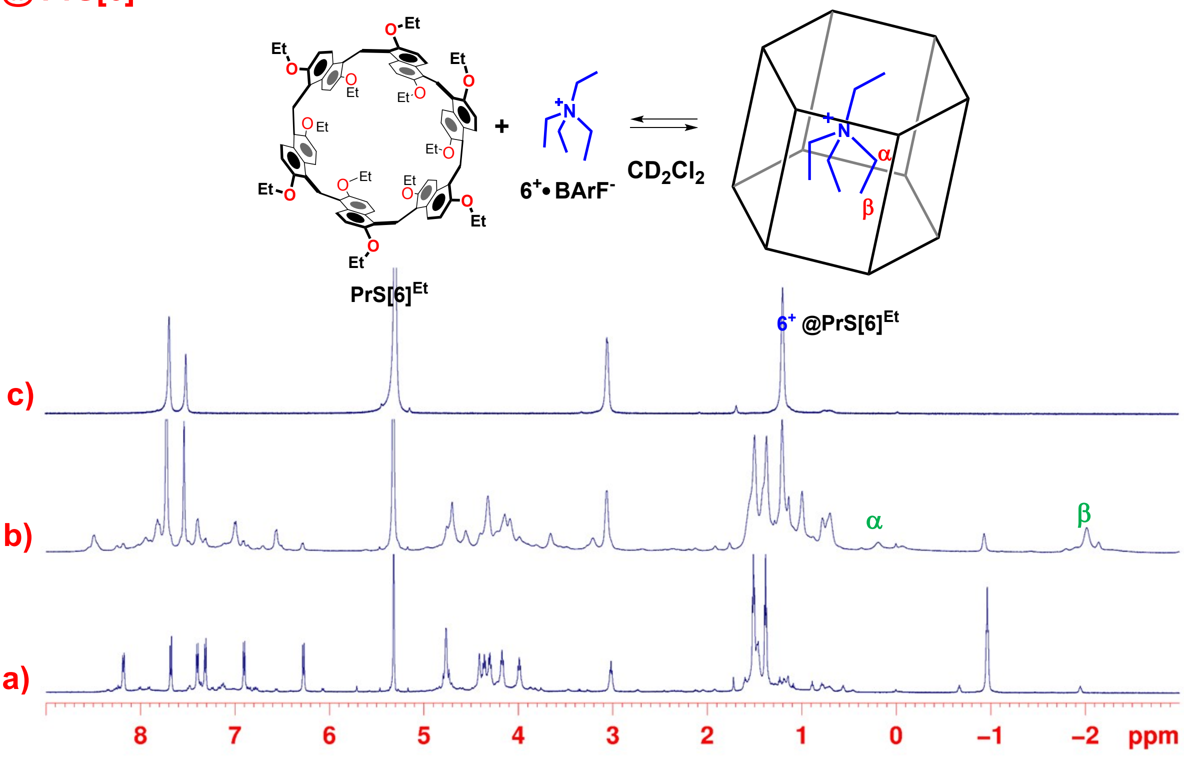

Figure S43: ^{1}H NMR spectra (600 MHz, CD_2Cl_2, 183 K) of: (a) **PrS[6]^{Et}**, (b) an equimolar solution of **PrS[6]^{Et}** and **6$^+$**·(BArF$^-$) and (c) **6$^+$**·(BArF$^-$).

7^{2+}@ PrS[6]^{Et}

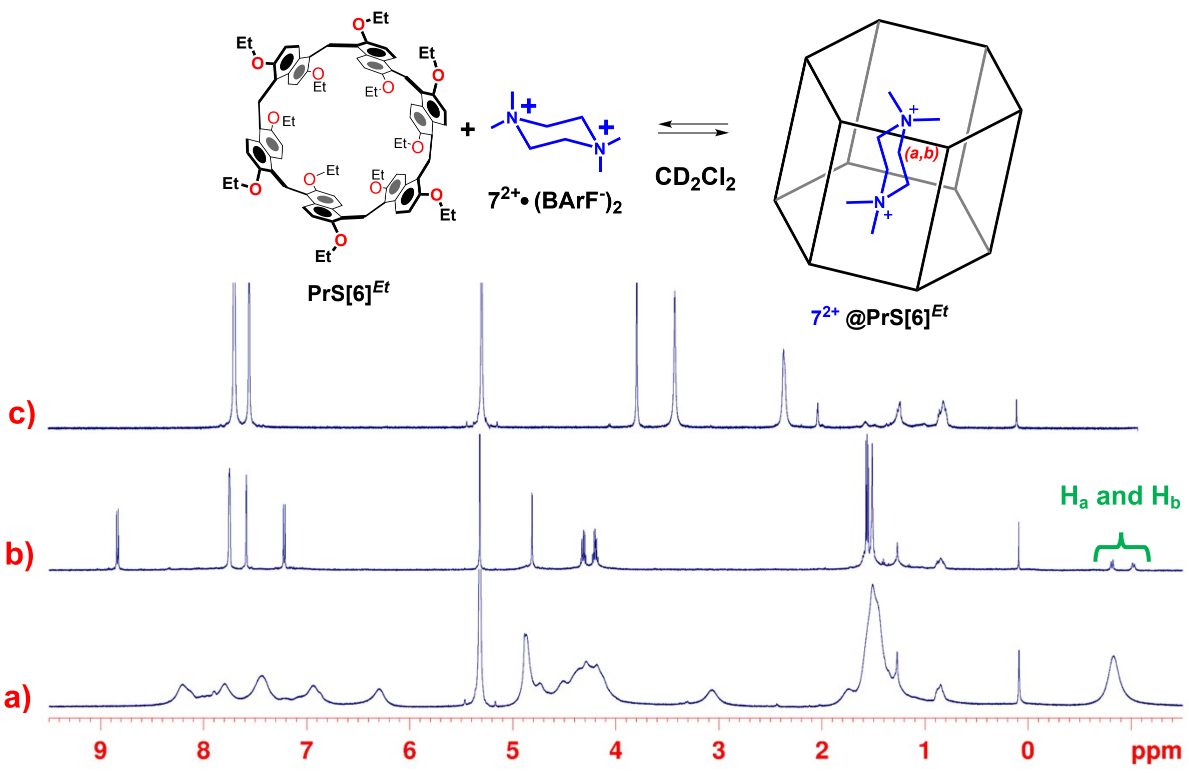

Figure S44: ^{1}H NMR spectra (600 MHz, CD_2Cl_2, 298 K) of: (a) **PrS[6]^{Et}**, (b) an equimolar solution of **PrS[6]^{Et}** and **7^{2+}**·(BArF$^-$)$_2$ and (c) **7^{2+}**·(BArF$^-$)$_2$.

5^{2+}@ PrS[5]^{nPr}

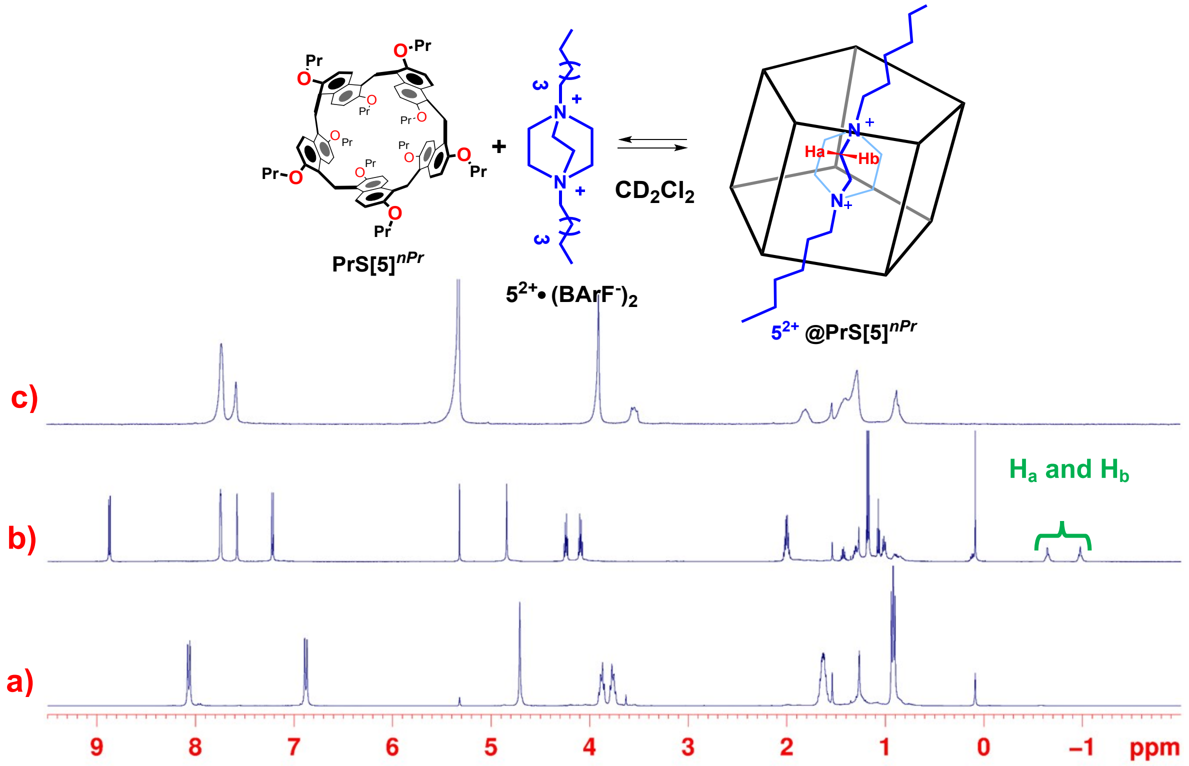

Figure S45: ^{1}H NMR spectra (600 MHz, CD_2Cl_2, 298 K) of: (a) **PrS[5]^{nPr}**, (b) an equimolar solution of **PrS[5]^{nPr}** and **5^{2+}**·(BArF$^-$)$_2$ and (c) **5^{2+}**·(BArF$^-$)$_2$.

7^{2+}@PrS[5]^{nPr}

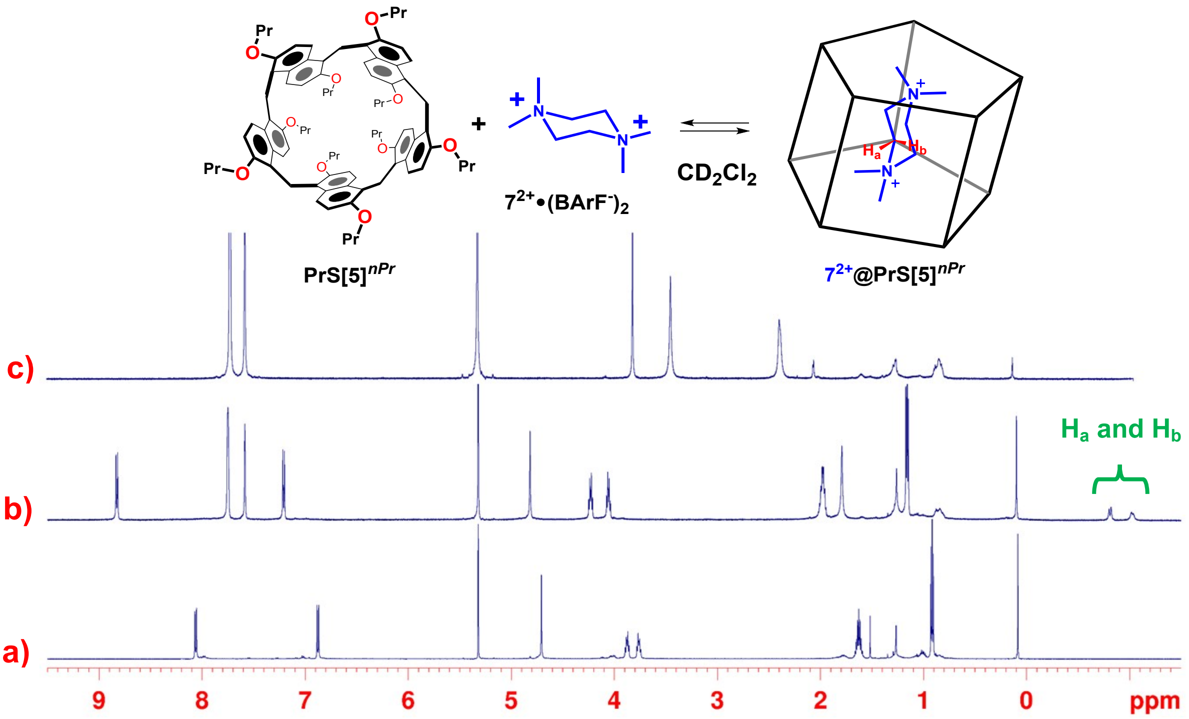

Figure S46: ^{1}H NMR spectra (600 MHz, CD_2Cl_2, 298 K) of: (a) PrS[5]^{nPr}, (b) an equimolar solution of PrS[5]^{nPr} and **7^{2+}**·(BArF$^-$)$_2$ and (c) **7^{2+}**·(BArF$^-$)$_2$.

8^{2+}@ PrS[5]^{nPr}

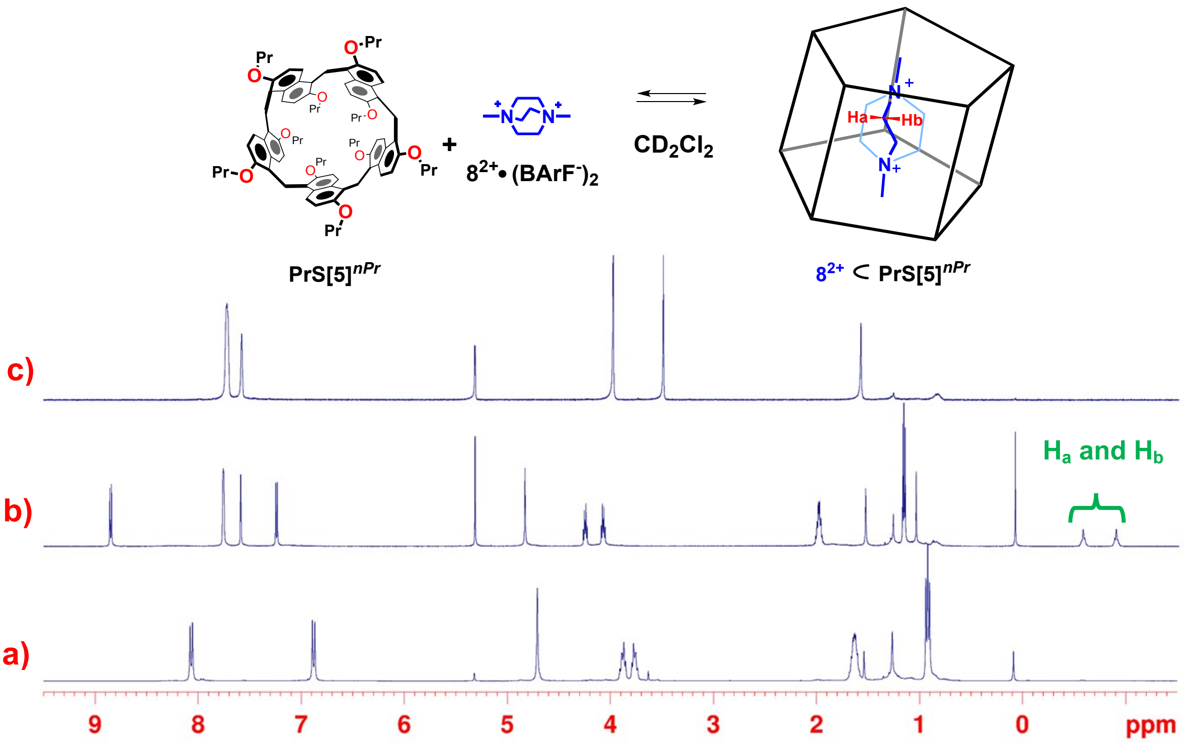

Figure S47: ^{1}H NMR spectra (600 MHz, CD_2Cl_2, 298 K) of: (a) **PrS[5]^{nPr}**, (b) an equimolar solution of **PrS[5]^{nPr}** and **8^{2+}**·$(BArF^-)_2$ and (c) **8^{2+}**·$(BArF^-)_2$.

9^{2+}@ PrS[5]^{nPr}

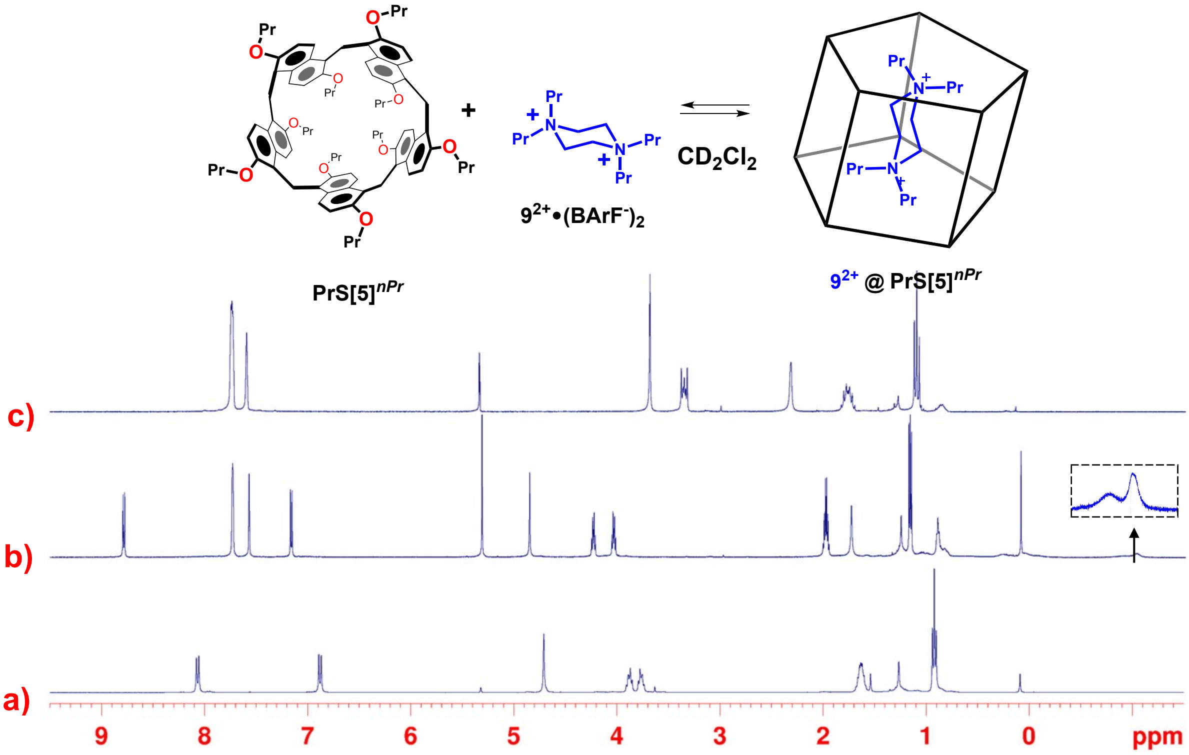

Figure 48. ^{1}H NMR spectra (600 MHz, CD_2Cl_2, 298 K) of: (a) **PrS[5]^{nPr}**, (b) an equimolar solution of **PrS[5]^{nPr}** and **9^{2+}**·$(BArF^-)_2$ and (c) **9^{2+}**·$(BArF^-)_2$.

6^+@ PrS[6]^{Pr}

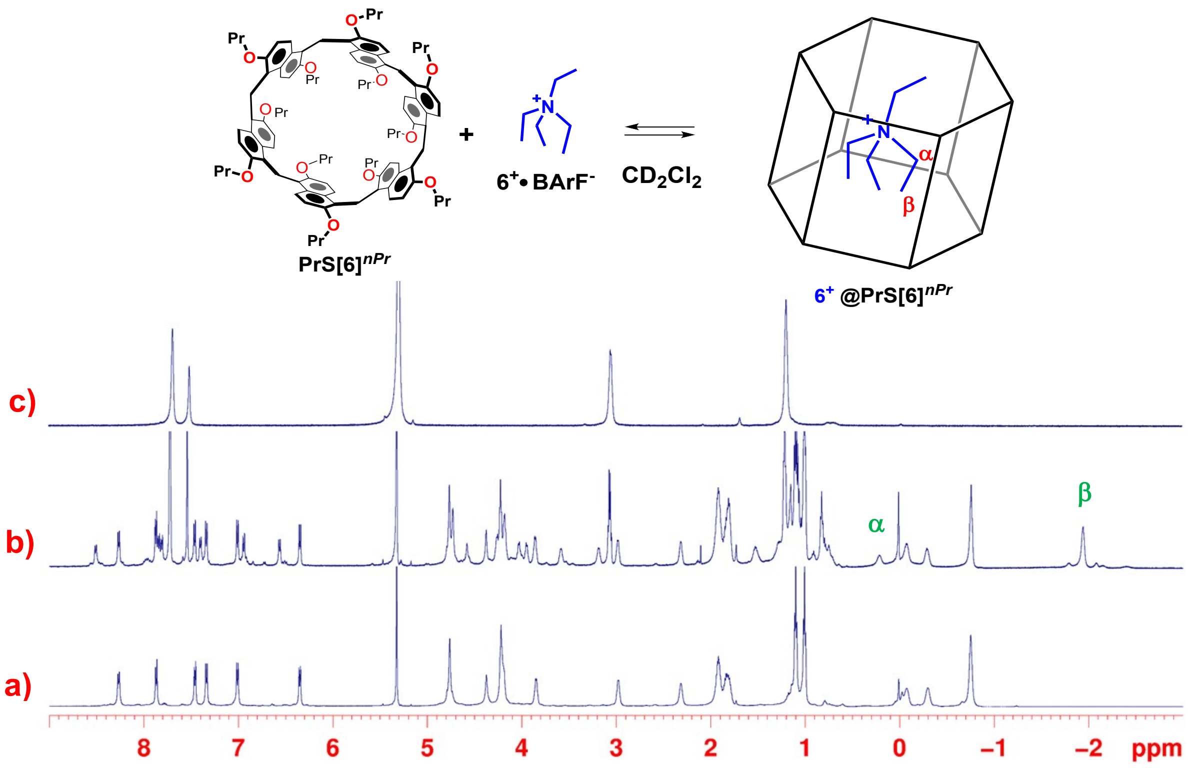

Figure S49: ^{1}H NMR spectra (600 MHz, CD_2Cl_2, 183 K) of: (a) **PrS[6]^{nPr}**, (b) an equimolar solution of **PrS[6]^{nPr}** and **6^+**·(BArF$^-$) and (c) **6^+**·(BArF$^-$).

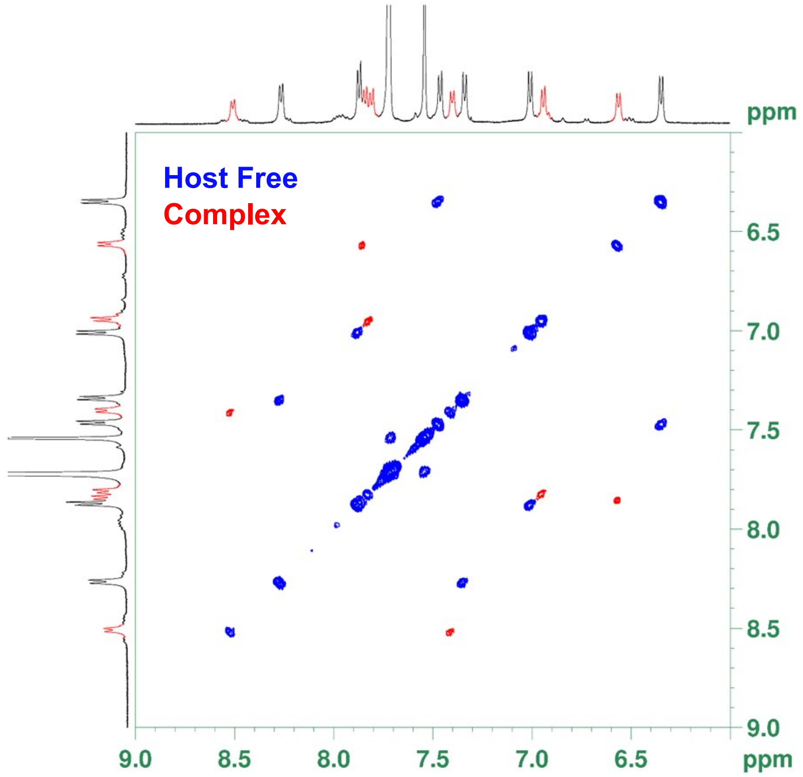

Figure S50: Significative portion of 2D-DQF COSY spectrum of **6^+@ PrS[6]^{nPr}** (CD_2Cl_2, 600 MHz, 183 K).

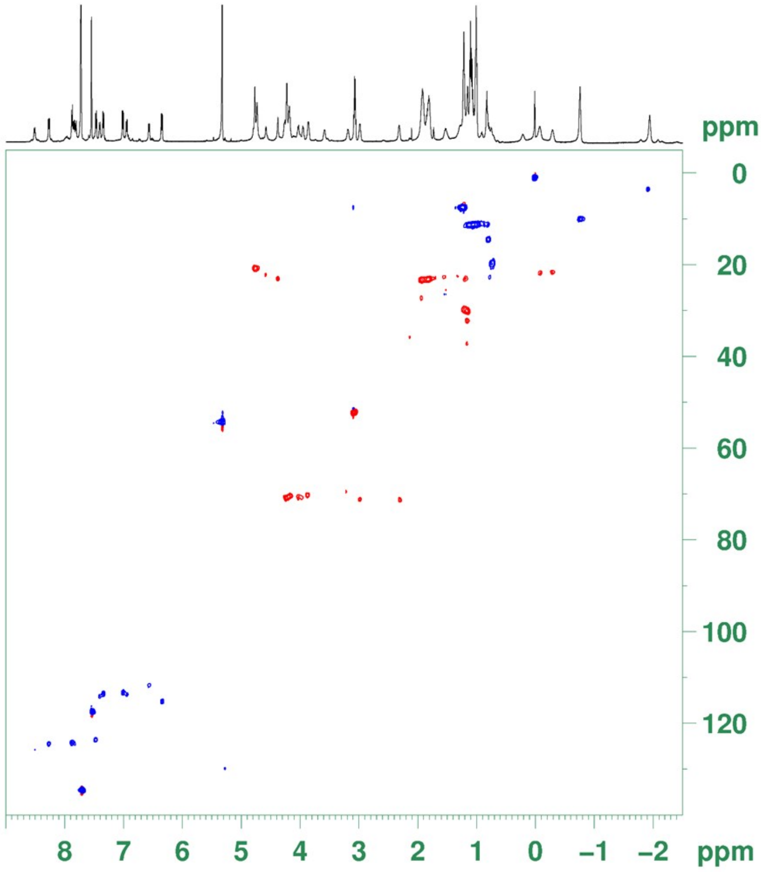

Figure S51: 2D-HSQC spectrum of **6^{+}@ PrS[6]^{nPr}** (CD_2Cl_2, 600 MHz, 183 K).

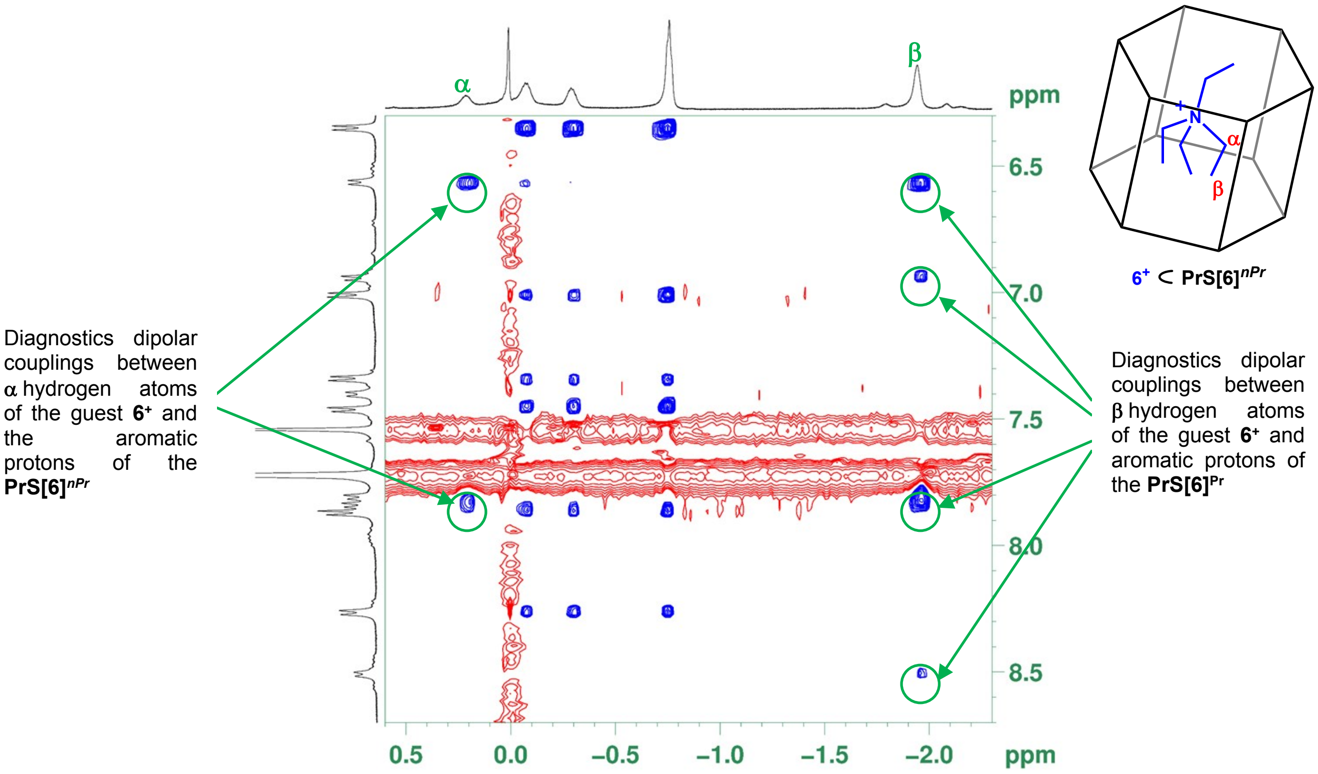

Figure S52: Significant portion of 2D-NOESY spectrum of **6^{+}@ PrS[6]^{nPr}** (CD_2Cl_2, 600 MHz, 183 K).

7^{2+}@ PrS[6]^{nPr}

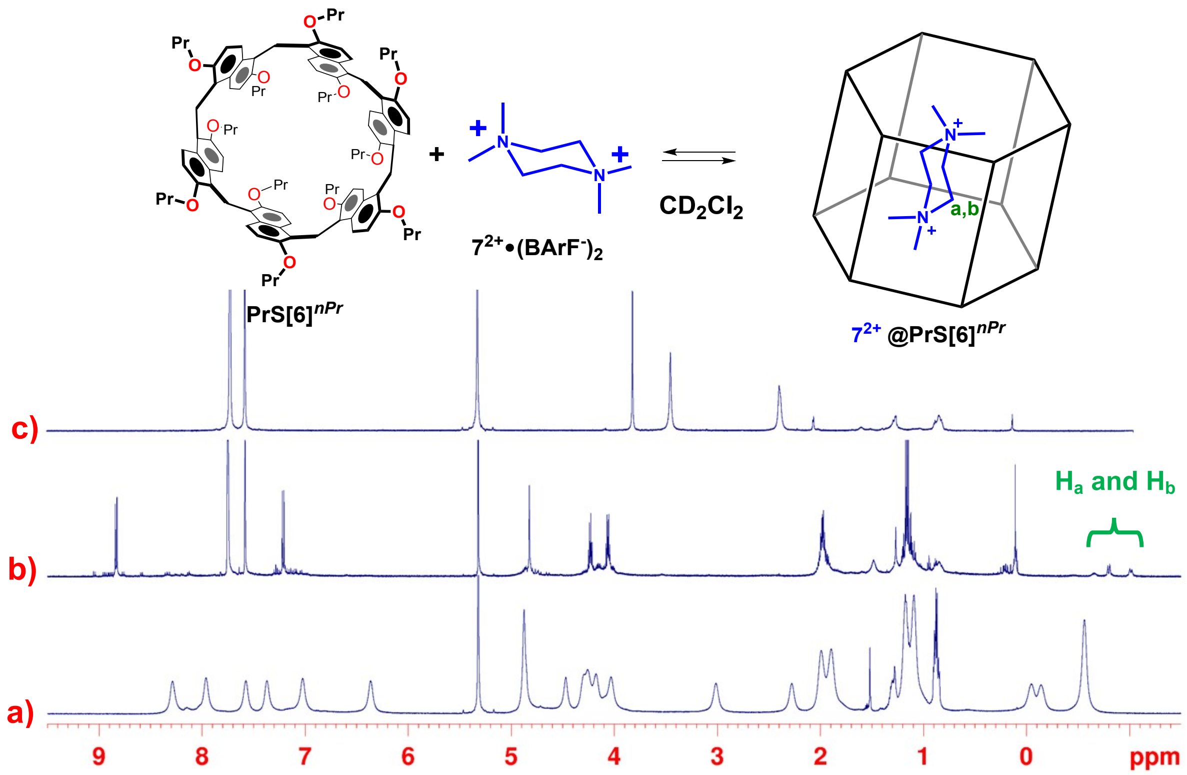

Figure S53: ^{1}H NMR spectra (600 MHz, CD_2Cl_2, 298 K) of: (a) **PrS[6]^{nPr}**, (b) an equimolar solution of **PrS[6]^{nPr}** and **7^{2+}**·(BArF$^-$)$_2$ and (c) **7^{2+}**·(BArF$^-$)$_2$.

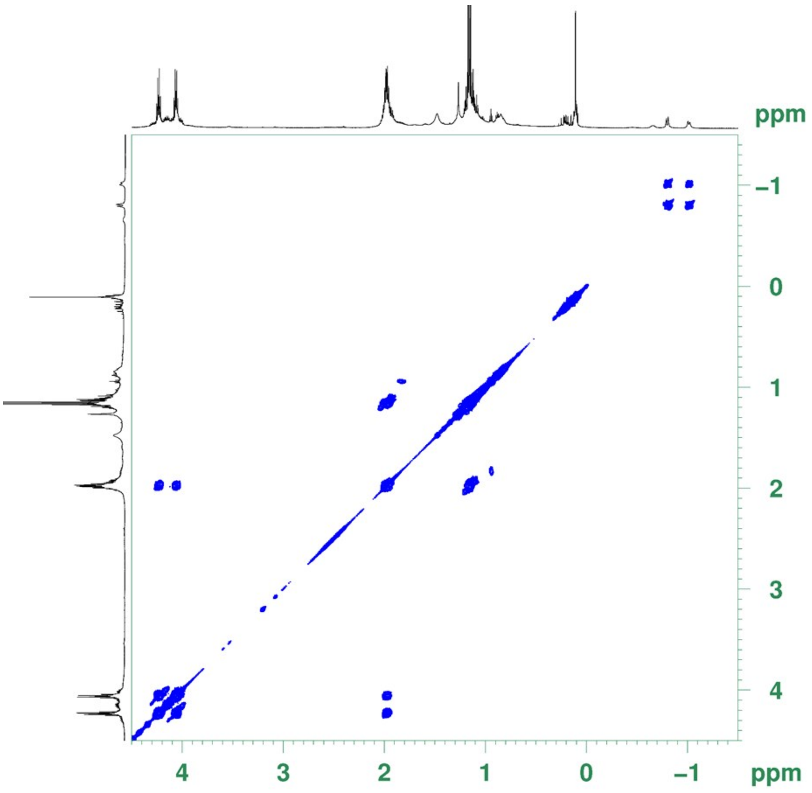

Figure S54: Significative portion of 2D-DQF COSY spectrum of **7^{2+}@ PrS[6]^{nPr}** (CD_2Cl_2, 600 MHz, 298 K).

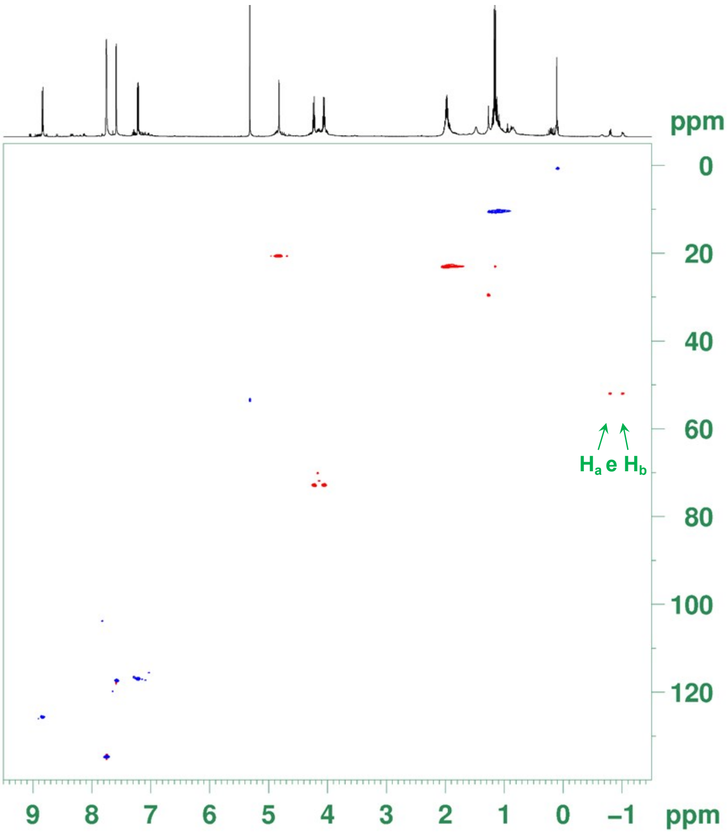

Figure S55: 2D-HSQC spectrum of **7^{2+}@ PrS[6]^{nPr}** (CD_2Cl_2, 600 MHz, 298 K).

^{1}H NMR determination of K_{ass} values.

The association constant values for the formation of the complexes were calculated by means of three methods[2]:

a) Integration of free and complexed ^{1}H NMR signals of host or guest. In this case, an equimolar solution of hosts and guests was solubilized in CD_2Cl_2 and equilibrated in NMR tube after mixing for 24-90 h at 40 °C.
b) ^{1}H NMR competition experiments. In this case, was performed an analysis of a 1:1:1 mixture of host, and two guests in an NMR tube and CD_2Cl_2 as solvent.
c) Quantitative ^{1}H NMR experiments using TCE as the internal standard[4]. In this case, ^{1}H NMR experiments were carried out on a 1:1 mixture of host and guest in CD_2Cl_2 containing 0.5 µL of 1,1,2,2-tetrachloroethane (d = 1.59 g/mL) as internal standard. *Further details are reported in the figure's captions*

Table S2. Association constant ($K_{ass,}$ M^{-1}) values for the formation of the complexes between the ammonium **5^{2+}** - **9^{2+}** cations as BArF^{-} salts and the prism[n]arenes. Determined by ^{1}H NMR experiments in CD_2Cl_2 . Errors < 15% calculated as mean values of three measures.

	**PrS[5]*Me***	**PrS[6]*Me***	**PrS[5]*Et***	**PrS[6]*Et***	**PrS[5]*nPr***	**PrS[6] *nPr***
**5^{2+}**	**3.9·10^{7} M^{-1} [1]**	50 M^{-1} [1]	**1.4·10^{8} M^{-1} [2]**	790 M^{-1}[5]	**1.7·10^{8} M^{-1} [3]**	400 M^{-1}[4]
**6^{+}**	90 M^{-1} [1]	2.7·10^{3} M^{-1} [1]	50 M^{-1}[6]	2745 M^{-1}[7]	100 M^{-1}[8]	340 M^{-1}[9]
**7^{2+}**	**1.8·10^{7} M^{-1} [1]**	----	**2.8·10^{8} M^{-1}[10]**	**1.0·10^{8} M^{-1}[11]**	**1.4·10^{9} M^{-1}[12]**	**1.2·10^{8} M^{-1}[13]**
**8^{2+}**	**6.6·10^{6} M^{-1}[14]**	**4.2·10^{3} M^{-1}[19]**	**2.9·10^{6} M^{-1}[15]**	**420 M^{-1}[21]**	**6.3·10^{7} M^{-1}[23]**	**370 M^{-1}[22]**
**9^{2+}**	**5780 M^{-1}[16]**	**3.7·10^{3} M^{-1}[18]**	**1.0·10^{5} M^{-1}[17]**	----	**4.8·10^{5} M^{-1}[20]**	----

[1] Reported in reference 2. **[2]** Calculated by competition experiment at 298 K with **PrS[5]*Me*** (see Figure S56 and its caption). **[3]** Calculated by competition experiment at 298 K with **PrS[5]*Me*** (see Figure S57 and its caption). [4]Calculated by integration at 298 K of ^{1}H NMR signals of free and complexed species (see Figure S58 and its caption).[5] Calculated by integration at 298 K of ^{1}H NMR signals of free and complexed species (see Figure S59 and its caption). [6]Calculated by quantitative ^{1}H NMR spectroscopy using TCE as internal standard.[7] Calculated by integration at 183 K of ^{1}H NMR signals of free and complexed species (see Figure S60 and its caption). [8]Calculated by quantitative ^{1}H NMR spectroscopy at 183 K. [9]Calculated by integration at 183 K of ^{1}H NMR signals of free and complexed species (see Figure S61 and its caption). [10] Calculated by competition experiment at 298 K with **PrS[5]*Me*** (see Figure S62 and its caption). [11] Calculated by competition experiment at 298 K with **PrS[5]*Me*** (see Figure S63 and its caption). [12] Calculated by competition experiment at 298 K with **PrS[5]*Me*** (see Figure S64 and its caption).[13] Calculated by competition experiment at 298 K with **PrS[6]*Et*** (see Figure S65 and its caption).[14] Calculated by competition experiment at 298 K with **5^{2+}** (see Figure S66 and its caption). [15]Calculated by competition experiment at 298 K with **PrS[5]*Me*** (see Figure S67 and its caption).[16] Calculated by integration at 298 K of ^{1}H NMR signals of free and complexed species (see Figure S68 and its caption).[17] Calculated by competition experiment at 298 K with **PrS[5]*Me*** (see Figure S69 and its caption).[18] Calculated by competition experiment at 298 K with **PrS[5]*Et*** (see Figure S70 and its caption).[19] Calculated by competition experiment at 298 K with **9^{2+}** (see Figure S71 and its caption).[20] Calculated by competition experiment at 298 K with **PrS[5]*Me*** (see Figure S72 and its caption).[21] Calculated by competition experiment at 298 K with **PrS[6]*Me*** (see Figure S74 and its caption).[22] Calculated by competition experiment at 298 K with **PrS[6]*Me*** (see Figure S75 and its caption).[23] Calculated by competition experiment at 298 K with **PrS[5]*Me*** (see Figure S73 and its caption).

2 P. Della Sala, R. Del Regno, C. Talotta, A. Capobianco, N. Hickey, S. Geremia, M. De Rosa, A. Spinella, A. Soriente, P. Neri, C. Gaeta, *J. Am. Chem. Soc.* **2020**, *142*, 1752–1756.

The competition experiments in red and orange are described at pag S54

The competition experiments in purple are described at pag S67

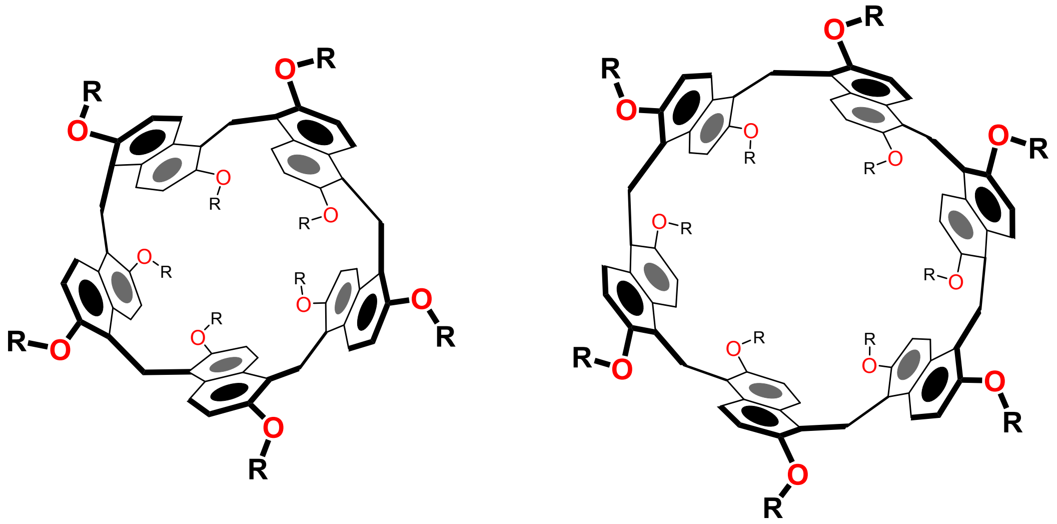

PrS[5]^{R} **PrS[6]^{R}**

R = Me R= Et R= *n*Pr

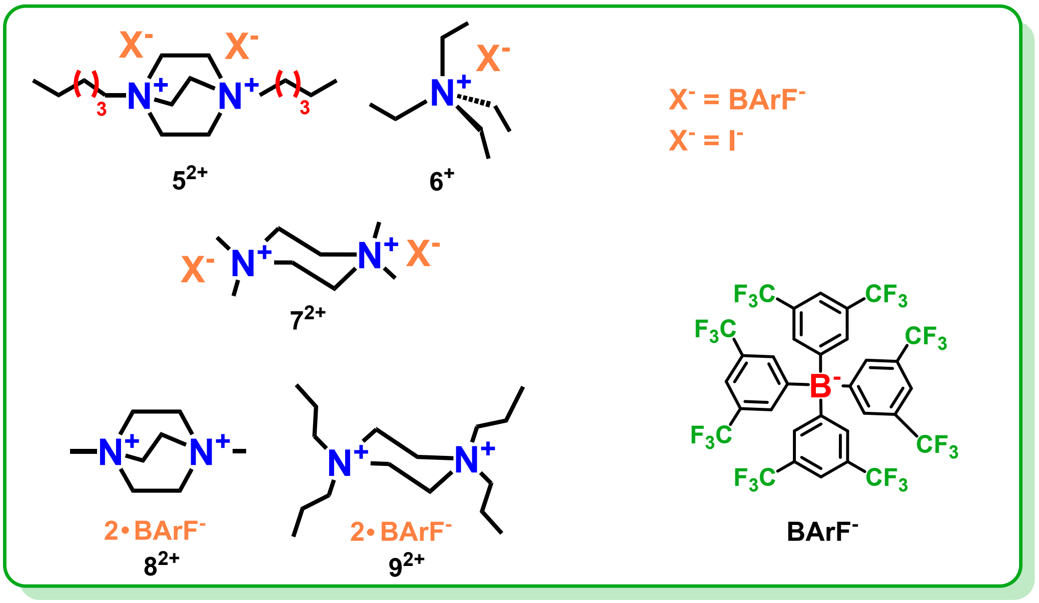

K (5^{2+}@PrS[5]^{Me}) = 3.9·10^{7} M^{-1} (by reference [2])

Competition experiment: **5^{2+}+ PrS[5]^{Me} + PrS[5]^{Et} = 5^{2+}@PrS[5]^{Me} + 5^{2+}@PrS[5]^{Et}**→K (**5^{2+}@PrS[5]^{Et}**) = 1.4·10^{8} M^{-1}(figure S56)

Competition experiment: **5^{2+}+ PrS[5]^{Me} + PrS[5]^{nPr} = 5^{2+}@PrS[5]^{Me} + 5^{2+}@PrS[5]^{nPr}**→K (**5^{2+}@PrS[5] ^{nPr}**) = 1.7·10^{8} M^{-1}(figure S57)

Competition experiment: **5^{2+}+ PrS[5]^{Me} + 8^{2+} = 5^{2+}@PrS[5]^{Me} + 8^{2+}@PrS[5]^{Me}**→K (**8^{2+}@PrS[5]^{Me}**) = 6.6·10^{6} M^{-1}(figure S66)

Competition experiment: **8^{2+}+ PrS[5]^{Me} + PrS[5]^{Et} = 8^{2+}@PrS[5]^{Me} + 8^{2+}@PrS[5]^{Et}**→K (**8^{2+}@PrS[5]^{Et}**) = 2.9·10^{6} M^{-1}(figure S67)

K (**7^{2+}@PrS[5]^{Me}**) = 1.8·10^{7} M^{-1} (by reference [2])

Competition experiment: **7^{2+}+ PrS[5]^{Me} + PrS[5]^{Et} = 7^{2+}@PrS[5]^{Me} + 7^{2+}@PrS[5]^{Et}**→K (**7^{2+}@PrS[5]^{Et}**) = 2.8·10^{8} M^{-1}(figure S62)

Competition experiment: **7^{2+}+ PrS[5]^{Me} + PrS[6]^{Et} = 7^{2+}@PrS[5]^{Me} +7^{2+}@PrS[6]^{Et}** →K **(7^{2+}@PrS[6]^{Et})** = 1.0·10^{8} M^{-1}(figure S63)

Competition experiment: **7^{2+}+ PrS[5]^{Me} + PrS[5]^{nPr} = 7^{2+}@PrS[5]^{Me} + 7^{2+}@PrS[5]^{nPr}** →K (**7^{2+}@PrS[5]^{nPr}**) = 1.4·10^{9} M^{-1}(figure S64)

Competition experiment: **7^{2+}+ PrS[6]^{nPr} + PrS[6]^{Et} = 7^{2+}@PrS[6]^{Et} +7^{2+}@PrS[6]^{nPr}**→K (**7^{2+}@PrS[6]^{nPr}**) = 1.2·10^{8} M^{-1}(figure S65)

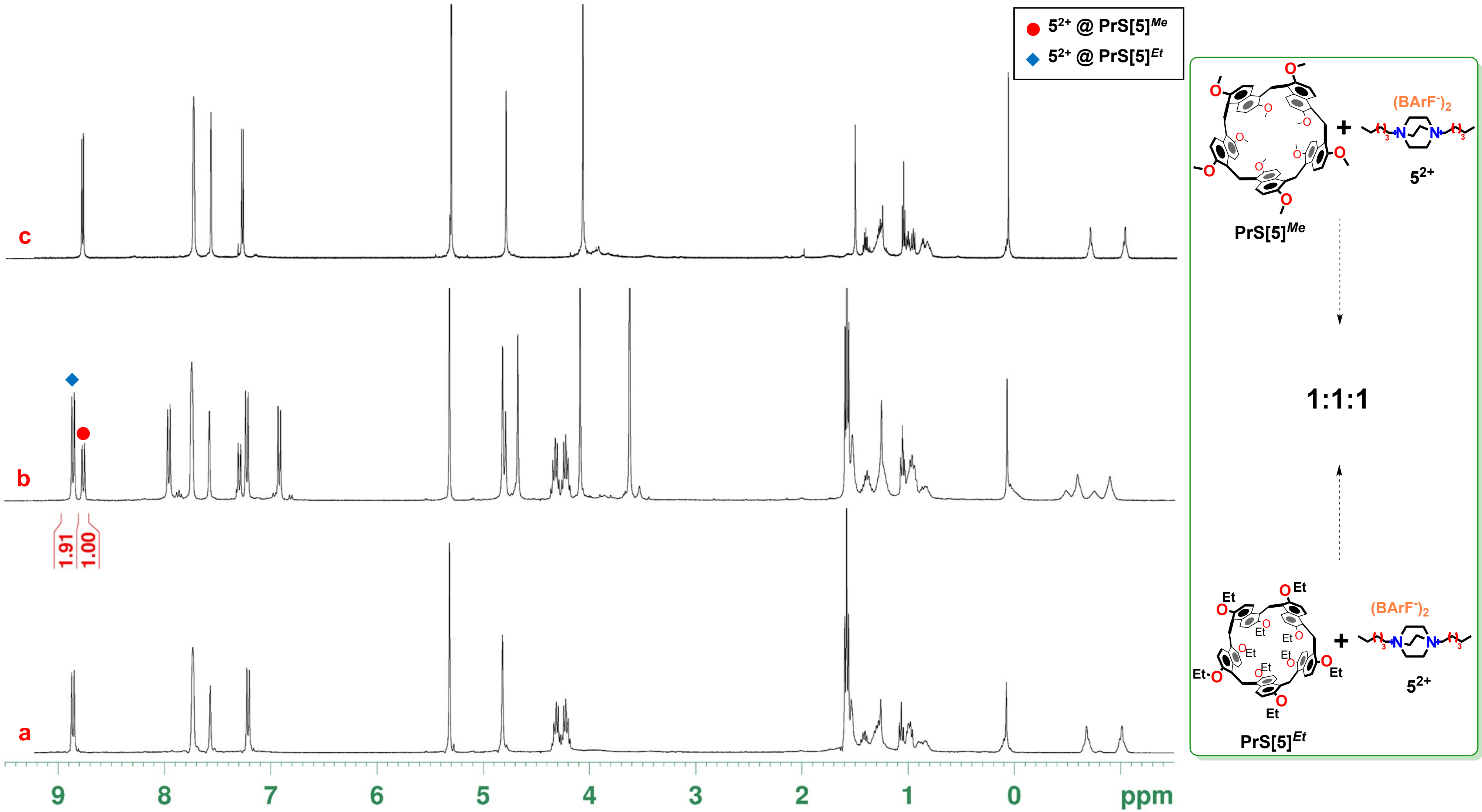

Figure S56: ^{1}H NMR spectra (400 MHz, CD_2Cl_2, 298 K) of: (a) an equimolar solution (2.50 mM) of **PrS[5]^{Et}** and **5**$(BArF)_2$ in 0.7 mL of CD_2Cl_2, (b) of **PrS[5]^{Me}** in the presence of 1 equivalent of **PrS[5]^{Et}** and 1 equivalent of **5**$(BArF)_2$ and (c) an equimolar solution (2.50 mM) of **PrS[5]^{Me}** and **5**$(BArF)_2$ in 0.7 mL of CD_2Cl_2.

Method B: $K_{rel} = K\,(\mathbf{5^{2+}@PrS[5]^{\mathit{Et}}}) / K\,(\mathbf{5^{2+}@PrS[5]^{\mathit{Me}}}) = K\,(\mathbf{5^{2+}@PrS[5]^{\mathit{Et}}}) / 3.9\text{x}10^{7} = [(1.91/2.91)\text{x}0.0025]^2 / [(1.00/2.91)\text{x}0.0025]^2$

$K\,(\mathbf{5^{2+}@PrS[5]^{\mathit{Et}}}) = 3.65 \text{ x } 3.9{\cdot}10^{7} = \mathbf{1.4{\cdot}10^{8}}\ M^{-1}$

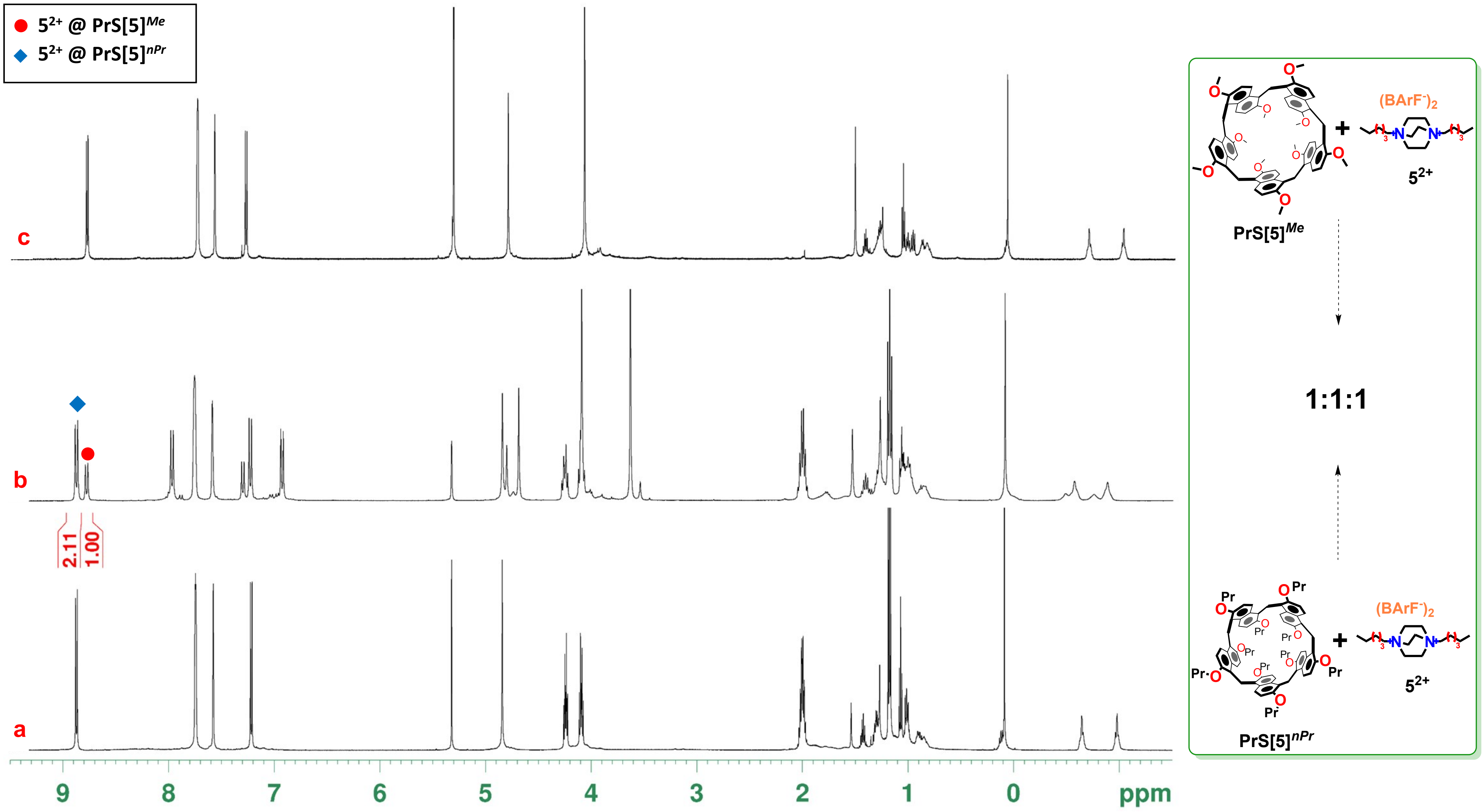

Figure S57: ^{1}H NMR spectra (400 MHz, CD_2Cl_2, 298 K) of: (a) an equimolar solution (2.23 mM) of **PrS[5]*^{nPr}*** and **5**(BArF)$_2$ in 0.7 mL of CD_2Cl_2, (b) of **PrS[5]*^{Me}*** in the presence of 1 equivalent of **PrS[5]*^{nPr}*** and 1 equivalent of **5**(BArF)$_2$ and (c) an equimolar solution (2.23 mM) of **PrS[5]*^{Me}*** and **5**(BArF)$_2$ in 0.7 mL of CD_2Cl_2.

Method B: $K_{rel} = K$ (**5^{2+}@PrS[5]^{nPr}**) / K (**5^{2+}@PrS[5]^{Me}**) = K (**5^{2+}@PrS[5]^{nPr}**) / 3.9x10^7 = [(2.11/3.11)x0.0022]^2 / [(1.00/3.11)x0.0022]^2

K (**5^{2+}@PrS[5]^{nPr}**) = 4.46 x 3.9·10^7= **1.7·10^8** M^{-1}

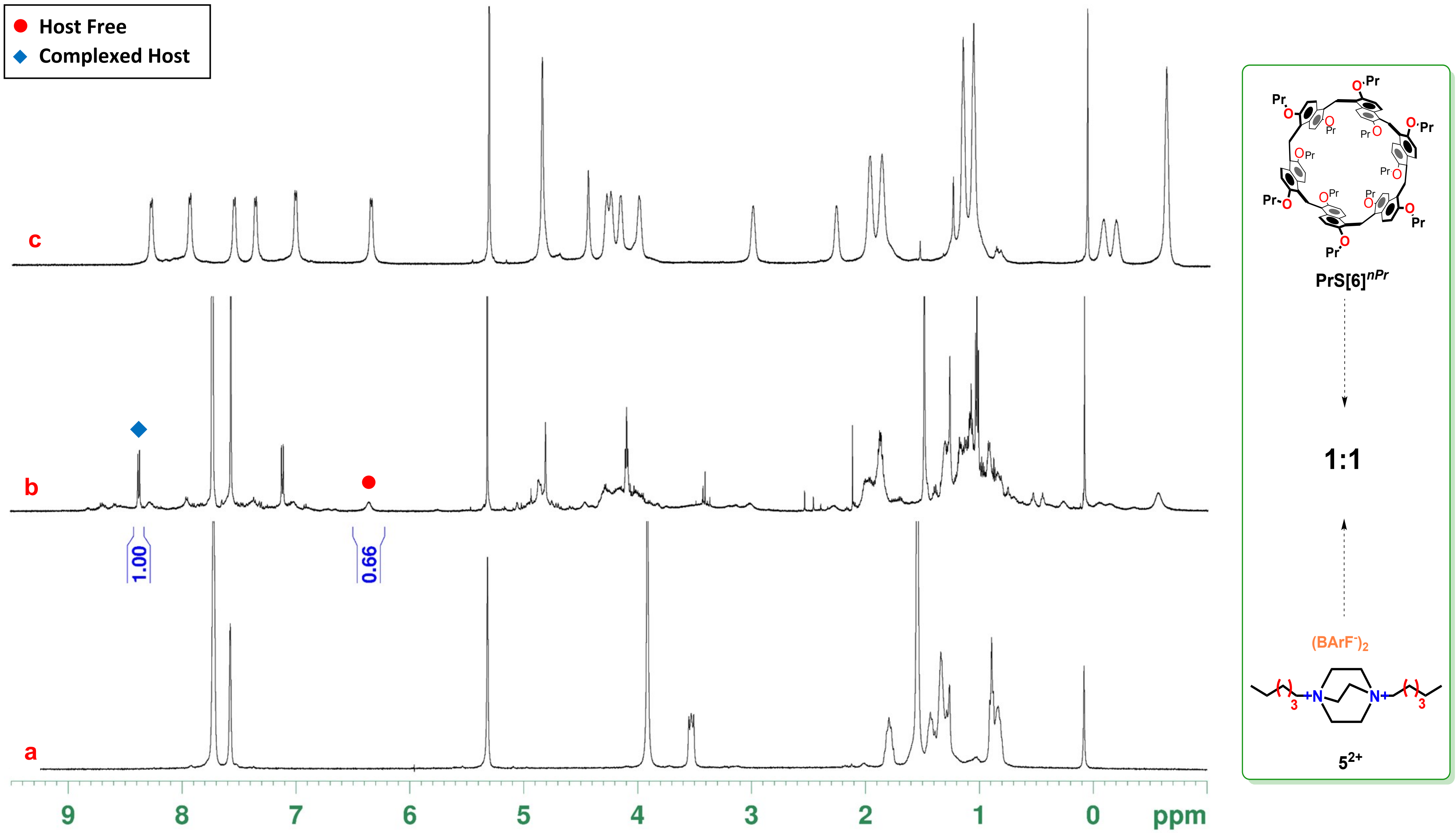

Figure S58: ^{1}H NMR spectra (600 MHz, CD_2Cl_2, 298 K) of: (a) a solution of **5**(BArF)$_2$ (b) an equimolar solution (2.00 mM) of **PrS[6]**^{nPr} and **5**(BArF)$_2$ in 0.7 mL of CD_2Cl_2 and (c) a solution of **PrS[6]**^{nPr}.

Method A: Integral normalization: 1/12 = 0.08 and 0.66/4 = 0.16. [**5**$^{2+}$@**PrS[6]**^{nPr}] **=** = [(0.08/0.24) x 0.002] = 6.7·10^{-4}; [**PrS[6]**^{nPr}]$_{free}$= [**5**$^{2+}$]$_{free}$ = 0.0013: *K* (**5**$^{2+}$@**PrS[6]**^{nPr}) = 6.7·10^{-4}/1.7·10^{-6}= **400** M^{-1}

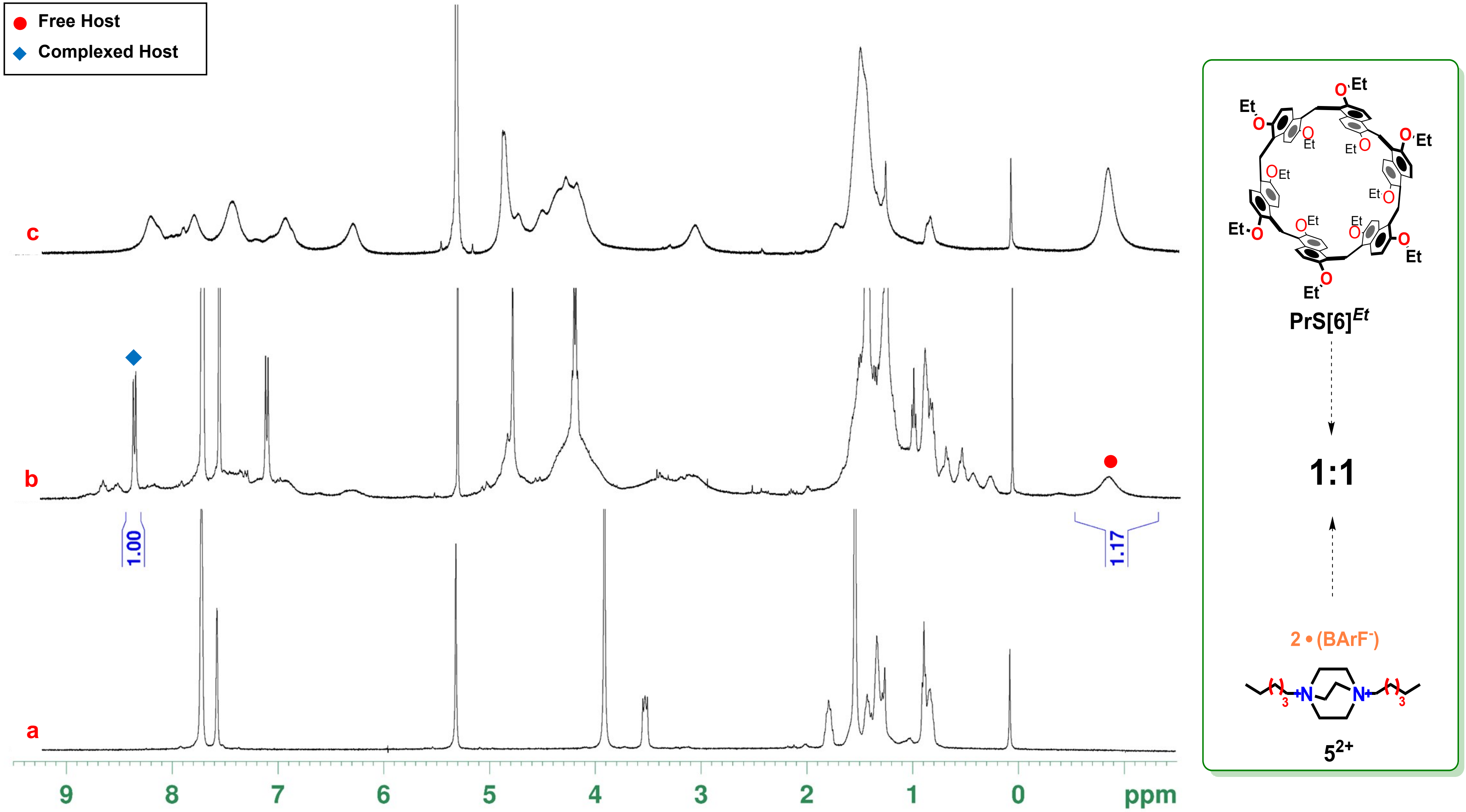

Figure S59: ^{1}H NMR spectra (600 MHz, CD_2Cl_2, 298 K) of: (a) a solution of **PrS[6]**^{Et} (b) an equimolar solution (2.00 mM) of **PrS[6]**^{Et} and **5**$(BArF)_2$ in 0.7 mL of CD_2Cl_2 and (c) a solution of **5**$(BArF)_2$.

Method A: [5^{2+}@**PrS[6]**^{Et}] **=** = [(1.00/2.17) x 0.002] = $9.21 \cdot 10^{-4}$; [**PrS[6]**^{Et}]$_{free}$= [5^{2+}]$_{free}$ = 0.0011: *K* (5^{2+}@**PrS[6]**^{Et}) =

$9.21 \cdot 10^{-4} / 1.16 \cdot 10^{-6}$= **790** M^{-1}

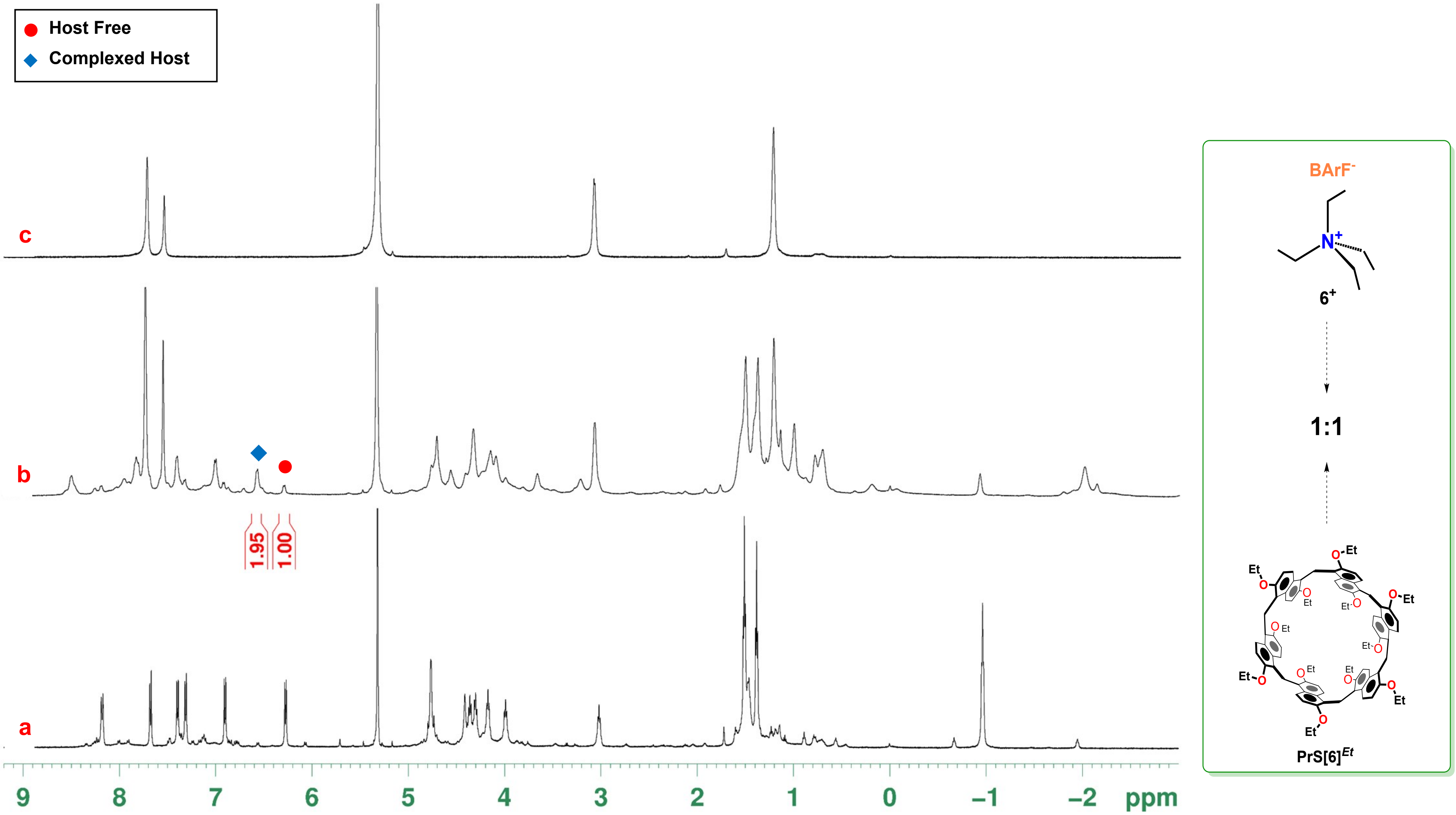

Figure S60: ^{1}H NMR spectra (600 MHz, CD_2Cl_2, 183 K) of: (a) a solution of **PrS[6]**^{Et} (b) an equimolar solution (2.1 mM) of **PrS[6]**^{Et} and **6**(BArF) in 0.7 mL of CD_2Cl_2 and (c) a solution of **6**(BArF).

Method A: [6^+@**PrS[6]**^{Et}] **=** = [(1.95/2.95) x 0.0021] = 0.0014; [**PrS[6]**^{Et}]$_{free}$= [6^+]$_{free}$ = 7.1·10^{-4}: K (6^+@**PrS[6]**^{Et}) = 0.0014/5.1·10^{-7}= **2745** M^{-1}

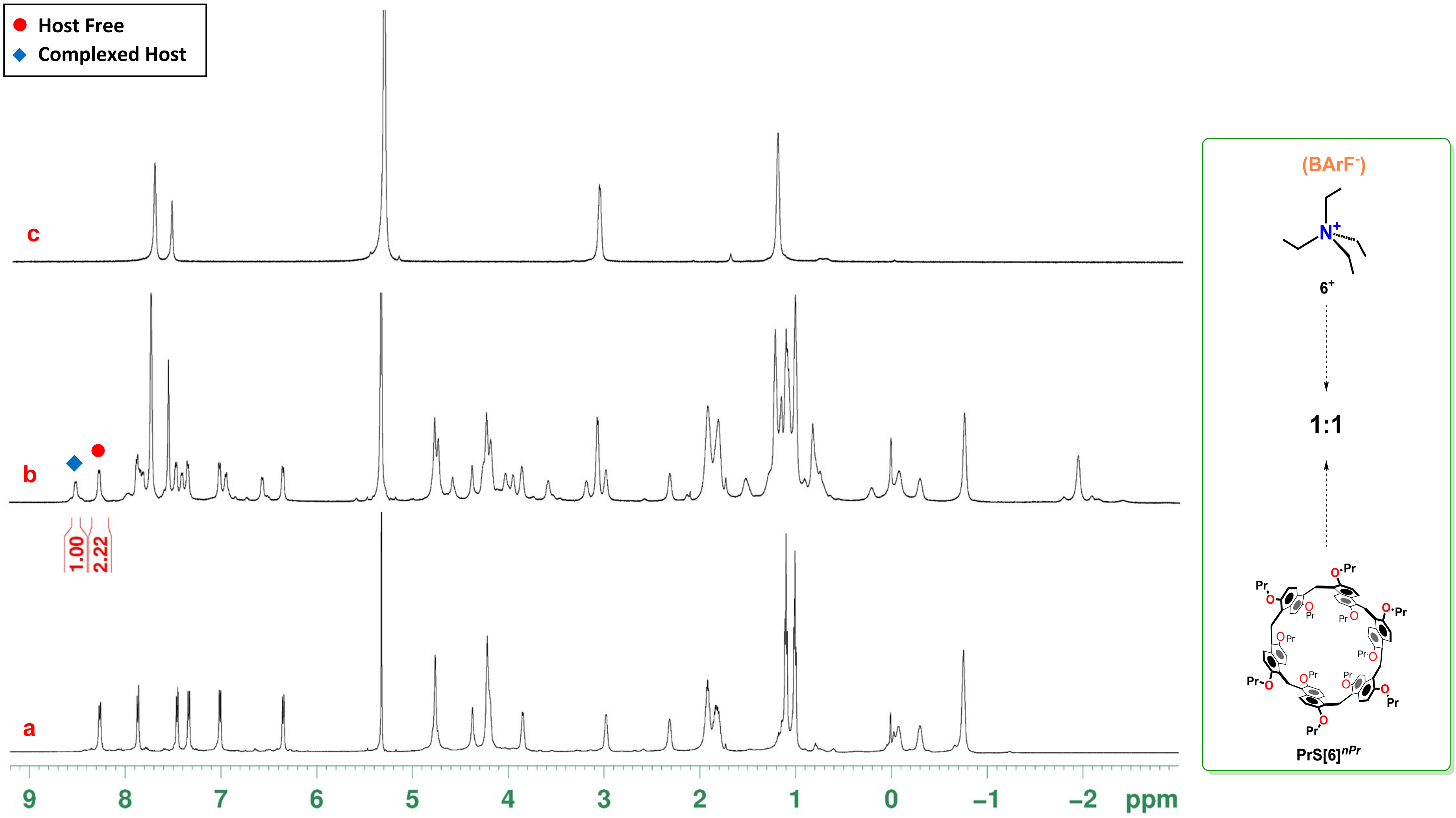

Figure S61: ^{1}H NMR spectra (600 MHz, CD_2Cl_2, 183 K) of: (a) a solution of **PrS[6]**^{nPr} (b) an equimolar solution (1.86 mM) of **PrS[6]**^{nPr} and **6**(BArF) in 0.7 mL of CD_2Cl_2 and (c) a solution of **6**(BArF).

Method A: [**6**$^+$**@PrS[6]**^{nPr}] **=** = [(1.00/3.22) x 0.00186] = $5.8\cdot10^{-4}$; [**PrS[6]**^{nPr}]$_{free}$= [**6**$^+$]$_{free}$ = 0.0013: *K* (**6**$^+$**@PrS[6]**^{Et}) = $5.8\cdot10^{-4}/1.7\cdot10^{-6}$= **340** M^{-1}

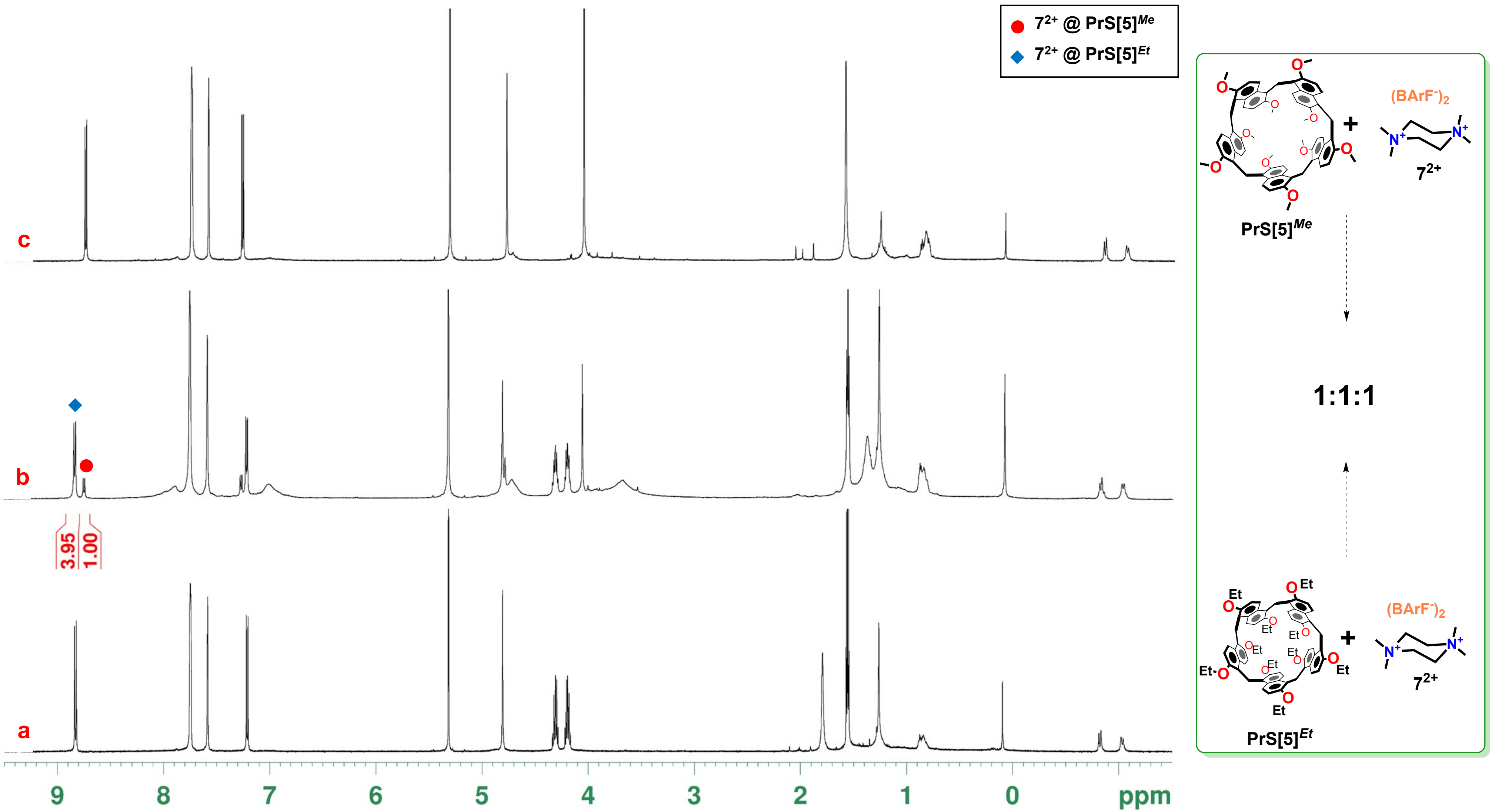

Figure S62: ^{1}H NMR spectra (600 MHz, CD_2Cl_2, 298 K) of: (a) an equimolar solution (2.50 mM) of **PrS[5]**^{Et} and **7**$(BArF)_2$ in 0.7 mL of CD_2Cl_2, (b) of **PrS[5]**^{Me} in the presence of 1 equivalent of **PrS[5]**^{Et} and 1 equivalent of **7**$(BArF)_2$ and (c) an equimolar solution (2.50 mM) of **PrS[5]**^{Me} and **7**$(BArF)_2$ in 0.7 mL of CD_2Cl_2.

Method B: $K_{rel} = K\,(\mathbf{7}^{2+}@\mathbf{PrS[5]}^{Et}) / K\,(\mathbf{7}^{2+}@\mathbf{PrS[5]}^{Me}) = K\,(\mathbf{7}^{2+}@\mathbf{PrS[5]}^{Et}) / 1.8\text{x}10^{7} = [(3.95/4.95)\text{x}0.0025]^2 / [(1.00/4.95)\text{x}0.0025]^2$

$K\,(\mathbf{7}^{2+}@\mathbf{PrS[5]}^{Et}) = 16 \times 1.8\cdot10^{7} = \mathbf{2.8\cdot10^{8}}\ M^{-1}$

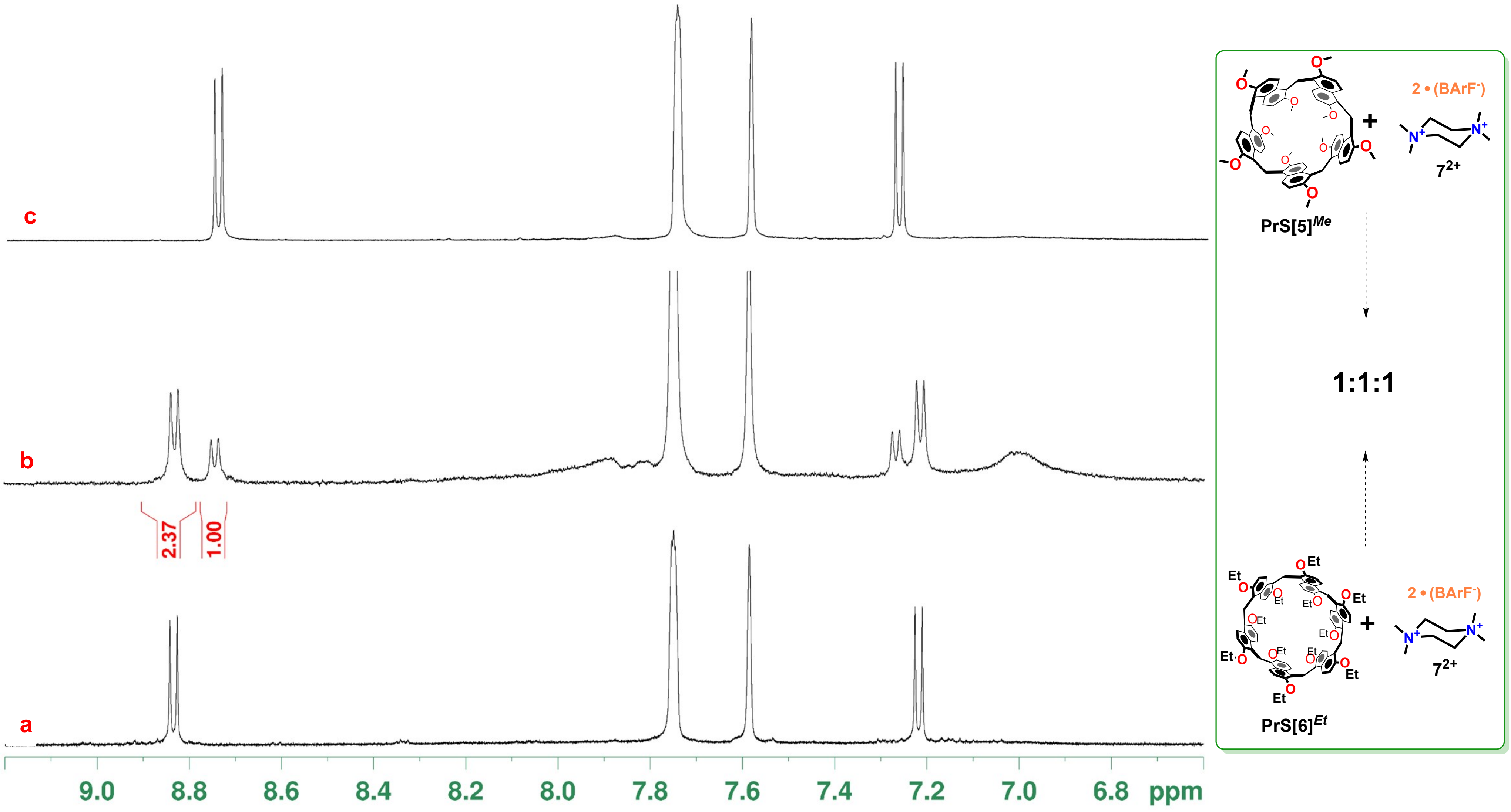

Figure S63: Significant portion of ^{1}H NMR spectra (600 MHz, CD_2Cl_2, 298 K) of: (a) an equimolar solution (2.10 mM) of **PrS[6]**^{Et} and **7**$(BArF)_2$ in 0.7 mL of CD_2Cl_2, (b) of **PrS[5]**^{Me} in the presence of 1 equivalent of **PrS[6]**^{Et} and 1 equivalent of **7**$(BArF)_2$ and (c) an equimolar solution (2.10 mM) of **PrS[5]**^{Me} and **7**$(BArF)_2$ in 0.7 mL of CD_2Cl_2.

Method B: $K_{rel} = K\,(\mathbf{7}^{2+}@\mathbf{PrS[6]}^{Et}) / K\,(\mathbf{7}^{2+}@\mathbf{PrS[5]}^{Me}) = K\,(\mathbf{5}^{2+}@\mathbf{PrS[6]}^{Et}) / 1.8\text{x}10^{7} = [(2.37/3.37)\text{x}0.0021]^2 / [(1.00/3.37)\text{x}0.0021]^2$

$K\,(\mathbf{7}^{2+}@\mathbf{PrS[6]}^{Et}) = 5.6 \text{ x } 1.8\cdot10^{7} = \mathbf{1.0\cdot10^{8}}\ M^{-1}$

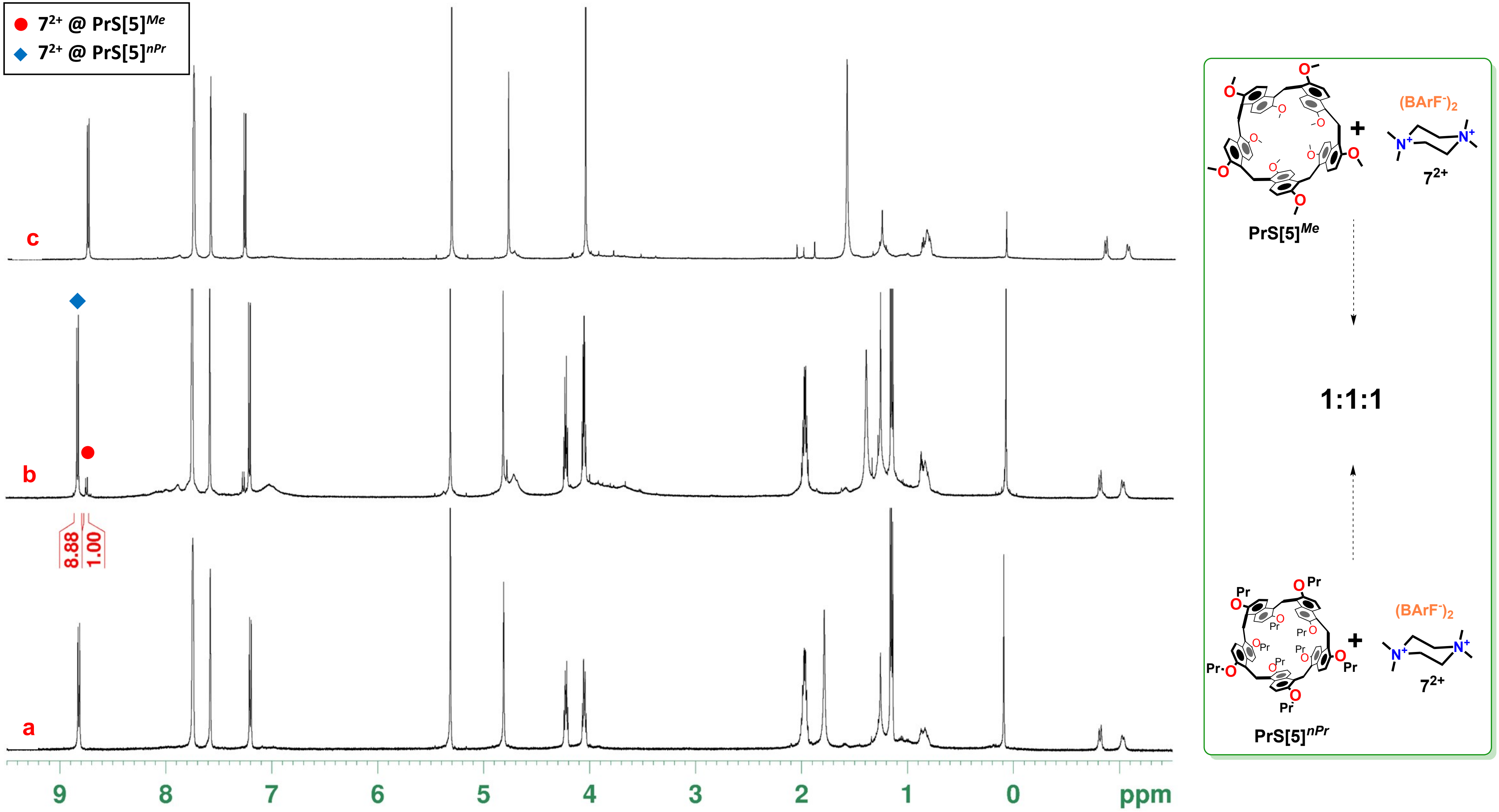

Figure S64: ^{1}H NMR spectra (600 MHz, CD_2Cl_2, 298 K) of: (a) an equimolar solution (2.10 mM) of **PrS[5]**^{nPr} and **7**(BArF)$_2$ in 0.7 mL of CD_2Cl_2, (b) of **PrS[5]**^{Me} in the presence of 1 equivalent of **PrS[5]**^{nPr} and 1 equivalent of **7**(BArF)$_2$ and (c) an equimolar solution (2.10 mM) of **PrS[5]**^{Me} and **7**(BArF)$_2$ in 0.7 mL of CD_2Cl_2.

Method B: $K_{rel} = K\,(\mathbf{7}^{2+}@\mathbf{PrS[5]}^{nPr}) / K\,(\mathbf{7}^{2+}@\mathbf{PrS[5]}^{Me}) = K\,(\mathbf{5}^{2+}@\mathbf{PrS[5]}^{nPr}) / 1.8\text{x}10^{7} = [(8.88/9.88)\text{x}0.0021]^2 / [(1.00/9.88)\text{x}0.0021]^2$

$K\,(\mathbf{7}^{2+}@\mathbf{PrS[5]}^{nPr}) = 79 \text{ x } 1.8\cdot10^{7} = \mathbf{1.4\cdot10^{9}}\ \text{M}^{-1}$

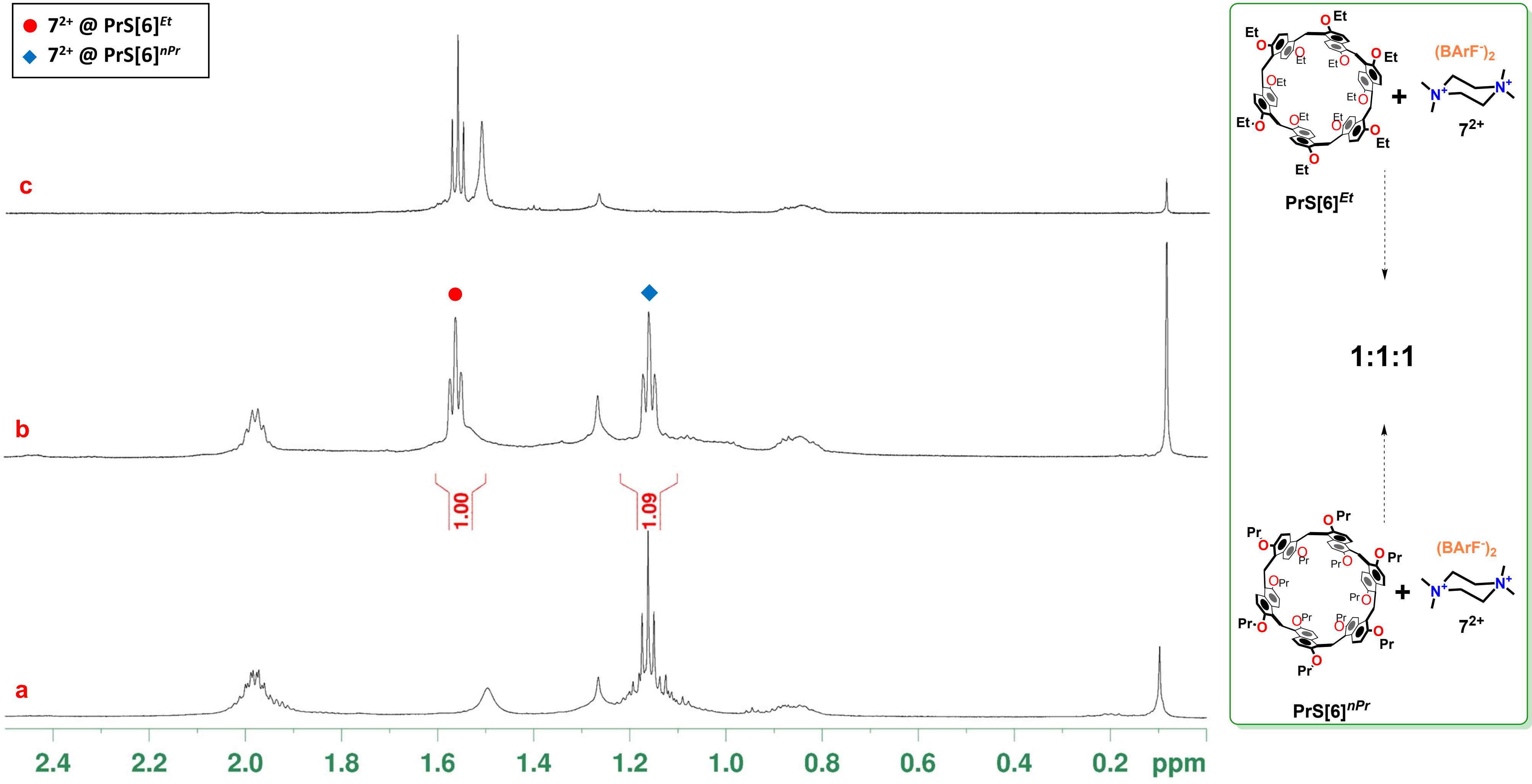

Figure S65: Significant portion of ^{1}H NMR spectra (600 MHz, CD_2Cl_2, 298 K) of: (a) an equimolar solution (2.40 mM) of **PrS[6]*nPr*** and **7**(BArF)$_2$ in 0.7 mL of CD_2Cl_2, (b) of **PrS[6]*Et*** in the presence of 1 equivalent of **PrS[6]*nPr*** and 1 equivalent of **7**(BArF)$_2$ and (c) an equimolar solution (2.40 mM) of **PrS[6]*Et*** and **7**(BArF)$_2$ in 0.7 mL of CD_2Cl_2.

Method B: $K_{rel} = K\,(\mathbf{7}^{2+}@\mathbf{PrS[6]}^{nPr}) / K\,(\mathbf{7}^{2+}@\mathbf{PrS[6]}^{Et}) = K\,(\mathbf{7}^{2+}@\mathbf{PrS[6]}^{nPr}) / 1.0\text{x}10^8 = [(1.09/2.09)\text{x}0.0024]^2 / [(1.00/2.09)\text{x}0.0024]^2$

$K\,(\mathbf{7}^{2+}@\mathbf{PrS[6]}^{nPr}) = 79 \times 1.0{\cdot}10^8 = \mathbf{1.2{\cdot}10^8}\ M^{-1}$

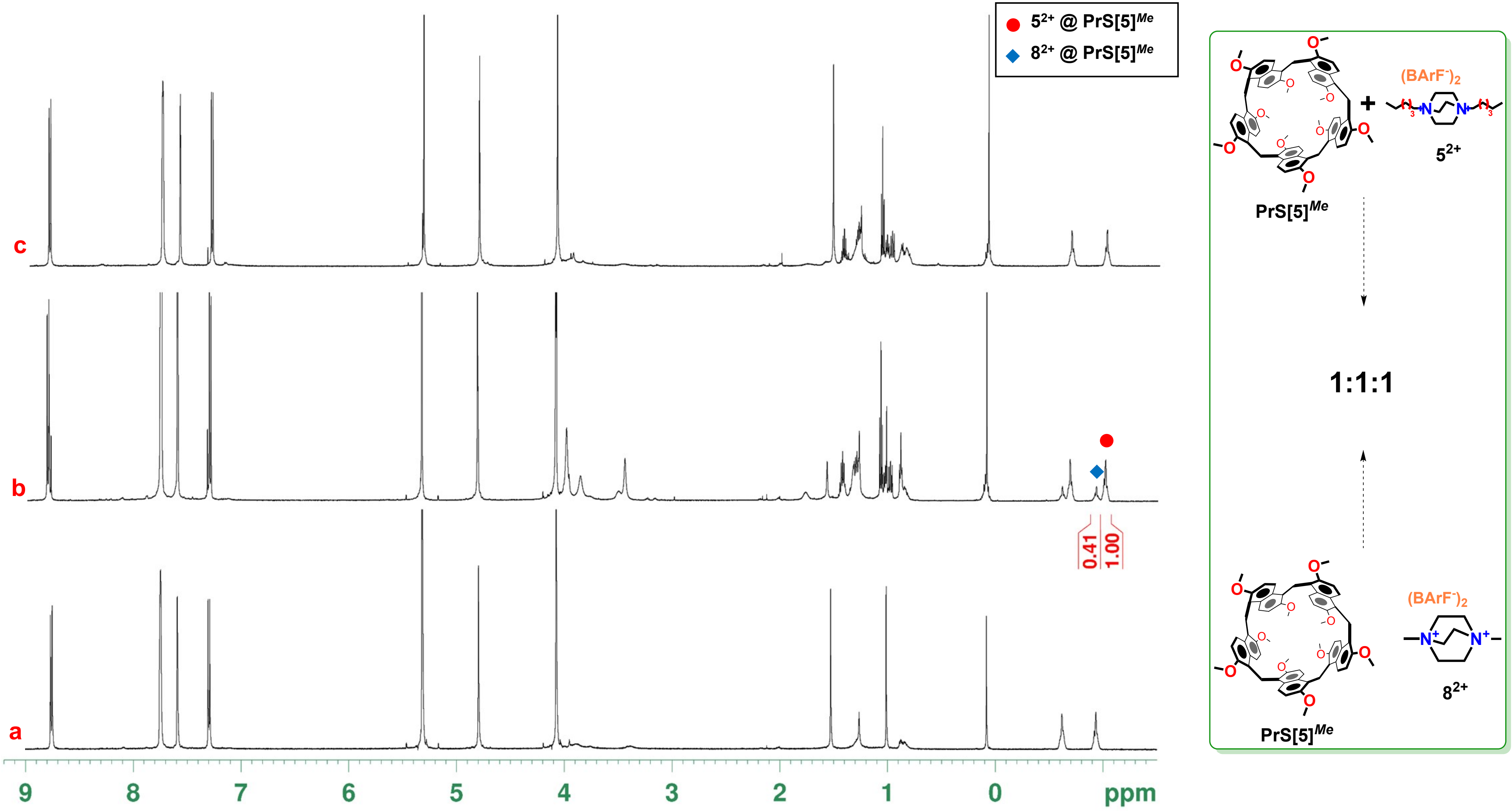

Figure S66: ^{1}H NMR spectra (600 MHz, CD_2Cl_2, 298 K) of: (a) an equimolar solution (2.85 mM) of **PrS[5]**^{Me} and **8**$(BArF)_2$ in 0.7 mL of CD_2Cl_2, (b) of **PrS[5]**^{Me} in the presence of 1 equivalent of **8**$(BArF)_2$ and 1 equivalent of **5**$(BArF)_2$ and (c) an equimolar solution (2.85 mM) of **PrS[5]**^{Me} and **5**$(BArF)_2$ in 0.7 mL of CD_2Cl_2.

Method B: $K_{rel} = K\,(\mathbf{8}^{2+}@\mathbf{PrS[5]}^{Me}) / K\,(\mathbf{5}^{2+}@\mathbf{PrS[5]}^{Me}) = K\,(\mathbf{8}^{2+}@\mathbf{PrS[5]}^{Me}) / 3.9\text{x}10^7 = [(0.41/1.41)\text{x}0.0028]^2 / [(1.00/1.41)\text{x}0.0028]^2$

$K\,(\mathbf{8}^{2+}@\mathbf{PrS[5]}^{Me}) = 0.17 \text{ x } 3.9\text{x}10^7 = \mathbf{6.6{\cdot}10^6}\ M^{-1}$

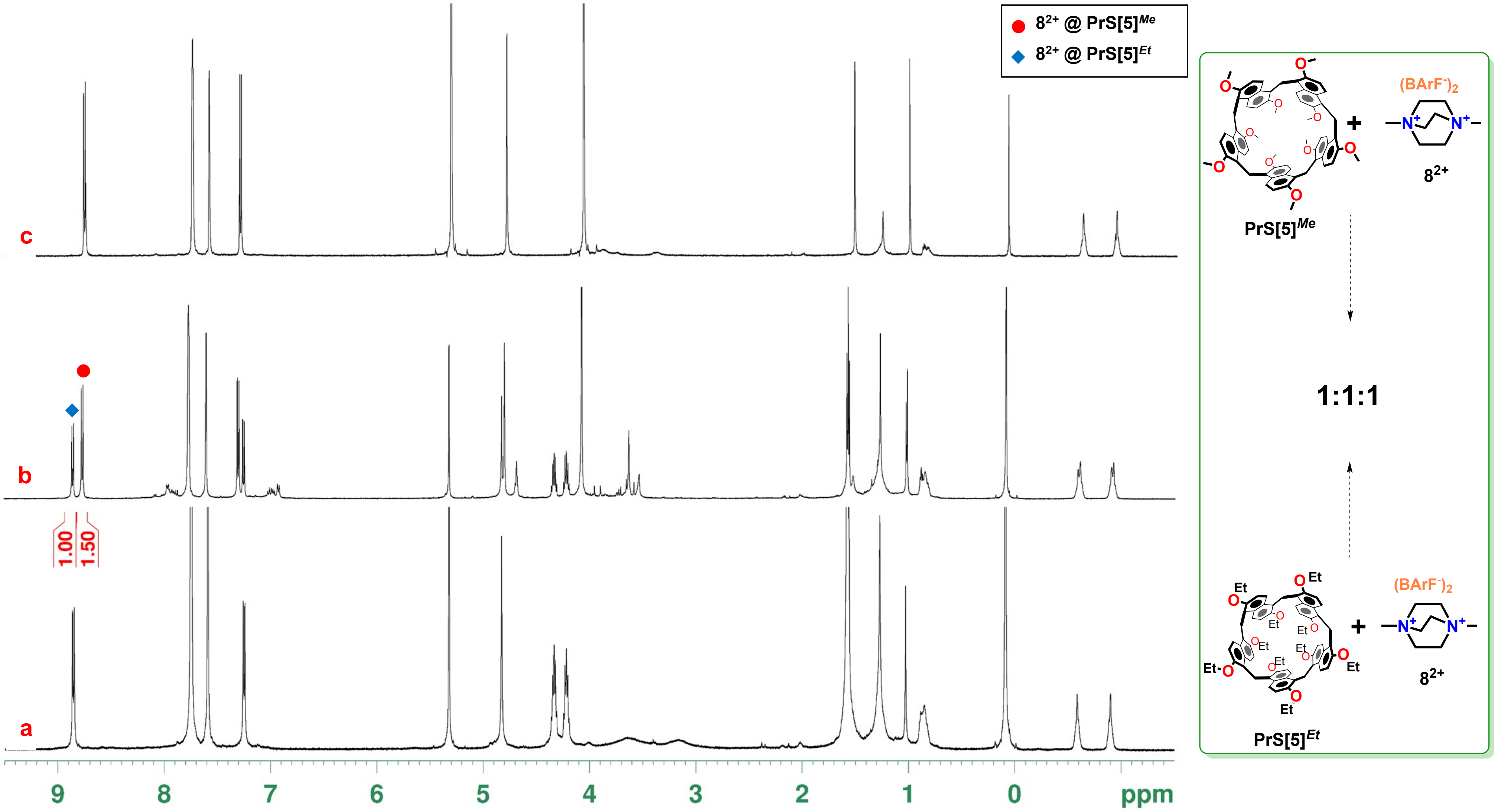

Figure S67: ^{1}H NMR spectra (600 MHz, CD_2Cl_2, 298 K) of: (a) an equimolar solution (7.25 mM) of **PrS[5]*Et*** and **8**$(BArF)_2$ in 0.7 mL of CD_2Cl_2, (b) of **PrS[5]*Me*** in the presence of 1 equivalent of **PrS[5]*Et*** and 1 equivalent of **8**$(BArF)_2$ and (c) an equimolar solution (7.25 mM) of **PrS[5]*Me*** and **8**$(BArF)_2$ in 0.7 mL of CD_2Cl_2.

Method B: $K_{rel} = K\,(\mathbf{8^{2+}@PrS[5]^{\mathit{Et}}}) \,/\, K\,(\mathbf{8^{2+}@PrS[5]^{\mathit{Me}}}) = K\,(\mathbf{8^{2+}@PrS[5]^{\mathit{Et}}}) \,/\, 6.6\text{x}10^{6} = [(1.00/2.50)\text{x}0.0072]^{2} \,/\, [(1.50/2.50)\text{x}0.0072]^{2}$

$K\,(\mathbf{8^{2+}@PrS[5]^{\mathit{Et}}}) = 0.45 \text{ x } 6.6\text{x}10^{6} = \mathbf{2.9\cdot10^{6}}\ M^{-1}$

K (**9^{2+}@PrS[5]^{Me}**) = 5780 M^{-1} (by integration, see Figure S68)

Competition experiment: **9^{2+}+ PrS[5]^{Me}+ PrS[5]^{Et}** = **9^{2+}@PrS[5]^{Me}** + **9^{2+}@PrS[5]^{Et}**→K (**9^{2+}@PrS[5]^{Et}**) = 1.0·10^5 M^{-1}(figure S69)

Competition experiment: **9^{2+}+ PrS[6]^{Me}+ PrS[5]^{Et}** = **9^{2+}@PrS[6]^{Me}** + **9^{2+}@PrS[5]^{Et}**→K (**9^{2+}@PrS[6]^{Me}**) = 3.7·10^3 M^{-1}(figure S70)

Competition experiment: **9^{2+}+ PrS[6]^{Me}+ 8^{2+}**= **9^{2+}@PrS[6]^{Me}** + **8^{2+}@PrS[6]^{Me}**→K (**8^{2+}@PrS[6]^{Me}**) = 4.2·10^3 M^{-1}(figure S71)

Competition experiment: **9^{2+}+ PrS[5]^{Me}+ PrS[5]^{nPr}**= **9^{2+}**@PrS[5]^{Me} +**9^{2+}@PrS[5]^{nPr}** →K **(9^{2+}@PrS[5]^{nPr})** = 4.8·10^5 M^{-1}(figure S72)

Competition experiment: **8^{2+}+ PrS[5]^{Me}+ PrS[5]^{nPr}**= **8^{2+}@PrS[5]^{Me} (Figure S59)+8^{2+}@PrS[5]^{nPr}** →K (**8^{2+}@PrS[5]^{nPr}**) = 6.3·10^7 M^{-1}(figure S73)

Competition experiment: **8^{2+}+ PrS[6]^{Me}+ PrS[6]^{Et}=8^{2+}@PrS[6]^{Me} +8^{2+}@PrS[6]^{Et}** →K (**8^{2+}@PrS[6]^{Et}**) = 420 M^{-1}(figure S74)

Competition experiment: **8^{2+}+ PrS[6]^{Me}+ PrS[6]^{nPr}** = **8^{2+}@PrS[6]^{Me}** +**8^{2+}@PrS[6]^{nPr}** →K (**8^{2+}@PrS[6]^{nPr}**) = 370 M^{-1}(figure S75)

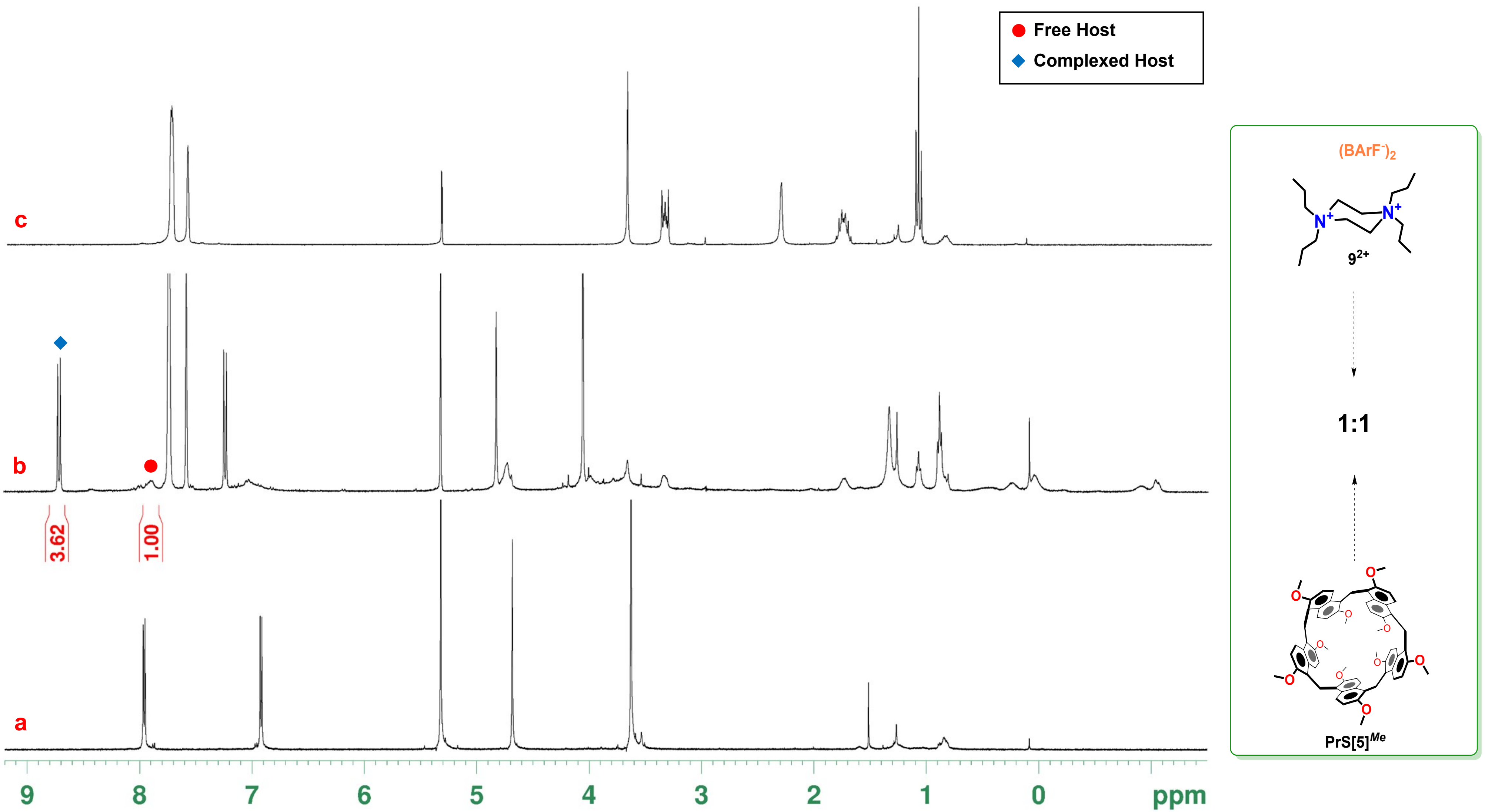

Figure S68: ^{1}H NMR spectra (400 MHz, CD_2Cl_2, 298 K) of: (a) a solution of **PrS[5]^{Me}** (b) an equimolar solution (2.85 mM) of **PrS[5]^{Me}** and **9**$(BArF)_2$ in 0.7 mL of CD_2Cl_2 and (c) a solution of **9**$(BArF)_2$.

Method A: [**9**$^{2+}$@**PrS[5]^{Me}**] **=** = [(3.62/4.62) x 0.0028] = 0.0022; [**PrS[6]^{nPr}**]$_{free}$= [**9**$^{2+}$]$_{free}$ = 0.0006: K (**6**$^{+}$@**PrS[6]^{Et}**) = 0.0022/3.8·10^{-7}= **5780** M^{-1}

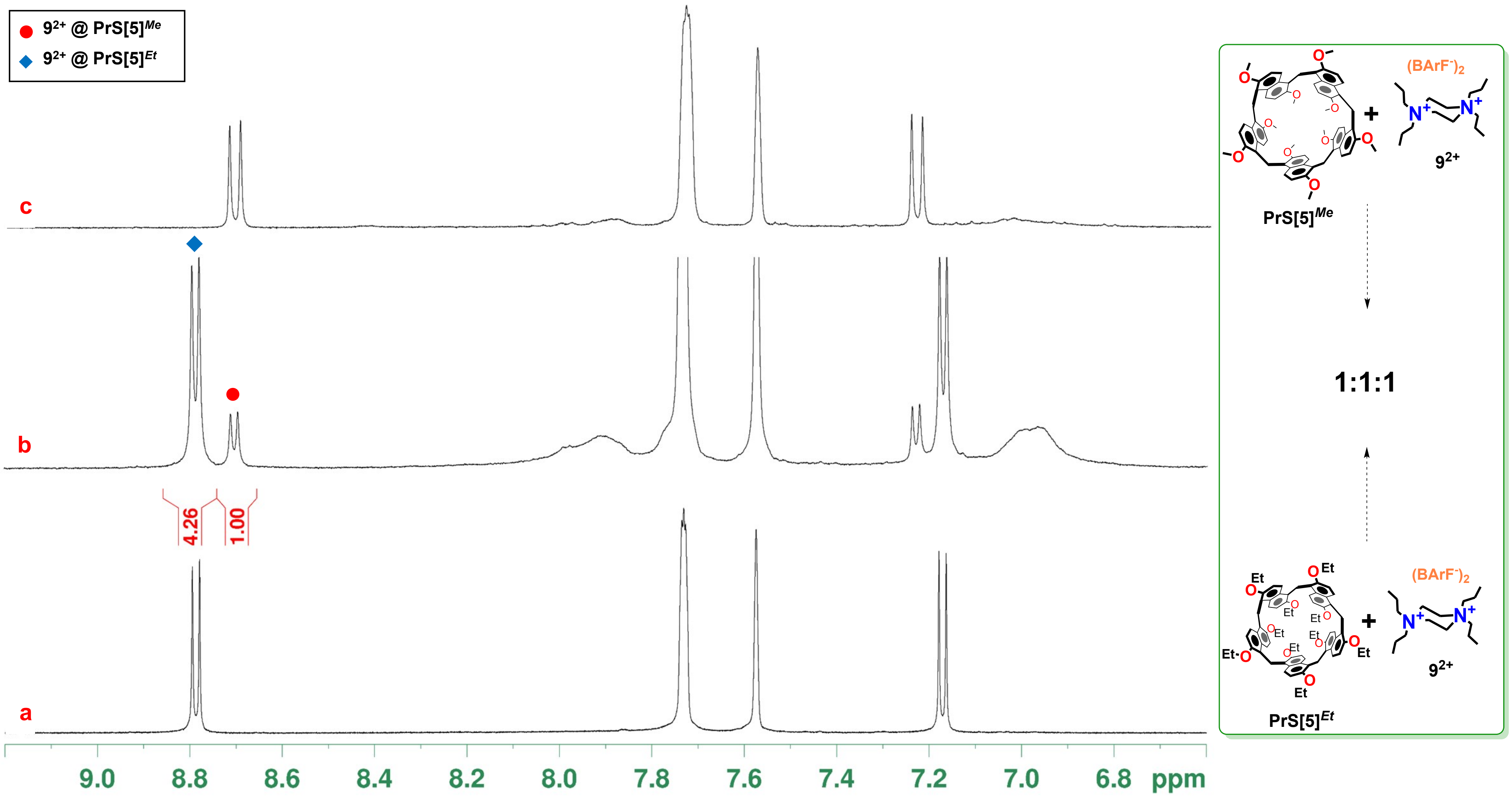

Figure S69: Significant portion of ^{1}H NMR spectra (600 MHz, CD_2Cl_2, 298 K) of: (a) an equimolar solution (2.50 mM) of **PrS[5]**^{Et} and **9**$(BArF)_2$ in 0.7 mL of CD_2Cl_2, (b) of **PrS[5]**^{Me} in the presence of 1 equivalent of **PrS[5]**^{Et} and 1 equivalent of **9**$(BArF)_2$ and (c) an equimolar solution (2.50 mM) of **PrS[5]**^{Me} and **9**$(BArF)_2$ in 0.7 mL of CD_2Cl_2.

Method B: $K_{rel} = K$ (**9**$^{2+}$**@PrS[5]**^{Et}) / K (**9**$^{2+}$**@PrS[5]**^{Me}) = K (**9**$^{2+}$**@PrS[5]**^{Et}) / 5780 = $[(4.26/5.26)x0.0025]^2$ / $[(1.00/5.26)x0.0025]^2$

K (**9**$^{2+}$**@PrS[5]**^{Et}) = 18 x 5780 = $\mathbf{1.0 \cdot 10^5}$ M^{-1}

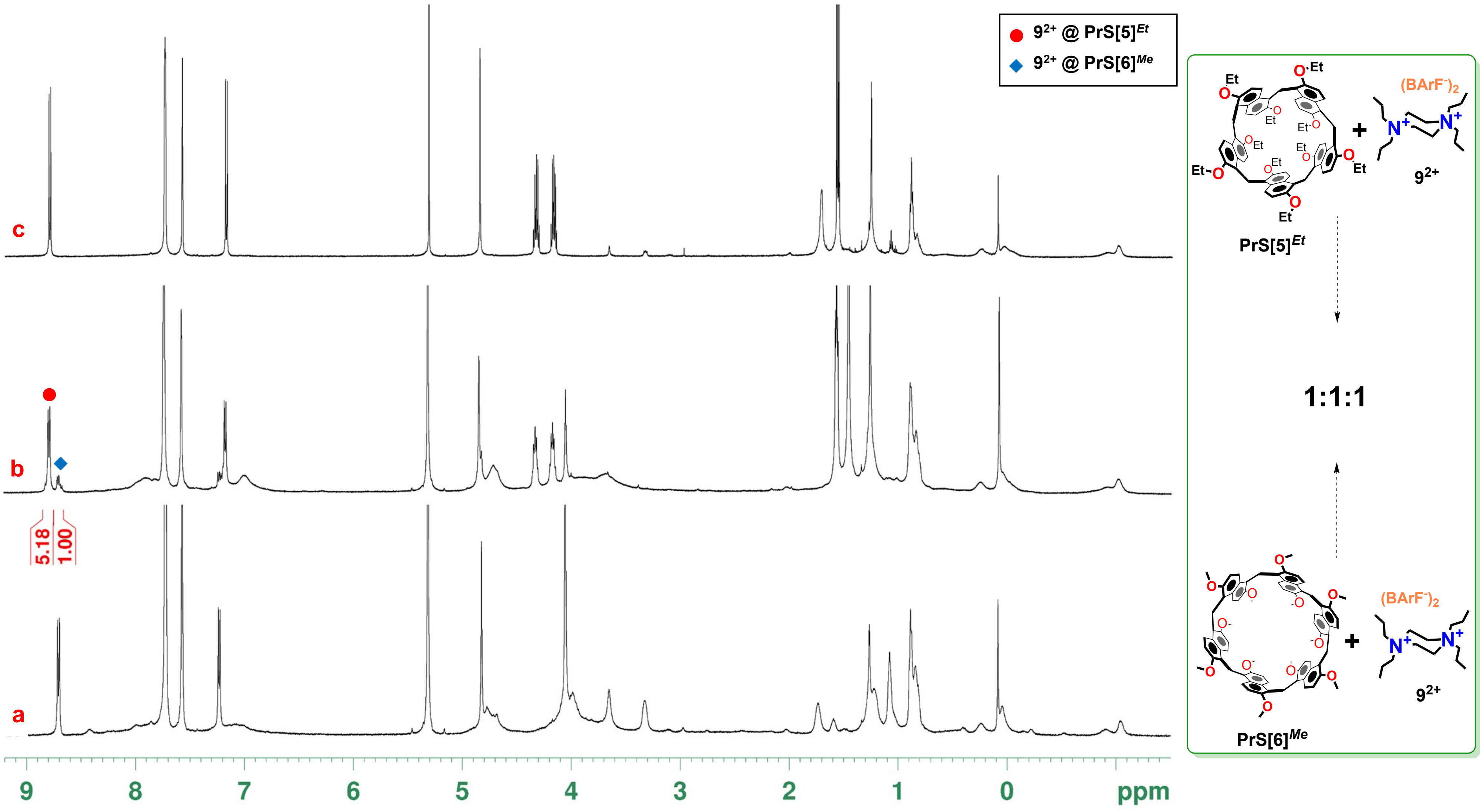

Figure S70: ^{1}H NMR spectra (600 MHz, CD_2Cl_2, 298 K) of: (a) an equimolar solution (2.38 mM) of **PrS[6]*^Me^*** and **9**$(BArF)_2$ in 0.7 mL of CD_2Cl_2, (b) of **PrS[5]*^Et^*** in the presence of 1 equivalent of **PrS[6]*^Me^*** and 1 equivalent of **9**$(BArF)_2$ and (c) an equimolar solution (2.38 mM) of **PrS[5]*^Et^*** and **9**$(BArF)_2$ in 0.7 mL of CD_2Cl_2.

Method B: $K_{rel} = K\,(\mathbf{9^{2+}@PrS[6]^{\mathit{Me}}}) / K\,(\mathbf{9^{2+}@PrS[5]^{\mathit{Et}}}) = K\,(\mathbf{9^{2+}@PrS[6]^{\mathit{Me}}}) / 1.0\cdot10^{5} = [(1.00/6.18)\mathrm{x}0.0024]^2 / [(5.18/6.18)\mathrm{x}0.0024]^2$

$K\,(\mathbf{9^{2+}@PrS[6]^{\mathit{Me}}}) = 0.04 \times 1.0\cdot10^{5} = \mathbf{3700}\ \mathrm{M}^{-1}$

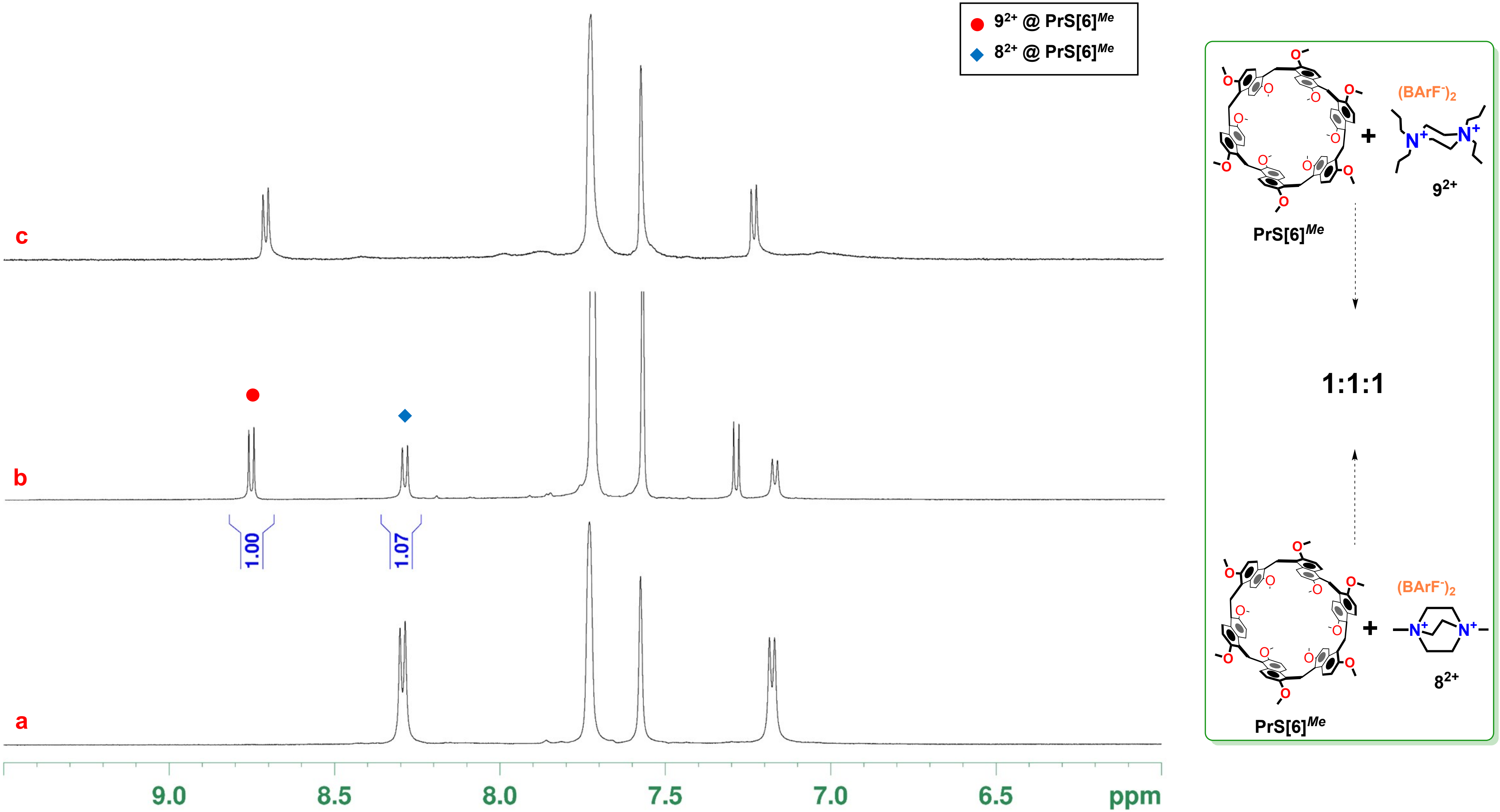

Figure S71: ^{1}H NMR spectra (600 MHz, CD_2Cl_2, 298 K) of: (a) an equimolar solution (2.38 mM) of **PrS[6]*^Me^*** and **8**$(BArF)_2$ in 0.7 mL of CD_2Cl_2, (b) of **PrS[6]*^Me^*** in the presence of 1 equivalent of **8**$(BArF)_2$ and 1 equivalent of **9**$(BArF)_2$ and (c) an equimolar solution (2.38 mM) of **PrS[6]*^Me^*** and **9**$(BArF)_2$ in 0.7 mL of CD_2Cl_2.

Method B: $K_{rel} = K\,(\mathbf{8^{2+}@PrS[6]^{\mathit{Me}}}) / K\,(\mathbf{9^{2+}@PrS[6]^{\mathit{Me}}}) = K\,(\mathbf{8^{2+}@PrS[6]^{\mathit{Me}}}) / 3700 = [(1.07/2.07)x0.0024]^2 / [(1.00/2.07)x0.0024]^2$

$K\,(\mathbf{8^{2+}@PrS[6]^{\mathit{Me}}}) = 1.14 \times 3700 = \mathbf{4200}\ M^{-1}$

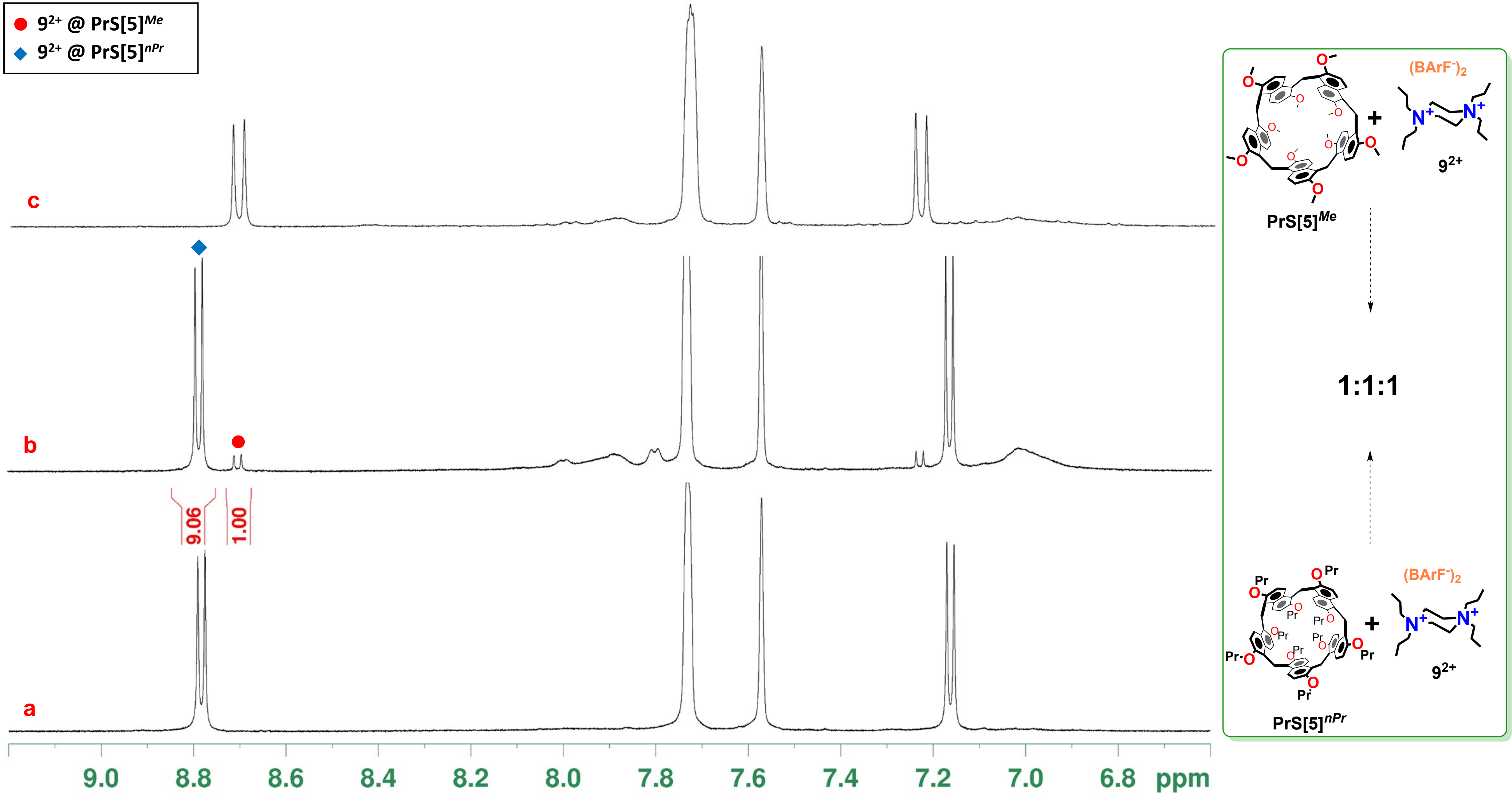

Figure S72: Significant portion of ^{1}H NMR spectra (600 MHz, CD_2Cl_2, 298 K) of: (a) an equimolar solution (3.12 mM) of **PrS[5]**^{nPr} and **9**(BArF)$_2$ in 0.7 mL of CD_2Cl_2, (b) of **PrS[5]**^{Me} in the presence of 1 equivalent of **PrS[5]**^{nPr} and 1 equivalent of **9**(BArF)$_2$ and (c) an equimolar solution (3.12 mM) of **PrS[5]**^{Me} and **9**(BArF)$_2$ in 0.7 mL of CD_2Cl_2.

Method B: $K_{rel} = K\,(\mathbf{9^{2+}@PrS[5]^{nPr}}) / K\,(\mathbf{9^{2+}@PrS[5]^{Me}}) = K\,(\mathbf{9^{2+}@PrS[5]^{nPr}}) / 5780 = [(9.1/10.1)x0.0031]^2 / [(1.00/10.1)x0.0031]^2$

$K\,(\mathbf{9^{2+}@PrS[5]^{nPr}}) = 82 \times 5780 = \mathbf{4.8 \cdot 10^5}\ \ M^{-1}$

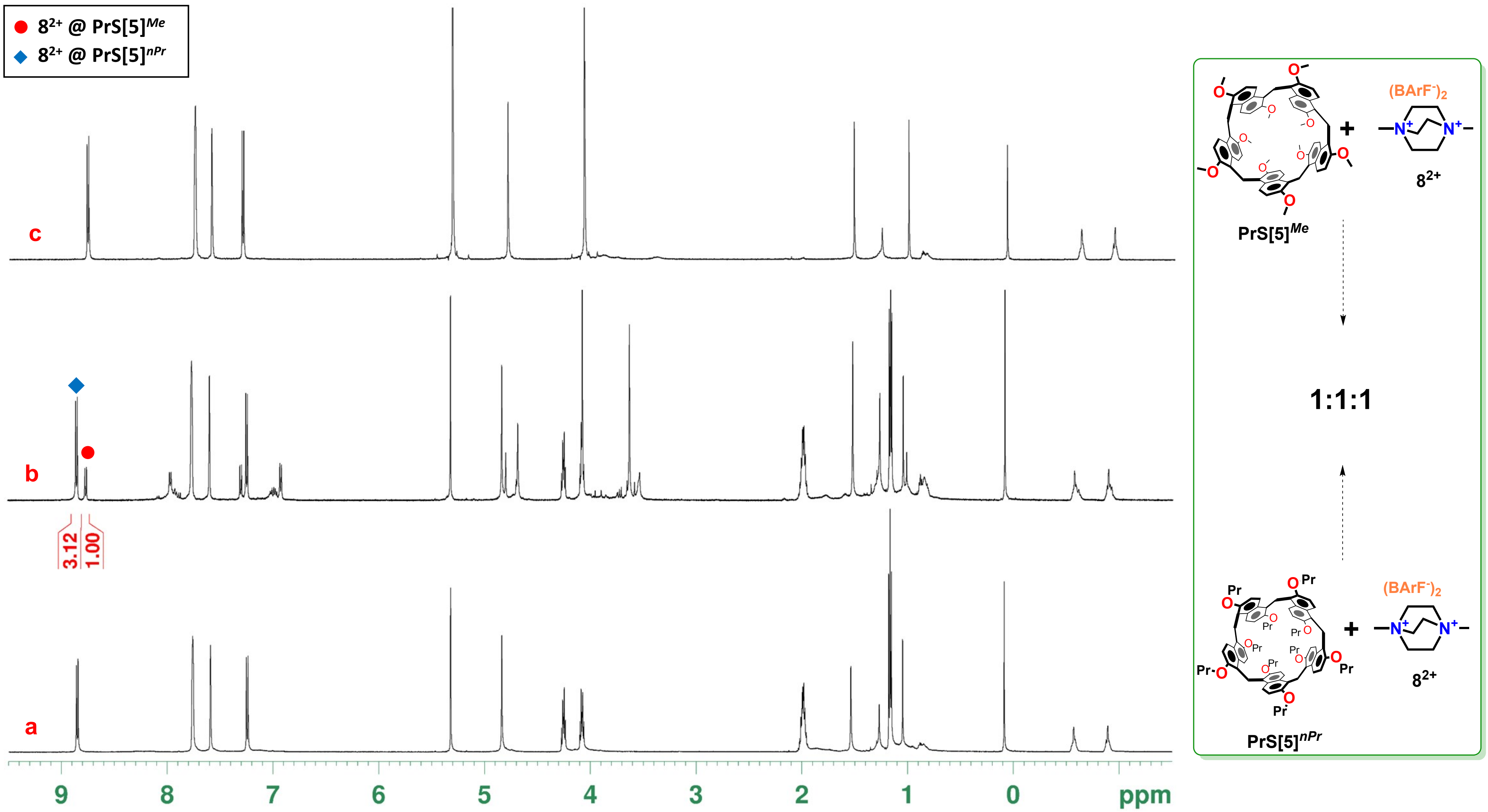

Figure S73: ^{1}H NMR spectra (600 MHz, CD_2Cl_2, 298 K) of: (a) an equimolar solution (4.68 mM) of **PrS[5]**^{nPr} and **8**$(BArF)_2$ in 0.7 mL of CD_2Cl_2, (b) of **PrS[5]**^{Me} in the presence of 1 equivalent of **PrS[5]**^{nPr} and 1 equivalent of **8**$(BArF)_2$ and (c) an equimolar solution (4.68 mM) of **PrS[5]**^{Me} and **8**$(BArF)_2$ in 0.7 mL of CD_2Cl_2.

Method B: $K_{rel} = K\,(\mathbf{8^{2+}@PrS[5]^{nPr}}) / K\,(\mathbf{8^{2+}@PrS[5]^{Me}}) = K\,(\mathbf{8^{2+}@PrS[5]^{nPr}}) / 6.6\cdot10^6 = [(3.12/4.12)x0.0047]^2 / [(1.00/4.12)x0.0047]^2$

$K\,(\mathbf{8^{2+}@PrS[5]^{nPr}}) = 9.6 \times 6.6\cdot10^6 = \mathbf{6.3\cdot10^7}\ M^{-1}$

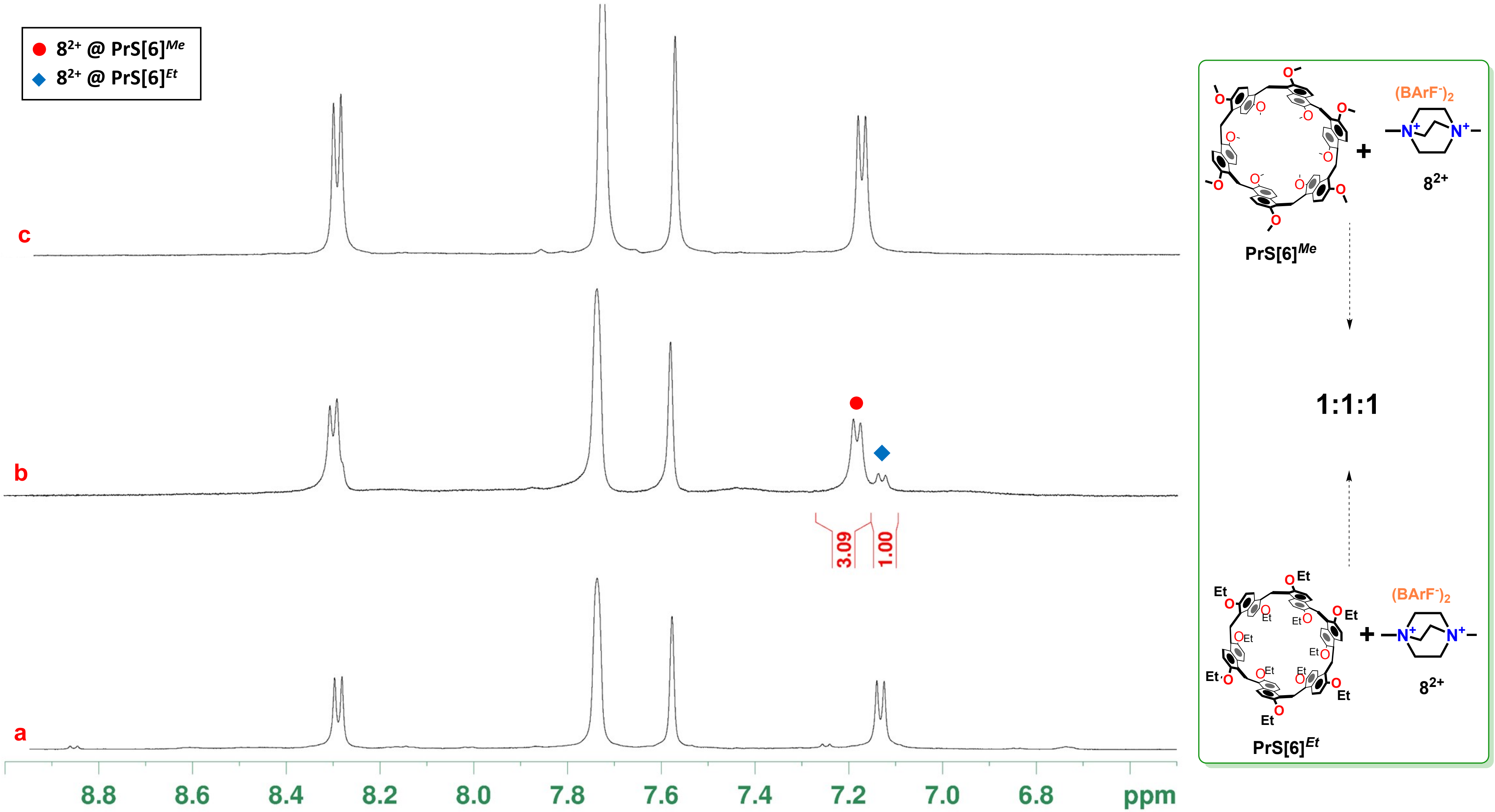

Figure S74: Significant portion of ^{1}H NMR spectra (600 MHz, CD_2Cl_2, 298 K) of: (a) an equimolar solution (2.38 mM) of **PrS[6]^{Et}** and **8**$(BArF)_2$ in 0.7 mL of CD_2Cl_2, (b) of **PrS[6]^{Me}** in the presence of 1 equivalent of **PrS[6]^{Et}** and 1 equivalent of **8**$(BArF)_2$ and (c) an equimolar solution (2.38 mM) of **PrS[6]^{Me}** and **8**$(BArF)_2$ in 0.7 mL of CD_2Cl_2.

Method B: $K_{rel} = K\,(\mathbf{8^{2+}@PrS[6]^{Et}}) / K\,(\mathbf{8^{2+}@PrS[6]^{Me}}) = K\,(\mathbf{8^{2+}@PrS[6]^{Et}}) / 4.2 \cdot 10^3 \quad = [(1.00/4.10)x0.0024]^2 / [(3.10/4.10)x0.0024]^2$

$K\,(\mathbf{8^{2+}@PrS[6]^{Et}}) = 0.1 \times 4.2 \cdot 10^3 = \mathbf{420}\ M^{-1}$

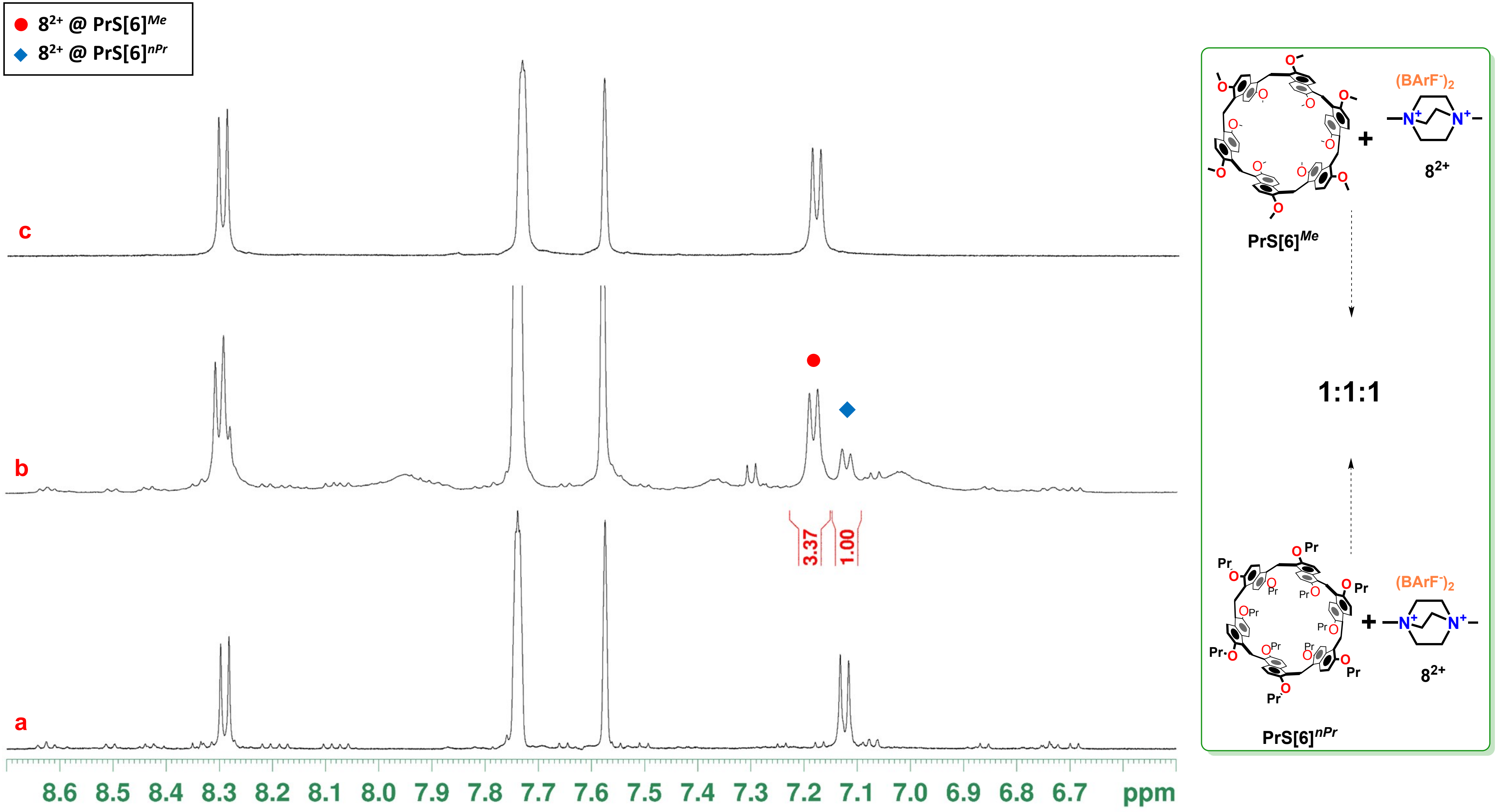

Figure S75: Significant portion of ^{1}H NMR spectra (600 MHz, CD_2Cl_2, 298 K) of: (a) an equimolar solution (2.38 mM) of **PrS[6]**^{nPr} and **8**$(BArF)_2$ in 0.7 mL of CD_2Cl_2, (b) of **PrS[6]**^{Me} in the presence of 1 equivalent of **PrS[6]**^{nPr} and 1 equivalent of **8**$(BArF)_2$ and (c) an equimolar solution (2.38 mM) of **PrS[6]**^{Me} and **8**$(BArF)_2$ in 0.7 mL of CD_2Cl_2.

Method B: $K_{rel} = K\,(\mathbf{8^{2+}@PrS[6]^{nPr}}) / K\,(\mathbf{8^{2+}@PrS[6]^{Me}}) = K\,(\mathbf{8^{2+}@PrS[6]^{nPr}}) / 4200 \quad = [(1.00/4.37)\text{x}0.0024]^2 / [(3.37/4.37)\text{x}0.0024]^2$

$K\,(\mathbf{8^{2+}@PrS[6]^{nPr}}) = 0.09 \text{ x } 4.2\cdot10^3 = \mathbf{370}\ M^{-1}$

Fluorescence Titration

Determination of association constant by fluorescence titration was performed at 298 K in a 1 cm Quartz cuvette in dichloromethane. The increase in the fluorescent intensity of **PrS[5]**$^{n\mathbf{Pr}}$ at 383 nm were recorded after excitation at 350 nm.

The host concentration was kept constant (2 μM) while the [**7(**BArF)$_2$] was varied from 0.0 - 4.0 μM (0.0 - 2.0 equivalents). Binding Constant (K) was determined by fitting of experimental data according to literature[3] and experiment was repeated three time. Error is smaller of ±15%.

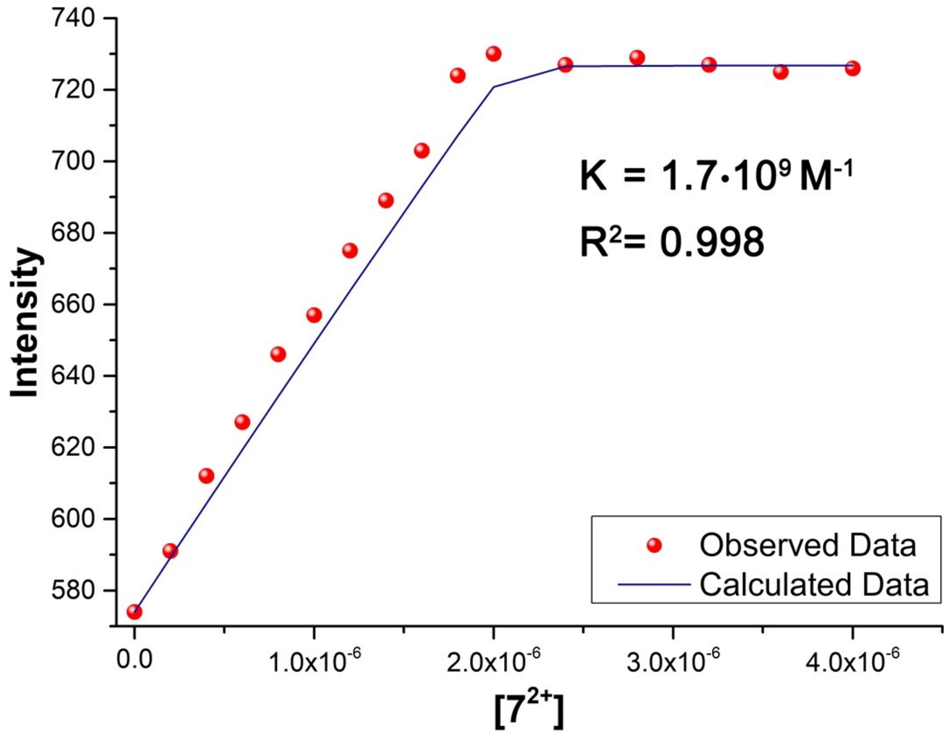

Figure S76: Correlation of [**7**$^{2+}$] on the fluorescence intensity of **PrS[5]**$^{n\mathbf{Pr}}$ for obtaining K.

[3] H. Yao, H. Ke, X. Zhang, S.-J. Pan, M.-S. Li, L.-P. Yang, G. Schreckenbach and W. Jiang, *J. Am. Chem. Soc.*, **2018**, *140*, 13466-13477.

DFT Calculations

Conformational studies have been performed using the DFT method incorporated in the Gaussian 16 package,[4] and using B97D3/SVP/SVPFIT level of theory in CH_2Cl_2 as solvent. The starting structure for DFT calculations were obtained by molecular mechanics calculation performed by YASARA software. All optimized structures were characterized by 0 imaginary frequency.

Guess structures for the transition states were located by using a homemade docking program able to manage dependent dihedrals and based on the MMFF94 force field. Starting from the reactive carbocation of the last closure step, initial structures of the final transition state were generated for the attack of the CH_2 moiety at both the Re and Si faces of the naphthyl unit, by activating al the rotatable bonds. All the guessed structures were optimized at the B97D3/SVP level of density functional theory (DFT).[5] Located transition states exhibit one large imaginary frequency associated to a normal coordinate corresponding to the stretching vibration of the forming (C-C) bond. Solvent (1,2-dichloroethane) effects were included via the polarizable continuum model.[6]

Gibbs free energy was evaluated at T = 298.15 K, assuming the standard state c_0 = 1 M.[7,8,9] The so-called ΔG_{50} approach was adopted according to which free energies are estimated by halving gas-phase computed entropic terms.[6,10,11] Indeed previous investigations suggest that including half of the 'gas-phase' (based on the particle in a box, rigid rotor, harmonic oscillator model) predicted entropy ($\Delta S_{soln} = 0.5\ \Delta S_{gas}$) is expected to yield solvation Gibbs free energies in good agreement with experimental data.[12] All DFT computations were carried out by using the Gaussian 16 software.[3]

[4] M. J. Frisch, G. W. Trucks, H. B. Schlegel, G. E. Scuseria, M. A. Robb, J. R. Cheeseman, G. Scalmani, V. Barone, B. Mennucci, G. A. Petersson, H. Nakatsuji, M. Caricato, X. Li, H. P. Hratchian, A. F. Izmaylov, J. Bloino, G. Zheng, J. L. Sonnenberg, M. Hada, M. Ehara, K. Toyota, R. Fukuda, J. Hasegawa, M. Ishida, T. Nakajima, Y. Honda, O. Kitao, H. Nakai, T. Vreven, J. A. Montgomery, J. E. Peralta, F. Ogliaro, M. Bearpark, J. J. Heyd, E. Brothers, K. N. Kudin, V. N. Staroverov, R. Kobayashi, J. Normand, K. Raghavachari, A. Rendell, J. C. Burant, S. S. Iyengar, J. Tomasi, M. Cossi, N. Rega, J. M. Millam, M. Klene, J. E. Knox, J. B. Cross, V. Bakken, C. Adamo, J. Jaramillo, R. Gomperts, R. E. Stratmann, O. Yazyev, A. J. Austin, R. Cammi, C. Pomelli, J. W. Ochterski, R. L. Martin, K. Morokuma, V. G. Zakrzewski, G. A. Voth, P. Salvador, J. J. Dannenberg, S. Dapprich, A. D. Daniels, Farkas, J. B. Foresman, J. V. Ortiz, J. Cioslowski, D. J. Fox, Gaussian 16 revision A.03.

[5] S. Grimme, S. Ehrlich, L. Goerigk, *J. Comp. Chem.* 2011, **32**, 1456-1465.

[6] J. Tomasi, B. Mennucci, R. Cammi, *Chem. Rev.* 2005, **105**, 2999-3094.

[7] R. E. Plata, D. A. Singleton, *J. Am. Chem. Soc.* 2015, **137**, 3811-3826.

[8] A. J. Garza, *J. Chem. Theory Comput.* 2019, **15**, 3204-3214.

[9] J. H. Jensen, *Phys. Chem. Chem. Phys.* 2015, **17**, 12441-12451.

[10] X. Huang, K. K. Zhang, Y. Shao, Y. Li, F. Gu, Qu, C. L.-B. Zhao, Z. Ke, *ACS Catal.* 2019, **9**, 5279-5289.

[11] A. Capobianco, S. Meninno, A. Lattanzi, *Catal. Sci. Technol.* 2020, **10**, 1422-1430.

[12] M. H. Abraham, *J. Am. Chem. Soc.* 1981, **103**, 6742-6744.

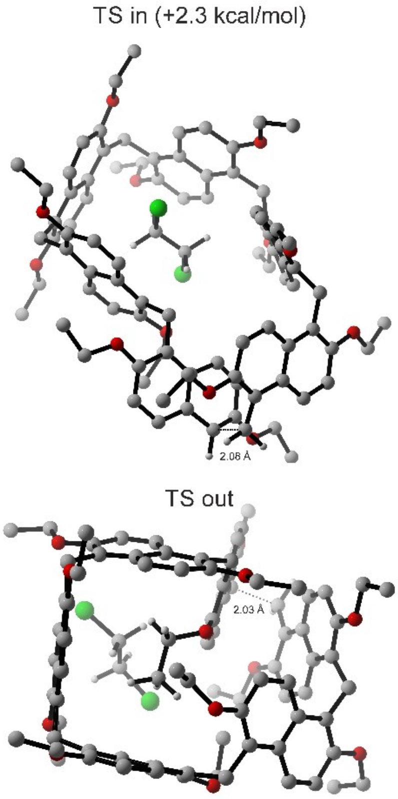

Figure S77. The most stable transition states for the closure step of **PrS[6]Et**: TS holding DCE inside the cavity (TS in, top); TS holding DCE outside the cavity and an ethyl group inside the cavity (TS out, bottom). Noncritical hydrogen atoms have been omitted for clarity. Gibbs free energy (B97D3/SVP, kcal/mol, in parentheses) refers to the most stable TS. Distances of the forming bond are given in Å.

DFT optimized structure of PrS[6]^{nPr}

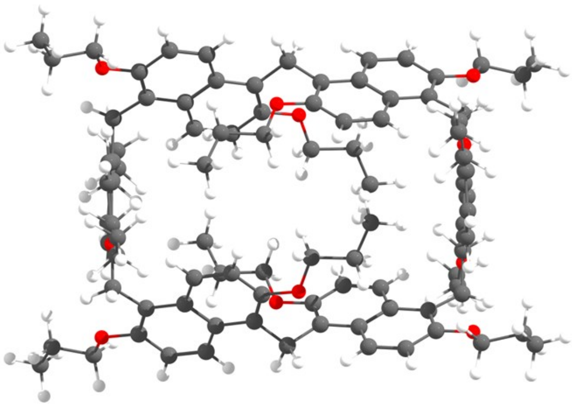

Figure S78. DFT-optimized structure (B97D3/SVP/SVPFIT) of **PrS[6]^{nPr}**.

C	-2.50566300	4.31649500	-0.10690400
C	-3.58851700	3.60603100	-0.76371100
C	-4.94062800	3.72539300	-0.29225400
C	-5.19367000	4.52824600	0.82897000
C	-4.13859800	5.21993800	1.48271700
C	-2.83985200	5.11775800	1.02224500
C	-1.15660800	4.19263900	-0.58378700
C	-0.90039400	3.35550500	-1.67624900
C	-1.95605100	2.65960200	-2.32471200
C	-3.26320800	2.79515100	-1.89004800
O	-6.49189700	4.61117200	1.26315600
C	-6.77447300	5.10222200	2.57465400
C	-8.23500900	4.80362900	2.88871500
C	-8.64181800	5.30564100	4.27665700
O	0.39195100	3.28977800	-2.11012100
C	0.82382600	2.15679900	-2.86414700
C	2.34214100	2.19510500	-2.96823900
C	2.89042700	1.01049100	-3.76459100
C	-0.00007200	4.98972400	0.00000200
H	-4.34628600	5.84038400	2.35826100
H	-2.05626500	5.64878100	1.56619200
H	-1.74271000	2.03450300	-3.19576600
H	-4.05268800	2.27425900	-2.43183700
H	-6.58104900	6.19361000	2.62499400
H	-6.10815300	4.60893900	3.31207200
H	-8.39559800	3.71154800	2.81185700
H	-8.86596200	5.27122600	2.10935600
H	-9.70221400	5.07939400	4.48360700
H	-8.03597100	4.83006300	5.07026300
H	-8.50827800	6.39976500	4.36501900
H	0.37217900	2.17120400	-3.87847800
H	0.48684500	1.22630100	-2.36253000
H	2.77267600	2.20837800	-1.95096400
H	2.63783700	3.15023300	-3.44043900
H	3.98241100	1.09408400	-3.87763800
H	2.68872000	0.05504100	-3.24981100
H	2.44557300	0.95408800	-4.77600300
C	2.50561100	-4.31656000	-0.10668500
C	3.58847200	-3.60612600	-0.76351600
C	4.94057300	-3.72542600	-0.29201900
C	5.19361200	-4.52821100	0.82925300
C	4.13853900	-5.21987600	1.48302500

C	2.83980200	-5.11774000	1.02252600
C	1.15655900	-4.19274200	-0.58360200
C	0.90037100	-3.35573300	-1.67616000
C	1.95603400	-2.65985900	-2.32463200
C	3.26318100	-2.79532500	-1.88991200
O	6.49183400	-4.61107600	1.26346500
C	6.77443800	-5.10223000	2.57491600
C	8.23499300	-4.80370500	2.88894600
C	8.64182000	-5.30579300	4.27685500
O	-0.39195300	-3.29006100	-2.11010700
C	-0.82376500	-2.15706600	-2.86414700
C	-2.34201800	-2.19572100	-2.96895500
C	-2.89025600	-1.01088900	-3.76503100
C	0.00000000	-4.98973500	0.00025300
H	4.34621200	-5.84025300	2.35862000
H	2.05622100	-5.64872400	1.56651300
H	1.74269600	-2.03483600	-3.19573900
H	4.05266300	-2.27443700	-2.43170800
H	6.58097700	-6.19361500	2.62518600
H	6.10816200	-4.60897400	3.31239400
H	8.39561900	-3.71162600	2.81213300
H	8.86590700	-5.27128900	2.10954800
H	9.70222900	-5.07959100	4.48378800
H	8.03600900	-4.83022600	5.07049700
H	8.50824300	-6.39991600	4.36517400
H	-0.37168400	-2.17119300	-3.87828600
H	-0.48721600	-1.22656600	-2.36223500
H	-2.77301000	-2.20956200	-1.95188900
H	-2.63724000	-3.15071200	-3.44174100
H	-3.98211000	-1.09495500	-3.87899600
H	-2.68941700	-0.05564800	-3.24952000
H	-2.44465200	-0.95367100	-4.77606900
C	-6.16818400	0.72235700	0.05620600
C	-6.16810100	-0.72173600	-0.05655100
C	-6.13544200	-1.54938100	1.11449300
C	-6.19637300	-0.93556400	2.37461100
C	-6.28761600	0.47856000	2.48951500
C	-6.25256600	1.28206900	1.36348600
C	-6.13555500	1.55002200	-1.11482500
C	-6.19636800	0.93621300	-2.37494500
C	-6.28741200	-0.47792500	-2.48986400
C	-6.25233700	-1.28145000	-1.36384500
O	-6.15535800	-1.75813600	3.46879800
C	-6.14512000	-1.20972200	4.78644500
C	-5.97620100	-2.35620600	5.77479400
C	-5.94646300	-1.86702000	7.22519700
O	-6.15538500	1.75880200	-3.46912500
C	-6.14492900	1.21040300	-4.78678200
C	-5.97609000	2.35692800	-5.77509600
C	-5.94618600	1.86778100	-7.22550700
C	-6.10787000	3.06915200	-1.01974600
H	-6.38440700	0.94730800	3.47197100
H	-6.32732100	2.36208900	1.48680100
H	-6.38408600	-0.94667100	-3.47232900
H	-6.32695600	-2.36148100	-1.48719600
H	-7.09205100	-0.66388700	4.97804900
H	-5.31310100	-0.48565700	4.89099800
H	-5.04084500	-2.89437200	5.53013300
H	-6.80306100	-3.07626000	5.62684100
H	-5.82142400	-2.71144800	7.92500000

H	-5.11051600	-1.16321600	7.39526200
H	-6.88314600	-1.34420100	7.49368300
H	-7.09176500	0.66443400	-4.97847700
H	-5.31279800	0.48645900	-4.89126900
H	-5.04082400	2.89521800	-5.53036300
H	-6.80305700	3.07686600	-5.62718000
H	-5.82119900	2.71224100	-7.92528000
H	-5.11013800	1.16408600	-7.39552700
H	-6.88278300	1.34485100	-7.49407700
C	6.16812100	-0.72232500	0.05628700
C	6.16810200	0.72176300	-0.05656300
C	6.13541900	1.54948400	1.11443200
C	6.19631500	0.93574500	2.37458900
C	6.28750600	-0.47837100	2.48958200
C	6.25244600	-1.28195200	1.36360600
C	6.13550500	-1.55006800	-1.11469100
C	6.19640900	-0.93634200	-2.37484800
C	6.28758400	0.47778300	-2.48985300
C	6.25247400	1.28138800	-1.36389000
O	6.15526700	1.75837900	3.46872300
C	6.14501900	1.21003800	4.78640000
C	5.97609600	2.35657700	5.77468400
C	5.94636500	1.86747700	7.22511500
O	6.15542400	-1.75899800	-3.46897900
C	6.14522200	-1.21069000	-4.78667600
C	5.97640300	-2.35726700	-5.77493200
C	5.94674200	-1.86821500	-7.22538100
C	6.10781100	-3.06919700	-1.01953000
H	6.38428000	-0.94705700	3.47206800
H	6.32717900	-2.36196300	1.48699500
H	6.38440800	0.94645700	-3.47233900
H	6.32719300	2.36140600	-1.48731300
H	7.09194900	0.66420800	4.97802600
H	5.31299800	0.48598500	4.89100000
H	5.04073400	2.89471800	5.52999400
H	6.80294700	3.07663300	5.62668600
H	5.82131400	2.71194400	7.92486800
H	5.11042700	1.16367200	7.39522200
H	6.88305400	1.34468600	7.49363400
H	7.09214000	-0.66483500	-4.97827900
H	5.31318000	-0.48666300	-4.89132900
H	5.04105200	-2.89544800	-5.53028600
H	6.80328000	-3.07727700	-5.62685800
H	5.82177800	-2.71271100	-7.92511500
H	5.11078100	-1.16445700	-7.39556500
H	6.88342300	-1.34538700	-7.49385500
C	-3.58831200	-3.60540500	0.76342200
C	-2.50550900	-4.31612700	0.10681200
C	-1.15645900	-4.19230700	0.58372400
C	-0.90017900	-3.35489500	1.67596300
C	-1.95577300	-2.65864400	2.32415400
C	-3.26293900	-2.79421600	1.88951500
C	-4.94043400	-3.72482000	0.29201800
C	-5.19352900	-4.52788000	-0.82904900
C	-4.13850000	-5.21978500	-1.48264300
C	-2.83975600	-5.11761800	-1.02215900
O	0.39215200	-3.28930000	2.10989000
C	0.82417100	-2.15645000	2.86399900
C	2.34238000	-2.19581700	2.96927600
C	2.89102000	-1.01133300	3.76559000

O	-6.49175900	-4.61068200	-1.26323100
C	-6.77460200	-5.10335000	-2.57404200
C	-8.23530000	-4.80545600	-2.88800700
C	-8.64237100	-5.30903000	-4.27530500
C	-6.10766100	-3.06850800	1.01946700
H	-1.74238800	-2.03325400	3.19498800
H	-4.05238700	-2.27307800	2.43112000
H	-4.34619600	-5.84037600	-2.35807800
H	-2.05621200	-5.64883400	-1.56597500
H	0.37177100	-2.17033200	3.87800200
H	0.48818000	-1.22582700	2.36193800
H	2.77370000	-2.20983600	1.95234900
H	2.63696200	-3.15098600	3.44209600
H	3.98276900	-1.09613900	3.88005900
H	2.69106200	-0.05596100	3.24998200
H	2.44499600	-0.95381500	4.77642400
H	-6.58093800	-6.19475300	-2.62314600
H	-6.10862900	-4.61079100	-3.31225900
H	-8.39613600	-3.71333300	-2.81225200
H	-8.86591700	-5.27238500	-2.10797700
H	-9.70288500	-5.08328100	-4.48218900
H	-8.03687500	-4.83412200	-5.06958100
H	-8.50856500	-6.40321100	-4.36255800
C	3.58826600	3.60549800	0.76328600
C	2.50545900	4.31619000	0.10664700
C	1.15640500	4.19239200	0.58356400
C	0.90014000	3.35509400	1.67588100
C	1.95573300	2.65887400	2.32410200
C	3.26289400	2.79438600	1.88943800
C	4.94038700	3.72486900	0.29185100
C	5.19347100	4.52787000	-0.82926300
C	4.13843600	5.21972500	-1.48289700
C	2.83969800	5.11758800	-1.02239000
O	-0.39217500	3.28957400	2.10989600
C	-0.82425200	2.15654400	2.86372400
C	-2.34243300	2.19606500	2.96922700
C	-2.89108800	1.01138600	3.76523400
O	6.49170100	4.61065000	-1.26344900
C	6.77458100	5.10347200	-2.57419300
C	8.23531700	4.80570400	-2.88809600
C	8.64243300	5.30940100	-4.27533700
C	6.10762900	3.06861100	1.01932600
H	1.74233700	2.03355100	3.19498300
H	4.05232600	2.27325900	2.43106900
H	4.34611900	5.84026100	-2.35837400
H	2.05615500	5.64878300	-1.56622900
H	-0.37173300	2.17006700	3.87767500
H	-0.48841700	1.22603700	2.36134300
H	-2.77388700	2.21046100	1.95236300
H	-2.63682200	3.15111900	3.44240800
H	-3.98279400	1.09632800	3.88000200
H	-2.69141500	0.05617700	3.24922200
H	-2.44482800	0.95341700	4.77593900
H	6.58084700	6.19486600	-2.62319400
H	6.10868500	4.61094100	-3.31250000
H	8.39622500	3.71358600	-2.81240900
H	8.86585900	5.27262200	-2.10799900
H	9.70297900	5.08375000	-4.48217000
H	8.03702400	4.83449600	-5.06968000
H	8.50854700	6.40357700	-4.36252600

H	-6.14096800	-3.46719300	2.04560100
H	-7.03531700	-3.40313400	0.52831800
H	-7.03551500	3.40373300	-0.52854400
H	-6.14122800	3.46788500	-2.04585700
H	-0.37524700	5.65710500	0.79031700
H	0.37514700	5.65733000	-0.79010000
H	6.14094400	3.46734900	2.04543800
H	7.03527700	3.40322800	0.52815600
H	7.03546000	-3.40374800	-0.52831200
H	6.14117300	-3.46798000	-2.04562000
H	0.37514700	-5.65705100	0.79063400
H	-0.37523000	-5.65740900	-0.78978500

1 2 1.0 6 1.5 7 1.5
2 3 1.5 10 1.5
3 4 1.5 93 1.0
4 5 1.5 11 1.0
5 6 2.0 20 1.0
6 21 1.0
7 8 1.5 19 1.0
8 9 1.5 15 1.0
9 10 2.0 22 1.0
10 23 1.0
11 12 1.0
12 13 1.0 24 1.0 25 1.0
13 14 1.0 26 1.0 27 1.0
14 28 1.0 29 1.0 30 1.0
15 16 1.0
16 17 1.0 31 1.0 32 1.0
17 18 1.0 33 1.0 34 1.0
18 35 1.0 36 1.0 37 1.0
19 188 1.0 227 1.0 228 1.0
20
21
22
23
24
25
26
27
28
29
30
31
32
33
34
35
36
37
38 39 1.0 43 1.5 44 1.5
39 40 1.5 47 1.5
40 41 1.5 130 1.0
41 42 1.5 48 1.0
42 43 2.0 57 1.0
43 58 1.0
44 45 1.5 56 1.0
45 46 1.5 52 1.0
46 47 2.0 59 1.0
47 60 1.0

48 49 1.0
49 50 1.0 61 1.0 62 1.0
50 51 1.0 63 1.0 64 1.0
51 65 1.0 66 1.0 67 1.0
52 53 1.0
53 54 1.0 68 1.0 69 1.0
54 55 1.0 70 1.0 71 1.0
55 72 1.0 73 1.0 74 1.0
56 151 1.0 233 1.0 234 1.0
57
58
59
60
61
62
63
64
65
66
67
68
69
70
71
72
73
74
75 76 1.0 80 1.5 81 1.5
76 77 1.5 84 1.5
77 78 1.5 167 1.0
78 79 1.5 85 1.0
79 80 2.0 94 1.0
80 95 1.0
81 82 1.5 93 1.0
82 83 1.5 89 1.0
83 84 2.0 96 1.0
84 97 1.0
85 86 1.0
86 87 1.0 98 1.0 99 1.0
87 88 1.0 100 1.0 101 1.0
88 102 1.0 103 1.0 104 1.0
89 90 1.0
90 91 1.0 105 1.0 106 1.0
91 92 1.0 107 1.0 108 1.0
92 109 1.0 110 1.0 111 1.0
93 225 1.0 226 1.0
94
95
96
97
98
99
100
101
102
103
104
105
106
107

108
109
110
111
112 113 1.0 117 1.5 118 1.5
113 114 1.5 121 1.5
114 115 1.5 204 1.0
115 116 1.5 122 1.0
116 117 2.0 131 1.0
117 132 1.0
118 119 1.5 130 1.0
119 120 1.5 126 1.0
120 121 2.0 133 1.0
121 134 1.0
122 123 1.0
123 124 1.0 135 1.0 136 1.0
124 125 1.0 137 1.0 138 1.0
125 139 1.0 140 1.0 141 1.0
126 127 1.0
127 128 1.0 142 1.0 143 1.0
128 129 1.0 144 1.0 145 1.0
129 146 1.0 147 1.0 148 1.0
130 231 1.0 232 1.0
131
132
133
134
135
136
137
138
139
140
141
142
143
144
145
146
147
148
149 150 1.0 154 1.5 155 1.5
150 151 1.5 158 1.5
151 152 1.5
152 153 1.5 159 1.0
153 154 2.0 168 1.0
154 169 1.0
155 156 1.5 167 1.0
156 157 1.5 163 1.0
157 158 2.0 170 1.0
158 171 1.0
159 160 1.0
160 161 1.0 172 1.0 173 1.0
161 162 1.0 174 1.0 175 1.0
162 176 1.0 177 1.0 178 1.0
163 164 1.0
164 165 1.0 179 1.0 180 1.0
165 166 1.0 181 1.0 182 1.0
166 183 1.0 184 1.0 185 1.0
167 223 1.0 224 1.0

168
169
170
171
172
173
174
175
176
177
178
179
180
181
182
183
184
185
186 187 1.0 191 1.5 192 1.5
187 188 1.5 195 1.5
188 189 1.5
189 190 1.5 196 1.0
190 191 2.0 205 1.0
191 206 1.0
192 193 1.5 204 1.0
193 194 1.5 200 1.0
194 195 2.0 207 1.0
195 208 1.0
196 197 1.0
197 198 1.0 209 1.0 210 1.0
198 199 1.0 211 1.0 212 1.0
199 213 1.0 214 1.0 215 1.0
200 201 1.0
201 202 1.0 216 1.0 217 1.0
202 203 1.0 218 1.0 219 1.0
203 220 1.0 221 1.0 222 1.0
204 229 1.0 230 1.0
205
206
207
208
209
210
211
212
213
214
215
216
217
218
219
220
221
222
223
224
225
226
227

228
229
230
231
232
233
234

0 imaginary frequency

DFT optimized structure of the PrS[5]^{nPr}

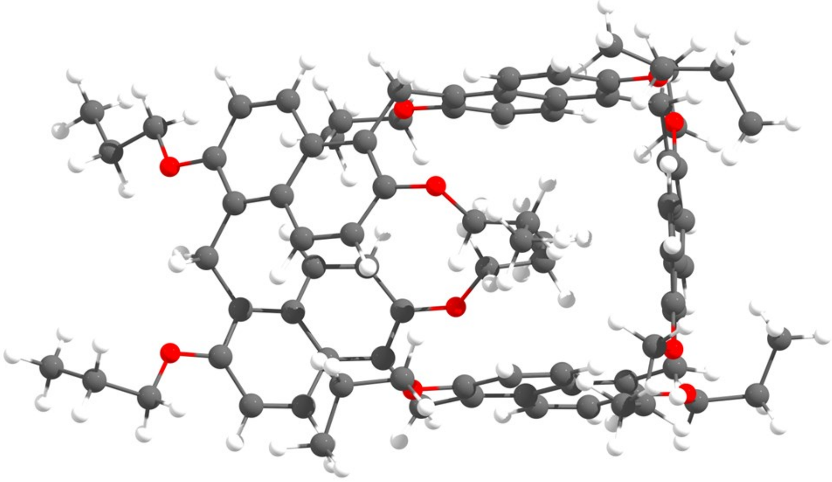

Figure S79. DFT-optimized structure (B97D3/SVP/SVPFIT) of the **PrS[5]^{nPr}**.

C	-4.05515200	-0.18062000	1.50819400
C	-2.43087900	2.10928700	2.00701800
C	-1.95650500	0.81841600	2.23826400
C	-2.79267200	-0.31336400	2.04261800
H	-4.67592300	-1.06546800	1.36269700
H	-2.40822000	-1.29438100	2.33200300
C	-4.55536800	1.09996400	1.12767100
C	-3.76367600	2.26396300	1.47650700
C	-4.34325600	3.53836000	1.23319600
C	-5.56949000	3.67430700	0.60727700
C	-6.26857500	2.53007400	0.14258100
C	-5.77199300	1.23897600	0.38159400
H	-3.81032600	4.44516600	1.52607400
H	-5.97170800	4.67604100	0.44000900
C	-6.53771300	0.06184000	-0.20800000
H	-7.24149600	-0.32587400	0.54905200
H	-7.18758400	0.46481800	-1.00447900
C	-1.53895800	3.30193100	2.33372800
H	-1.34887800	3.30349500	3.41732100
H	-2.07965400	4.23510000	2.12056900
O	-7.43372200	2.62316300	-0.57125500
O	-0.67956100	0.61886300	2.71012700
C	-7.92943100	3.89426300	-0.97273400
H	-8.15716400	4.51452300	-0.07949000
H	-7.16119400	4.43159000	-1.56763700
C	0.20217400	-0.01084800	1.75874000
H	-0.28280600	-0.92177500	1.35311900

H	0.36103500	0.68660800	0.91556000
C	2.16484500	3.45049200	0.04424800
C	-0.22227300	3.35086200	1.57734700
C	-0.25996100	3.59598800	0.19746000
C	0.93792600	3.64484900	-0.56333200
H	3.06464200	3.51527500	-0.56337600
H	0.90673500	3.84300500	-1.63703600
C	1.05125500	3.18704800	2.21913700
C	2.27430100	3.21027200	1.44188000
C	3.55375300	3.01205100	2.06373400
C	3.60533900	2.86305600	3.45524400
C	2.41226200	2.85296600	4.22860100
C	1.17738300	2.99257900	3.62393800
H	2.45684100	2.71314800	5.31138100
O	-1.49173600	3.80315100	-0.35688000
O	4.84530600	2.70454900	4.02389300
C	4.83771100	2.98602400	1.24179200
H	5.68858100	2.92363000	1.93792800
H	4.94331600	3.94408500	0.70925000
C	5.05907200	-0.20022500	-1.73989500
C	5.12478100	1.12747700	-2.11763500
C	5.04558100	2.16237600	-1.14793600
C	4.94315200	1.86303100	0.21658900
H	5.11438200	-0.96761100	-2.51020500
H	5.23287400	1.37407500	-3.17711900
C	4.95417000	-0.57446000	-0.37028100
C	4.95180600	0.48421700	0.61646500
C	4.98635800	0.10752700	1.98771800
C	4.99369800	-1.22155800	2.36820100
C	4.92196900	-2.25505900	1.39614200
C	4.88722700	-1.95155000	0.02778300
H	5.02848400	0.87342100	2.76073600
H	5.04332600	-1.46525400	3.43201900
O	5.05748100	3.48838800	-1.50453700
O	4.86837200	-3.58158300	1.74517600
H	4.84943100	-4.03213000	-0.46825200
H	5.70668200	-3.03548400	-1.64102200
C	4.80915600	-3.07075300	-1.00385300
C	1.32696100	-2.99465500	-3.63846900
C	3.58492200	-3.05959900	-1.91232800
C	3.73903600	-2.92688400	-3.29805500
C	2.60496100	-2.89310200	-4.15465200
H	0.48350900	-2.92206100	-4.32734400
H	2.73052900	-2.76725200	-5.23278700
C	2.26002100	-3.21453700	-1.38074700
C	1.09612800	-3.16964400	-2.24441400
C	-0.22353500	-3.29802900	-1.69462400
C	-0.36374700	-3.53078200	-0.31921900
C	0.77447900	-3.60450200	0.52492200
C	2.04591000	-3.43741900	0.00771200
H	0.66136200	-3.79996800	1.59327900
H	2.89534200	-3.51066600	0.68376700
O	5.02003700	-2.80829400	-3.77834500
O	-1.63652700	-3.69604000	0.14765900
C	-1.48177600	-3.22897800	-2.54421600
H	-2.04434700	-4.16060100	-2.38615000
H	-1.21274000	-3.21547500	-3.61039900
C	-4.01130600	0.27052200	-1.84851600
C	-2.72853600	0.39178500	-2.33661300
C	-1.89190000	-0.74653100	-2.48601400

C	-2.38431100	-2.03265300	-2.26650800
H	-4.62959200	1.16185900	-1.73104200
H	-2.32989700	1.36895300	-2.62107900
C	-3.73705400	-2.17651000	-1.78762600
C	-4.53177000	-1.00450700	-1.47584900
C	-5.76756200	-1.12978400	-0.75846200
C	-6.27175900	-2.41535400	-0.51024000
C	-5.57341400	-3.56778600	-0.95664600
C	-4.33214800	-3.44637600	-1.55383400
H	-5.99017800	-4.56472300	-0.79178200
H	-3.79417500	-4.35944900	-1.81717100
O	-0.59078900	-0.56136400	-2.89619600
O	-7.44602300	-2.50139300	0.19197300
C	4.72176700	3.86081100	-2.83836200
H	3.83355200	3.28979000	-3.17716300
H	5.55754400	3.61822700	-3.52867100
C	4.96231200	2.42397500	5.41464300
H	4.54374100	3.26380200	6.00668500
H	4.38028900	1.51120600	5.66574000
C	-1.60609400	4.00533800	-1.76235600
H	-1.00373400	4.88545600	-2.07297100
H	-1.20242700	3.12307100	-2.29787000
C	4.77243600	-3.95436400	3.11672700
H	5.69299200	-3.64843100	3.65567000
H	3.91404200	-3.43320100	3.58994100
C	5.24284500	-2.52602000	-5.15583200
H	4.70020600	-1.59982800	-5.44327700
H	4.84979500	-3.35439600	-5.78065200
C	-7.84492200	-3.74540400	0.75209800
H	-7.00210100	-4.19761600	1.31772600
H	-8.12519800	-4.46102600	-0.05028900
C	0.24937500	0.05442100	-1.89922100
H	-0.23616300	0.97956600	-1.52740700
H	0.34212900	-0.63919900	-1.04349100
C	-1.85712000	-3.95749700	1.52788900
H	-1.36207800	-4.90718800	1.82388900
H	-1.40858900	-3.15130600	2.14463100
H	0.28532300	2.93442400	4.25007800
C	-9.19299500	3.67335200	-1.79700000
H	-8.92912300	3.06939700	-2.68627100
H	-9.52062300	4.66169700	-2.17362100
C	1.52897100	-0.36155200	2.40805300
H	2.06088900	0.56495300	2.67640500
H	2.14451100	-0.85333300	1.63317100
C	4.43195100	5.35674300	-2.85994000
H	3.61281700	5.56529500	-2.14620300
H	5.32142900	5.89495800	-2.48182100
C	6.43636700	2.22421100	5.74834500
H	6.98376400	3.14827700	5.48104700
H	6.51000200	2.12425700	6.84866400
C	-3.07535200	4.22151000	-2.10356800
H	-3.18496600	4.11200800	-3.19949600
H	-3.65906000	3.40662600	-1.63954600
C	4.58482800	-5.46485900	3.19812500
H	5.44499100	-5.95277600	2.70104300
H	4.64309400	-5.74284700	4.26840500
C	6.74113100	-2.35734700	-5.38108100
H	6.89649300	-2.25391000	-6.47254000
H	7.24757100	-3.29453600	-5.08068500
C	-9.03352400	-3.49594900	1.67279900

H	-8.72804300	-2.77006600	2.44985400
H	-9.83411600	-3.00744700	1.08597800
C	1.61995400	0.36708900	-2.47368800
H	2.20249600	0.85364800	-1.67033500
H	2.14320700	-0.57519400	-2.70122900
C	-3.35909900	-4.03114100	1.76675100
H	-3.82036700	-3.12897800	1.33177500
H	-3.76962900	-4.88282000	1.19476400
C	-10.32416600	2.99847800	-1.01608400
H	-11.21753300	2.85620600	-1.64973100
H	-10.00674100	2.01000200	-0.64258700
H	-10.62287200	3.60746700	-0.14217500
C	1.40044500	-1.26179400	3.63652200
H	2.39317500	-1.48176300	4.06842100
H	0.78627300	-0.77812100	4.41556700
H	0.92145400	-2.22672700	3.38411600
C	4.05847700	5.85157100	-4.25975600
H	3.85060200	6.93572100	-4.25636600
H	4.87367700	5.67004200	-4.98493000
H	3.15519000	5.33899300	-4.64058900
C	7.07687100	1.01321500	5.06416200
H	8.12483500	0.88281600	5.38740900
H	6.53090400	0.08251500	5.30850400
H	7.07190500	1.12116600	3.96688300
C	-3.61403000	5.58103600	-1.65289500
H	-4.69217400	5.67617600	-1.87243600
H	-3.47854300	5.71522500	-0.56710900
H	-3.08898300	6.40879600	-2.16563100
C	3.26911300	-5.96379800	2.59389000
H	3.15841800	-7.05446400	2.72786000
H	2.40085500	-5.47508900	3.07475800
H	3.21415900	-5.74712800	1.51427200
C	7.35578300	-1.16399700	-4.64422800
H	8.42743700	-1.05567600	-4.88820100
H	6.85002800	-0.22022300	-4.92241100
H	7.26666300	-1.27656400	-3.55103200
C	-9.55065300	-4.78484100	2.31680800
H	-10.40952400	-4.58156100	2.97984000
H	-9.88292200	-5.51353600	1.55392600
H	-8.76783500	-5.27478400	2.92571600
C	1.58269700	1.25403700	-3.71820400
H	2.60112200	1.42827800	-4.10926600
H	0.98550000	0.78297700	-4.51781200
H	1.13071300	2.24049600	-3.50050300
C	-3.71208400	-4.15437200	3.25007000
H	-4.80535900	-4.22592400	3.38969900
H	-3.25732500	-5.05327000	3.70728400
H	-3.36306400	-3.27450000	3.82167700

1 4 2.0 5 1.0 7 1.5
2 3 1.5 8 1.5 18 1.0
3 4 1.5 22 1.0
4 6 1.0
5
6
7 8 1.0 12 1.5
8 9 1.5
9 10 2.0 13 1.0
10 11 1.5 14 1.0
11 12 1.5 21 1.0

12 15 1.0
13
14
15 16 1.0 17 1.0 93 1.0
16
17
18 19 1.0 20 1.0 30 1.0
19
20
21 23 1.0
22 26 1.0
23 24 1.0 25 1.0 126 1.0
24
25
26 27 1.0 28 1.0 129 1.0
27
28
29 32 2.0 33 1.0 36 1.5
30 31 1.5 35 1.5
31 32 1.5 42 1.0
32 34 1.0
33
34
35 36 1.0 40 1.5
36 37 1.5
37 38 1.5 44 1.0
38 39 1.5 43 1.0
39 40 2.0 41 1.0
40 125 1.0
41
42 107 1.0
43 104 1.0
44 45 1.0 46 1.0 50 1.0
45
46
47 48 2.0 51 1.0 53 1.5
48 49 1.5 52 1.0
49 50 1.5 61 1.0
50 54 1.5
51
52
53 54 1.5 58 1.5
54 55 1.5
55 56 2.0 59 1.0
56 57 1.5 60 1.0
57 58 1.5 62 1.0
58 65 1.0
59
60
61 101 1.0
62 110 1.0
63 65 1.0
64 65 1.0
65 67 1.0
66 69 2.0 70 1.0 73 1.5
67 68 1.5 72 1.5
68 69 1.5 80 1.0
69 71 1.0
70
71

72 73 1.0 77 1.5
73 74 1.5
74 75 1.5 82 1.0
75 76 1.5 81 1.0
76 77 2.0 78 1.0
77 79 1.0
78
79
80 113 1.0
81 122 1.0
82 83 1.0 84 1.0 88 1.0
83
84
85 86 2.0 89 1.0 92 1.5
86 87 1.5 90 1.0
87 88 1.5 99 1.0
88 91 1.5
89
90
91 92 1.0 96 1.5
92 93 1.5
93 94 1.5
94 95 1.5 100 1.0
95 96 2.0 97 1.0
96 98 1.0
97
98
99 119 1.0
100 116 1.0
101 102 1.0 103 1.0 132 1.0
102
103
104 105 1.0 106 1.0 135 1.0
105
106
107 108 1.0 109 1.0 138 1.0
108
109
110 111 1.0 112 1.0 141 1.0
111
112
113 114 1.0 115 1.0 144 1.0
114
115
116 117 1.0 118 1.0 147 1.0
117
118
119 120 1.0 121 1.0 150 1.0
120
121
122 123 1.0 124 1.0 153 1.0
123
124
125
126 127 1.0 128 1.0 156 1.0
127
128
129 130 1.0 131 1.0 160 1.0
130
131

132 133 1.0 134 1.0 164 1.0
133
134
135 136 1.0 137 1.0 168 1.0
136
137
138 139 1.0 140 1.0 172 1.0
139
140
141 142 1.0 143 1.0 176 1.0
142
143
144 145 1.0 146 1.0 180 1.0
145
146
147 148 1.0 149 1.0 184 1.0
148
149
150 151 1.0 152 1.0 188 1.0
151
152
153 154 1.0 155 1.0 192 1.0
154
155
156 157 1.0 158 1.0 159 1.0
157
158
159
160 161 1.0 162 1.0 163 1.0
161
162
163
164 165 1.0 166 1.0 167 1.0
165
166
167
168 169 1.0 170 1.0 171 1.0
169
170
171
172 173 1.0 174 1.0 175 1.0
173
174
175
176 177 1.0 178 1.0 179 1.0
177
178
179
180 181 1.0 182 1.0 183 1.0
181
182
183
184 185 1.0 186 1.0 187 1.0
185
186
187
188 189 1.0 190 1.0 191 1.0
189
190
191

192 193 1.0 194 1.0 195 1.0
193
194
195

DFT - optimized structures of Complexes

Atomic coordinates of **6⁺@ PrS[6]**^{nPr} complex

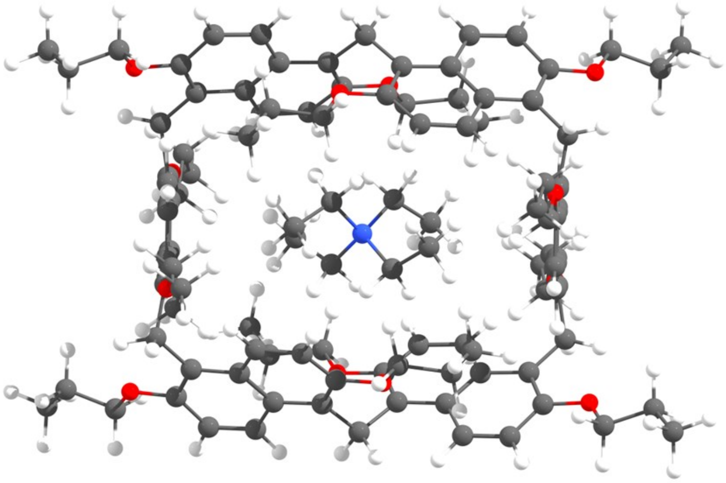

Figure S80. DFT-optimized structure (B97D3/SVP/SVPFIT) of the **6^{+} @ PrS[6]^{nPr}** complex.

C	-2.42573000	4.76421500	-0.35250000
C	-3.38067500	3.92345300	-1.04651200
C	-4.74849500	3.86331800	-0.62333200
C	-5.17132500	4.69923100	0.41883500
C	-4.25771400	5.58365200	1.05236600
C	-2.92605200	5.59678200	0.68896900
C	-1.04422500	4.76711500	-0.73980600
C	-0.65505000	4.02054700	-1.86178800
C	-1.60487800	3.25529400	-2.59032400
C	-2.92532300	3.19051800	-2.17812500
O	-6.47880100	4.60456100	0.80367800
C	-6.90540900	5.16898500	2.04244100
C	-8.31127200	4.66186100	2.33656700
C	-8.86401200	5.21718200	3.65173000
O	0.66995700	4.06680600	-2.20869300
C	1.10464200	3.48872000	-3.43495300
C	2.61379500	3.65996000	-3.55078400
C	3.17965100	2.96784400	-4.79258500
C	0.00015500	5.58779200	-0.00054700
H	-4.60013000	6.25239200	1.84615700
H	-2.25246000	6.26617100	1.22624200
H	-1.30528900	2.71635800	-3.49306100
H	-3.63083300	2.59500800	-2.75974000
H	-6.89374700	6.27725400	1.98288000
H	-6.20589100	4.87087000	2.85247200
H	-8.28537100	3.55598100	2.36141700
H	-8.96823000	4.94104100	1.49177200
H	-9.88207300	4.83765500	3.84436800
H	-8.23168800	4.92737500	4.51190900
H	-8.91761100	6.32142200	3.63475700
H	0.58320900	3.96993700	-4.28866600
H	0.84780400	2.40874400	-3.46694000
H	3.08810200	3.26305200	-2.63492300
H	2.85056800	4.73789600	-3.56763400
H	4.27081100	3.11903700	-4.86545400
H	2.99608700	1.87750200	-4.76680400
H	2.72743200	3.35992100	-5.72202300
C	2.42573300	-4.76428800	-0.35203200
C	3.38067500	-3.92361000	-1.04615500
C	4.74848400	-3.86337200	-0.62294700
C	5.17130900	-4.69912700	0.41934600
C	4.25771200	-5.58349000	1.05297700
C	2.92605600	-5.59670100	0.68956200
C	1.04423400	-4.76726500	-0.73935200
C	0.65506500	-4.02087100	-1.86145500
C	1.60489900	-3.25574600	-2.59011500

C	2.92533600	-3.19088000	-2.17790200
O	6.47877700	-4.60437100	0.80420800
C	6.90535900	-5.16858400	2.04307700
C	8.31118400	-4.66134800	2.33718600
C	8.86389300	-5.21644000	3.65246000
O	-0.66993900	-4.06717100	-2.20835600
C	-1.10464700	-3.48918400	-3.43465000
C	-2.61381400	-3.66036700	-3.55037800
C	-3.17972400	-2.96830700	-4.79218300
C	-0.00015300	-5.58785200	0.00000400
H	4.60013300	-6.25211100	1.84686600
H	2.25246300	-6.26601600	1.22692400
H	1.30531700	-2.71698100	-3.49295600
H	3.63084300	-2.59545000	-2.75960200
H	6.89375200	-6.27686300	1.98368500
H	6.20579200	-4.87037900	2.85303200
H	8.28523100	-3.55546500	2.36186500
H	8.96819400	-4.94062700	1.49246400
H	9.88192900	-4.83683800	3.84508300
H	8.23152000	-4.92652700	4.51256600
H	8.91754000	-6.32068000	3.63566100
H	-0.58328000	-3.97051500	-4.28834000
H	-0.84777600	-2.40921800	-3.46678300
H	-3.08801500	-3.26337200	-2.63450200
H	-2.85064100	-4.73829300	-3.56713500
H	-4.27089400	-3.11946000	-4.86497300
H	-2.99611100	-1.87797100	-4.76648200
H	-2.72758000	-3.36046300	-5.72162400
C	-5.48250700	0.72507000	-0.00984500
C	-5.48259900	-0.72503200	0.00993700
C	-5.51633100	-1.44514600	1.25230500
C	-5.46373100	-0.71774100	2.45078300
C	-5.45824100	0.70319500	2.43954300
C	-5.49324600	1.39924800	1.24480900
C	-5.51632100	1.44518600	-1.25220700
C	-5.46394600	0.71777800	-2.45069500
C	-5.45864000	-0.70315600	-2.43945600
C	-5.49359800	-1.39920300	-1.24471600
O	-5.43511800	-1.43873600	3.61462300
C	-5.27946200	-0.77706700	4.86604300
C	-5.15547500	-1.83409500	5.95554500
C	-4.97795500	-1.21304800	7.34362800
O	-5.43542800	1.43877700	-3.61453300
C	-5.27972400	0.77712300	-4.86595600
C	-5.15558000	1.83416200	-5.95542900
C	-4.97799700	1.21312300	-7.34350800
C	-5.75015600	2.95006800	-1.30568900
H	-5.45207500	1.26280700	3.37781100
H	-5.53457700	2.48546300	1.27330900
H	-5.45267400	-1.26276900	-3.37772600
H	-5.53511500	-2.48540700	-1.27322000
H	-6.15007300	-0.11641900	5.06031400
H	-4.37442200	-0.13388900	4.84454300
H	-4.29928800	-2.49191500	5.71252800
H	-6.05727500	-2.47363300	5.92946400
H	-4.88786500	-1.99361700	8.11828400
H	-4.06824400	-0.58554100	7.39391600
H	-5.83860300	-0.57368300	7.61371300
H	-6.15036700	0.11654100	-5.06031200
H	-4.37473500	0.13388000	-4.84439200
H	-4.29936600	2.49191800	-5.71233500
H	-6.05733700	2.47376200	-5.92940800
H	-4.88779400	1.99369700	-8.11814700
H	-4.06832500	0.58555400	-7.39373400

H	-5.83866700	0.57382000	-7.61366800
C	5.48251200	-0.72506300	-0.00980200
C	5.48257400	0.72504000	0.00983300
C	5.51638000	1.44528400	1.25212300
C	5.46386700	0.71800200	2.45068000
C	5.45840200	-0.70293500	2.43958400
C	5.49334000	-1.39911300	1.24491800
C	5.51627700	-1.44530400	-1.25209300
C	5.46383100	-0.71801700	-2.45065300
C	5.45847300	0.70291900	-2.43955600
C	5.49347000	1.39908600	-1.24488800
O	5.43534200	1.43911300	3.61445000
C	5.27958100	0.77758600	4.86593200
C	5.15552200	1.83473500	5.95530800
C	4.97790500	1.21383900	7.34344600
O	5.43527700	-1.43912700	-3.61442200
C	5.27967900	-0.77758600	-4.86591600
C	5.15561600	-1.83472300	-5.95530400
C	4.97814300	-1.21380900	-7.34345300
C	5.75013200	-2.95018800	-1.30541500
H	5.45231900	-1.26245000	3.37790900
H	5.53472500	-2.48532400	1.27353000
H	5.45242500	1.26243800	-3.37788000
H	5.53493000	2.48529100	-1.27349900
H	6.15017100	0.11695100	5.06034600
H	4.37453900	0.13441300	4.84443100
H	4.29935400	2.49253200	5.71216100
H	6.05732600	2.47426700	5.92921900
H	4.88776600	1.99449200	8.11801200
H	4.06818900	0.58634100	7.39374000
H	5.83853200	0.57450000	7.61366100
H	6.15034400	-0.11702900	-5.06025600
H	4.37469300	-0.13433300	-4.84449000
H	4.29937900	-2.49245300	-5.71221700
H	6.05736800	-2.47432500	-5.92915400
H	4.88800100	-1.99445300	-8.11802800
H	4.06847700	-0.58624300	-7.39380800
H	5.83883700	-0.57453300	-7.61360300
C	-3.38088000	-3.92355200	1.04640200
C	-2.42599500	-4.76421100	0.35217900
C	-1.04448100	-4.76726600	0.73944500
C	-0.65524400	-4.02097300	1.86159200
C	-1.60501400	-3.25584300	2.59032700
C	-2.92546300	-3.19089100	2.17816400
C	-4.74871600	-3.86327900	0.62329100
C	-5.17163700	-4.69902700	-0.41896900
C	-4.25808000	-5.58333000	-1.05274100
C	-2.92639100	-5.59654200	-0.68944500
O	0.66976200	-4.06741200	2.20846900
C	1.10459000	-3.48934600	3.43467900
C	2.61376500	-3.66054700	3.55028000
C	3.17981700	-2.96839100	4.79196700
O	-6.47914600	-4.60428300	-0.80368700
C	-6.90588700	-5.16856900	-2.04246800
C	-8.31180100	-4.66145100	-2.33635800
C	-8.86469200	-5.21663100	-3.65151800
C	-5.75026900	-2.95001600	1.30579300
H	-1.30538100	-2.71715100	3.49319300
H	-3.63092200	-2.59546600	2.75992900
H	-4.60055600	-6.25190500	-1.84664600
H	-2.25283300	-6.26580800	-1.22691100
H	0.58329700	-3.97060100	4.28845700
H	0.84772000	-2.40937900	3.46674300
H	3.08789900	-3.26369400	2.63430600

H	2.85055000	-4.73848000	3.56714000
H	4.27098100	-3.11961900	4.86468900
H	2.99628600	-1.87804400	4.76617400
H	2.72771600	-3.36041900	5.72148300
H	-6.89418200	-6.27684500	-1.98304000
H	-6.20647800	-4.87033500	-2.85255000
H	-8.28593500	-3.55556700	-2.36108100
H	-8.96864500	-4.94075000	-1.49151300
H	-9.88278700	-4.83710700	-3.84398300
H	-8.23248400	-4.92670500	-4.51174100
H	-8.91826000	-6.32087400	-3.63466900
C	3.38091900	3.92366300	1.04599300
C	2.42601300	4.76422100	0.35167800
C	1.04450500	4.76730400	0.73896800
C	0.65528900	4.02111600	1.86119100
C	1.60508400	3.25611200	2.59002400
C	2.92553300	3.19115100	2.17786600
C	4.74874300	3.86333700	0.62285000
C	5.17162800	4.69893100	-0.41954800
C	4.25805300	5.58315000	-1.05341200
C	2.92637700	5.59641400	-0.69007200
O	-0.66973200	4.06748600	2.20802800
C	-1.10446600	3.48994500	3.43452600
C	-2.61362500	3.66127300	3.55017900
C	-3.17968400	2.96935800	4.79199700
O	6.47912100	4.60412300	-0.80430700
C	6.90578600	5.16812500	-2.04324500
C	8.31165000	4.66087700	-2.33714700
C	8.86445800	5.21575600	-3.65246900
C	5.75031900	2.95015900	1.30543200
H	1.30546100	2.71752400	3.49295600
H	3.63101600	2.59582200	2.75970100
H	4.60050800	6.25161700	-1.84741600
H	2.25280100	6.26560600	-1.22760900
H	-0.58305700	3.97152800	4.28804600
H	-0.84765500	2.40997400	3.46702900
H	-3.08781700	3.26429700	2.63428900
H	-2.85034400	4.73922400	3.56686400
H	-4.27082900	3.12070900	4.86475300
H	-2.99626700	1.87898700	4.76634800
H	-2.72749700	3.36146800	5.72143700
H	6.89414100	6.27641500	-1.98405100
H	6.20629300	4.86975400	-2.85320300
H	8.28572800	3.55498900	-2.36163800
H	8.96858000	4.94032000	-1.49241600
H	9.88251800	4.83614400	-3.84494100
H	8.23216400	4.92568100	-4.51257900
H	8.91807900	6.32000000	-3.63585500
H	-5.82890700	-3.24139700	2.36463700
H	-6.73800500	-3.14483100	0.85837600
H	-6.73783600	3.14494900	-0.85817600
H	-5.82888200	3.24143100	-2.36453200
H	-0.49138000	6.24855900	0.72670600
H	0.49171100	6.24833800	-0.72798700
H	5.82900400	3.24166600	2.36423800
H	6.73803600	3.14491800	0.85794700
H	6.73781500	-3.14501000	-0.85788500
H	5.82885700	-3.24166900	-2.36422500
H	0.49137700	-6.24856100	0.72731300
H	-0.49173100	-6.24845700	-0.72736700
C	-1.89941600	-0.24664300	-1.72925600
H	-1.44728800	0.36870500	-2.52231900
H	-2.60843900	0.38064700	-1.16837800
C	-0.86621100	-0.92650500	-0.85200000

H	-2.50067900	-1.03443000	-2.20841500
H	-1.34342600	-1.62394400	-0.14770300
H	-0.15651300	-1.51993600	-1.44284900
N	-0.00001400	-0.00003900	0.00054600
C	0.86641800	-0.92605000	0.85332800
C	0.86604900	0.92618500	-0.85237500
C	-0.86632800	0.92620500	0.85319400
H	1.34313400	1.62402600	-0.14838700
C	1.89939400	0.24615400	-1.72933400
H	0.15627400	1.51923000	-1.44351800
H	2.50014900	1.03393000	-2.20914400
H	2.60882600	-0.38040400	-1.16815500
H	1.44740500	-0.36995300	-2.52188200
C	1.89928300	-0.24565300	1.73056500
H	0.15689300	-1.51961500	1.44424700
H	1.34393100	-1.62345800	0.14919900
H	2.50070600	-1.03309500	2.21008900
H	2.60819200	0.38171600	1.16963400
H	1.44681900	0.36977700	2.52337100
H	-0.15672900	1.51979900	1.44399000
H	-1.34379200	1.62355100	0.14897100
C	-1.89924500	0.24606600	1.73056800
H	-2.50038600	1.03367200	2.21017600
H	-2.60839700	-0.38110700	1.16972400
H	-1.44683500	-0.36948300	2.52331400

1 2 1.0 6 1.5 7 1.5
2 3 1.5 10 1.5
3 4 1.5 93 1.0
4 5 1.5 11 1.0
5 6 2.0 20 1.0
6 21 1.0
7 8 1.5 19 1.0
8 9 1.5 15 1.0
9 10 2.0 22 1.0
10 23 1.0
11 12 1.0
12 13 1.0 24 1.0 25 1.0
13 14 1.0 26 1.0 27 1.0
14 28 1.0 29 1.0 30 1.0
15 16 1.0
16 17 1.0 31 1.0 32 1.0
17 18 1.0 33 1.0 34 1.0
18 35 1.0 36 1.0 37 1.0
19 188 1.0 227 1.0 228 1.0
20
21
22
23
24
25
26
27
28
29
30
31
32
33
34
35
36
37
38 39 1.0 43 1.5 44 1.5

39 40 1.5 47 1.5
40 41 1.5 130 1.0
41 42 1.5 48 1.0
42 43 2.0 57 1.0
43 58 1.0
44 45 1.5 56 1.0
45 46 1.5 52 1.0
46 47 2.0 59 1.0
47 60 1.0
48 49 1.0
49 50 1.0 61 1.0 62 1.0
50 51 1.0 63 1.0 64 1.0
51 65 1.0 66 1.0 67 1.0
52 53 1.0
53 54 1.0 68 1.0 69 1.0
54 55 1.0 70 1.0 71 1.0
55 72 1.0 73 1.0 74 1.0
56 151 1.0 233 1.0 234 1.0
57
58
59
60
61
62
63
64
65
66
67
68
69
70
71
72
73
74
75 76 1.0 80 1.5 81 1.5
76 77 1.5 84 1.5
77 78 1.5 167 1.0
78 79 1.5 85 1.0
79 80 2.0 94 1.0
80 95 1.0
81 82 1.5 93 1.0
82 83 1.5 89 1.0
83 84 2.0 96 1.0
84 97 1.0
85 86 1.0
86 87 1.0 98 1.0 99 1.0
87 88 1.0 100 1.0 101 1.0
88 102 1.0 103 1.0 104 1.0
89 90 1.0
90 91 1.0 105 1.0 106 1.0
91 92 1.0 107 1.0 108 1.0
92 109 1.0 110 1.0 111 1.0
93 225 1.0 226 1.0
94
95
96
97
98
99
100
101
102

103
104
105
106
107
108
109
110
111
112 113 1.0 117 1.5 118 1.5
113 114 1.5 121 1.5
114 115 1.5 204 1.0
115 116 1.5 122 1.0
116 117 2.0 131 1.0
117 132 1.0
118 119 1.5 130 1.0
119 120 1.5 126 1.0
120 121 2.0 133 1.0
121 134 1.0
122 123 1.0
123 124 1.0 135 1.0 136 1.0
124 125 1.0 137 1.0 138 1.0
125 139 1.0 140 1.0 141 1.0
126 127 1.0
127 128 1.0 142 1.0 143 1.0
128 129 1.0 144 1.0 145 1.0
129 146 1.0 147 1.0 148 1.0
130 231 1.0 232 1.0
131
132
133
134
135
136
137
138
139
140
141
142
143
144
145
146
147
148
149 150 1.0 154 1.5 155 1.5
150 151 1.5 158 1.5
151 152 1.5
152 153 1.5 159 1.0
153 154 2.0 168 1.0
154 169 1.0
155 156 1.5 167 1.0
156 157 1.5 163 1.0
157 158 2.0 170 1.0
158 171 1.0
159 160 1.0
160 161 1.0 172 1.0 173 1.0
161 162 1.0 174 1.0 175 1.0
162 176 1.0 177 1.0 178 1.0
163 164 1.0
164 165 1.0 179 1.0 180 1.0
165 166 1.0 181 1.0 182 1.0
166 183 1.0 184 1.0 185 1.0

167 223 1.0 224 1.0
168
169
170
171
172
173
174
175
176
177
178
179
180
181
182
183
184
185
186 187 1.0 191 1.5 192 1.5
187 188 1.5 195 1.5
188 189 1.5
189 190 1.5 196 1.0
190 191 2.0 205 1.0
191 206 1.0
192 193 1.5 204 1.0
193 194 1.5 200 1.0
194 195 2.0 207 1.0
195 208 1.0
196 197 1.0
197 198 1.0 209 1.0 210 1.0
198 199 1.0 211 1.0 212 1.0
199 213 1.0 214 1.0 215 1.0
200 201 1.0
201 202 1.0 216 1.0 217 1.0
202 203 1.0 218 1.0 219 1.0
203 220 1.0 221 1.0 222 1.0
204 229 1.0 230 1.0
205
206
207
208
209
210
211
212
213
214
215
216
217
218
219
220
221
222
223
224
225
226
227
228
229
230

231
232
233
234
235 236 1.0 237 1.0 238 1.0 239 1.0
236
237
238 240 1.0 241 1.0 242 1.0
239
240
241
242 243 1.0 244 1.0 245 1.0
243 252 1.0 253 1.0 254 1.0
244 246 1.0 247 1.0 248 1.0
245 258 1.0 259 1.0 260 1.0
246
247 249 1.0 250 1.0 251 1.0
248
249
250
251
252 255 1.0 256 1.0 257 1.0
253
254
255
256
257
258
259
260 261 1.0 262 1.0 263 1.0
261
262
263

---

0 imaginary frequency

Atomic coordinates of **7^{2+}@ PrS[6]^{nPr}** complex

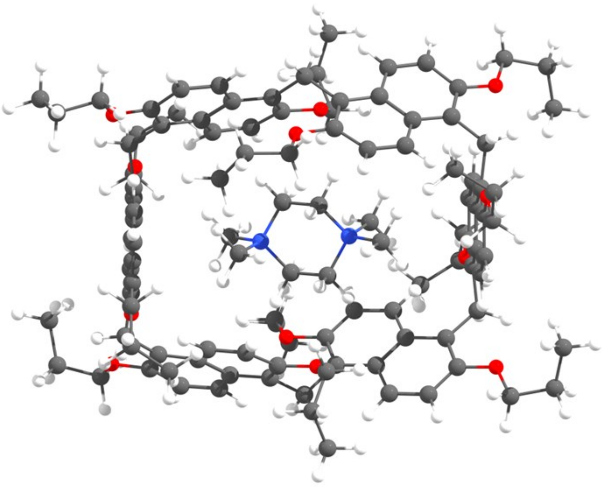

Figure S81. DFT-optimized structure (B97D3/SVP/SVPFIT) of the **7^{2+} @ PrS[6]^{nPr}** complex.

C	2.05588400	4.71380900	-0.00270700
C	3.11276200	4.01664900	0.70669800
C	4.46371600	4.04381600	0.23022100
C	4.76453900	4.81395500	-0.90462300
C	3.74393800	5.54482900	-1.57141400
C	2.43400700	5.48321200	-1.13949800

C	0.69856400	4.65134200	0.46823100
C	0.42613900	3.94468800	1.64871600
C	1.47154800	3.33116900	2.39059800
C	2.77424400	3.35661600	1.92443200
O	6.05413900	4.80264300	-1.33446400
C	6.44776600	5.53311200	-2.50237000
C	7.92736100	5.27315300	-2.74821200
C	8.44530800	6.01470700	-3.98370900
O	-0.89172300	3.83357100	2.00820200
C	-1.26774700	3.53550300	3.35790800
C	-0.86012000	4.60063800	4.37736400
C	-1.36205600	6.00715800	4.04384000
C	-0.42598100	5.41339600	-0.22004000
H	3.98679700	6.15720700	-2.44299700
H	1.68125300	6.03946300	-1.69901500
H	1.25826800	2.83462300	3.34157800
H	3.55863900	2.89530500	2.52602200
H	6.25677300	6.61431900	-2.34833700
H	5.84159500	5.20308500	-3.37198400
H	8.07934600	4.18240000	-2.85714100
H	8.49112300	5.58026500	-1.84785800
H	9.51794300	5.81219600	-4.14199800
H	7.90825500	5.70430400	-4.89925300
H	8.32510500	7.10848000	-3.87932600
H	-0.87526200	2.53864900	3.65455100
H	-2.36789600	3.45582800	3.30433400
H	0.23958100	4.61428000	4.48959800
H	-1.26134800	4.27266100	5.35609800
H	-1.09158000	6.71899800	4.84191600
H	-0.91558300	6.37748600	3.10480600
H	-2.46088100	6.02703300	3.93013100
C	-2.11077400	-4.64921200	0.17694000
C	-3.16388400	-3.90403400	0.84054800
C	-4.51227200	-3.93429100	0.35271900
C	-4.80608500	-4.72642000	-0.76889400
C	-3.77674200	-5.45652100	-1.42667100
C	-2.47476000	-5.41267800	-0.96891100
C	-0.77051100	-4.63321000	0.69570900
C	-0.50243000	-3.89560700	1.85760000
C	-1.53266900	-3.17842100	2.52082900
C	-2.82531800	-3.18665400	2.02501900
O	-6.09723600	-4.74358500	-1.19431100
C	-6.49491600	-5.54950400	-2.31346900
C	-7.98396400	-5.34221300	-2.55247900
C	-8.36221900	-3.91182600	-2.94745700
O	0.80187900	-3.86765700	2.27538300
C	1.13901900	-3.49658900	3.61367200
C	0.70077200	-4.52398400	4.65542000
C	1.08755100	-4.10393900	6.07591000
C	0.35531500	-5.44461600	0.07197400
H	-4.00982600	-6.07088500	-2.29979700
H	-1.71930600	-5.98470500	-1.50563400
H	-1.32670600	-2.64412800	3.45265300
H	-3.60264500	-2.66927500	2.58702600
H	-6.27281300	-6.61268900	-2.09607800
H	-5.91128900	-5.25032600	-3.20912800
H	-8.53300600	-5.64865800	-1.64243600
H	-8.28283400	-6.05035000	-3.34837400
H	-9.44077800	-3.83665900	-3.16829500
H	-8.13319800	-3.19564800	-2.14033500
H	-7.81227700	-3.58924100	-3.85225400
H	0.73153400	-2.49066800	3.85502200
H	2.23963800	-3.40690000	3.59747200
H	1.16362300	-5.49472300	4.39695400

H	-0.39270900	-4.67319700	4.58806000
H	0.78717000	-4.87012100	6.81018800
H	2.17935100	-3.95875400	6.17357400
H	0.59558400	-3.15622300	6.36501100
C	5.61523500	0.95980000	-0.04670400
C	5.76693600	-0.47498500	0.10377400
C	5.89202900	-1.33029200	-1.04336900
C	5.83781700	-0.75300900	-2.32252700
C	5.68879100	0.65313200	-2.47570200
C	5.59641700	1.48236500	-1.37187400
C	5.53859300	1.81963000	1.10219400
C	5.56156600	1.23837400	2.38161000
C	5.72142400	-0.16680900	2.53505600
C	5.84074600	-0.99086200	1.42975400
O	5.93792000	-1.59645900	-3.39144100
C	5.93448000	-1.08289000	-4.72810200
C	6.02475200	-2.25045200	-5.70145400
C	4.82412900	-3.20057800	-5.66116800
O	5.43582100	2.08099100	3.44827500
C	5.49821300	1.57799000	4.78726000
C	5.28133500	2.73481800	5.75342100
C	3.90149500	3.39185300	5.64946600
C	5.58468900	3.33677400	0.97309200
H	5.67818400	1.09915100	-3.47295300
H	5.53824500	2.55732800	-1.53082400
H	5.79638500	-0.60834400	3.53184700
H	6.02744200	-2.05065900	1.59147500
H	6.79202700	-0.39397100	-4.86035200
H	5.00130900	-0.50508500	-4.90133500
H	6.96031200	-2.80544400	-5.50179600
H	6.13192300	-1.81625900	-6.71351800
H	4.90583600	-3.97147500	-6.44658700
H	4.75010800	-3.71968300	-4.69075500
H	3.87606600	-2.65314500	-5.82764200
H	6.48384600	1.10149300	4.95737600
H	4.71471800	0.80297100	4.92953700
H	6.07671100	3.48574700	5.59032200
H	5.43680400	2.33492400	6.77327700
H	3.75852500	3.87964900	4.67056100
H	3.09320500	2.64612000	5.78006200
H	3.77264100	4.16228700	6.42904800
C	-5.72227400	-0.88677500	-0.04346300
C	-5.83033300	0.55653300	0.04222900
C	-6.01781200	1.35444500	-1.13661400
C	-6.05325100	0.71345200	-2.38648700
C	-5.95842200	-0.70297500	-2.47546700
C	-5.81427900	-1.47706100	-1.33679200
C	-5.58674600	-1.68897900	1.14095800
C	-5.53393900	-1.04604900	2.38897400
C	-5.63919000	0.36946300	2.47876100
C	-5.79935400	1.14031500	1.34168200
O	-6.19035800	1.50863400	-3.48619000
C	-6.28549200	0.93583500	-4.79500100
C	-6.38770500	2.06385600	-5.81261900
C	-5.15573600	2.97199100	-5.86858400
O	-5.38678900	-1.83799400	3.49241300
C	-5.38885600	-1.26668300	4.80542900
C	-5.18776700	-2.38024900	5.82457900
C	-3.83398800	-3.09027700	5.72649000
C	-5.63446500	-3.21027000	1.07992000
H	-6.03978600	-1.20165800	-3.44440500
H	-5.80800100	-2.56041000	-1.44301200
H	-5.63876100	0.86286400	3.45375600
H	-5.93870700	2.21228500	1.45831000

H	-7.17412700	0.27597800	-4.84559500
H	-5.38603600	0.31465400	-4.99427000
H	-7.29342900	2.65867900	-5.59110800
H	-6.55935500	1.59276500	-6.79895700
H	-5.01482300	3.52153200	-4.92244400
H	-4.23629700	2.38615800	-6.06227500
H	-5.25210200	3.71760000	-6.67635200
H	-6.35035500	-0.74429000	4.97842700
H	-4.57288900	-0.51677000	4.88550800
H	-6.01167900	-3.11018900	5.71760800
H	-5.30604300	-1.92383100	6.82553600
H	-3.71482800	-3.82473600	6.54162000
H	-3.72894800	-3.63097300	4.77100900
H	-2.99766400	-2.36816300	5.80308500
C	3.81606000	-3.81340300	-0.76305600
C	2.79101300	-4.56515700	-0.06596600
C	1.46162000	-4.65067000	-0.60776400
C	1.18896800	-4.02802200	-1.83460000
C	2.21255100	-3.36500800	-2.56041000
C	3.48861200	-3.26011900	-2.03535500
C	5.12905200	-3.66462500	-0.20863900
C	5.44170500	-4.34512300	0.97837500
C	4.45774600	-5.13397500	1.63743700
C	3.17300000	-5.22440600	1.13761100
O	-0.11118600	-4.06522100	-2.27227400
C	-0.41170100	-3.98770600	-3.67330400
C	0.15940900	-5.14143700	-4.49757000
C	-0.26188500	-6.52632500	-4.00107900
O	6.69987600	-4.18689200	1.46899600
C	7.09976400	-4.82126500	2.69197900
C	8.52484900	-4.39244300	3.01255400
C	8.68097700	-2.89220600	3.27724800
C	6.18607000	-2.81884300	-0.89954200
H	2.00567200	-2.93685200	-3.54542100
H	4.25869000	-2.76070300	-2.62418300
H	4.70950400	-5.67536700	2.55260700
H	2.44566200	-5.82091100	1.68933400
H	-0.08287900	-3.00962800	-4.08430700
H	-1.51569200	-4.00487200	-3.69957500
H	1.26252900	-5.07165800	-4.52436600
H	-0.18076200	-4.98746200	-5.53996400
H	0.12331300	-7.31652100	-4.66735500
H	0.13333500	-6.72582700	-2.99003500
H	-1.36228800	-6.62436200	-3.96324000
H	7.03335800	-5.92075000	2.57430100
H	6.40912500	-4.51864200	3.50697000
H	9.18303700	-4.70740800	2.18124900
H	8.84441600	-4.97295300	3.89860100
H	9.71887600	-2.64799900	3.56156500
H	8.42719200	-2.29606500	2.38435000
H	8.02132900	-2.56165500	4.10233200
C	-3.91034000	3.80818300	-0.98197700
C	-2.86899100	4.57227000	-0.32595300
C	-1.53774800	4.60222900	-0.86686600
C	-1.28199600	3.92859700	-2.07100600
C	-2.32625300	3.26307500	-2.76880400
C	-3.59895000	3.19166300	-2.22798300
C	-5.22333600	3.71668100	-0.41789700
C	-5.52048400	4.46226900	0.73272300
C	-4.52012500	5.26547200	1.34711600
C	-3.23538100	5.30061500	0.84155300
O	0.01578600	3.92034900	-2.51910000
C	0.28439100	3.53669500	-3.87106100
C	1.77270700	3.69293600	-4.15025800

C	2.66164500	2.74755200	-3.34110800
O	-6.77770000	4.34547100	1.23694900
C	-7.14894700	5.00975100	2.45221600
C	-8.55927700	4.57336900	2.82430800
C	-8.68551000	3.07867400	3.13312000
C	-6.29503100	2.85024400	-1.05421500
H	-2.14124000	2.80311900	-3.74294500
H	-4.38292300	2.68599300	-2.79422100
H	-4.75787100	5.86040900	2.23244200
H	-2.49684600	5.90906000	1.36126700
H	-0.31740900	4.16171100	-4.55986500
H	-0.01803700	2.47798300	-4.03286700
H	2.06085500	4.74148600	-3.95879300
H	1.92069600	3.52044000	-5.23301400
H	3.72834300	2.91732700	-3.56104800
H	2.52807000	2.90463400	-2.25798700
H	2.43970500	1.68945800	-3.57903500
H	-7.09763000	6.10643300	2.30338500
H	-6.43288300	4.73679300	3.25567700
H	-9.24387800	4.85612400	2.00279600
H	-8.86318900	5.17373400	3.70258700
H	-9.71382200	2.82486400	3.44288100
H	-8.43610500	2.46291100	2.25227200
H	-8.00529800	2.78208000	3.95438600
H	6.37396100	-3.22736800	-1.90447700
H	7.12843700	-2.93145200	-0.34134000
H	6.53091800	3.59711600	0.47322300
H	5.64763000	3.75758300	1.98870400
H	-0.00126600	6.06154500	-0.99793100
H	-0.86300000	6.09448100	0.52395200
H	-6.49905200	3.21587600	-2.07237800
H	-7.22720700	2.99236400	-0.48491300
H	-6.58393600	-3.50079100	0.60406300
H	-5.67950700	-3.58735100	2.11346000
H	-0.06279500	-6.14092400	-0.66768300
H	0.78905800	-6.07236600	0.86288300
C	-0.69979700	-1.34563600	-0.51788900
N	-1.51957000	-0.06685100	-0.47386500
C	-0.58203800	1.09847300	-0.72411600
C	0.61250100	1.12102100	0.20643100
N	1.43878700	-0.14801400	0.17368600
C	0.50584000	-1.32458000	0.39761000
H	-0.41168700	-1.50345100	-1.56660300
H	-1.34868300	-2.18603900	-0.23007400
H	-1.14440200	2.03476400	-0.58897500
H	-0.28206600	1.04823800	-1.78027600
H	1.26069900	1.96612500	-0.06709900
H	0.30831300	1.27668200	1.24851100
H	0.19984800	-1.28078600	1.45111300
H	1.07685300	-2.25539300	0.25880600
C	-2.25532200	0.10490400	0.83509100
H	-2.79800800	1.05995800	0.79657100
H	-2.96487100	-0.72625700	0.94847000
H	-1.54368600	0.10147600	1.66920300
C	-2.56021500	-0.13144000	-1.56630800
H	-3.15665000	0.79043100	-1.53835100
H	-2.04760200	-0.22163200	-2.53436200
H	-3.20393400	-1.00220300	-1.38035900
C	2.20846200	-0.25663100	-1.11648100
H	2.80204800	-1.17900100	-1.09305300
H	1.51439500	-0.29170700	-1.96457100
H	2.87192200	0.61531200	-1.19706700
C	2.44771600	-0.12808700	1.29738800
H	3.00945800	-1.07131500	1.27000400

H	3.12966700	0.71917800	1.14536800
H	1.91200700	-0.01750900	2.25017700

1 2 1.0 6 1.5 7 1.5
2 3 1.5 10 1.5
3 4 1.5 93 1.0
4 5 1.5 11 1.0
5 6 2.0 20 1.0
6 21 1.0
7 8 1.5 19 1.0
8 9 1.5 15 1.0
9 10 2.0 22 1.0
10 23 1.0
11 12 1.0
12 13 1.0 24 1.0 25 1.0
13 14 1.0 26 1.0 27 1.0
14 28 1.0 29 1.0 30 1.0
15 16 1.0
16 17 1.0 31 1.0 32 1.0
17 18 1.0 33 1.0 34 1.0
18 35 1.0 36 1.0 37 1.0
19 188 1.0 227 1.0 228 1.0
20
21
22
23
24
25
26
27
28
29
30
31
32
33
34
35
36
37
38 39 1.0 43 1.5 44 1.5
39 40 1.5 47 1.5
40 41 1.5 130 1.0
41 42 1.5 48 1.0
42 43 2.0 57 1.0
43 58 1.0
44 45 1.5 56 1.0
45 46 1.5 52 1.0
46 47 2.0 59 1.0
47 60 1.0
48 49 1.0
49 50 1.0 61 1.0 62 1.0
50 51 1.0 63 1.0 64 1.0
51 65 1.0 66 1.0 67 1.0
52 53 1.0
53 54 1.0 68 1.0 69 1.0
54 55 1.0 70 1.0 71 1.0
55 72 1.0 73 1.0 74 1.0
56 151 1.0 233 1.0 234 1.0
57
58
59
60
61

62
63
64
65
66
67
68
69
70
71
72
73
74
75 76 1.0 80 1.5 81 1.5
76 77 1.5 84 1.5
77 78 1.5 167 1.0
78 79 1.5 85 1.0
79 80 2.0 94 1.0
80 95 1.0
81 82 1.5 93 1.0
82 83 1.5 89 1.0
83 84 2.0 96 1.0
84 97 1.0
85 86 1.0
86 87 1.0 98 1.0 99 1.0
87 88 1.0 100 1.0 101 1.0
88 102 1.0 103 1.0 104 1.0
89 90 1.0
90 91 1.0 105 1.0 106 1.0
91 92 1.0 107 1.0 108 1.0
92 109 1.0 110 1.0 111 1.0
93 225 1.0 226 1.0
94
95
96
97
98
99
100
101
102
103
104
105
106
107
108
109
110
111
112 113 1.0 117 1.5 118 1.5
113 114 1.5 121 1.5
114 115 1.5 204 1.0
115 116 1.5 122 1.0
116 117 2.0 131 1.0
117 132 1.0
118 119 1.5 130 1.0
119 120 1.5 126 1.0
120 121 2.0 133 1.0
121 134 1.0
122 123 1.0
123 124 1.0 135 1.0 136 1.0
124 125 1.0 137 1.0 138 1.0
125 139 1.0 140 1.0 141 1.0

126 127 1.0
127 128 1.0 142 1.0 143 1.0
128 129 1.0 144 1.0 145 1.0
129 146 1.0 147 1.0 148 1.0
130 231 1.0 232 1.0
131
132
133
134
135
136
137
138
139
140
141
142
143
144
145
146
147
148
149 150 1.0 154 1.5 155 1.5
150 151 1.5 158 1.5
151 152 1.5
152 153 1.5 159 1.0
153 154 2.0 168 1.0
154 169 1.0
155 156 1.5 167 1.0
156 157 1.5 163 1.0
157 158 2.0 170 1.0
158 171 1.0
159 160 1.0
160 161 1.0 172 1.0 173 1.0
161 162 1.0 174 1.0 175 1.0
162 176 1.0 177 1.0 178 1.0
163 164 1.0
164 165 1.0 179 1.0 180 1.0
165 166 1.0 181 1.0 182 1.0
166 183 1.0 184 1.0 185 1.0
167 223 1.0 224 1.0
168
169
170
171
172
173
174
175
176
177
178
179
180
181
182
183
184
185
186 187 1.0 191 1.5 192 1.5
187 188 1.5 195 1.5
188 189 1.5
189 190 1.5 196 1.0

190 191 2.0 205 1.0
191 206 1.0
192 193 1.5 204 1.0
193 194 1.5 200 1.0
194 195 2.0 207 1.0
195 208 1.0
196 197 1.0
197 198 1.0 209 1.0 210 1.0
198 199 1.0 211 1.0 212 1.0
199 213 1.0 214 1.0 215 1.0
200 201 1.0
201 202 1.0 216 1.0 217 1.0
202 203 1.0 218 1.0 219 1.0
203 220 1.0 221 1.0 222 1.0
204 229 1.0 230 1.0
205
206
207
208
209
210
211
212
213
214
215
216
217
218
219
220
221
222
223
224
225
226
227
228
229
230
231
232
233
234
235 236 1.0 240 1.0 241 1.0 242 1.0
236 237 1.0 249 1.0 253 1.0
237 238 1.0 243 1.0 244 1.0
238 239 1.0 245 1.0 246 1.0
239 240 1.0 257 1.0 261 1.0
240 247 1.0 248 1.0
241
242
243
244
245
246
247
248
249 250 1.0 251 1.0 252 1.0
250
251
252
253 254 1.0 255 1.0 256 1.0

254
255
256
257 258 1.0 259 1.0 260 1.0
258
259
260
261 262 1.0 263 1.0 264 1.0
262
263
264

0 imaginary frequency

Atomic coordinates of **5^{2+}@ PrS[6]*^{nPr}*** complex.

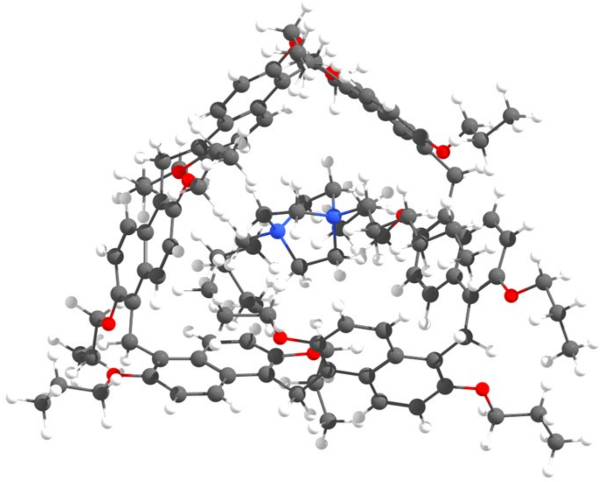

Figure S82. DFT-optimized structure (B97D3/SVP/SVPFIT) of the **5^{2+} @ PrS[6]*^{nPr}*** complex.

C	6.69271700	0.87117800	-0.79327300
C	6.10713600	-1.91643300	-0.67972600
C	7.28874600	-1.39020900	-0.12323800
C	7.58658000	-0.00615200	-0.21117300
H	6.95705000	1.92664200	-0.82780300
H	8.52923100	0.37840600	0.18529800
C	5.45593800	0.42849800	-1.33599000
C	5.19551300	-1.00843300	-1.32347900
C	4.03873000	-1.45080400	-2.02840300
C	3.15775500	-0.56773900	-2.62229200
C	3.35779300	0.83494900	-2.52825700
C	4.50795500	1.35280900	-1.91449800
H	3.83804100	-2.51295100	-2.12776000
H	2.30740800	-0.97718600	-3.17235800
H	5.82421300	3.05566600	-1.76713200
H	4.57047600	3.19873700	-2.98365000
C	5.99466800	-3.44108600	-0.58336700
H	6.22705900	-3.71316200	0.46177200
H	6.83801400	-3.85624000	-1.16007400
O	2.43265800	1.72732500	-3.02565900
O	8.11773500	-2.27937000	0.48003900
C	1.36638800	1.23422800	-3.83436300
H	1.77310200	0.69778100	-4.71527600
H	0.76585500	0.48990300	-3.26348000
C	9.46331900	-1.91305800	0.82384800

H	9.94663600	-1.44031400	-0.05470100
H	9.44811200	-1.17567900	1.65033700
C	2.34311900	-5.33710400	-1.95205400
C	4.73562800	-4.14709300	-1.01412000
C	4.70290700	-4.79495500	-2.25750100
C	3.49491500	-5.39424900	-2.71549600
H	1.43500900	-5.77935600	-2.36229300
H	3.46008900	-5.88842600	-3.68976300
C	3.55866200	-4.13281900	-0.19898200
C	2.31857700	-4.71506900	-0.66930000
C	1.13527900	-4.65632500	0.14131600
C	1.19458500	-3.99753000	1.37826300
C	2.41763900	-3.46226400	1.86081400
C	3.56527300	-3.53071000	1.08679300
H	2.47517000	-3.01772700	2.85719700
O	5.83760200	-4.75708300	-2.99851300
O	0.02544900	-3.91496800	2.09256500
C	-0.13552200	-5.39234200	-0.24404000
H	-0.28952700	-6.18285300	0.50518400
H	0.02806300	-5.92267900	-1.19235700
C	-3.83818100	-3.21030400	-0.92664000
C	-2.72648200	-2.99989500	-1.72377300
C	-1.50453700	-3.66628500	-1.44635600
C	-1.41071200	-4.58457200	-0.39616700
H	-4.77729200	-2.71094700	-1.17380100
H	-2.80642200	-2.34147500	-2.58899700
C	-3.78895300	-4.07883700	0.20100900
C	-2.55565100	-4.79949000	0.44528300
C	-2.54567900	-5.72378900	1.52717000
C	-3.64989400	-5.90603400	2.33804600
C	-4.83926400	-5.15610500	2.12548200
C	-4.92234000	-4.24650900	1.05789000
H	-1.64483000	-6.29864500	1.74943200
H	-3.59352000	-6.62721100	3.15698700
O	-0.35413000	-3.42348700	-2.16599700
O	-5.92424400	-5.25594400	2.93712700
H	-6.91851400	-3.78961000	1.63503200
H	-6.69033000	-3.94389300	-0.09314300
C	-6.23052300	-3.52136500	0.81582100
C	-6.66500700	0.80233900	0.47141500
C	-6.26485100	-2.00637100	0.67098300
C	-7.17443100	-1.46819400	-0.25583600
C	-7.40690800	-0.06854900	-0.30276600
H	-6.87077800	1.86691900	0.39155500
H	-8.19091500	0.33338000	-0.94797400
C	-5.51148900	-1.10230900	1.49039200
C	-5.64505000	0.33996300	1.34648800
C	-4.80883200	1.24656600	2.09969800
C	-3.94517800	0.69393200	3.05871000
C	-3.88421700	-0.71028700	3.27247100
C	-4.63174200	-1.57931100	2.50104400
H	-3.26170800	-1.12109100	4.06820700
H	-4.55978600	-2.65017600	2.69085100
O	-7.82675300	-2.34303700	-1.06646200
O	-3.14016400	1.54812200	3.77708800
C	-4.90218700	2.76957300	1.92967100
H	-4.82404000	3.21861900	2.92982400
H	-5.91036000	3.01811000	1.56841200
C	-2.17188600	4.90412700	-0.77951500
C	-3.29020300	4.25158000	-1.25837800

C	-4.19105000	3.59029600	-0.37812200
C	-3.90770300	3.47571000	0.99622800
H	-1.55267700	5.45655900	-1.48306000
H	-3.50388000	4.29286000	-2.32885400
C	-2.75691700	4.19178100	1.50157900
C	-1.88234100	4.93998300	0.61340100
C	-0.81798800	5.76286400	1.11915000
C	-0.62682000	5.83270200	2.50785800
C	-1.47380100	5.09999300	3.38261000
C	-2.49921100	4.31284000	2.89652400
H	-1.34540000	5.17876000	4.46425700
H	-3.13219500	3.79025800	3.60947100
O	-5.36712100	3.08311800	-0.84456300
O	0.36520800	6.65157900	2.96569700
C	-0.42805600	-2.95993700	-3.51862500
H	0.63129000	-2.85266000	-3.81277400
H	-0.88688800	-1.94747500	-3.55675300
C	0.04102700	-3.41954200	3.42423900
H	0.39136700	-2.35991100	3.43563300
H	0.75725300	-3.99652700	4.04447100
C	5.91671200	-5.44628500	-4.25108700
H	5.67227200	-6.51833100	-4.10568100
H	5.17441900	-5.02286300	-4.95951800
C	-5.92571300	-6.15719600	4.04881800
H	-5.75994800	-7.19410800	3.69176900
H	-5.09505900	-5.90180300	4.74013100
C	-8.67753200	-1.86490000	-2.11225000
H	-9.54915900	-1.33454500	-1.67719900
H	-8.12203000	-1.13778300	-2.74250800
C	0.71750600	6.66576400	4.35422400
H	1.32145200	7.58013600	4.47841000
H	-0.18820100	6.78961400	4.97869500
C	-5.85992900	3.52298100	-2.12044500
H	-5.86465400	4.63100100	-2.14515000
H	-5.18783500	3.17040400	-2.92731600
C	-2.32177200	1.02840300	4.82320700
H	-2.95219000	0.55996000	5.60556900
H	-1.66012300	0.22730800	4.42793500
H	4.49734300	-3.12774900	1.48639200
C	4.76014500	2.86075800	-1.95541400
C	2.37639800	5.12009500	0.94609500
C	3.49748500	4.41321700	1.32843500
C	4.27905600	3.70975900	0.37307300
C	3.93873800	3.69900700	-0.98118700
H	1.80379800	5.66079100	1.69951800
H	3.80889900	4.38284500	2.37925000
C	2.82920200	4.51863800	-1.40791900
C	2.00764500	5.20637800	-0.43065800
C	0.88245400	5.99353400	-0.83521100
C	0.65682100	6.18873500	-2.20773600
C	1.50289400	5.57836900	-3.17174600
C	2.53615500	4.74834800	-2.77977300
H	1.33857400	5.75500400	-4.23724300
H	3.14347700	4.27725800	-3.55359300
C	-0.01320300	6.66924700	0.19250400
H	-0.71802100	7.32137800	-0.34613100
H	0.60106700	7.33405400	0.81894900
O	5.36056300	2.99631200	0.82916300
O	-0.40606200	6.96488000	-2.55612000
C	6.49245900	3.78386900	1.25757400

H	6.13811300	4.73467300	1.70161500
H	7.09773000	4.05086900	0.36559500
C	-0.78468300	7.11631700	-3.92834000
H	0.01989300	7.63868900	-4.48406000
H	-0.91941700	6.11555900	-4.39135000
N	1.35226100	0.76620800	0.92870100
C	0.40360300	0.68941000	2.09780300
C	-1.04919700	0.75923200	1.59053300
N	-1.08689400	0.51831200	0.10119700
C	-0.29572200	-0.74022000	-0.18214400
C	1.19102500	-0.49529800	0.10993700
H	0.62567500	1.53031100	2.77091000
H	0.61013800	-0.25307500	2.62369600
H	-1.51504600	1.73790900	1.77733000
H	-1.67906900	-0.01189400	2.05536300
H	-0.72072500	-1.54120800	0.44132500
H	-0.44748100	-1.02545800	-1.22979600
H	1.79383000	-0.36314300	-0.79989800
H	1.61661800	-1.33877200	0.67201400
C	-2.54794400	0.36791600	-0.34516200
H	-2.87060500	-0.61941400	0.02260000
H	-3.10257200	1.14093400	0.20606900
C	0.94401500	1.95770000	0.08401400
C	2.80902400	0.97475200	1.36166500
H	3.36774900	1.09462800	0.42094600
H	2.81160000	1.94710800	1.87610400
C	-0.41375800	1.69015700	-0.57301300
H	-1.07645100	2.56478300	-0.48677900
H	-0.30295900	1.43079700	-1.63122700
H	1.72036000	2.12250700	-0.67530800
H	0.92502700	2.83606300	0.74592700
C	-2.76691500	0.50711600	-1.84176300
H	-2.51132700	1.52972200	-2.16995700
H	-2.11309600	-0.19100000	-2.39314300
C	3.41520100	-0.13192800	2.20071700
H	2.82211500	-0.32200700	3.11568300
H	3.43019000	-1.06628800	1.62088700
C	-4.23982300	0.24400700	-2.19356000
H	-4.53416400	-0.75615200	-1.83626400
H	-4.86040000	0.95160900	-1.62349100
C	4.86000200	0.22219400	2.58806800
H	4.85565100	1.08985200	3.27475000
H	5.40120000	0.55454800	1.68709900
C	-4.53999300	0.37299600	-3.69277100
H	-5.63817800	0.39736900	-3.82832600
H	-4.17121400	1.35254800	-4.05987600
C	5.60564700	-0.95320400	3.22561300
H	5.03484300	-1.34301600	4.09326100
H	5.65778800	-1.78060300	2.49281600
C	-3.96246800	-0.74348100	-4.57351700
H	-2.86665000	-0.81313300	-4.42327200
H	-4.37643700	-1.71460100	-4.23857600
C	7.03285300	-0.60700000	3.66263000
H	7.00283400	0.20962100	4.41073700
H	7.57617400	-0.19638800	2.79070300
C	-4.24646800	-0.54578000	-6.06460800
H	-3.83480100	-1.37282800	-6.66921300
H	-5.33312700	-0.49684100	-6.26058900
H	-3.80050500	0.39634500	-6.43416800
C	7.79234900	-1.80972500	4.22654300

H	8.82619900	-1.54247300	4.50733700
H	7.29532000	-2.21243000	5.12803800
H	7.84409500	-2.62449100	3.48218500
C	-1.36737400	-3.50708100	3.99458000
H	-2.05798800	-2.99399500	3.30104600
H	-1.68221100	-4.56413400	3.99811600
C	7.32972800	-5.28283600	-4.79377200
H	7.54832500	-4.20172900	-4.88009000
H	8.03968900	-5.68933800	-4.04930600
C	-1.46443200	-2.91697500	5.40291800
H	-2.49841100	-2.97352000	5.78419400
H	-0.81835800	-3.45874600	6.11701200
H	-1.15789800	-1.85384800	5.42625300
C	7.51363300	-5.97853500	-6.14527300
H	8.54372000	-5.84792500	-6.51770300
H	7.32166300	-7.06468900	-6.07124200
H	6.82814100	-5.56801900	-6.90966500
C	0.47309800	2.38791500	-4.26912900
H	0.15816000	2.96087900	-3.37678000
H	1.06818600	3.09076100	-4.87709600
C	10.21158200	-3.17393800	1.23458800
H	11.21598500	-2.85734100	1.57459000
H	9.71066400	-3.61172900	2.11849400
C	-0.74634400	1.89996000	-5.05686300
H	-1.38396200	2.74746100	-5.36162900
H	-0.44957100	1.36175200	-5.97496500
H	-1.37167600	1.21325000	-4.45822200
C	10.33072800	-4.21404700	0.11731000
H	10.89744700	-5.09718600	0.45878900
H	9.33511200	-4.55576700	-0.21433100
H	10.85540300	-3.79671700	-0.76205500
C	7.32060400	2.99722900	2.26117900
H	6.69895900	2.79394100	3.15286100
H	7.56339300	2.01297000	1.82498300
C	-2.08225700	7.91120400	-3.97800900
H	-2.84627900	7.37567900	-3.38212800
H	-1.91797000	8.88216300	-3.47536200
C	8.60195000	3.73421800	2.65989800
H	9.17956300	3.14947900	3.39609300
H	9.25500800	3.91071300	1.78566200
H	8.38157800	4.71752900	3.11412100
C	-2.57226100	8.12633700	-5.41259600
H	-3.51137800	8.70479600	-5.42489100
H	-1.83109300	8.68370200	-6.01377200
H	-2.76437500	7.16535600	-5.92565000
C	1.52060400	5.43237400	4.77666800
H	0.97719600	4.51981900	4.46411200
H	2.47866400	5.43027200	4.22388500
C	-7.25805400	2.96781000	-2.34366200
H	-7.21124700	1.86625800	-2.29522900
H	-7.91371100	3.29668200	-1.51613000
C	1.77535600	5.39661700	6.28626000
H	2.38299800	4.51942700	6.56876400
H	2.31524200	6.29854500	6.62657900
H	0.82712500	5.34517800	6.85256700
C	-7.83233400	3.41185200	-3.69223200
H	-8.84588300	3.00304600	-3.84198800
H	-7.90440700	4.51239200	-3.76126300
H	-7.20382800	3.06272600	-4.53254200
C	-9.13441800	-3.05691300	-2.94162700

H	-8.23912200	-3.57941400	-3.32877300
H	-9.65075200	-3.77118300	-2.27381600
C	-1.46723100	2.14432000	5.40853500
H	-0.90506600	2.62816200	4.58868800
H	-2.12842300	2.92502100	5.82710500
C	-10.05327200	-2.63926700	-4.09296000
H	-10.37242300	-3.51691900	-4.67994100
H	-10.96577700	-2.13802900	-3.72144100
H	-9.54570500	-1.94163500	-4.78519300
C	-0.50571300	1.62823800	6.48252600
H	0.10572900	2.45027000	6.89216800
H	-1.04814500	1.16410200	7.32555100
H	0.18701000	0.86667800	6.07552600
C	-1.14851000	-3.90346000	-4.48256000
H	-1.06961300	-3.44771100	-5.48895900
H	-2.22751300	-3.93006900	-4.24560600
C	-7.26860800	-6.03951500	4.75633300
H	-7.41373500	-4.98585200	5.06118200
H	-8.06890500	-6.26961000	4.02853700
C	-0.58429900	-5.32563100	-4.49593100
H	-1.09062100	-5.94156700	-5.25827000
H	0.49776200	-5.32786400	-4.72036200
H	-0.73087400	-5.81928200	-3.51997600
C	-7.36480700	-6.96823700	5.96990700
H	-8.34494900	-6.86671600	6.46568800
H	-7.24702700	-8.02821800	5.67909300
H	-6.58596400	-6.73792200	6.72040100

1 4 2.0 5 1.0 7 1.5
2 3 1.5 8 1.5 17 1.0
3 4 1.5 21 1.0
4 6 1.0
5
6
7 8 1.0 12 1.5
8 9 1.5
9 10 2.0 13 1.0
10 11 1.5 14 1.0
11 12 1.5 20 1.0
12 125 1.0
13
14
15 125 1.0
16 125 1.0
17 18 1.0 19 1.0 29 1.0
18
19
20 22 1.0
21 25 1.0
22 23 1.0 24 1.0 223 1.0
23
24
25 26 1.0 27 1.0 226 1.0
26
27
28 31 2.0 32 1.0 35 1.5
29 30 1.5 34 1.5
30 31 1.5 41 1.0
31 33 1.0
32

33
34 35 1.0 39 1.5
35 36 1.5
36 37 1.5 43 1.0
37 38 1.5 42 1.0
38 39 2.0 40 1.0
39 124 1.0
40
41 106 1.0
42 103 1.0
43 44 1.0 45 1.0 49 1.0
44
45
46 47 2.0 50 1.0 52 1.5
47 48 1.5 51 1.0
48 49 1.5 60 1.0
49 53 1.5
50
51
52 53 1.0 57 1.5
53 54 1.5
54 55 2.0 58 1.0
55 56 1.5 59 1.0
56 57 1.5 61 1.0
57 64 1.0
58
59
60 100 1.0
61 109 1.0
62 64 1.0
63 64 1.0
64 66 1.0
65 68 2.0 69 1.0 72 1.5
66 67 1.5 71 1.5
67 68 1.5 79 1.0
68 70 1.0
69
70
71 72 1.0 76 1.5
72 73 1.5
73 74 1.5 81 1.0
74 75 1.5 80 1.0
75 76 2.0 77 1.0
76 78 1.0
77
78
79 112 1.0
80 121 1.0
81 82 1.0 83 1.0 87 1.0
82
83
84 85 2.0 88 1.0 91 1.5
85 86 1.5 89 1.0
86 87 1.5 98 1.0
87 90 1.5
88
89
90 91 1.0 95 1.5
91 92 1.5
92 93 1.5 140 1.0

93 94 1.5 99 1.0
94 95 2.0 96 1.0
95 97 1.0
96
97
98 118 1.0
99 115 1.0
100 101 1.0 102 1.0 279 1.0
101
102
103 104 1.0 105 1.0 209 1.0
104
105
106 107 1.0 108 1.0 212 1.0
107
108
109 110 1.0 111 1.0 282 1.0
110
111
112 113 1.0 114 1.0 265 1.0
113
114
115 116 1.0 117 1.0 251 1.0
116
117
118 119 1.0 120 1.0 254 1.0
119
120
121 122 1.0 123 1.0 268 1.0
122
123
124
125 129 1.0
126 127 2.0 130 1.0 133 1.5
127 128 1.5 131 1.0
128 129 1.5 143 1.0
129 132 1.5
130
131
132 133 1.0 137 1.5
133 134 1.5
134 135 1.5 140 1.0
135 136 1.5 144 1.0
136 137 2.0 138 1.0
137 139 1.0
138
139
140 141 1.0 142 1.0
141
142
143 145 1.0
144 148 1.0
145 146 1.0 147 1.0 237 1.0
146
147
148 149 1.0 150 1.0 240 1.0
149
150
151 152 1.0 156 1.0 168 1.0 169 1.0
152 153 1.0 157 1.0 158 1.0

153 154 1.0 159 1.0 160 1.0
154 155 1.0 165 1.0 172 1.0
155 156 1.0 161 1.0 162 1.0
156 163 1.0 164 1.0
157
158
159
160
161
162
163
164
165 166 1.0 167 1.0 177 1.0
166
167
168 172 1.0 175 1.0 176 1.0
169 170 1.0 171 1.0 180 1.0
170
171
172 173 1.0 174 1.0
173
174
175
176
177 178 1.0 179 1.0 183 1.0
178
179
180 181 1.0 182 1.0 186 1.0
181
182
183 184 1.0 185 1.0 189 1.0
184
185
186 187 1.0 188 1.0 192 1.0
187
188
189 190 1.0 191 1.0 195 1.0
190
191
192 193 1.0 194 1.0 198 1.0
193
194
195 196 1.0 197 1.0 201 1.0
196
197
198 199 1.0 200 1.0 205 1.0
199
200
201 202 1.0 203 1.0 204 1.0
202
203
204
205 206 1.0 207 1.0 208 1.0
206
207
208
209 210 1.0 211 1.0 215 1.0
210
211
212 213 1.0 214 1.0 219 1.0

213
214
215 216 1.0 217 1.0 218 1.0
216
217
218
219 220 1.0 221 1.0 222 1.0
220
221
222
223 224 1.0 225 1.0 229 1.0
224
225
226 227 1.0 228 1.0 233 1.0
227
228
229 230 1.0 231 1.0 232 1.0
230
231
232
233 234 1.0 235 1.0 236 1.0
234
235
236
237 238 1.0 239 1.0 243 1.0
238
239
240 241 1.0 242 1.0 247 1.0
241
242
243 244 1.0 245 1.0 246 1.0
244
245
246
247 248 1.0 249 1.0 250 1.0
248
249
250
251 252 1.0 253 1.0 257 1.0
252
253
254 255 1.0 256 1.0 261 1.0
255
256
257 258 1.0 259 1.0 260 1.0
258
259
260
261 262 1.0 263 1.0 264 1.0
262
263
264
265 266 1.0 267 1.0 271 1.0
266
267
268 269 1.0 270 1.0 275 1.0
269
270
271 272 1.0 273 1.0 274 1.0
272

273
274
275 276 1.0 277 1.0 278 1.0
276
277
278
279 280 1.0 281 1.0 285 1.0
280
281
282 283 1.0 284 1.0 289 1.0
283
284
285 286 1.0 287 1.0 288 1.0
286
287
288
289 290 1.0 291 1.0 292 1.0
290
291
292

0 imaginary frequency

Atomic coordinates of **5^{2+}@ PrS[5]^{nPr}** complex.

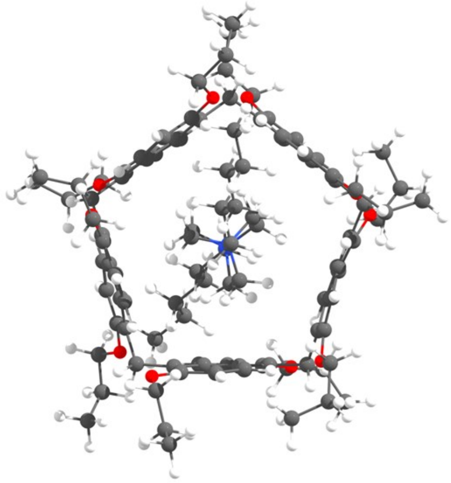

Figure S83. DFT-optimized structure (B97D3/SVP/SVPFIT) of the **5^{2+} @ PrS[5]^{nPr}** complex.

C	2.26720000	-2.56540000	3.13550000
C	0.53790000	-4.32650000	1.70660000
C	0.26470000	-3.96350000	3.03830000
C	1.11470000	-3.05480000	3.72690000
H	2.90720000	-1.90720000	3.72290000
H	0.88550000	-2.75940000	4.75390000
C	2.66740000	-2.98660000	1.83370000
C	1.77820000	-3.88590000	1.12340000
C	2.22160000	-4.38070000	-0.13800000
C	3.47040000	-4.06460000	-0.63760000
C	4.34660000	-3.19840000	0.07070000
C	3.94740000	-2.61870000	1.28480000
H	1.58590000	-5.05540000	-0.70830000
H	3.79090000	-4.49950000	-1.58830000
C	4.90900000	-1.71130000	2.04470000

H	5.04920000	-2.10800000	3.06140000
H	5.88930000	-1.76520000	1.54880000
C	-0.42900000	-5.22950000	0.93780000
H	-1.09690000	-5.71680000	1.66250000
H	0.13500000	-6.03650000	0.44850000
O	5.58690000	-2.89350000	-0.40970000
O	-0.81840000	-4.53010000	3.63810000
C	6.28370000	-3.82390000	-1.25940000
H	6.22870000	-4.83240000	-0.80360000
H	5.79760000	-3.86500000	-2.25220000
C	-1.17770000	-4.19530000	4.98390000
H	-0.33660000	-4.44290000	5.66210000
H	-1.36760000	-3.10350000	5.05590000
C	-2.73200000	-3.10680000	-2.12420000
C	-1.26550000	-4.51410000	-0.12460000
C	-0.97760000	-4.68610000	-1.49140000
C	-1.69140000	-3.94980000	-2.47690000
H	-3.27820000	-2.59940000	-2.91860000
H	-1.44940000	-4.07080000	-3.53600000
C	-2.40350000	-3.70810000	0.23350000
C	-3.15650000	-2.98030000	-0.76970000
C	-4.34640000	-2.24660000	-0.42130000
C	-4.80040000	-2.29960000	0.90540000
C	-4.05640000	-2.99810000	1.89460000
C	-2.88780000	-3.66120000	1.57380000
H	-4.41820000	-3.02260000	2.92620000
O	-0.01730000	-5.59110000	-1.82560000
O	-5.96210000	-1.65390000	1.21480000
C	-5.17500000	-1.52660000	-1.48010000
H	-6.13980000	-1.25410000	-1.02770000
H	-5.40040000	-2.23120000	-2.29470000
C	-3.26430000	1.98140000	-3.23870000
C	-3.49070000	0.84750000	-3.99710000
C	-4.13860000	-0.28610000	-3.43490000
C	-4.53410000	-0.28570000	-2.08630000
H	-2.77920000	2.83090000	-3.71540000
H	-3.18790000	0.84680000	-5.04680000
C	-3.70170000	2.07360000	-1.88760000
C	-4.37380000	0.92070000	-1.32290000
C	-4.90570000	1.04960000	-0.00670000
C	-4.79650000	2.23450000	0.69910000
C	-4.11930000	3.35820000	0.14720000
C	-3.52790000	3.28030000	-1.12540000
H	-5.44660000	0.22160000	0.45320000
H	-5.25810000	2.30230000	1.68730000
O	-4.40090000	-1.40730000	-4.16160000
O	-4.01450000	4.54140000	0.81570000
H	-2.95300000	5.34470000	-1.05720000
H	-3.17520000	4.72080000	-2.68970000
C	-2.76550000	4.47160000	-1.69930000
C	1.54750000	3.87250000	-2.15580000
C	-1.25890000	4.27020000	-1.84640000
C	-0.67910000	4.16040000	-3.12310000
C	0.71150000	3.88720000	-3.25910000
H	2.61580000	3.72440000	-2.31620000
H	1.14630000	3.73710000	-4.25090000
C	-0.38670000	4.29200000	-0.70210000
C	1.04980000	4.15130000	-0.84910000
C	1.93930000	4.38810000	0.25670000
C	1.38570000	4.81670000	1.47670000

C	-0.02600000	4.82810000	1.65930000
C	-0.88470000	4.56180000	0.60640000
H	-0.45190000	5.09950000	2.62890000
H	-1.95820000	4.64500000	0.77850000
O	-1.49030000	4.32930000	-4.20300000
O	2.24410000	5.21460000	2.45630000
C	3.45370000	4.28140000	0.09160000
H	3.92740000	4.78600000	0.94700000
H	3.76040000	4.83800000	-0.80610000
C	4.97600000	0.19190000	-0.24980000
C	5.03410000	1.06020000	-1.32620000
C	4.55870000	2.39690000	-1.21850000
C	3.99720000	2.85870000	-0.01540000
H	5.36990000	-0.81710000	-0.37800000
H	5.47330000	0.70780000	-2.26260000
C	3.99750000	1.97870000	1.12040000
C	4.46940000	0.61330000	1.01390000
C	4.47910000	-0.25570000	2.15950000
C	4.13580000	0.27830000	3.41210000
C	3.66180000	1.61450000	3.51710000
C	3.58130000	2.42970000	2.40490000
H	3.38140000	2.02440000	4.49010000
H	3.23500000	3.45210000	2.54000000
O	4.62470000	3.27650000	-2.25770000
O	4.24880000	-0.53910000	4.49950000
C	-4.05130000	-1.48720000	-5.54970000
H	-2.95620000	-1.34710000	-5.66440000
H	-4.56150000	-0.67370000	-6.10370000
C	-6.78840000	-2.12990000	2.29450000
H	-6.90700000	-3.22700000	2.19430000
H	-6.29900000	-1.92100000	3.26410000
C	0.35910000	-5.79820000	-3.19240000
H	-0.52680000	-6.12500000	-3.77310000
H	0.72070000	-4.84170000	-3.62540000
C	-4.61270000	4.71550000	2.10620000
H	-5.70540000	4.54450000	2.03070000
H	-4.19710000	3.96710000	2.81310000
C	-0.93920000	4.39760000	-5.52790000
H	-0.49820000	3.41370000	-5.79440000
H	-0.13110000	5.15420000	-5.54570000
C	4.23790000	0.00190000	5.83090000
H	4.15530000	-0.88560000	6.48110000
H	3.32390000	0.60920000	5.99160000
C	5.20810000	2.89640000	-3.51000000
H	6.26140000	2.59110000	-3.34710000
H	4.66000000	2.02560000	-3.92660000
C	1.74670000	5.78740000	3.67580000
H	1.07050000	6.62950000	3.43140000
H	1.16120000	5.02330000	4.22990000
H	-2.35350000	-4.18980000	2.36110000
C	7.72620000	-3.35760000	-1.39780000
H	7.72750000	-2.34810000	-1.85310000
H	8.21050000	-4.02640000	-2.13450000
C	-2.42320000	-4.98740000	5.36170000
H	-3.25520000	-4.68680000	4.69540000
H	-2.71230000	-4.66490000	6.38020000
C	-4.48170000	-2.85200000	-6.07160000
H	-4.12770000	-2.92440000	-7.11760000
H	-3.93990000	-3.63460000	-5.50510000
C	-8.13200000	-1.41870000	2.21800000

H	-8.70670000	-1.72130000	3.11390000
H	-7.95960000	-0.32930000	2.31790000
C	1.45090000	-6.86010000	-3.23630000
H	1.76540000	-6.95390000	-4.29330000
H	2.33510000	-6.48810000	-2.68310000
C	-4.31730000	6.13170000	2.58300000
H	-4.71960000	6.21700000	3.61050000
H	-3.21980000	6.25560000	2.66850000
C	-2.04680000	4.77070000	-6.50320000
H	-1.56290000	4.93220000	-7.48520000
H	-2.46630000	5.74830000	-6.20080000
C	5.49920000	0.79580000	6.16460000
H	6.37270000	0.13140000	6.02980000
H	5.62030000	1.62110000	5.43860000
C	5.12910000	4.08770000	-4.45590000
H	5.51260000	3.74720000	-5.43660000
H	4.06340000	4.34630000	-4.61280000
C	2.92410000	6.27020000	4.51170000
H	3.49290000	7.01210000	3.92090000
H	2.49710000	6.82120000	5.37100000
C	8.51450000	-3.35250000	-0.08580000
H	9.56000000	-3.04310000	-0.25510000
H	8.07110000	-2.65550000	0.64600000
H	8.53110000	-4.35800000	0.37280000
C	-2.22950000	-6.50550000	5.32040000
H	-3.15090000	-7.02870000	5.62770000
H	-1.96630000	-6.84690000	4.30480000
H	-1.41930000	-6.82130000	6.00260000
C	-5.99190000	-3.09380000	-6.00430000
H	-6.25070000	-4.08540000	-6.41290000
H	-6.35730000	-3.04840000	-4.96410000
H	-6.54280000	-2.33440000	-6.58890000
C	-8.93150000	-1.71820000	0.94750000
H	-9.90940000	-1.20760000	0.96740000
H	-8.39330000	-1.37990000	0.04530000
H	-9.12000000	-2.80220000	0.84180000
C	1.01420000	-8.22170000	-2.68950000
H	1.83170000	-8.95850000	-2.76840000
H	0.72130000	-8.15030000	-1.62820000
H	0.14890000	-8.61710000	-3.25220000
C	-4.90900000	7.22400000	1.68830000
H	-4.68640000	8.22680000	2.09100000
H	-4.49780000	7.16840000	0.66590000
H	-6.00760000	7.12650000	1.61580000
C	-3.16090000	3.72880000	-6.63300000
H	-3.88410000	4.02330000	-7.41250000
H	-2.75140000	2.73990000	-6.91510000
H	-3.71980000	3.60970000	-5.68950000
C	5.46040000	1.35490000	7.58990000
H	6.38920000	1.90200000	7.82360000
H	4.61660000	2.05730000	7.72560000
H	5.35040000	0.54990000	8.33930000
C	5.91020000	5.31390000	-3.97650000
H	5.83950000	6.13690000	-4.70810000
H	5.52220000	5.68190000	-3.01140000
H	6.98080000	5.07510000	-3.84080000
C	3.85370000	5.16070000	5.01180000
H	4.63370000	5.57260000	5.67470000
H	3.29370000	4.40060000	5.58990000
H	4.36260000	4.64800000	4.17840000

N	0.49070000	-0.14350000	-1.19010000
C	1.49800000	0.54990000	-0.29910000
C	0.78880000	1.08570000	0.94390000
N	-0.52470000	0.35410000	1.12360000
C	-0.26720000	-1.12820000	0.96200000
C	0.06580000	-1.42480000	-0.50570000
H	1.96250000	1.37090000	-0.86320000
H	2.26950000	-0.18380000	-0.03740000
H	0.55660000	2.15680000	0.86450000
H	1.40600000	0.92040000	1.83790000
H	0.55780000	-1.38980000	1.64050000
H	-1.16750000	-1.68010000	1.26060000
H	-0.79850000	-1.81550000	-1.06180000
H	0.88810000	-2.14890000	-0.56380000
C	-1.09380000	0.69650000	2.49220000
H	-0.33220000	0.37010000	3.21970000
H	-1.13560000	1.79900000	2.50670000
C	-0.72520000	0.75050000	-1.31790000
C	1.06580000	-0.39460000	-2.57570000
H	0.24990000	-0.85710000	-3.15570000
H	1.25290000	0.61290000	-2.98500000
C	-1.46450000	0.78600000	0.01890000
H	-1.82240000	1.80220000	0.23620000
H	-2.31750000	0.09740000	0.02370000
H	-1.37640000	0.34350000	-2.10390000
H	-0.37220000	1.74430000	-1.62740000
C	-2.45570000	0.08560000	2.78680000
H	-3.12730000	0.20000000	1.91980000
H	-2.36260000	-0.99840000	2.97180000
C	2.32660000	-1.24660000	-2.59640000
H	3.02410000	-0.92650000	-1.80450000
H	2.08290000	-2.30360000	-2.39280000
C	-3.12350000	0.76600000	3.98930000
H	-2.47750000	0.68830000	4.88610000
H	-3.22560000	1.84910000	3.77660000
C	3.05750000	-1.12830000	-3.94030000
H	3.32250000	-0.06550000	-4.11060000
H	2.38520000	-1.41470000	-4.77320000
C	-4.50550000	0.17560000	4.28620000
H	-5.10050000	0.17840000	3.35220000
H	-4.39020000	-0.89080000	4.56540000
C	4.32970000	-1.98080000	-3.97790000
H	4.93900000	-1.75050000	-3.08240000
H	4.05120000	-3.04950000	-3.88400000
C	-5.27590000	0.91140000	5.38780000
H	-4.67500000	0.90850000	6.31850000
H	-5.38390000	1.97740000	5.10200000
C	5.17680000	-1.78210000	-5.23930000
H	5.44930000	-0.71070000	-5.32540000
H	4.56170000	-2.00890000	-6.13240000
C	-6.65660000	0.30790000	5.65900000
H	-7.18630000	0.85260000	6.45920000
H	-7.29230000	0.33970000	4.75520000
H	-6.57650000	-0.74960000	5.97260000
C	6.44540000	-2.63930000	-5.25100000
H	7.03330000	-2.47950000	-6.17110000
H	7.09790000	-2.40090000	-4.39110000
H	6.20100000	-3.71640000	-5.19470000

1 4 2.0 5 1.0 7 1.5

2 3 1.5 8 1.5 18 1.0
3 4 1.5 22 1.0
4 6 1.0
5
6
7 8 1.0 12 1.5
8 9 1.5
9 10 2.0 13 1.0
10 11 1.5 14 1.0
11 12 1.5 21 1.0
12 15 1.0
13
14
15 16 1.0 17 1.0 93 1.0
16
17
18 19 1.0 20 1.0 30 1.0
19
20
21 23 1.0
22 26 1.0
23 24 1.0 25 1.0 126 1.0
24
25
26 27 1.0 28 1.0 129 1.0
27
28
29 32 2.0 33 1.0 36 1.5
30 31 1.5 35 1.5
31 32 1.5 42 1.0
32 34 1.0
33
34
35 36 1.0 40 1.5
36 37 1.5
37 38 1.5 44 1.0
38 39 1.5 43 1.0
39 40 2.0 41 1.0
40 125 1.0
41
42 107 1.0
43 104 1.0
44 45 1.0 46 1.0 50 1.0
45
46
47 48 2.0 51 1.0 53 1.5
48 49 1.5 52 1.0
49 50 1.5 61 1.0
50 54 1.5
51
52
53 54 1.0 58 1.5
54 55 1.5
55 56 2.0 59 1.0
56 57 1.5 60 1.0
57 58 1.5 62 1.0
58 65 1.0
59
60
61 101 1.0

62 110 1.0
63 65 1.0
64 65 1.0
65 67 1.0
66 69 2.0 70 1.0 73 1.5
67 68 1.5 72 1.5
68 69 1.5 80 1.0
69 71 1.0
70
71
72 73 1.0 77 1.5
73 74 1.5
74 75 1.5 82 1.0
75 76 1.5 81 1.0
76 77 2.0 78 1.0
77 79 1.0
78
79
80 113 1.0
81 122 1.0
82 83 1.0 84 1.0 88 1.0
83
84
85 86 2.0 89 1.0 92 1.5
86 87 1.5 90 1.0
87 88 1.5 99 1.0
88 91 1.5
89
90
91 92 1.0 96 1.5
92 93 1.5
93 94 1.5
94 95 1.5 100 1.0
95 96 2.0 97 1.0
96 98 1.0
97
98
99 119 1.0
100 116 1.0
101 102 1.0 103 1.0 132 1.0
102
103
104 105 1.0 106 1.0 135 1.0
105
106
107 108 1.0 109 1.0 138 1.0
108
109
110 111 1.0 112 1.0 141 1.0
111
112
113 114 1.0 115 1.0 144 1.0
114
115
116 117 1.0 118 1.0 147 1.0
117
118
119 120 1.0 121 1.0 150 1.0
120
121

122 123 1.0 124 1.0 153 1.0
123
124
125
126 127 1.0 128 1.0 156 1.0
127
128
129 130 1.0 131 1.0 160 1.0
130
131
132 133 1.0 134 1.0 164 1.0
133
134
135 136 1.0 137 1.0 168 1.0
136
137
138 139 1.0 140 1.0 172 1.0
139
140
141 142 1.0 143 1.0 176 1.0
142
143
144 145 1.0 146 1.0 180 1.0
145
146
147 148 1.0 149 1.0 184 1.0
148
149
150 151 1.0 152 1.0 188 1.0
151
152
153 154 1.0 155 1.0 192 1.0
154
155
156 157 1.0 158 1.0 159 1.0
157
158
159
160 161 1.0 162 1.0 163 1.0
161
162
163
164 165 1.0 166 1.0 167 1.0
165
166
167
168 169 1.0 170 1.0 171 1.0
169
170
171
172 173 1.0 174 1.0 175 1.0
173
174
175
176 177 1.0 178 1.0 179 1.0
177
178
179
180 181 1.0 182 1.0 183 1.0
181

182
183
184 185 1.0 186 1.0 187 1.0
185
186
187
188 189 1.0 190 1.0 191 1.0
189
190
191
192 193 1.0 194 1.0 195 1.0
193
194
195
196 197 1.0 201 1.0 213 1.0 214 1.0
197 198 1.0 202 1.0 203 1.0
198 199 1.0 204 1.0 205 1.0
199 200 1.0 210 1.0 217 1.0
200 201 1.0 206 1.0 207 1.0
201 208 1.0 209 1.0
202
203
204
205
206
207
208
209
210 211 1.0 212 1.0 222 1.0
211
212
213 217 1.0 220 1.0 221 1.0
214 215 1.0 216 1.0 225 1.0
215
216
217 218 1.0 219 1.0
218
219
220
221
222 223 1.0 224 1.0 228 1.0
223
224
225 226 1.0 227 1.0 231 1.0
226
227
228 229 1.0 230 1.0 234 1.0
229
230
231 232 1.0 233 1.0 237 1.0
232
233
234 235 1.0 236 1.0 240 1.0
235
236
237 238 1.0 239 1.0 243 1.0
238
239
240 241 1.0 242 1.0 246 1.0
241

242
243 244 1.0 245 1.0 250 1.0
244
245
246 247 1.0 248 1.0 249 1.0
247
248
249
250 251 1.0 252 1.0 253 1.0
251
252
253

---

0 imaginary frequency

Atomic coordinates of **7^{2+}@ PrS[5]**^{nPr} complex

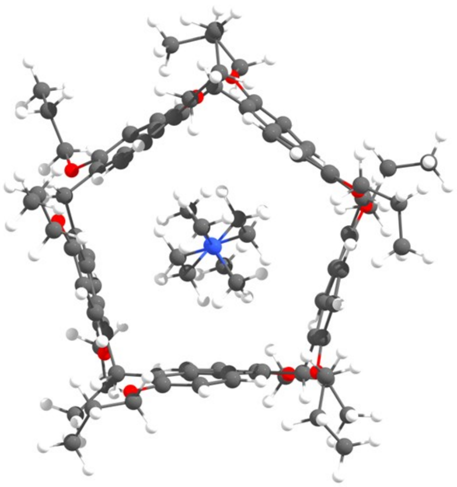

Figure S84. DFT-optimized structures (B97D3/SVP/SVPFIT) of the **7^{2+} @ PrS[5]**^{nPr} complex.

C	-0.68850000	-4.34010000	-1.80930000
C	1.96570000	-4.45880000	-0.78060000
C	1.72410000	-4.51760000	-2.16420000
C	0.39710000	-4.40990000	-2.66740000
H	-1.68780000	-4.30470000	-2.24420000
H	0.21590000	-4.42740000	-3.74540000
C	-0.51960000	-4.40280000	-0.39550000
C	0.83820000	-4.45080000	0.11110000
C	1.00910000	-4.55400000	1.52210000
C	-0.07600000	-4.67530000	2.37200000
C	-1.40500000	-4.68450000	1.86450000
C	-1.64670000	-4.49080000	0.49370000
H	2.01020000	-4.59810000	1.94980000
H	0.10520000	-4.81270000	3.44130000
C	-3.07670000	-4.47560000	-0.03560000
H	-3.16460000	-5.20280000	-0.85640000
H	-3.74230000	-4.82860000	0.76620000
C	3.39500000	-4.45490000	-0.24750000
H	4.07410000	-4.72890000	-1.06800000
H	3.50460000	-5.23590000	0.51890000
O	-2.48530000	-4.89450000	2.66740000
O	2.79960000	-4.68190000	-2.98130000
C	-2.32970000	-5.10860000	4.07450000
H	-1.69490000	-6.00040000	4.24820000
H	-1.81780000	-4.23530000	4.53250000
C	2.64490000	-4.76260000	-4.40410000

H	1.96970000	-5.60590000	-4.65270000
H	2.17970000	-3.82690000	-4.77970000
C	4.58240000	-0.62990000	1.51190000
C	3.83200000	-3.12140000	0.35290000
C	4.05280000	-3.00440000	1.73570000
C	4.40160000	-1.74790000	2.30640000
H	4.87600000	0.30470000	1.99030000
H	4.56580000	-1.66100000	3.38390000
C	4.09350000	-1.97970000	-0.48100000
C	4.46330000	-0.70120000	0.09380000
C	4.79070000	0.42090000	-0.74540000
C	4.84240000	0.22570000	-2.13640000
C	4.46180000	-1.02140000	-2.70480000
C	4.08200000	-2.08120000	-1.90190000
H	4.49780000	-1.16610000	-3.78790000
O	3.93160000	-4.13350000	2.48630000
O	5.27150000	1.26980000	-2.89970000
C	5.17770000	1.76910000	-0.14780000
H	5.63420000	2.37970000	-0.94110000
H	5.95760000	1.61620000	0.61260000
C	1.93940000	4.04250000	1.71400000
C	2.95400000	3.49750000	2.48080000
C	4.02110000	2.77520000	1.87780000
C	4.03750000	2.55590000	0.48990000
H	1.15990000	4.61270000	2.21810000
H	2.94010000	3.65890000	3.56130000
C	1.94720000	3.94240000	0.29260000
C	3.01760000	3.17440000	-0.31430000
C	3.07220000	3.13870000	-1.73830000
C	2.18790000	3.86970000	-2.51190000
C	1.17320000	4.66390000	-1.91040000
C	0.99660000	4.65770000	-0.51570000
H	3.86600000	2.58790000	-2.24210000
H	2.30930000	3.86400000	-3.59850000
O	5.05920000	2.27590000	2.60240000
O	0.34770000	5.46060000	-2.64280000
H	-0.54780000	6.13830000	-0.65070000
H	0.28460000	6.11910000	0.90180000
C	-0.12980000	5.47010000	0.11630000
C	-3.28460000	3.02780000	1.90310000
C	-1.25030000	4.63490000	0.72610000
C	-1.42370000	4.59660000	2.12060000
C	-2.41500000	3.75290000	2.69820000
H	-4.05230000	2.42750000	2.39050000
H	-2.52680000	3.70120000	3.78460000
C	-2.19210000	3.92960000	-0.10210000
C	-3.23900000	3.11620000	0.48190000
C	-4.23130000	2.48010000	-0.34030000
C	-4.22640000	2.73830000	-1.72150000
C	-3.17100000	3.49330000	-2.30520000
C	-2.17960000	4.05820000	-1.52100000
H	-3.15430000	3.67220000	-3.38370000
H	-1.41710000	4.66500000	-2.00830000
O	-0.62220000	5.39610000	2.87920000
O	-5.26220000	2.24600000	-2.45930000
C	-5.30840000	1.59430000	0.27450000
H	-6.09140000	1.42420000	-0.47950000
H	-5.79060000	2.12590000	1.10790000
C	-3.83090000	-2.23690000	1.77760000
C	-4.29880000	-1.26780000	2.64910000
C	-4.80690000	-0.03010000	2.16580000
C	-4.79850000	0.25180000	0.78890000
H	-3.48030000	-3.18160000	2.19220000
H	-4.29840000	-1.48020000	3.72160000

C	-4.38420000	-0.78200000	-0.11970000
C	-3.88440000	-2.05330000	0.36570000
C	-3.58030000	-3.12890000	-0.54330000
C	-3.86270000	-2.95050000	-1.90680000
C	-4.32110000	-1.69330000	-2.38930000
C	-4.55600000	-0.64000000	-1.52760000
H	-4.52450000	-1.55620000	-3.45400000
H	-4.93510000	0.29280000	-1.94190000
O	-5.31930000	0.91950000	2.99540000
O	-3.66130000	-4.01180000	-2.74240000
C	5.16570000	2.52250000	4.01140000
H	4.28530000	2.09100000	4.53130000
H	5.16920000	3.61630000	4.19180000
C	5.42270000	1.13770000	-4.31910000
H	6.12650000	0.30930000	-4.53690000
H	4.44290000	0.88470000	-4.77570000
C	4.16850000	-4.11330000	3.90000000
H	5.20010000	-3.75690000	4.09390000
H	3.46290000	-3.40590000	4.38360000
C	0.49470000	5.57460000	-4.06430000
H	1.52180000	5.91860000	-4.30000000
H	0.34880000	4.57910000	-4.53330000
C	-0.87180000	5.55640000	4.28430000
H	-0.68290000	4.59340000	4.80500000
H	-1.93470000	5.82660000	4.43590000
C	-4.28760000	-4.05770000	-4.03650000
H	-3.82620000	-4.93200000	-4.52610000
H	-4.00600000	-3.16550000	-4.63200000
C	-5.40400000	0.70000000	4.40960000
H	-6.01280000	-0.20580000	4.60410000
H	-4.38710000	0.52560000	4.81920000
C	-5.39860000	2.59040000	-3.84560000
H	-5.38360000	3.69260000	-3.95010000
H	-4.53800000	2.17870000	-4.41530000
H	3.82730000	-3.02660000	-2.37870000
C	-3.71220000	-5.30100000	4.68290000
H	-4.20340000	-6.15000000	4.17280000
H	-4.32580000	-4.40630000	4.46230000
C	4.02120000	-4.97030000	-5.02320000
H	4.65910000	-4.10050000	-4.77140000
H	3.88760000	-4.94900000	-6.12150000
C	6.45590000	1.88550000	4.51080000
H	6.47530000	2.01480000	5.60970000
H	6.40650000	0.79410000	4.32830000
C	5.95010000	2.45720000	-4.86780000
H	5.98010000	2.35960000	-5.96970000
H	5.21240000	3.25340000	-4.64690000
C	3.97420000	-5.52550000	4.43710000
H	4.08240000	-5.47020000	5.53700000
H	2.92940000	-5.83850000	4.24400000
C	-0.54050000	6.56970000	-4.57290000
H	-0.46360000	6.57940000	-5.67680000
H	-1.55110000	6.18300000	-4.33610000
C	0.04120000	6.64690000	4.82680000
H	-0.25980000	6.81810000	5.87770000
H	-0.17480000	7.58730000	4.28620000
C	-5.80400000	-4.22020000	-3.96300000
H	-6.02690000	-5.14340000	-3.39670000
H	-6.23780000	-3.38500000	-3.38220000
C	-6.04110000	1.92840000	5.04620000
H	-6.01970000	1.77220000	6.14150000
H	-5.39640000	2.80650000	4.84550000
C	-6.70930000	2.02050000	-4.37060000
H	-7.53960000	2.44270000	-3.77450000

H	-6.83610000	2.41180000	-5.39780000
C	-3.64830000	-5.54700000	6.19290000
H	-4.65940000	-5.68450000	6.61120000
H	-3.06460000	-6.45500000	6.42990000
H	-3.17890000	-4.69830000	6.72460000
C	4.70240000	-6.27320000	-4.59610000
H	5.68330000	-6.38420000	-5.08880000
H	4.86460000	-6.29970000	-3.50500000
H	4.08790000	-7.15080000	-4.86760000
C	7.72230000	2.47480000	3.88430000
H	8.62590000	1.99550000	4.29780000
H	7.73150000	2.33040000	2.79030000
H	7.79630000	3.55980000	4.08220000
C	7.33050000	2.84520000	-4.33140000
H	7.67410000	3.79350000	-4.77850000
H	7.31190000	2.97300000	-3.23550000
H	8.08170000	2.06980000	-4.56820000
C	4.95370000	-6.54970000	3.85800000
H	4.78430000	-7.54810000	4.29600000
H	4.84190000	-6.63490000	2.76350000
H	6.00010000	-6.26270000	4.06890000
C	-0.36640000	7.98500000	-4.01590000
H	-1.12540000	8.66830000	-4.43320000
H	-0.46570000	7.99680000	-2.91700000
H	0.62930000	8.39260000	-4.26930000
C	1.53590000	6.32300000	4.75600000
H	2.13170000	7.11260000	5.24470000
H	1.76200000	5.36880000	5.26980000
H	1.88560000	6.24090000	3.71310000
C	-6.43940000	-4.27350000	-5.35560000
H	-7.53070000	-4.41680000	-5.28450000
H	-6.26170000	-3.33710000	-5.91690000
H	-6.03230000	-5.10730000	-5.95620000
C	-7.47290000	2.19870000	4.57620000
H	-7.89340000	3.08330000	5.08390000
H	-7.50850000	2.38180000	3.48870000
H	-8.13250000	1.33930000	4.79570000
C	-6.78520000	0.49100000	-4.38070000
H	-7.72190000	0.14810000	-4.85250000
H	-5.94500000	0.05320000	-4.95340000
H	-6.75610000	0.07480000	-3.35970000
N	-0.02570000	0.03040000	1.40270000
C	0.02720000	-1.27030000	0.61990000
C	0.74580000	-1.11600000	-0.70190000
N	0.11800000	-0.05460000	-1.59060000
C	0.02800000	1.24730000	-0.81190000
C	-0.66530000	1.09180000	0.52530000
H	-1.00960000	-1.62090000	0.49950000
H	0.55880000	-2.02200000	1.22400000
H	0.71270000	-2.07450000	-1.24010000
H	1.80500000	-0.84790000	-0.57290000
H	1.05340000	1.63180000	-0.70620000
H	-0.54020000	1.97600000	-1.41100000
H	-1.72810000	0.82650000	0.42270000
H	-0.61270000	2.05540000	1.05530000
C	1.01780000	0.15580000	-2.77960000
H	0.58100000	0.94260000	-3.41040000
H	2.01160000	0.46350000	-2.42430000
H	1.09240000	-0.79480000	-3.32580000
C	-0.89450000	-0.16860000	2.61620000
H	-0.98100000	0.79460000	3.13890000
H	-1.88840000	-0.51570000	2.29820000
H	-0.42100000	-0.92580000	3.25730000
C	1.35180000	0.45410000	1.84810000

H	1.25970000	1.37690000	2.43610000
H	1.79410000	-0.35890000	2.43950000
H	1.99860000	0.65440000	0.98620000
C	-1.24460000	-0.49390000	-2.06690000
H	-1.65000000	0.28690000	-2.72410000
H	-1.13590000	-1.44950000	-2.59730000
H	-1.92180000	-0.63040000	-1.21680000

1 4 2.0 5 1.0 7 1.5
2 3 1.5 8 1.5 18 1.0
3 4 1.5 22 1.0
4 6 1.0
5
6
7 8 1.0 12 1.5
8 9 1.5
9 10 2.0 13 1.0
10 11 1.5 14 1.0
11 12 1.5 21 1.0
12 15 1.0
13
14
15 16 1.0 17 1.0 93 1.0
16
17
18 19 1.0 20 1.0 30 1.0
19
20
21 23 1.0
22 26 1.0
23 24 1.0 25 1.0 126 1.0
24
25
26 27 1.0 28 1.0 129 1.0
27
28
29 32 2.0 33 1.0 36 1.5
30 31 1.5 35 1.5
31 32 1.5 42 1.0
32 34 1.0
33
34
35 36 1.0 40 1.5
36 37 1.5
37 38 1.5 44 1.0
38 39 1.5 43 1.0
39 40 2.0 41 1.0
40 125 1.0
41
42 107 1.0
43 104 1.0
44 45 1.0 46 1.0 50 1.0
45
46
47 48 2.0 51 1.0 53 1.5
48 49 1.5 52 1.0
49 50 1.5 61 1.0
50 54 1.5
51
52
53 54 1.0 58 1.5
54 55 1.5
55 56 2.0 59 1.0
56 57 1.5 60 1.0

57 58 1.5 62 1.0
58 65 1.0
59
60
61 101 1.0
62 110 1.0
63 65 1.0
64 65 1.0
65 67 1.0
66 69 2.0 70 1.0 73 1.5
67 68 1.5 72 1.5
68 69 1.5 80 1.0
69 71 1.0
70
71
72 73 1.0 77 1.5
73 74 1.5
74 75 1.5 82 1.0
75 76 1.5 81 1.0
76 77 2.0 78 1.0
77 79 1.0
78
79
80 113 1.0
81 122 1.0
82 83 1.0 84 1.0 88 1.0
83
84
85 86 2.0 89 1.0 92 1.5
86 87 1.5 90 1.0
87 88 1.5 99 1.0
88 91 1.5
89
90
91 92 1.0 96 1.5
92 93 1.5
93 94 1.5
94 95 1.5 100 1.0
95 96 2.0 97 1.0
96 98 1.0
97
98
99 119 1.0
100 116 1.0
101 102 1.0 103 1.0 132 1.0
102
103
104 105 1.0 106 1.0 135 1.0
105
106
107 108 1.0 109 1.0 138 1.0
108
109
110 111 1.0 112 1.0 141 1.0
111
112
113 114 1.0 115 1.0 144 1.0
114
115
116 117 1.0 118 1.0 147 1.0
117
118
119 120 1.0 121 1.0 150 1.0
120

121
122 123 1.0 124 1.0 153 1.0
123
124
125
126 127 1.0 128 1.0 156 1.0
127
128
129 130 1.0 131 1.0 160 1.0
130
131
132 133 1.0 134 1.0 164 1.0
133
134
135 136 1.0 137 1.0 168 1.0
136
137
138 139 1.0 140 1.0 172 1.0
139
140
141 142 1.0 143 1.0 176 1.0
142
143
144 145 1.0 146 1.0 180 1.0
145
146
147 148 1.0 149 1.0 184 1.0
148
149
150 151 1.0 152 1.0 188 1.0
151
152
153 154 1.0 155 1.0 192 1.0
154
155
156 157 1.0 158 1.0 159 1.0
157
158
159
160 161 1.0 162 1.0 163 1.0
161
162
163
164 165 1.0 166 1.0 167 1.0
165
166
167
168 169 1.0 170 1.0 171 1.0
169
170
171
172 173 1.0 174 1.0 175 1.0
173
174
175
176 177 1.0 178 1.0 179 1.0
177
178
179
180 181 1.0 182 1.0 183 1.0
181
182
183
184 185 1.0 186 1.0 187 1.0

185
186
187
188 189 1.0 190 1.0 191 1.0
189
190
191
192 193 1.0 194 1.0 195 1.0
193
194
195
196 197 1.0 201 1.0 214 1.0 218 1.0
197 198 1.0 202 1.0 203 1.0
198 199 1.0 204 1.0 205 1.0
199 200 1.0 210 1.0 222 1.0
200 201 1.0 206 1.0 207 1.0
201 208 1.0 209 1.0
202
203
204
205
206
207
208
209
210 211 1.0 212 1.0 213 1.0
211
212
213
214 215 1.0 216 1.0 217 1.0
215
216
217
218 219 1.0 220 1.0 221 1.0
219
220
221
222 223 1.0 224 1.0 225 1.0
223
224
225

---

0 imaginary frequency

NBO and NCI analysis

Natural bond orbital (NBO) studies were performed with NBO 3.1 version implemented in Gaussian 16 and second-order perturbation theory analysis was performed on optimized structures using the B97D3/SVP/SVPFIT level of theory. The non-covalent interaction (NCI) investigations were carried out with the Multiwfn program[13] and its plot was graphed with VMD program.[14]

Plots describe the RDG values versus the electron density multiplied by the sign of the second Hessian eigenvalue (s = 0.5 a.u.; left) and gradient isosurfaces (s = 0.4 a.u.; right) for the complexes. The coloring scheme was chosen to assist in distinguishing the amplitude of the electron density corresponding to different types of interactions. Marked in green color represent medium-strong (cation···π, Van der Waals and C-H···π) interactions whereas the red color represents the repulsive ones.

---

[13] T. Lu, F. W. Chen, *J. Comput. Chem.* 2012, **33**, 580-592.
[14] W. Humphrey, A. Dalke, K. Schulten *J. Mol. Graph.* 1996, **14**, 33-38.

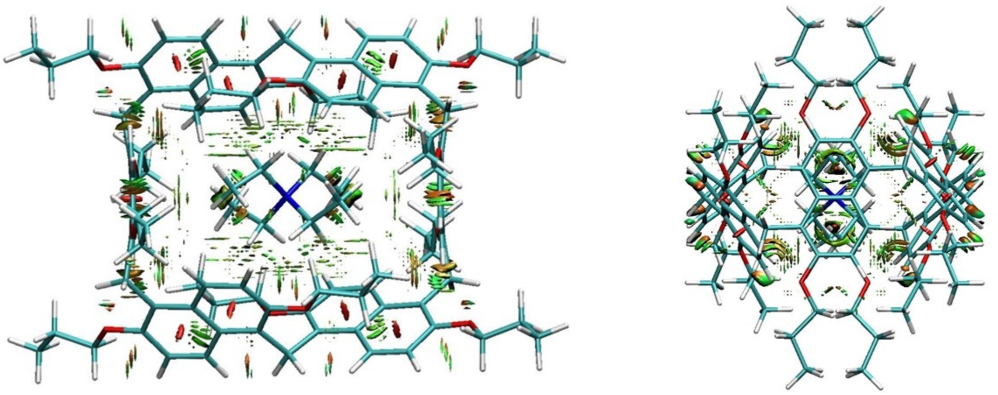

Figure S85. Gradient RDG isosurfaces (0.5) for the noncovalent interaction (NCI) regions in **6^+** @ **PrS[6]*^{nPr}*** complexes.

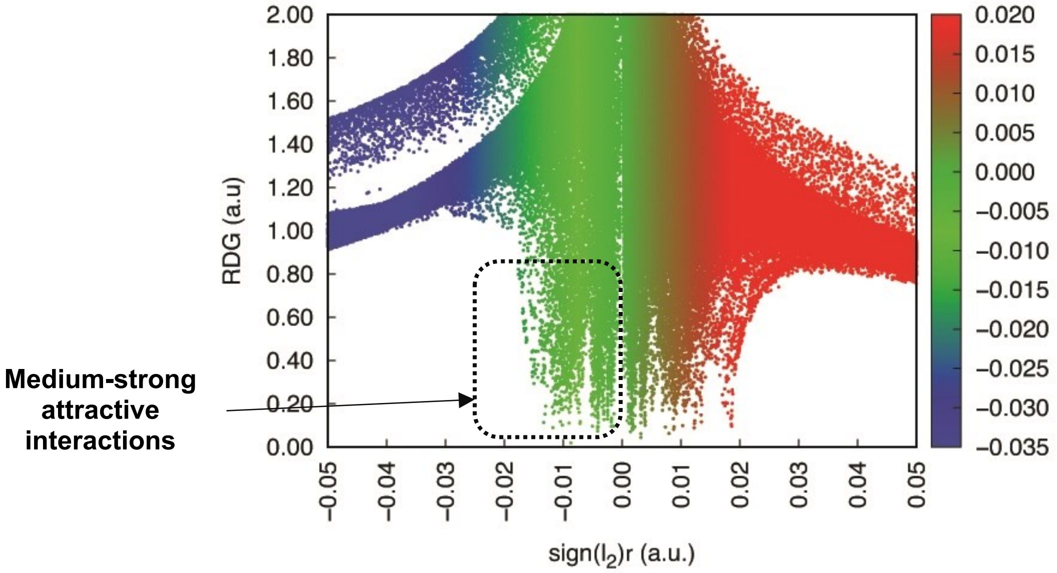

Figure S86. Plot of RDG versus sign(I_2)r for **6^+** @ **PrS[6]*^{nPr}*** complex (NCI-RDG isosurfaces with S = 0.5).

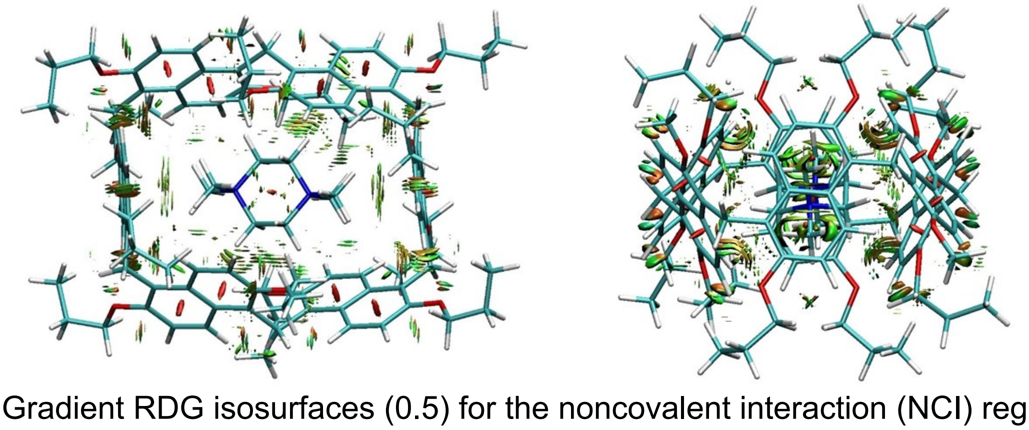

Figure S87. Gradient RDG isosurfaces (0.5) for the noncovalent interaction (NCI) regions in **7^{2+}**@ **PrS[6]*^{nPr}*** complexes.

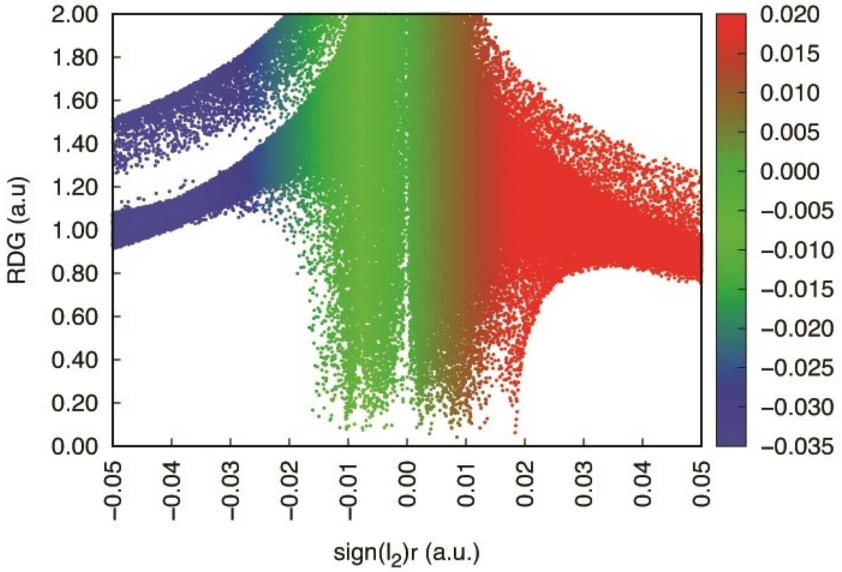

Figure S88. Plot of RDG versus sign(I_2)r for **7^{2+} @ PrS[6]^{nPr}** complex (NCI-RDG isosurfaces with S = 0.5).

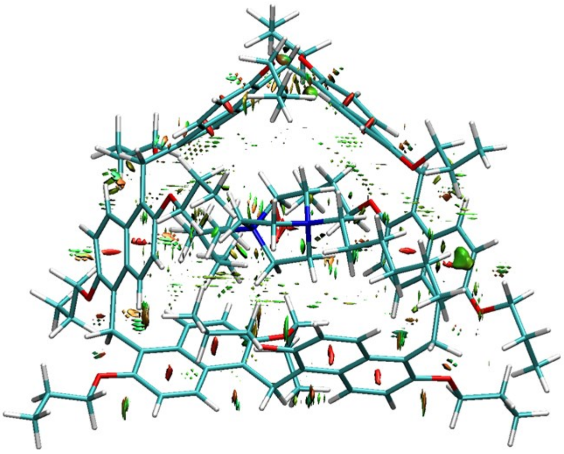

Figure S89. Gradient RDG isosurfaces (0.5) for the noncovalent interaction (NCI) regions in **5^{2+}@ PrS[6]^{nPr}** complexes.

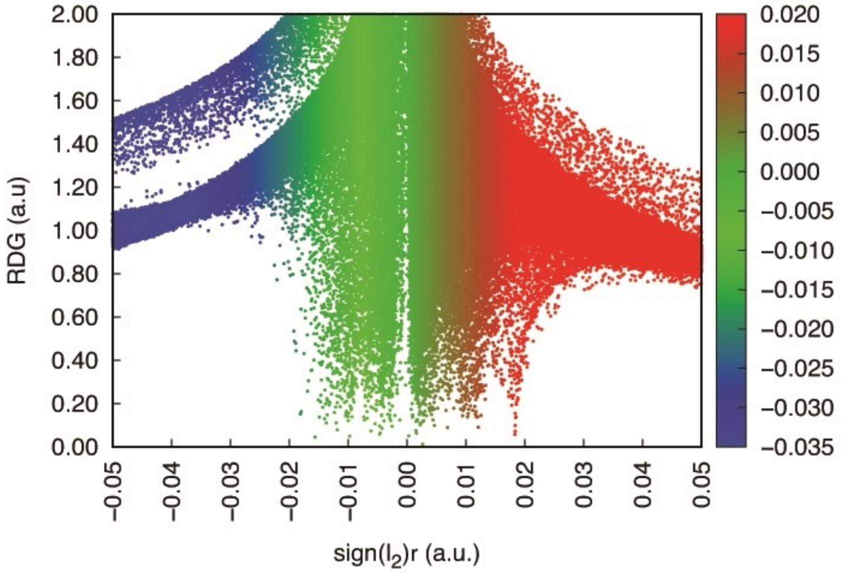

Figure S90. Plot of RDG versus sign(I_2)r for **5^{2+} @ PrS[6]^{nPr}** complex (NCI-RDG isosurfaces with S = 0.5).

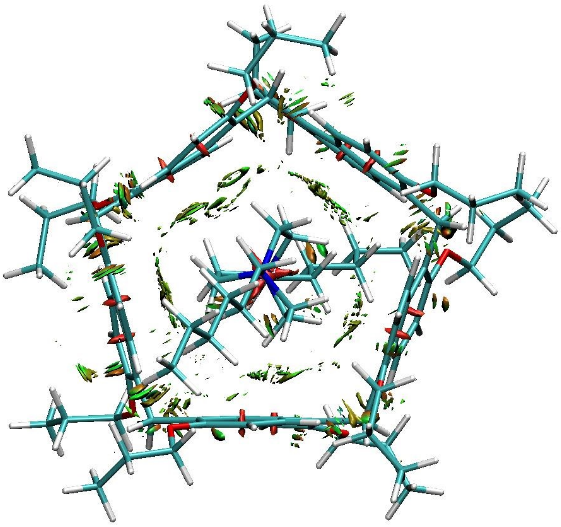

Figure S91. Gradient RDG isosurfaces (0.5) for the noncovalent interaction (NCI) regions in **5^{2+}@ PrS[5]*^{nPr}*** complexes.

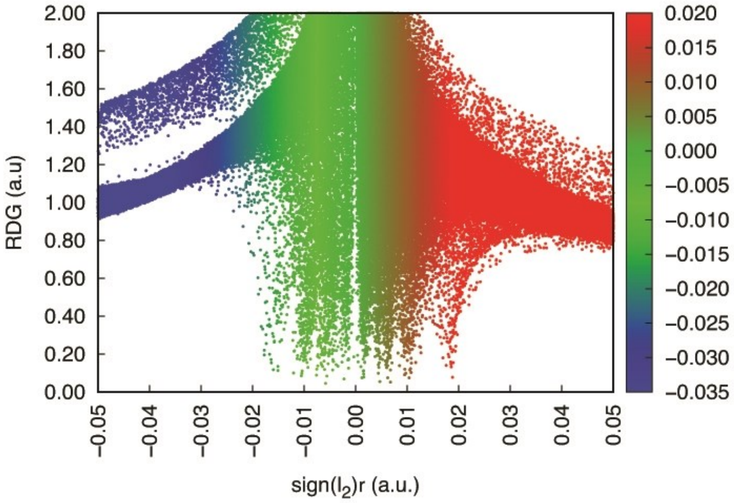

Figure S92. Plot of RDG versus sign(l_2)r for **5^{2+}** @ **PrS[5]*^{nPr}*** complex (NCI-RDG isosurfaces with S = 0.5).

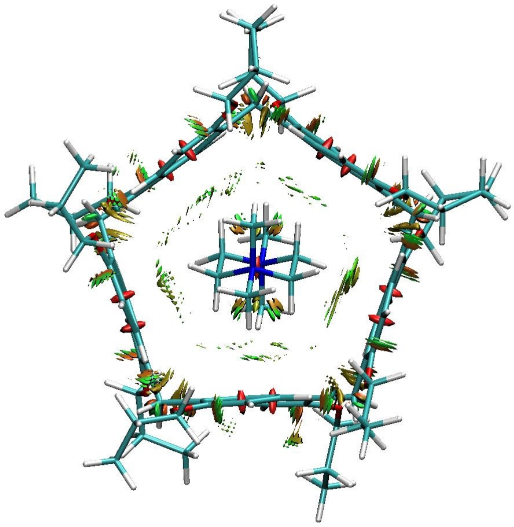

Figure S93. Gradient RDG isosurfaces (0.5) for the noncovalent interaction (NCI) regions in **7^{2+}@ PrS[5]*^{nPr}*** complexes.

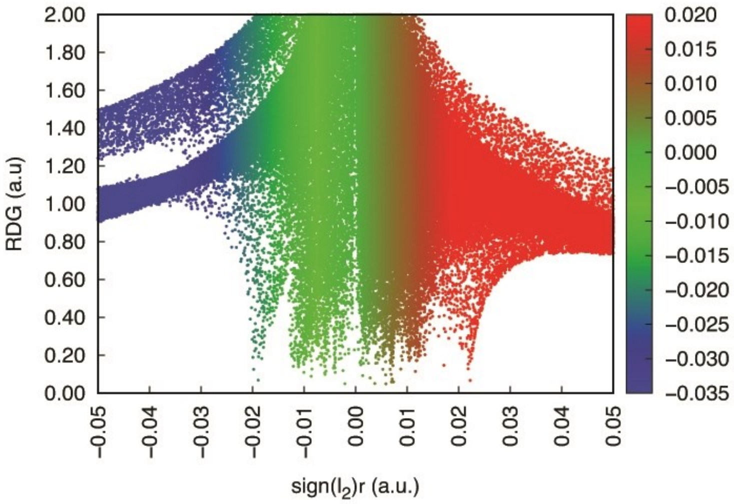

Figure S94. Plot of RDG versus sign(l_2)r for $\mathbf{7^{2+}}$ @ **PrS[5]*^{nPr}*** complex (NCI-RDG isosurfaces with S = 0.5).

Determination of crystallographic structures of prismarenes: PrS[5]^{Et}, PrS[5]^{nPr}, PrS[6]^{Et} and PrS[6]^{nPr} and prismarenes host-guest complexes: 7@PrS[5]^{Me}·(BArF)$_2$, 10@PrS[5]^{Me}·BArF and 6@PrS[6]^{Me}·BArF

Single crystals suitable for X-ray investigations were obtained by slow evaporation of solutions containing the molecules, using various solvents: ethyl acetate/dichloromethane for **PrS[5]^{Et};** methanol/dichloromethane for **PrS[5]^{nPr}** and **PrS[6]^{nPr};** hexane/dichloromethane for **PrS[6]^{Et}** and for the host-guest complexes **7@PrS[5]^{Me}·(BArF)$_2$**, **10@PrS[5]^{Me}·BArF** and **6@PrS[6]^{Me}·BArF**. Data collection was carried out at the Macromolecular crystallography XRD1 beamline of the Elettra synchrotron (Trieste, Italy), employing the rotating-crystal method with a Dectris Pilatus 2M area detector. Single crystals were dipped in paratone cryoprotectant, mounted on a nylon loop and flash-frozen under a nitrogen stream at a 100 K. Diffraction data were indexed and integrated using the XDS package,[15] while scaling was carried out with XSCALE.[16] The anisotropic X-ray diffraction pattern of **6@PrS[6]^{Me}·BArF** crystal was scaled using UCLA-DOE LAB - Diffraction Anisotropy Server.[17] Structures was solved using the SHELXT program[18] and structure refinement was performed with SHELXL-18/3,[19] operating through the WinGX GUI,[20] by full-matrix least-squares (FMLS) methods on F^2. Non-hydrogen atoms were refined anisotropically, with the exception of some disordered groups. Details of the treatment of disorder in each structure are described below. Hydrogen atoms were added at the calculated positions and refined using the riding model. Crystallographic data and refinement details are reported in **Table S5** and **S6**.

PrS[5]^{Et}

The asymmetric unit of the triclinic crystals of **PrS[5]^{Et}** (*P* -1) contains two P5E molecules and two co-crystallized ethyl acetate solvent molecules. In each independent molecule two ethyl groups show two-position disorder with 0.6/0.4 and 0.7/0.3 partial occupations. For three ethyl groups the disorder mainly involved the terminal methyl groups, while in one case the disorder involves the methylene carbon. In order to consider the position disorder of the hydrogen atoms, the methylene carbon atoms of the former three groups and the methyl carbon atom of the latter group were split and constrained with the EXYZ and EADP instructions. All non-hydrogen atoms were refined anisotropically. The structure was refined as a 2-component non-merohedral twin. The twin law (-1, 0, 0; -0.931, 1, -0.112; 0, 0, -1), which corresponds to a two-fold axis about the [-1 2 0] direct lattice direction, were detected with PLATON TwinRotMat. The fraction of overlapped reflections was calculated as 30%. The refinement as a 2-component twin with HKLF 5 card significantly

[15] W. Kabsch *Acta Crystallogr.* 2010, **D66**, 125-132.
[16] W. Kabsch *Acta Crystallogr.* 2010, **D66**, 133-144.
[17] M. Strong, M.R. Sawaya, S. Wang, M. Phillips, D. Cascio, D. Eisenberg *Proc. Natl. Acad. Sci. USA.* 2006, **103**, 8060-8065.
[18] G. M. Sheldrick *Acta Crystallogr.* 2015, **A71**, 3-8
[19] G. M. Sheldrick *Acta Crystallogr.* 2008, **A64**, 112-122.
[20] L. J. Farrugia *J. Appl. Cryst.* 2012, **45**, 849−854.

reduced the R-factor: The R_1 factor decreased by 0.067. The refined BASF factor is 0.32.

PrS[5]^{nPr}

The asymmetric unit of the monoclinic crystals of **PrS[5]^{nPr}** (*P* 21/c) contains one **PrS[5]^{nPr}** molecule only. No co-crystallized solvent molecules are present. The structure exhibits no significant disorder and all atoms were refined at full occupation, with all non-hydrogen atoms refined anisotropically.

PrS[6]^{Et}

The asymmetric unit of the triclinic crystals of **PrS[6]^{Et}** (*P* -1) contains two **PrS[6]^{Et}** molecules and approximately 1.775 co-crystallized dichloromethane solvent molecules (vide infra). Both crystallographically independent molecules show two-position disorder of one ethyl group, which was refined at 0.5/0.5 partial occupancies in both cases. In one case, the disorder mainly involved the terminal methyl group, while in the other it involves the entire fragment. In order to consider the position disorder of the hydrogen atoms, the methylene carbon atom in the former case was split and constrained with the EXYZ and EADP instructions. The carbon atom sites with partial occupancy were refined isotropically. The crystal structure shows the presence of highly disordered co-crystallized solvent dichloromethane molecules in voids present in the crystal packing. The PLATON SQUEEZE procedure was used to remove the electron density related to these highly disordered dichloromethane molecules.[21] The residual electron density of 299 electrons/cell in a total potential solvent area volume of 1249 Å^3 (15.46% of the cell volume) can be attributed to 7.1 dichloromethane solvent molecules.

PrS[6]^{nPr}

The asymmetric unit of the monoclinic crystals of **PrS[6]^{nPr}** (*C* 2/c) contains half **PrS[6]^{nPr}** molecule and a disordered co-crystallized toluene solvent molecule at 0.5 of occupancy factor. Both lie on crystallographic two-fold axes. The **PrS[6]^{nPr}** molecule exhibits no significant disorder, while the toluene molecule exhibits a two positions disorder related by crystallographic symmetry. All non-hydrogen atoms were refined anisotropically. The anisotropic thermal factors of toluene carbon atoms were restrained with the ISOR and SIMU instructions. The structure was refined as a 2-component twin. The twin law (-1, 0, 0; 0, -1, 0; 0.866, 0, 1), which corresponds to a two-fold axis about the [2 0 5] direct lattice direction, were detected with PLATON TwinRotMat. The fraction of overlapped reflections was calculated as 32%. The refinement as a 2-component twin with HKLF 5 card significantly reduced the R-factor: The R_1 factor decreased by 0,062, with a refined BASF factor of 0.37.

7@PrS[5]^{Me}·(BArF)$_2$

[21] A. L. Spek, *Acta Crystallogr. Sect. D Biol. Crystallogr.* 2009, **65**, 148–155.

The asymmetric unit of the monoclinic crystals of **7@PrS[5]*Me*·(BArF)₂** (*P* 2/c) contains a half **7^{2+}@PrS[5]*Me*** host-guest complex, one BArF counter ion and a disordered co-crystallized CH_2Cl_2 solvent molecule at 0.35 occupancy. The host-guest complex lies on a crystallographic two-fold axis. This symmetry axis passes through a methylene bridging atom and the center of the opposite naphthalene ring of the **PrS[5]*Me*** host. The **7^{2+}** guest ion is statistically disordered over two positions related by this crystallographic symmetry operation; while the C2 symmetric **PrS[5]*Me*** host exhibits no significant disorder. The BArF counter ion, located in a general position, exhibits a rotational disorder of two CF_3 groups. Finally, a CH_2Cl_2 solvent molecule is positioned close to a two-fold axis with 0.35 occupancy.

10@PrS[5]^Me·BArF

The asymmetric unit of the centrosymmetric triclinic crystals of **10@PrS[5]*Me*·BArF** contains two **10^{+}@PrS[5]*Me*** host-guest complexes, two BArF counter ion and approximately 1.1 co-crystallized, disordered dichloromethane solvent molecules (vide infra). The structure was refined as a 4-component twin. The twin laws (-1, 0, 0.3; 0, -1, 0.3; 0, 0, 1), (0, 1, -0.3; 1, 0, -0.3; 0, 0, -1) and (0, -1, 0; -1, 0, 0; 0, 0, -1) which correspond to two-fold axes about the [0 0 1], [3 3 -1] and [1 -1 0] direct lattice directions, respectively, were detected with PLATON TwinRotMat. The fraction of overlapped reflections was calculated as about 40% for each of these three twin components. The refinement as a 4-component twin with HKLF 5 card significantly reduced the R-factor: The R_1 factor decreased by 0.061, with refined BASF factors of 0.279, 0.008 and 0.013, respectively. The crystal structure also shows the presence of highly disordered co-crystallized solvent dichloromethane molecules in voids present in the crystal packing. The PLATON SQUEEZE procedure was used to remove the electron density related to these highly disordered dichloromethane molecules. The residual electron density of 185 electrons/cell in a total potential solvent area volume of 1812 $Å^3$ (17.68% of the cell volume) can be attributed to 4.4 dichloromethane solvent molecules. The anisotropic thermal factors of non-H atoms were refined with the restraint cards ISOR_* and SIMU_* because of the low reflections/parameters ratio.

6@PrS[6]^Me·BArF

The asymmetric unit of the orthorhombic crystals of **6@PrS[6]*Me*·BArF** (*C*mca) contains a half **6^{+}@PrS[6]*Me*** host-guest complex, one BArF counter ion with 0.5 occupancy and approximately 3.575 co-crystallized, highly disordered dichloromethane solvent molecules (vide infra). The host-guest complex lies on a crystallographic two-fold axis. This symmetry axis passes through the centers of two opposite naphthalene rings of the **PrS[6]*Me*** host. The 6^{+} guest ion, located on the two-fold axis, show a statistical disorder of two methylene C atoms, while the C2 symmetric **PrS[6]*Me*** host exhibits no significant disorder. The BArF counter ion across a mirror plane and it is severely disordered. This was isotropically refined using geometrical restraints. The crystal structure also shows the presence of highly disordered, co-crystallized solvent dichloromethane molecules in voids present in the crystal packing. The PLATON SQUEEZE procedure was used to remove the electron density related to these highly disordered solvent molecules. The residual electron density of 1201 electrons/cell in a total potential solvent area volume of 8247 $Å^3$ (29.70% of the cell volume) can be attributed to 28.6 dichloromethane solvent molecules. The anisotropic thermal factors of the non-H atoms of the host-guest complex were refined with the restraint cards

ISOR and SIMU because of the low reflections/parameters ratio.

Table S3. Crystal data and structure refinement for prismarenes: **PrS[5]*Et***, **PrS[5]*nPr***, **PrS[6]*Et*** and **PrS[6]*nPr***.

	**PrS[5]*Et***	**PrS[5]*nPr***	**PrS[6]*Et***	**PrS[6]*nPr***
Empirical formula	$C_{75}H_{80}O_{10}$, $C_4H_8O_2$	$C_{85}H_{100}O_{10}$	$C_{90}H_{96}O_{12}$, 1.775 (CH_2Cl_2)	$C_{102}H_{120}O_{12}$, C_7H_8
Formula weight	1229.49	1281.64	1520.41	1630.11
Temperature (K)	100(2)	100(2)	100(2)	100(2)
Wavelength (Å)	0.7	0.7	0.7	0.7
Crystal system	Triclinic	Monoclinic	Triclinic	Monoclinic
Space group	*P* -1	*P* 2_1/c	*P* -1	*C* 2/c
Unit cell dimensions (Å, °)	*a* = 16.42(8)<br>*b* = 20.342(12)<br>*c* = 21.545(17)<br>*α* = 83.045(14)<br>*β* = 80.54(8)<br>*γ* = 67.32(11)	*a* = 10.909(5)<br>*b* = 34.548(3)<br>*c* = 18.627(1)<br>*β* = 91.334(6)	*a* = 19.356(8)<br>*b* = 19.428(5)<br>*c* = 21.732(4)<br>*α* = 96.664(4)<br>*β* = 90.158(18)<br>*γ* = 95.38(2)	*a* = 13.308(9)<br>*b* = 30.108(18)<br>*c* = 23.23(3)<br>*β* = 104.36(16)
Volume (Å^3)	6540(30)	7018(3)	8081(4)	9017(14)
Z	4	4	4	4
ρ_{calcd} (g/cm^3)	1.249	1.213	1.25	1.201
μ (mm^{-1})	0.079	0.075	0.191	0.076
F(000)	2632	2760	3226	3512
Reflections collected	96777	121074	88777	20991
Indep. Reflections	31258	20580	25663	6384
restraints/parameters	0/1705	0/867	0/1861	84/569
GooF	1.017	1.093	1.027	1.036
Final *R* indices [I>2σ(I)]	R_1 = 0.0962<br>wR_2 = 0.2623	R_1 = 0.0762<br>wR_2 = 0.1869	R_1 = 0.0684<br>wR_2 = 0.1895	R_1 = 0.0912<br>wR_2 = 0.2549
R indices (all data)	R_1 = 0.145<br>wR_2 = 0.3057	R_1 = 0.0999<br>wR_2 = 0.2038	R_1 = 0.0987<br>wR_2 = 0.2133	R_1 = 0.1362<br>wR_2 = 0.2983
CCDC code	2025594	2025589	2025583	2025585

Table S4. Crystal data and structure refinement for the prismarene host-guest complexes **7@PrS[5]^{Me}·(BArF)$_2$**, **10@PrS[5]^{Me}·BArF**, and **6@PrS[6]^{Me}·BArF**.

	**7@PrS[5]^{Me}·(BArF)$_2$**	**10@PrS[5]^{Me}·BArF**	**6@PrS[6]^{Me}·BArF**
Empirical formula	$C_{65}H_{60}O_{10}$, $C_8H_20N_2$, 2($C_{32}H_{12}BF_{24}$), 0.7(CH_2Cl_2)	$C_{65}H_{60}O_{10}$, C_4 $H_{12}N$, $C_{32}H_{12}BF_{24}$, 1.1(CH_2Cl_2)	$C_{78}H_{72}O_{12}$, $C_8H_{20}N$, $C_{32}H_{12}BF_{24}$, 3.575(CH_2Cl_2)
Formula weight	2931.28	20131.92	2498.44
Temperature (K)	100(2)	100(2)	100(2)
Wavelength (Å)	0.7	0.7	0.7
Crystal system	Monoclinic	Triclinic	orthorhombic
Space group	*C* 2/*c*	*P* -1	*C* m c a
Unit cell dimensions (Å, °)	a = 38.56(3)<br>b = 17.306(2)<br>c = 22.875(7)<br>β = 120.862(15)	a = 21.686(11)<br>b = 22.528(10)<br>c = 22.906(7)<br>α = 81.367(6)<br>β = 80.862(14)<br>γ = 68.83(3)	a = 34.824(14)<br>b = 30.10(3)<br>c = 26.489(7)
Volume (Å3)	13105(11)	10251(8)	27770(30)
Z	4	4	8
ρ calcd (g/cm3)	1.486	1.317	1.195
μ (mm–1)	0.158	0.163	0.225
F(000)	5966	4177	10289
Reflections collected	121183	82571	79718
Indep. Reflections	19594	23532	6110
restraints/parameters	0/ 998	3486/2470	804/644
GooF	1.029	1.197	1.607
Final R indices [I>2σ(I)]	R_1 = 0.0646<br>wR_2 = 0.1691	R_1 = 0.1565<br>wR_2 = 0.3939	R_1 = 0.1760<br>wR_2 = 0.4399
R indices (all data)	R_1 = 0.0959<br>wR_2 = 0.202	R_1 = 0.2416<br>wR_2 = 0.4607	R_1 = 0.1959<br>wR_2 = 0.4581
CCDC code	2050614	2050617	2050618

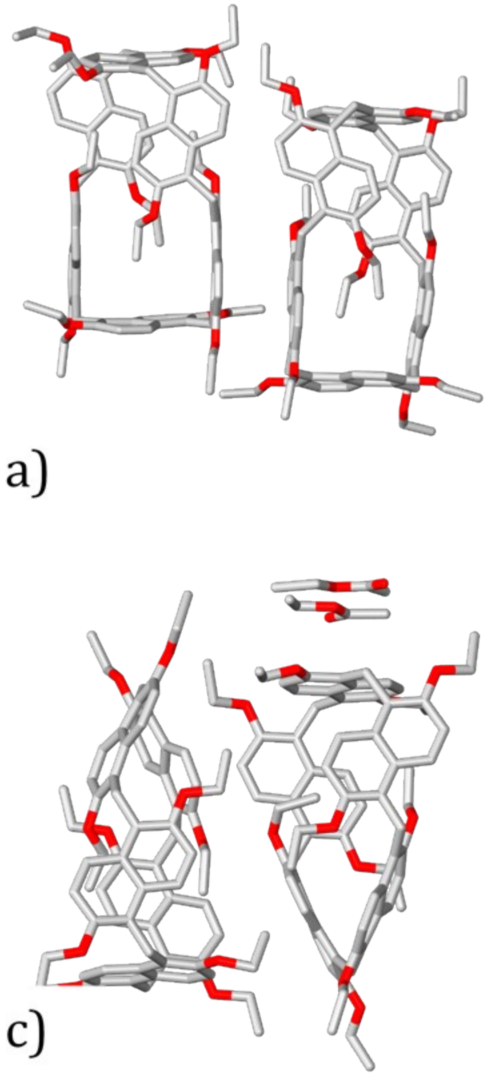

Figure S95. (a-d) Stick representation of the asymmetric units of a) **PrS[6]**^{Et}, b) **PrS[6]**^{nPr}, c) **PrS[5]**^{Et} and d) **PrS[5]**^{nPr}. Hydrogen atoms and one orientation of the disordered alkyl groups and toluene molecule are omitted for clarity. For **PrS[6]**^{nPr} the entire molecule generated by a crystallographic two-fold axis is shown.

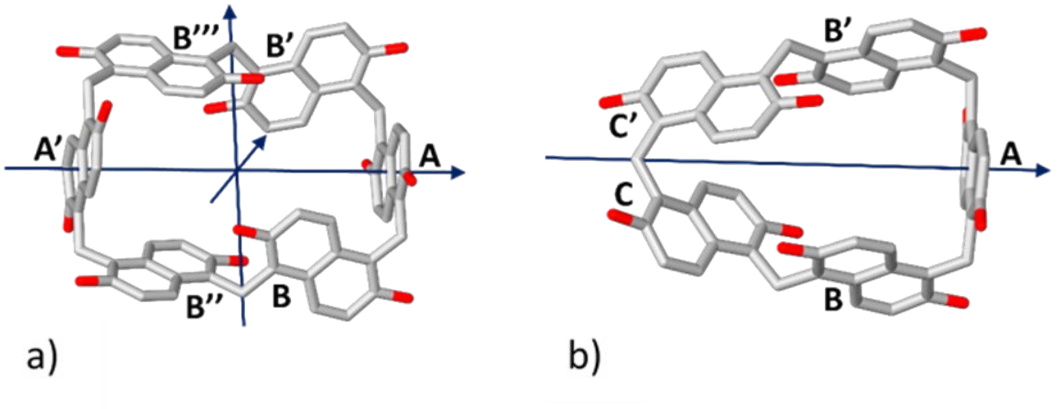

Figure S96. (a-b) Stick representation of (a) the C_2 symmetric **PrS[5]**^{R} and (b) the D_2 symmetric **PrS[6]**^{R} scaffolds. The pseudo two-fold symmetry axes are represented with arrows. The alkyl groups are omitted for clarity.

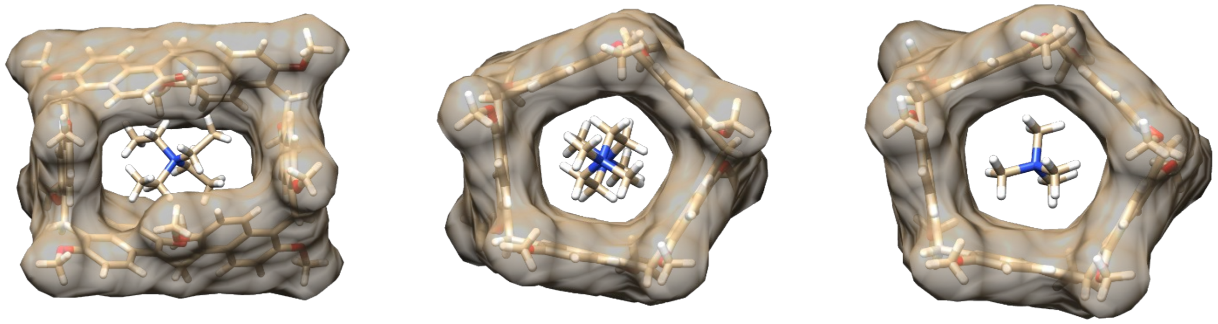

a) b) c)

Figure S97. X-ray structural models of the host-guest complexes **6^+@PrS[6]^{Me}**, **7^{2+}@PrS[5]^{Me}** and **10^+@PrS[5]^{Me}**. The transparent van der Waals surface of the host is shown to illustrate the effective cavities of the prismarens. The counter-ions, solvent and disordered guest atoms are omitted for clarity.

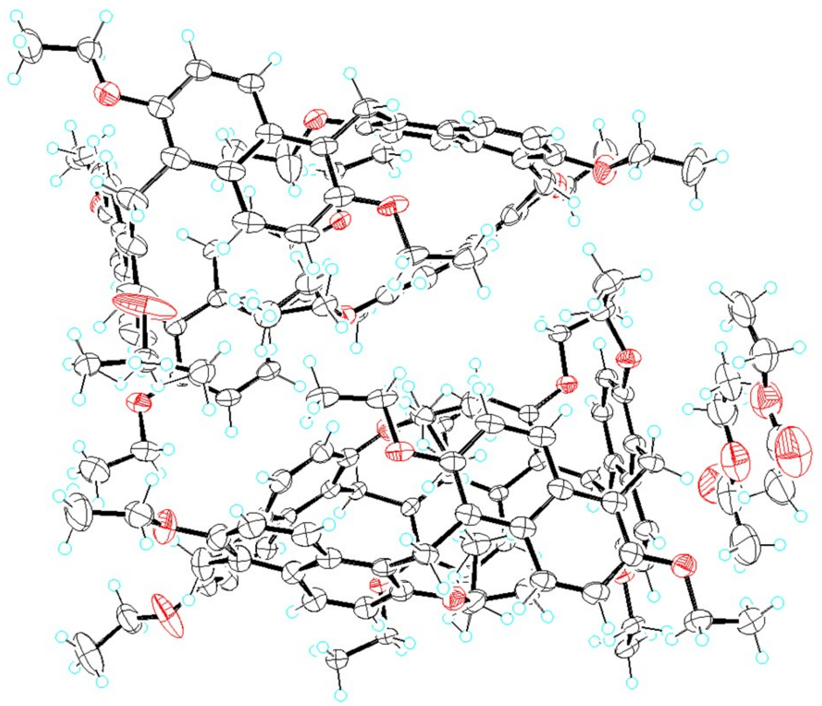

Figure S98. ORTEP drawing of **PrS[5]^{Et}** (thermal *ellipsoids* at the *50% probability* level).

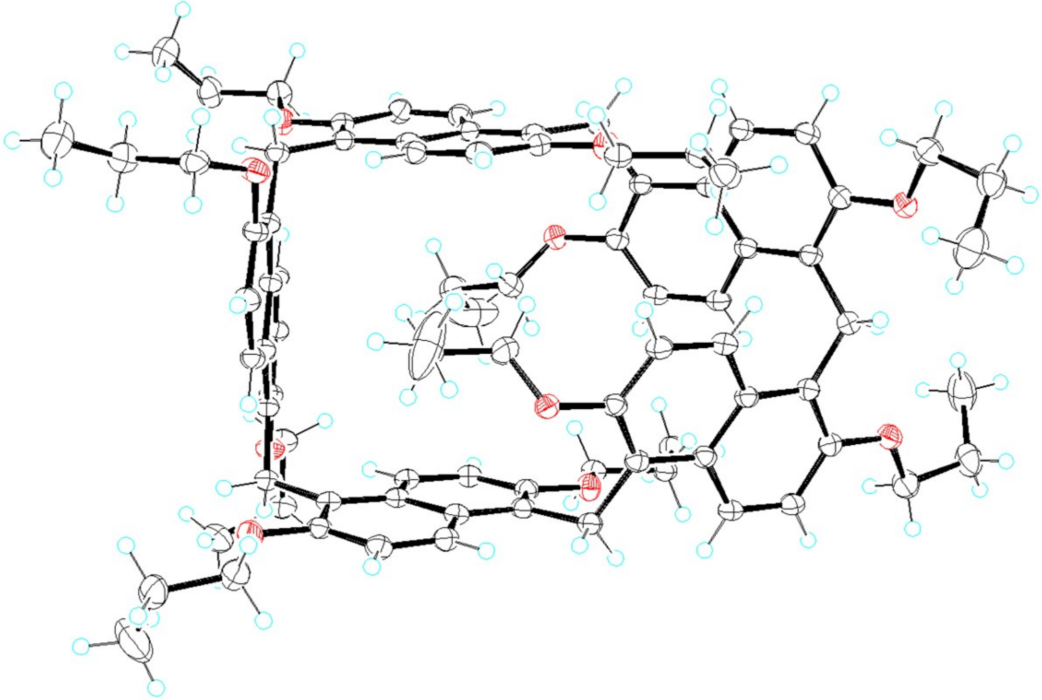

Figure S99. ORTEP drawing of **PrS[5]*nPr*** (thermal *ellipsoids* at the *50% probability* level).

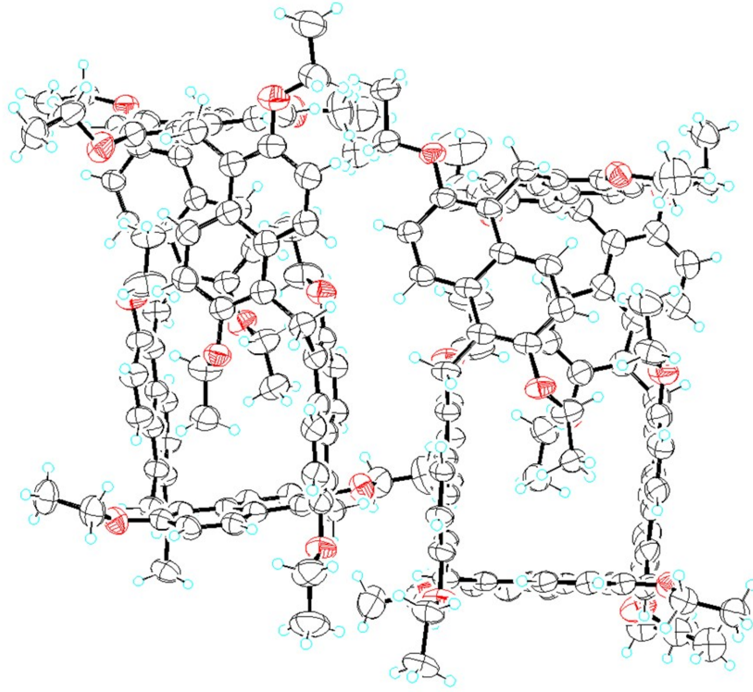

Figure S100. ORTEP drawing of **PrS[6]*Et*** (thermal *ellipsoids* at the *50% probability* level).

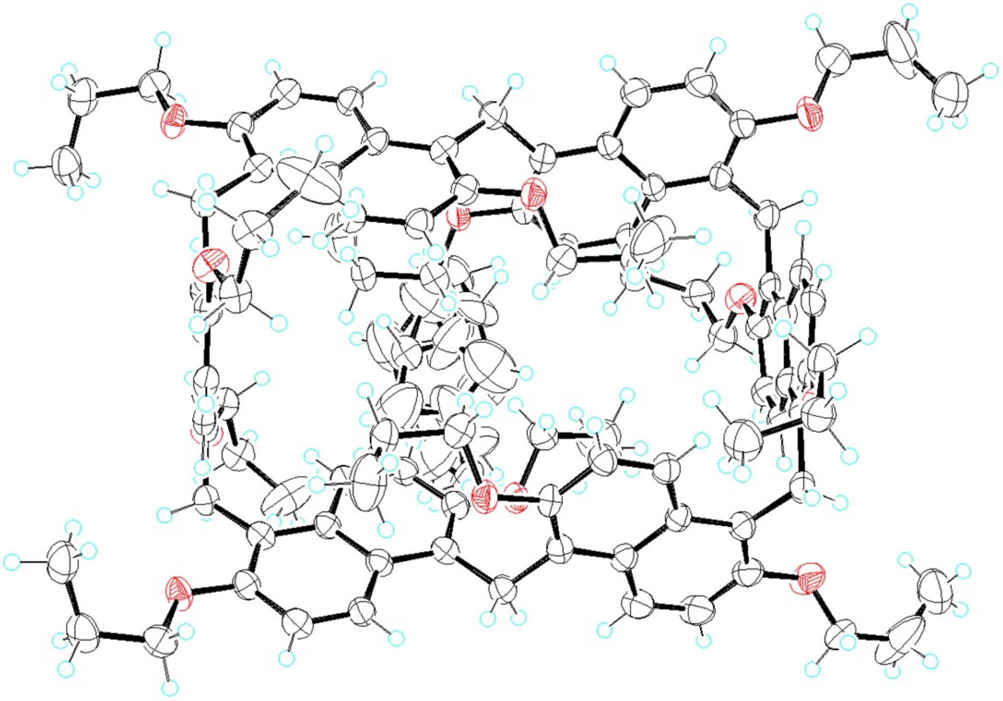

Figure S101. ORTEP drawing of **PrS[6]^{nPr}** (thermal *ellipsoids* at the *50% probability* level).

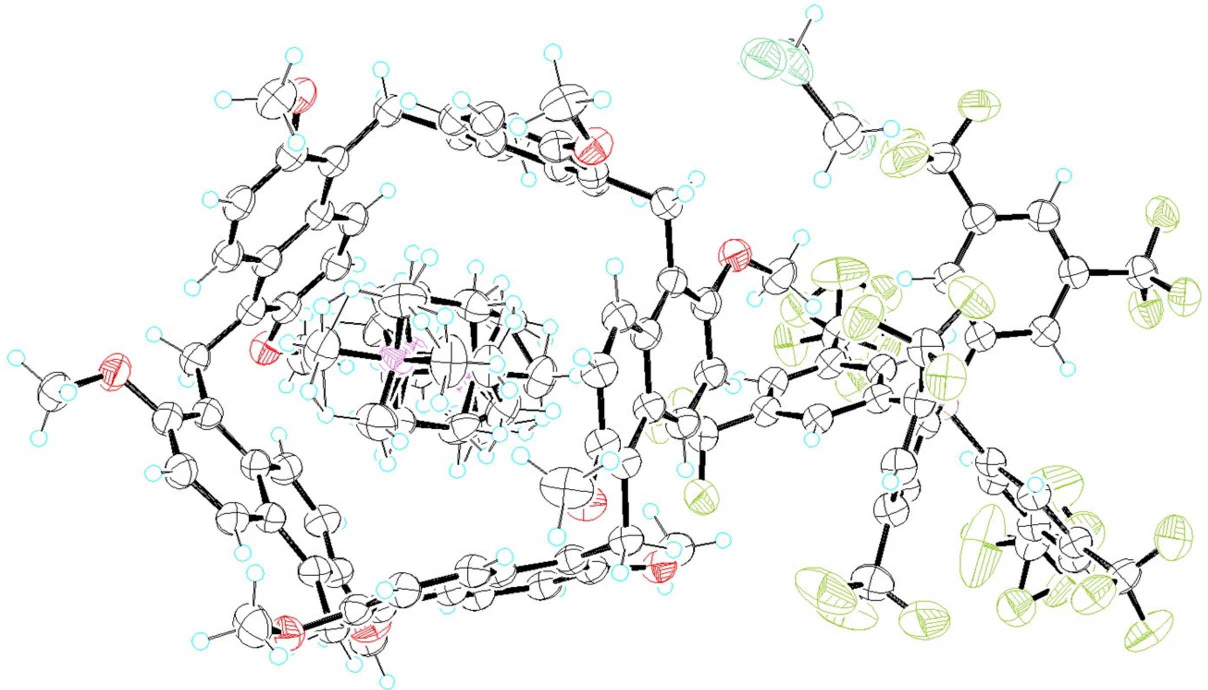

Figure S102. ORTEP drawing of **7@PrS[5]^{Me}·(BArF)$_2$** (thermal *ellipsoids* at the *50% probability* level).

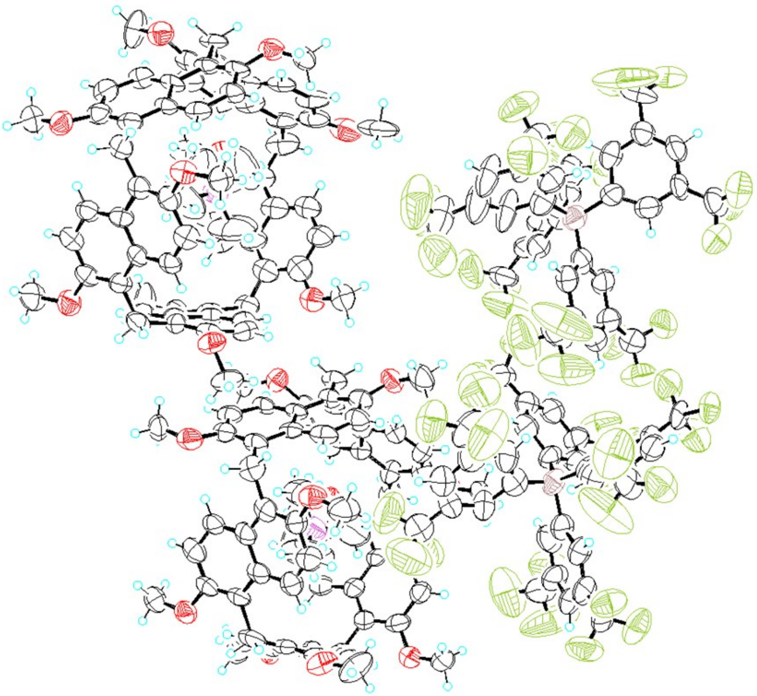

Figure S103. ORTEP drawing of **10@PrS[5]^{Me}·BArF** (thermal *ellipsoids* at the *50% probability* level).

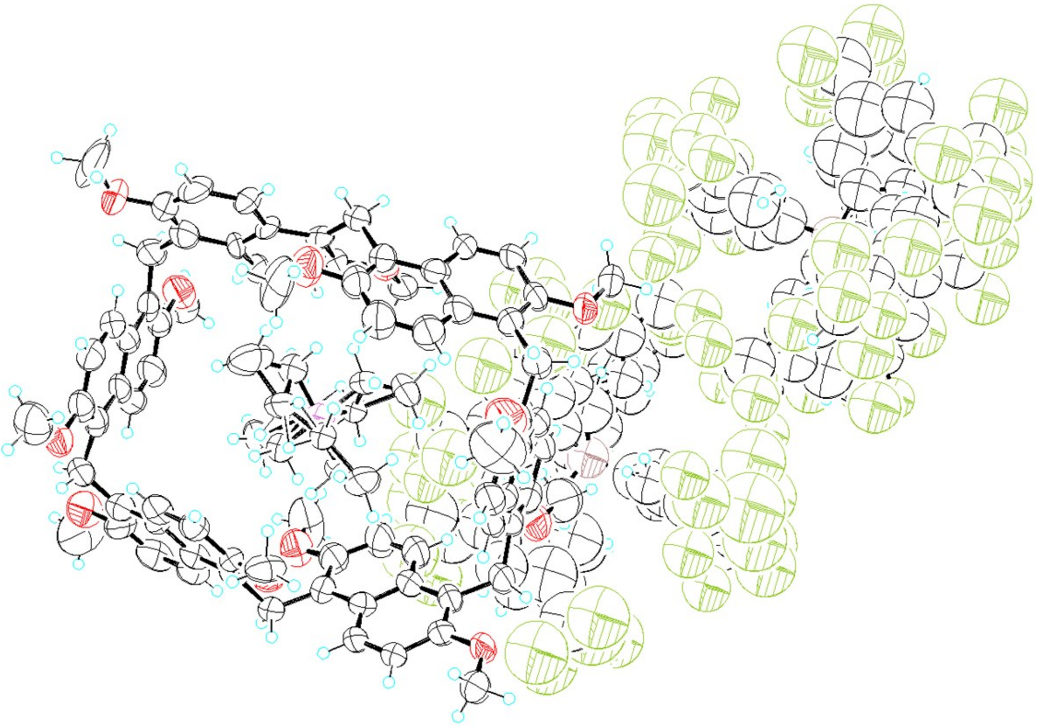

Figure S104. ORTEP drawing of **6@PrS[6]^{Me}·BArF** (thermal *ellipsoids* at the *50% probability* level).

Table S5. Dihedral canting angles between the naphthalene planes and the mean planes of the bridging methylene carbon atoms, observed in the crystal structures of **PrS[5]^{R}** and **PrS[6]^{R}**. Considering an arbitrary "up-side" of the macro-ring, dihedral angles greater than 90° correspond to outward orientations of the plane with respect to this up-side and vice-versa. For the labelling scheme see Figure S89.

	A	B	B′	C	C′
**PrS[5]^{Et}**	90.13(8)	71.13(8)	108.30(8)	140.82(13)	39.15(13)
*	90.35(9)	70.78(8)	110.59(8)	141.81(13)	36.77(14)
**PrS[5]^{nPr}**	91.03(3)	65.89(3)	113.58(3)	139.93(3)	41.14(3)
**α-PrS[5]^{Me}**	95.56(2)	63.97(2)	108.11(2)	132.35(2)	56.37(4)
**β-PrS[5]^{Me}**	94.36(1)	65.28(2)	109.53(2)	132.37(1)	53.68(1)
**γ-PrS[5]^{Me}**	90.00(2)	99.72(2)	80.28(2)	60.32(2)	119.68(2)
**7^{2+}@PrS[5]^{Me}**	90.00(4)	86.80(4)	93.20(4)	91.29(4)	88.71(4)
**10^{+}@PrS[5]^{Me}**	89.88(21)	89.00(19)	90.88(17)	92.97(16)	89.41(18)
*	89.43(21)	89.13(19)	90.45(17)	91.30(22)	89.47(20)

	A	B	B′	B′′	B′′′	A′
**PrS[6]^{Et}**	90.28(5)	43.41(5)	128.30(5)	137.97(6)	39.24(6)	88.46(5)
*	90.00(5)	37.58(6)	136.00(4)	140.14(6)	41.88(6)	89.48(5)
**PrS[6]^{nPr}**	90.00(8)	42.60(8)	137.40(8)	135.47(8)	44.53(8)	90.00(8)
**6^{+}@PrS[6]^{Me}**	90.0(3)	58.1(3)	121.9(3)	122.9(3)	57.1(3)	90.0(3)

* Dihedral angles of the second crystallographic independent molecule.

Table S6. Interior dihedral angles between the naphthalene planes observed in the crystal structures of **PrS[5]**^{R} and **PrS[6]**^{R}. For the labelling scheme see Figure S89

	**A-B**	**B-C**	**C-C′**	**C′-B′**	**B′-A**
**PrS[5]**^{Et}	91.62(8)	101.92(13)	46.54(15)	100.70(13)	94.84(8)
*	90.56(9)	109.97(14)	43.61(14)	98.07(14)	93.32(8)
**PrS[5]**^{nPr}	94.87(4)	94.90(3)	50.21(4)	98.70(4)	91.88(5)
**α-PrS[5]**^{Me}	91.61(2)	100.32(3)	64.74(3)	108.46(3)	98.06(2)
**β-PrS[5]**^{Me}	89.82(2)	101.94(2)	65.56(3)	101.62(2)	101.55(2)
**γ-PrS[5]**^{Me}	111.36(2)	90.86(2)	102.34(2)	90.86(2)	111.36(2)
**7**$^{2+}$**@PrS[5]**^{Me}	107.05(5)	110.48(5)	104.62(5)	110.48(5)	107.05(5)
**10**$^{+}$**@PrS[5]**^{Me}	111.0(2)	103.1(3)	113.7(3)	102.0(3)	110.1(3)
*	113.5(4)	102.1(3)	110.2(2)	110.5(3)	103.6(3)

	**A-B**	**B-B″**	**B″-A′**	**A′-B‴**	**B‴-B′**	**B′-A**
**PrS[6]**^{Et}	91.63(7)	85.31(7)	93.76(7)	90.26(6)	89.44(7)	95.58(6)
*	91.58(7)	77.42(8)	91.13(7)	95.31(6)	85.77(6)	90.70(7)
**PrS[6]**^{nPr}	94.97(17)	86.54(17)	96.23(16)	93.56(17)	83.77(16)	94.97(17)
**6**$^{+}$**@PrS[6]**^{Me}	105.2(3)	107.8(3)	103.1(3)	103.1(3)	107.8(3)	105.2(3)

* Dihedral angles of the second crystallographic independent molecule.

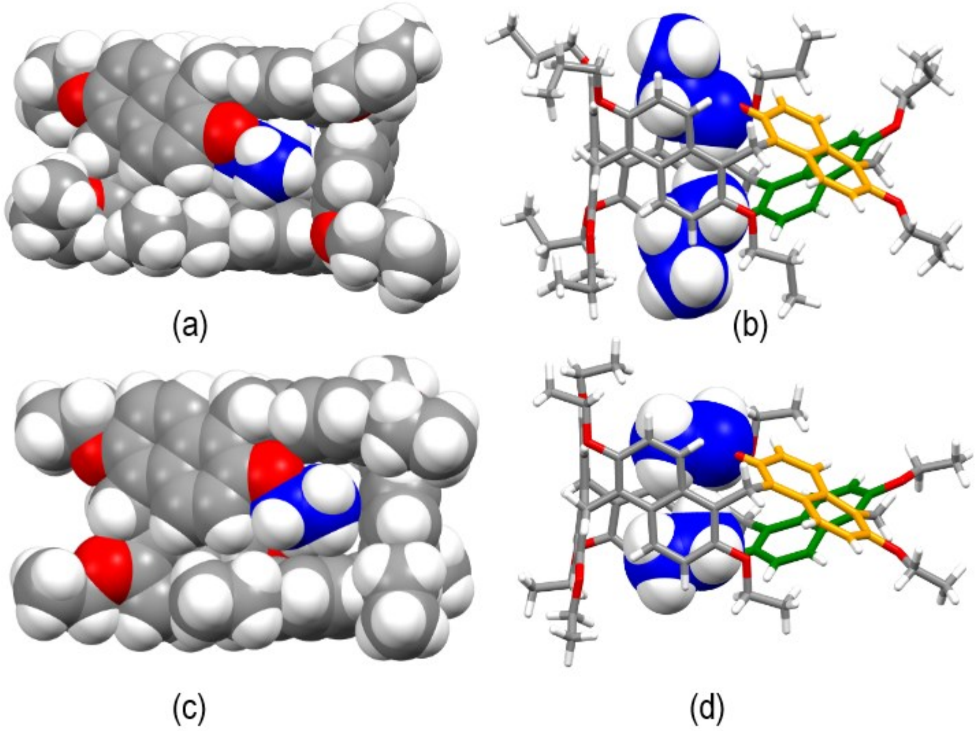

Figure S105. Self-filling of the cavities of of prism[5]arenes as obtained by X-ray structures. (a and b) Different views of **PrS[5]*^nPr^*** . (c and d) Different views of **PrS[5]*^Et^*.**

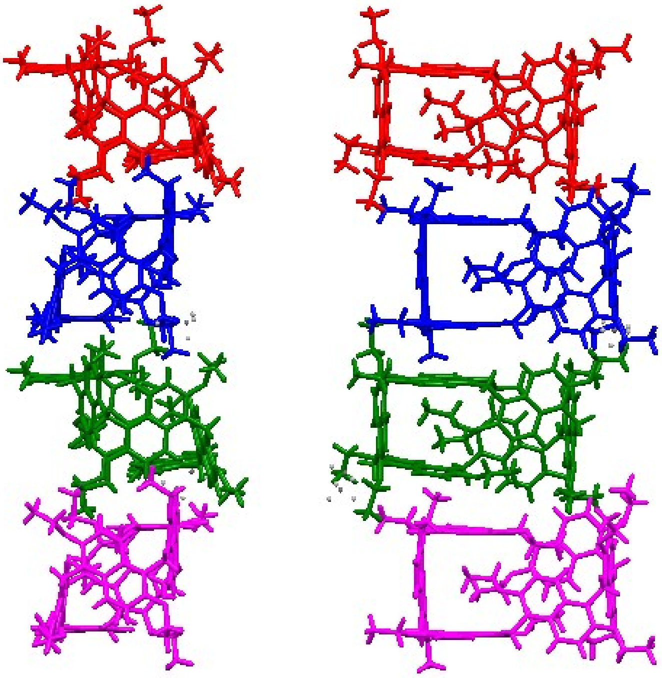

(a) (b) (c) (d)

Figure S106. (a and b) Significant portion of the crystal packing of **PrS[5]**$^{\boldsymbol{Et}}$ viewed along the *a*-axis**.** (c and d) Significant portion of the crystal packing of **PrS[5]**$^{\boldsymbol{nPr}}$ viewed along the *a*-axis.

Potential void volumes of prismatic macro-cycles.

In order to compare the potential cavity of the hosts for complex formation, the surface and volume of the ideal prismatic solids enclosed by the aromatic walls of some strictly related cyclophanes: pillar[5]arene, pillar[6]arene, prism[5]arene, prism[6]arene, and pagoda[4]rene were evaluated (Figure S99). The geometrical parameters used for these calculations were obtained from the CCDC database as mean values of distances and angles observed in the X-ray structures containing the relevant monomeric unit. The volumes of the ideal prismatic solids enclosed by the macrocycles (**V**) were evaluated from the areas of the polyhedron bases (**B**) and the geometric heights (**h**). The potential contact surface areas (**A**) were calculated as the total area of the rectangular prism faces. The opening of the cavity of these macrocycles (Figure S99) was evaluated as the area of the polyhedron base, **B**, namely, pentagon for the prism[5]arene and pillar[5]arene, square for the pagod[4]arene, and hexagon for the prism[6]arene and pillar[6]arene. The opening of the cavity is strictly related first to the number of monomers in the macrocycle and then to the number of fused-rings in the aromatic walls. Thus, the prism[6]arene (52.4 Å^2) shows a wider cavity than the pillar[6]arene (39.1 Å^2) and the prism[5]arene shows a wider cavity than pillar[5]arene (27.3 vs 19.6 Å^2) and pagod[4]arene (19.1 Å^2). The depth of the cavity, evaluated by the geometric height of the prism (**h**), is related to the number of fused-rings in the aromatic walls. Thus the order is pagodarene (10.78 Å), prismarene (9.35 Å), and pillararene (7.76 Å). As a result, the volumes of the cavities of these macrocycles clearly indicate that primarenes have bigger cavities than the corresponding pillararenes: prism[6]arene (490 Å^3) vs. pillar[6]arene (303 Å^3); prism[5]arene (255 Å^3) vs. pillar[5]arene (152 Å^3). The tetrameric pagoda[4]arene (206 Å^3) lie between the two pentamers, prism[5]arene and pillar[5]arene. Another important geometric feature is the potential contact surface areas (**A**) derived from the total area of the rectangular prism faces. The prism[6]arene (252 Å^2) shows the highest surface area, while the prism[5]arene (186 Å^2), pagod[4]arene (188 Å^2) and pillar[6]arene (181 Å^2) have comparable values. Finally, the pillar[5]arene (131 Å^2) exhibits the smallest potential contact area.

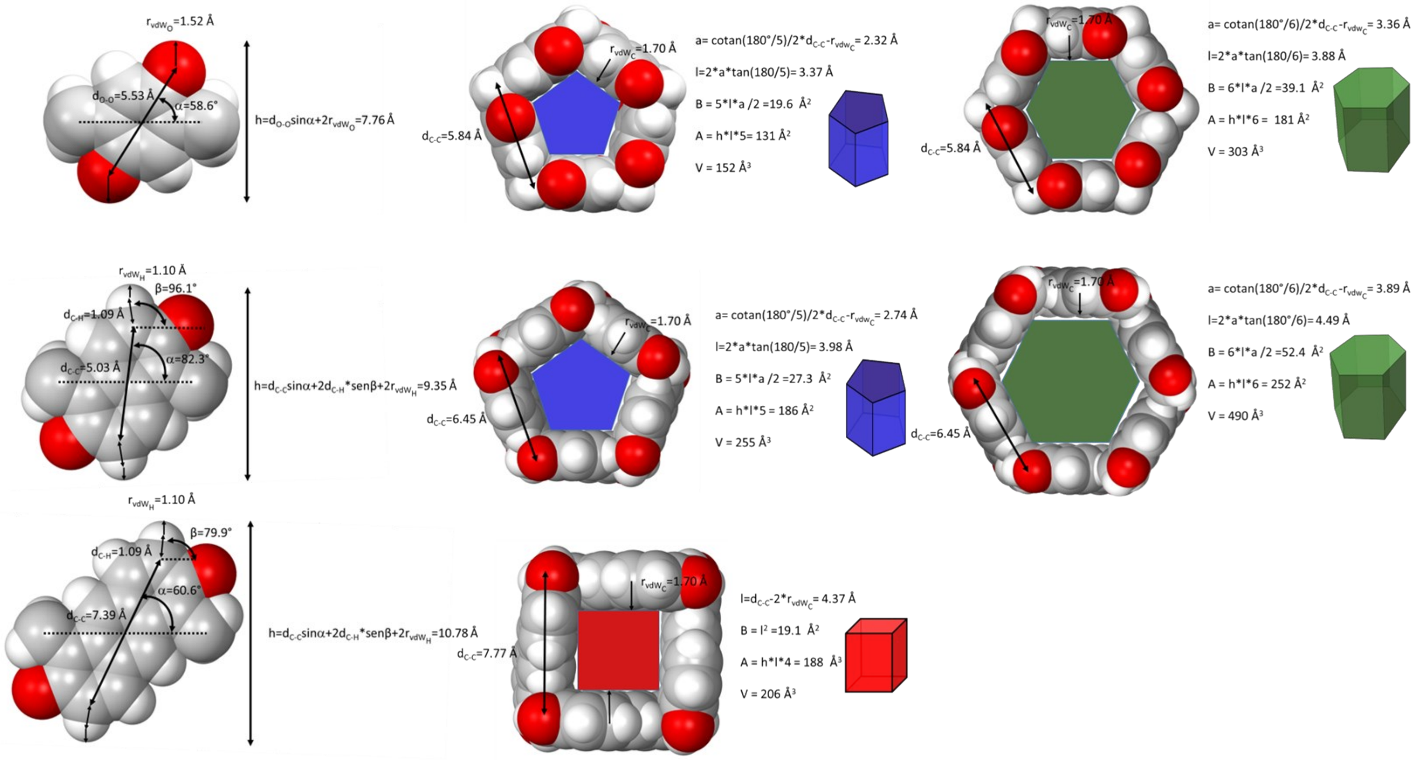

Figure S107. Comparison of the potential void volume in prismatic macro-cycles: Pillarenes, prismarenes and pagodarenes.

Comparison between DFT and Solid-State Structures of PrS[5]^{nPr} and PrS[6]^{nPr}

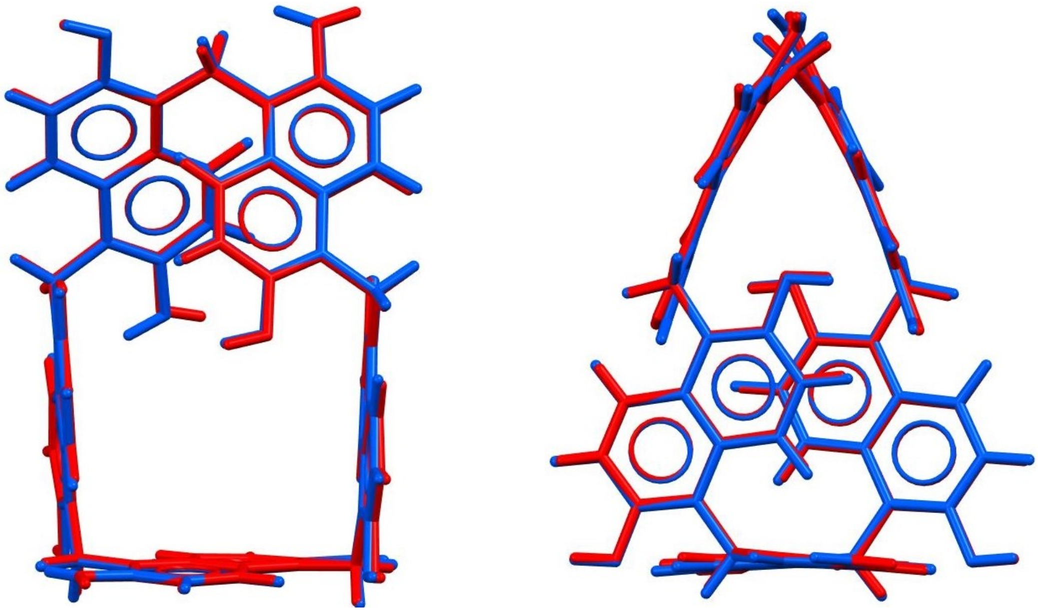

Figure S108. Superimposition of the solid-state structure (red) and DFT minimized structure (blue) of **PrS[5]**^{nPr} (rmsd = 0.07 Å). Propyl groups are omitted for clarity

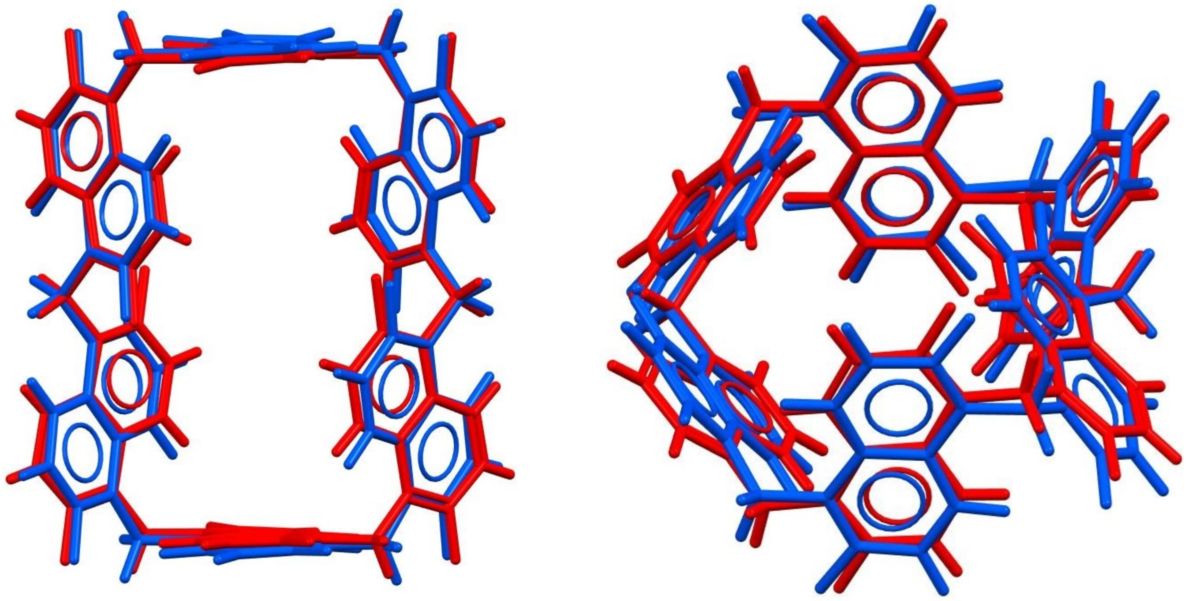

Figure S109. Superimposition of the solid-state structure (red) and DFT minimized structure (blue) of **PrS[6]**^{nPr} (rmsd = 0.34 Å).

HPLC analysis of the cyclocondensation kinetics

All samples were analyzed on 250 x 4.6 mm Waters Spherisorb® 10µm Silica using a gradient of hexane, dichloromethane and acetonitrile as the mobile phase at a flow rate of 1.5 ml/min and injecting 20µL of 10µg/mL solution of reaction mixture in dichloromethane. The gradient used for separation start to mixture 1:1 v:v of hexane-dichloromethane and in 30 minutes was added 5% of acetonitrile. The retention times are as follows: **PrS[5]*Et*** (12.6 min) and **PrS[6]*Et*** (21.0 min).

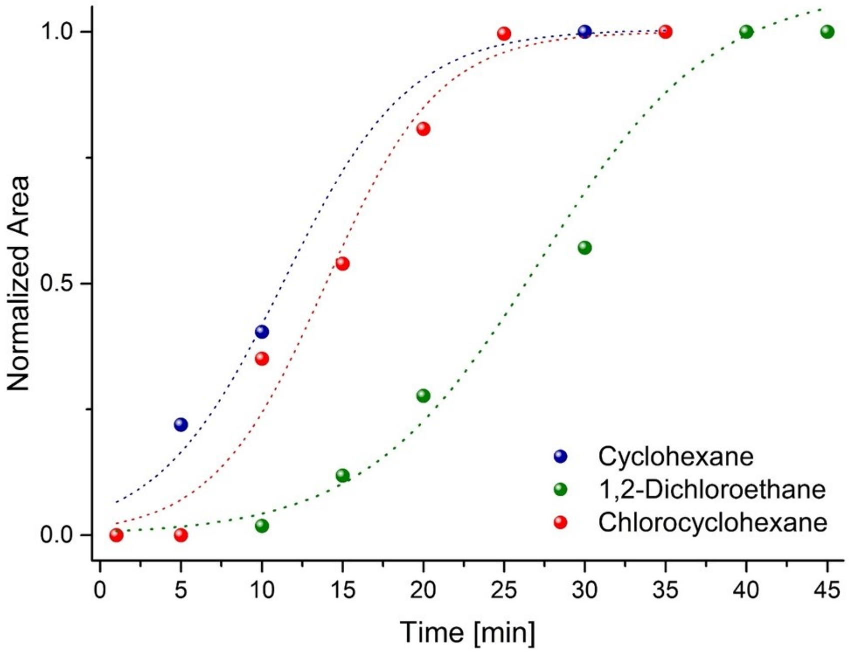

Figure S110: Relationship between time and **PrS[6]*Et*** concentration in the cyclocondensation reactions of **4b** in different solvents.

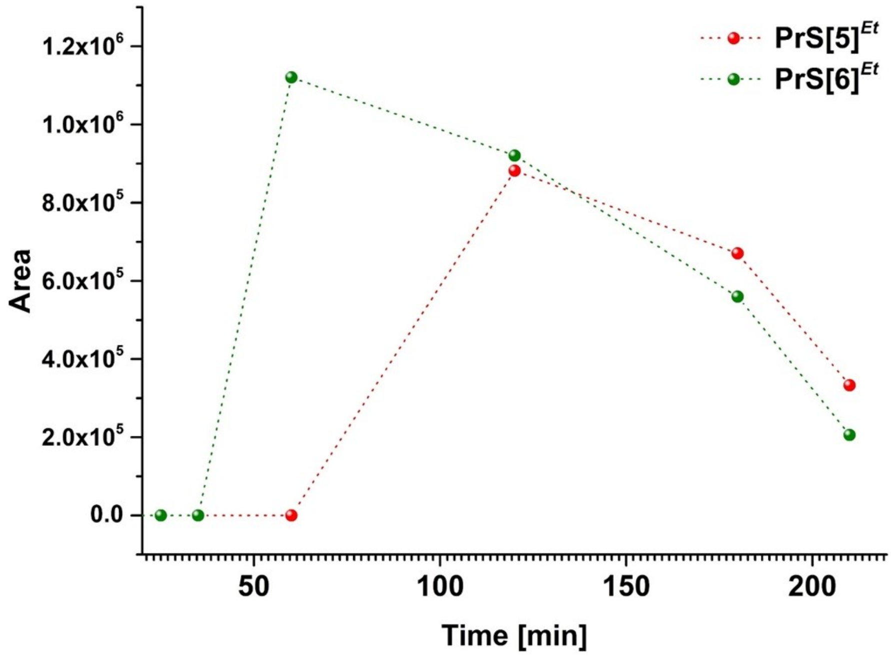

Figure S111: Relationship between time and products distribution in the cyclocondensation reactions of **4b** with templating agent **$5^{2+}\cdot 2I^-$**.
